# Supplementary material for: Targeting NUPR1-dependent stress granules formation to induce synthetic lethality in KrasG12D-driven tumors
Source: EMBO Mol Med. 2024 Feb 15;16(3):4. doi: 10.1038/s44321-024-00032-2 (PMC10940650; doi:10.1038/s44321-024-00032-2)
Supplement: Supplementary file 1 — Appendix [file 44321_2024_32_MOESM1_ESM.pdf]

Table of content:	Page
-Appendix Figure S1: Binding of NUPR1 to either PAR or RNA as monitored by spectroscopic probes.	1
-Appendix Figure S2: ZZW-115-treatment in vivo is safe	2
-Appendix Table S1: Reagents Table	3

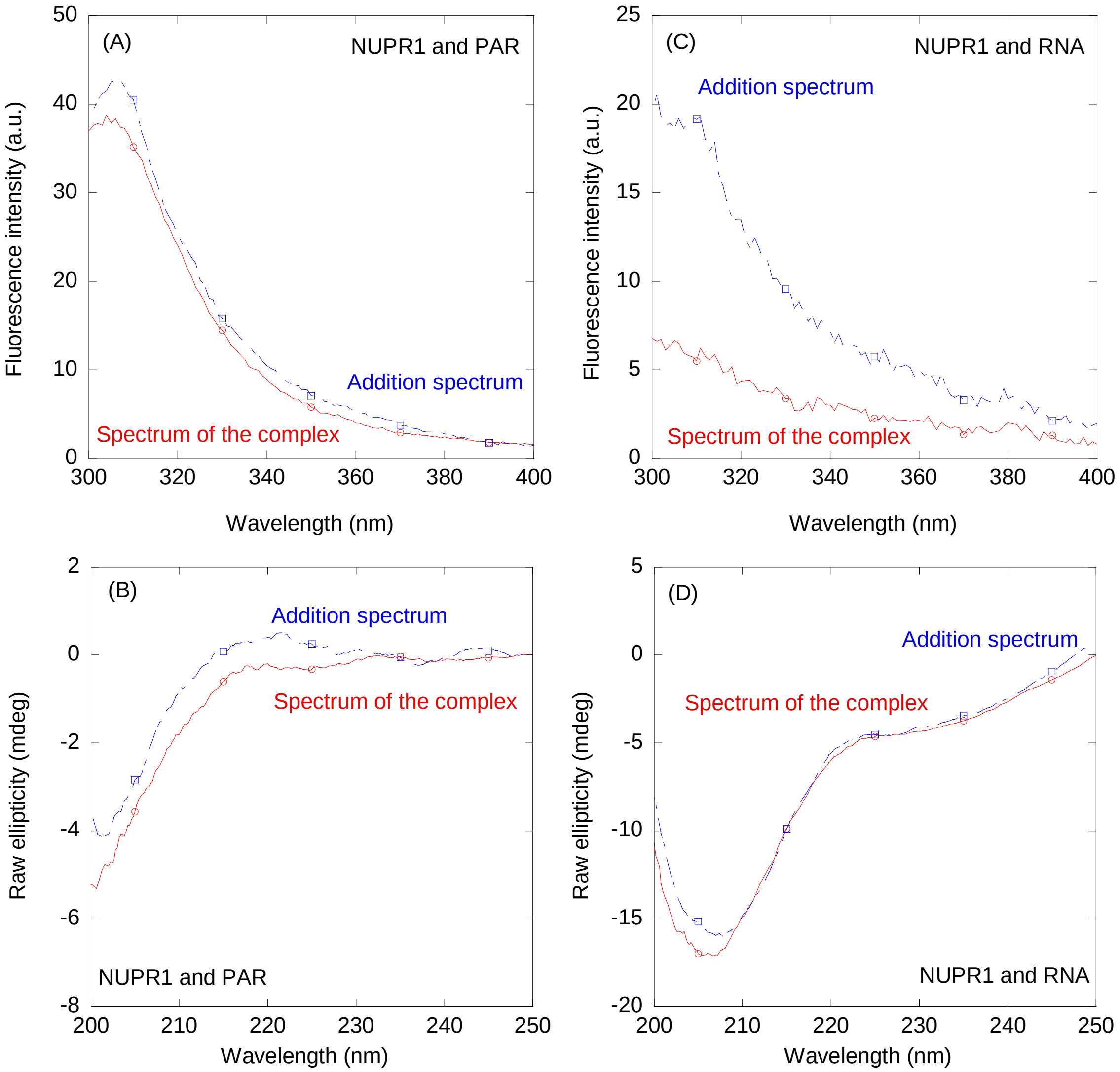

Appendix Figure S1: Binding of NUPR1 to either PAR or RNA as monitored by spectroscopic probes. Fluorescence spectrum of the complex and addition spectrum obtained by the sum of the spectra of NUPR1/PAR (A) or NUPR1/RNA (C). Far-UV CD spectrum of the complex and the addition spectrum obtained by the sum of the spectra of isolated macromolecules for NUPR1/PAR (B) or NUPR1/RNA (D). All experiments were performed at 25°C and 50 mM Tris (pH 7.5).

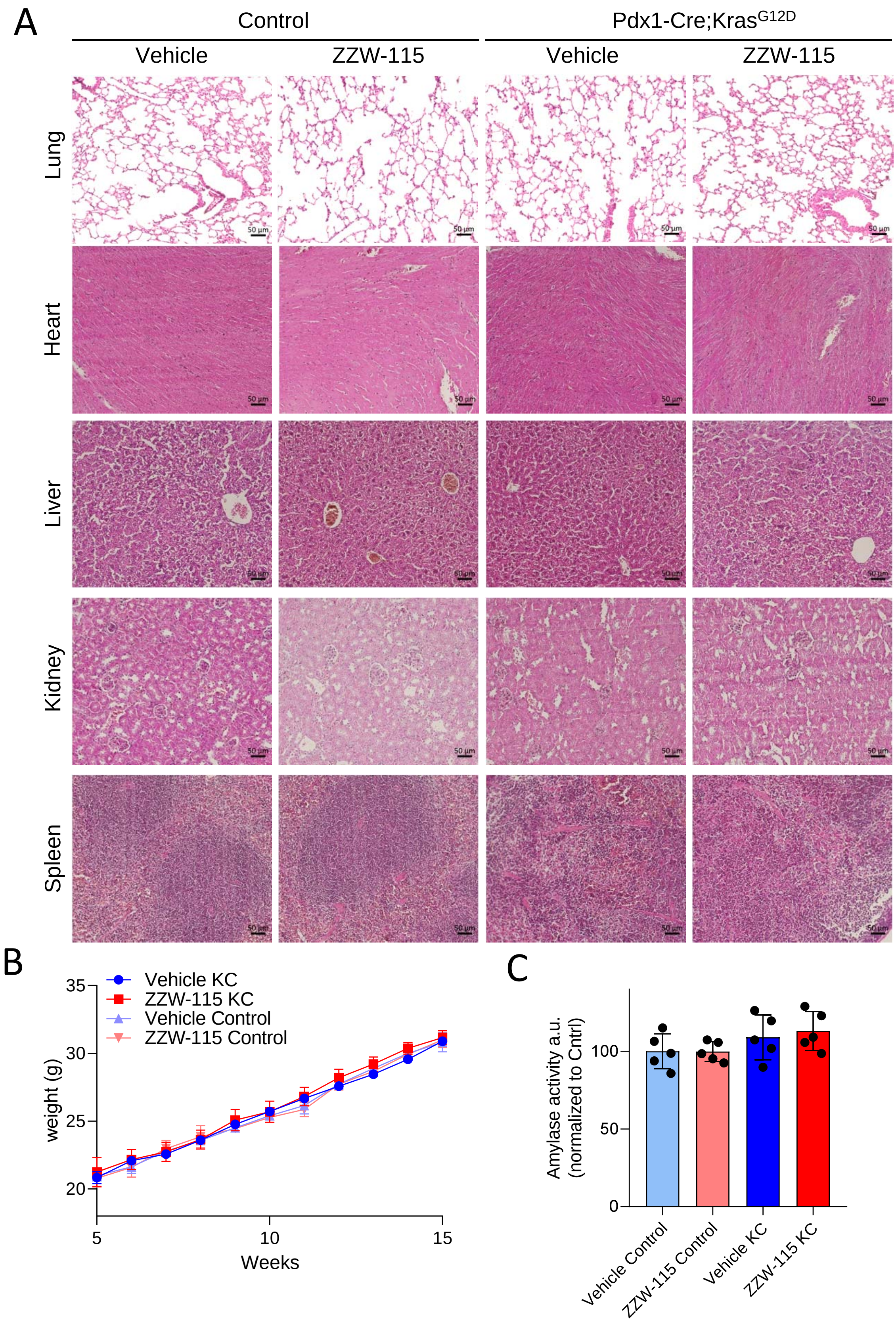

Appendix Figure S2: ZZW-115-treatment *in vivo* is safe. KC were treated daily with the vehicle or 5 mg/Kg of ZZW-115 for 10 weeks. (A) (B) mice body weight were measured once per week. (C) amylase activity levels were measured at the end of the treatment.

Appendix Table S1: Reagents Table

Imidazole, Sigma, Madrid, Spain
Trizma base and acid, Sigma, Madrid, Spain
DNase, Sigma, Madrid, Spain
SIGMAFAST protease tablets, Sigma, Madrid, Spain
NaCl, Sigma, Madrid, Spain
Ni2+-resin, Sigma, Madrid, Spain
β-mercaptoethanol, BioRad, Madrid, Spain
Ampicillin, Apollo Scientific, Stockport, UK
Isopropyl-β-D-1-thiogalactopyranoside, Apollo Scientific, Stockport, UK
Triton X-100, VWR, Barcelona, Spain
TCEP, VWR, Barcelona, Spain
dialysis tubing with a molecular weight cut-off of 3500 Da, VWR, Barcelona, Spain
PAGEmark Tricolor, VWR, Barcelona, Spain
Poly(ADP-ribose) (PAR) polymer, Trevigen, Minneapolis, USA
Amicon centrifugal devices with a molecular weight cut-off of 3 kDa, Millipore, Barcelona, Spain
MiaPaCa-2 cells, American Type Culture Collection (ATCC), USA
Dulbecco's modified Eagle's medium (DMEM), Thermo Fisher Scientific, France, 61965-026
Fetal bovine serum, Hyclone, Fisher Scientific, Loughborough, UK, SV30180.03
Complete media RPMI, Thermo Fisher Scientific, France, 61870010
Doxycycline, Sigma, France, 24390
Amylase Activity Assay Kit, Sigma-Aldrich, France, MAK009
1-cm-pathlength quartz cell, Hellma, Kruibeke, Belgium
Anti-Flag M2-coated beads, Millipore Sigma, France, F3165
GFP-Trap Agarose, Chromotek, GTA-10, Planegg-Martinsried, Germany
Flag peptide, Millipore Sigma, France, F3290
NuPAGE 4%-12% Bis-Tris acrylamide gels, Thermo Fisher Scientific, France
high-sequencing-grade trypsin, Promega, France
Sodium arsenate, Sigma, France, A6756
PrestoBlue™ reagent, Life Technologies, Paris, France
Duolink In Situ, Merck, Darmstadt, Germany
Anti-NUPR1, rabbit, homemade
Anti-G3BP1, mouse, Abcam, Cambridge, UK, ab56574
Anti-Flag, mouse, Millipore Sigma, France, F1804
Goat anti-mouse Alexa Fluor 568, Thermo Fisher Scientific, France, A110314
Donkey anti-rabbit Alexa Fluor 488, Thermo Fisher Scientific, France, A32790
Donkey anti-rabbit Alexa 647, Thermo Fisher Scientific, France, A31573
DAPI, Thermo Fisher Scientific, France, D1306
Mouse anti-G3BP1, Abcam, Cambridge, UK, ab56574
Mouse anti-PABP, Abcam, Cambridge, UK, GR3398464
Rabbit anti-p-EIF2α, Cell signaling Technology, Boston, US, 3398
Rabbit anti-p-ERK1/2, Cell Signaling Technology, Boston, US, 4376
Rabbit anti-p-AKT, Cell Signaling Technology, Boston, US, 9271
Rabbit anti-KRASG12D, Genetex, Irvine, CA, US, GTX635362
Rabbit anti CK19, Abnova; Millipore, Molsheim, France, PAB12676
Rabbit anti amylase, Abcam, Cambridge, UK, ab21156
Mouse anti-cleaved-caspase 3 (Asp175), Affinity Biosciences, Cincinnati, OH HQ, US, BF0711
Alexa 568 goat anti-mouse, Thermo Fisher Scientific, France, A11031
Alexa 488-donkey anti-rabbit, Thermo Fisher Scientific, France, A32790
Anti-ERK1/2, Merck-Calbiochem, Darmstadt, Germany, M5670
Anti-p-ERK1/2, Cell Signaling Technology, Boston, US, 4376

Anti-AKT rabbit, Cell Signaling Technology, Boston, US, 3063
Anti-p-AKT, rabbit, Cell Signaling Technology, Boston, US, 9271
Anti-vinculin, Abcam, Cambridge, UK, ab129002
Anti-β-actin, mouse, Sigma, France, A5316
ECL detection system, Millipore Corp., Bedford, MA
Accutase, Thermo Fisher Scientific, France, A1110501
Pacific-Blue annexin V, BioLegend, San Diego CA, US
Propidium iodide, Miltenyi Biotec, Bergisch Gladbach, Germany
IncucyteS3 Live-Cell Analysis System, Sartorius, Göttingen, Germany
CytoTox-ONE assays, Promega, France, G7890
Caspase-Glo 3/7 assays, Promega, France, G8091
Oligomycin, Millipore Sigma, Saint-Quentin-Fallavier, France
FCCP, Millipore Sigma, Saint-Quentin-Fallavier, France
Rotenone, Millipore Sigma, Saint-Quentin-Fallavier, France
Antimycine A, Millipore Sigma, Saint-Quentin-Fallavier, France
Go Script kit, Promega, France
INTERFERin reagent, Polyplus-transfection, Illkirch, France
siRNA control, Dharmacon, France, D-001810-10-05
Mouse siRNA Nupr1, Dharmacon, France, L-049433-01-0010
Mouse G3bp1 siRNA, Dharmacon, France, L-048735-01-0010
Lipofectamine 3000 Transfection Reagent, Thermo Fisher Scientific, France, L3000015
