# Supplementary material for: Targeting NUPR1-dependent stress granules formation to induce synthetic lethality in KrasG12D-driven tumors
Source: EMBO Mol Med. 2024 Feb 15;16(3):4. doi: 10.1038/s44321-024-00032-2 (PMC10940650; doi:10.1038/s44321-024-00032-2)
Supplement: Supplementary file 2 — Dataset EV1 [file 44321_2024_32_MOESM2_ESM.docx]

**DatasetEV1: Proteins identified to localize to stress granules**

| Gene ID | Protein Name | Description | References |
| --- | --- | --- | --- |
| ABCF1 | ABCF1 | ATP Binding Cassette Subfamily F Member 1 | [1] |
| ABRACL | ABRACL | ABRA C-Terminal Like | [1] |
| ACAP1 | ACAP1 | ArfGAP With Coiled-Coil, Ankyrin Repeat And PH Domains 1 | [1] |
| ACBD5 | ACBD5 | Acyl-CoA Binding Domain Containing 5 | [1] |
| ACTBL2 | ACTBL2 | Beta-actin-like protein 2 | [2] |
| ACTR1A | ACTR1A | Alpha-centractin | [2] |
| ACTR1B | ACTR1B | Beta-centractin | [2] |
| ADAR | ADAR1 | Adenosine Deaminase, RNA Specific | [2] |
| ADD1 | Adducin 1 | Adducin 1 | [1] |
| AGO1 | Argonaute 1/EIF2C1 | Argonaute 1, RISC Catalytic Component | [1] |
| AGO2 | Argonaute 2 | Argonaute 2, RISC Catalytic Component | [1] |
| AHSA1 | AHA1 | Activator Of HSP90 ATPase Activity 1 | [3] |
| AKAP8 | AKAP8 | A-Kinase Anchoring Protein 8 | [4] |
| AKAP9 | AKAP350 | A-Kinase Anchoring Protein 9 | [5] |
| AKAP13 | AKAP13/LBC | A-Kinase Anchoring Protein 13 | [1][4] |
| ALDH18A1 | ALDH18A1 | Delta-1-pyrroline-5-carboxylate synthase | [2] |
| ALG13 | ALG13 | ALG13, UDP-N-Acetylglucosaminyltransferase Subunit | [6] |
| ALPK2 | ALPK2/HAK | Alpha Kinase 2 | [4] |
| AMOTL2 | AMOTL2/LCCP | Angiomotin Like 2 | [4] |
| ANG | Angiogenin | Angiogenin | [7] |
| ANKHD1 | ANKHD1 | Ankyrin Repeat and KH Domain Containing 1 | [6] |
| ANKRD17 | ANKRD17/MASK2/GTAR | Ankyrin Repeat Domain 17 | [1][6] |
| ANP32E | ANP32E | Acidic leucine-rich nuclear phosphoprotein 32 family member E | [2] |
| ANXA1 | ANXA1 | Annexin A1 | [2] |
| ANXA6 | ANXA6 | Annexin 6 | [2] |
| ANXA7 | ANXA7 | Annexin 7 | [2][1] |
| ANXA11 | ANXA11 | Annexin 11 | [1] |
| APEX1 | APEX1 | DNA-(apurinic or apyrimidinic site) lyase | [2] |
| APOBEC3C | APOBEC3C | Apolipoprotein B mRNA Editing Enzyme Catalytic Subunit 3C | [1][4] |
| APOBEC3G | APOBEC3G | Apolipoprotein B mRNA Editing Enzyme Catalytic Subunit 3G | [8] |
| AQR | AQR/IBP160 | Aquarius Intron-Binding Spliceosomal Factor | [1] |
| ARID2 | ARID2/BAF200 | AT-Rich Interaction Domain 2 | [4] |
| ARMC6 | ARMC6 | Armadillo Repeat Containing 6 | [1] |
| ARPC1B | ARPC1B | Actin-related protein 2/3 complex subunit 1B | [2] |
| ASCC1 | ASCC1 | Activating Signal Cointegrator 1 Complex Subunit 1 | [1][6] |
| ASCC3 | ASCC3 | Activating Signal Cointegrator 1 Complex Subunit 3 | [6] |
| ATAD2 | ATAD2 | ATPase family AAA domain-containing protein 2 | [2] |
| ATAD3A | ATAD3A | ATPase family AAA domain-containing protein 3A | [2] |
| ATG3 | ATG3 | Autophagy Related 3 | [1] |
| ATP5A1 | ATP5A1 | ATP synthase subunit alpha, mitochondrial | [2] |
| ATP6V1G1 | ATP6V1G1/ATP6G | ATPase H+ Transporting V1 Subunit G1 | [1] |
| ATXN2 | Ataxin 2 | Ataxin 2 | [1][2][4][6] |
| ATXN2L | Ataxin-2 like | Ataxin 2 Like | [1][2][4][6] |
| BAG3 | BAG3 | BAG family molecular chaperone regulator 3 | [2] |
| BANF1 | BANF1 | Barrier-to-autointegration factor | [2] |
| BAZ1B | BAZ1B | Bromodomain Adjacent To Zinc Finger Domain 1B | [4] |
| BAZ2A | BAZ2A | Bromodomain Adjacent To Zinc Finger Domain 2A | [4] |
| BCCIP | BCCIP | BRCA2 And CDKN1A Interacting Protein | [1] |
| BCLAF1 | BCLAF1 | BCL2 Associated Transcription Factor 1 | [1] |
| BICC1 | BICC1 | BicC Family RNA Binding Protein 1 | [6] |
| BIRC2 | BIRC2/CIAP1 | Baculoviral IAP Repeat Containing 2 | [4] |
| BLM | BLM | BLM RecQ Like Helicase | [4] |
| BOD1L1 | BOD1L1/FAM44A | Biorientation Of Chromosomes In Cell Division 1 Like 1 | [4] |
| BOLL | BOULE | Boule Homolog, RNA Binding Protein | [9] |
| BRAT1 | BRAT1 | BRCA1-associated ATM activator 1 | [2] |
| BRF1 | BRF1 | BRF1, RNA Polymerase III Transcription Initiation Factor Subunit | [10] |
| BTG3 | BTG3 | BTG Anti-Proliferation Factor 3 | [6] |
| C2CD3 | C2CD3 | C2 Calcium Dependent Domain Containing 3 | [1] |
| C9orf72 | C9orf72 | Uncharacterized protein C9orf72 | [11] |
| C15orf52 | C15orf52 | Uncharacterized protein C15orf52 | [2] |
| C20orf27 | C20orf72 | Chromosome 20 Open Reading Frame 27 | [1] |
| CALML5 | CALML5 | Calmodulin-like protein 5 | [2] |
| CALR | Calreticulin/CRT | Calreticulin | [12] |
| CAMSAP1 | CAMSAP1 | Calmodulin Regulated Spectrin Associated Protein 1 | [4] |
| CAP1 | CAP1 | Adenylyl cyclase-associated protein 1 | [2] |
| CAPRIN1 | Caprin-1 | Cell Cycle Associated Protein 1 | [1][2][4][5][6] |
| CAPZA2 | CAPZA2 | F-actin-capping protein subunit alpha-2 | [2] |
| CAPZB | CAPZB | Capping Actin Protein Of Muscle Z-Line Subunit Beta | [4] |
| CARHSP1 | CARHSP1 | Calcium-regulated heat stable protein 1 | [2] |
| CASC3 | MLN51/BTZ | Cancer Susceptibility 3 | [1][4][6] |
| CBFB | CBFB | Core-binding factor subunit beta | [2] |
| CBS | CBS | Cystathionine Beta-Synthase | [4] |
| CBX1 | CBX1 | Chromobox protein homolog 1 | [2] |
| CBX3 | CBX3 | Chromobox protein homolog 3 | [4] |
| CCAR1 | CARP-1 | Cell Division Cycle and Apoptosis Regulator 1 | [4][5] |
| CCDC9 | CCDC9 | Coiled-Coil Domain Containing 9 | [4] |
| CCDC9B | CCDC9B | Coiled-Coil Domain Containing 9B | [4] |
| CCDC85C | CCDC85C | Coiled-Coil Domain Containing 85C | [1] |
| CCDC124 | CCDC124 | Coiled-Coil Domain Containing 124 | [1] |
| CCT3 | CCT3 | T-complex protein 1 subunit gamma | [2] |
| CCT6A | CCT6A | T-complex protein 1 subunit zeta | [2] |
| CDC5L | CDC5L | Cell division cycle 5-like protein | [2] |
| CDC20 | CDC20 | Cell Division Cycle 20 | [4] |
| CDC37 | CDC37 | Cell Division Cycle 37 | [3] |
| CDC73 | CDC73 | Parafibromin | [2] |
| CDK1 | CDK1 | Cyclin-dependent kinase 1 | [2] |
| CDK2 | CDK2 | Cyclin Dependent Kinase 2 | [68] |
| CDV3 | CDV3 | CDV3 Homolog | [1] |
| CELF1 | CUGBP1 | CUGBP Elav-Like Family Member 1 | [1][2][4][6] |
| CELF2 | CUGBP2/BRUNOL3 | CUGBP Elav-Like Family Member 2 | [1] |
| CELF3 | CUGBP3/BRUNOL1 | CUGBP Elav-Like Family Member 3 | [1] |
| CENPB | CENPB | Major centromere autoantigen B | [2] |
| CENPF | CENPF | Centromere Protein F | [4] |
| CEP78 | CEP78/CRDHL | Centrosomal Protein 78 | [1] |
| CEP85 | CEP85/CCDC21 | Centrosomal Protein 78 | [6] |
| CERKL | Ceramide-Kinase Like | Ceramide Kinase Like | [13] |
| CFL1 | Cofilin-1 | Cofilin-1 | [2] |
| CHCHD3 | CHCHD3 | Coiled-coil-helix-coiled-coil-helix domain-containing protein 3, mitochondrial | [2] |
| CHORDC1 | CHORDC1/CHP1 | Cysteine and histidine-rich domain-containing protein 1 | [2] |
| CIRBP | CIRP | Cold Inducible RNA Binding Protein | [1][4] |
| CIT | CIT | Citron Rho-interacting kinase | [2] |
| CLIC4 | CLIC4 | Chloride intracellular channel protein 4 | [2] |
| CLNS1A | CLNS1A | Chloride Nucleotide-Sensitive Channel 1A | [1] |
| CLPP | CLPP | Caseinolytic Mitochondrial Matrix Peptidase Proteolytic Subunit | [1] |
| CNBP | ZNF9 | CCHC-Type Zinc Finger Nucleic Acid Binding Protein | [4] |
| CNN3 | CNN3 | Calponin-3 | [2] |
| CNOT1 | CNOT1/CCR4 | [CCR4-Not Transcription Complex Subunit 1](https://en.wikipedia.org/wiki/CCR4-Not) | [2][6] |
| CNOT2 | CNOT2 | [CCR4-Not Transcription Complex Subunit 2](https://en.wikipedia.org/wiki/CCR4-Not) | [6] |
| CNOT3 | CNOT3 | [CCR4-Not Transcription Complex Subunit 3](https://en.wikipedia.org/wiki/CCR4-Not) | [6] |
| CNOT4 | CNOT4 | [CCR4-Not Transcription Complex Subunit 4](https://en.wikipedia.org/wiki/CCR4-Not) | [6] |
| CNOT6 | CNOT6 | [CCR4-Not Transcription Complex Subunit 6](https://en.wikipedia.org/wiki/CCR4-Not) | [6] |
| CNOT6L | CNOT6L | [CCR4-Not Transcription Complex Subunit 6L](https://en.wikipedia.org/wiki/CCR4-Not) | [6] |
| CNOT7 | CNOT7 | [CCR4-Not Transcription Complex Subunit 7](https://en.wikipedia.org/wiki/CCR4-Not) | [6] |
| CNOT8 | CNOT8 | [CCR4-Not Transcription Complex Subunit 8](https://en.wikipedia.org/wiki/CCR4-Not) | [6] |
| CNOT9 | CNOT9 | [CCR4-Not Transcription Complex Subunit 9](https://en.wikipedia.org/wiki/CCR4-Not) | [6] |
| CNOT10 | CNOT10 | [CCR4-Not Transcription Complex Subunit 10](https://en.wikipedia.org/wiki/CCR4-Not) | [6] |
| CNOT11 | CNOT11 | [CCR4-Not Transcription Complex Subunit 11](https://en.wikipedia.org/wiki/CCR4-Not) | [6] |
| CORO1B | CORO1B | Coronin-1B | [2] |
| CPB2 | Carboxypeptidase B2 | Carboxypeptidase B2 | [74] |
| CPEB1 | CPEB | Cytoplasmic Polyadenylation Element Binding Protein 1 | [75] |
| CPEB4 | CPEB4 | Cytoplasmic Polyadenylation Element Binding Protein 4 | [1][4][6] |
| CPSF3 | CPSF3 | Cleavage and polyadenylation specificity factor subunit 3 | [2] |
| CPSF6 | CPSF6 | Cleavage and polyadenylation specificity factor subunit 6 | [2] |
| CPSF7 | CPSF7 | Cleavage and polyadenylation specificity factor subunit 7 | [2] |
| CPVL | CPVL | Carboxypeptidase, Vitellogenic Like | [6] |
| CRKL | CRKL | CRK Like Proto-Oncogene, Adaptor Protein | [1] |
| CROCC | CROCC | Ciliary Rootlet Coiled-Coil, Rootletin | [1] |
| CRYAB | CRYAB | Alpha-crystallin B chain | [2] |
| CRYBG1 | CRYBG1 | Crystallin Beta-Gamma Domain Containing 1 | [4] |
| CSDE1 | CSDE1 | Cold shock domain-containing protein E1 | [1][2][4][6] |
| CSE1L | CSE1L/XPO2/Exportin-2 | Exportin-2 | [2] |
| CSNK2A1 | Casein Kinase 2 alpha | Casein Kinase 2 Alpha 1 | [14] |
| CSTB | Cystatin B | Cystatin B | [1] |
| CSTF1 | CSTF1 | Cleavage stimulation factor subunit 1 | [2] |
| CTNNA2 | CTNNA2 | Catenin alpha-2 | [2] |
| CTNND1 | CTNND1 | Catenin delta-1 | [2] |
| CTTNBP2NL | CTTNBP2NL | CTTNBP2 N-terminal-like protein | [2] |
| CWC22 | CWC22 | Pre-mRNA-splicing factor CWC22 homolog | [2] |
| DAZAP1 | DAZAP1 | DAZ-associated protein 1 | [1][2][4][6] |
| DAZAP2 | PRTB | DAZ Associated Protein 2 | [15] |
| DAZL | DAZL1 | Deleted In Azoospermia Like | [16] |
| DCD | DCD | Dermcidin | [2] |
| DCP1A | DCP1a | Decapping mRNA 1a | [1][2] |
| DCP1B | DCP1b | Decapping mRNA 1b | [1][4] |
| DCP2 | DCP2 | Decapping mRNA 2 | [6] |
| DCTN1 | DCTN1 | Dynactin subunit 1 | [2] |
| DDX1 | DEAD box protein 1 | DEAD-Box Helicase 1 | [1][2][4][6] |
| DDX3 | DEAD box protein 3 | DEAD-Box Helicase 3 | [2] |
| DDX3X | DDX3X | DEAD-Box Helicase 3, X-Linked | [1][4][6] |
| DDX3Y | DDX3Y | DEAD-Box Helicase 3, Y-Linked | [1] |
| DDX6 | DEAD box protein 6 | DEAD-Box Helicase 6 | [1][2][4][6][8] |
| DDX11 | DEAD box protein 11 | DEAD-Box Helicase 11 | [4] |
| DDX19A | DDX19A | ATP-dependent RNA helicase DDX19A | [2] |
| DDX21 | DDX21 | Nucleolar RNA helicase 2 | [2] |
| DDX31 | DDX31 | DEAD-Box Helicase 31 | [4] |
| DDX47 | DDX47 | Probable ATP-dependent RNA helicase DDX47 | [2] |
| DDX50 | DDX50 | ATP-dependent RNA helicase DDX50 | [2] |
| DDX58 | RIG-I | DExD/H-Box Helicase 58 | [17] |
| DERA | DERA | Deoxyribose-Phosphate Aldolase | [18] |
| DGCR8 | DGCR8 | DGCR8 Microprocessor Complex Subunit | [4] |
| DHX30 | DHX30 | Putative ATP-dependent RNA helicase DHX30 | [1][2] |
| DHX33 | DHX33 | DEAH-Box Helicase 33 | [1] |
| DHX36 | RHAU | DEAH-Box Helicase 36 | [1][4][6] |
| DHX57 | DHX57 | DExH-Box Helicase 57 | [4][6] |
| DHX58 | LGP2 | DExH-Box Helicase 58 | [17] |
| DIDO1 | DIDO1 | Death Inducer-Obliterator 1 | [4] |
| DIS3L2 | DIS3L2/FAM3A | DIS3 Like 3'-5' Exoribonuclease 2 | [1] |
| DISC1 | Disrupted in Schizophrenia 1 | Disrupted In Schizophrenia 1 | [19] |
| DKC1 | DKC1 | dyskerin; H/ACA ribonucleoprotein complex subunit 4 | [2] |
| DNAI1 | Axonemal Dynein Intermediate Chain 1 | Dynein Axonemal Intermediate Chain 1 | [20] |
| DNAJA1 | DNAJA1 | DnaJ homolog subfamily A member 1 | [2] |
| DNAJC8 | DNAJC8 | DnaJ homolog subfamily C member 8 | [2] |
| DOCK4 | DOCK4 | Dedicator Of Cytokinesis 4 | [4] |
| DPYSL2 | DPYSL2 | Dihydropyrimidinase-related protein 2 | [2] |
| DPYSL3 | DPYSL3 | Dihydropyrimidinase-related protein 3 | [2] |
| DROSHA | DROSHA | Drosha Ribonuclease III | [1] |
| DSP | DSP | Desmoplakin | [1][2] |
| DST | DST | Dystonin | [2] |
| DSTN | DSTN | Destrin | [2] |
| DTL | DTL | Denticleless E3 Ubiquitin Protein Ligase Homolog | [4] |
| DTX3L | DTX3L | E3 ubiquitin-protein ligase DTX3L | [2] |
| DUSP12 | DUSP12/YVH1 | Dual Specificity Phosphatase 12 | [21] |
| DYNC1H1 | Cytoplasmic Dynein Heavy Chain 1 | Dynein Cytoplasmic 1 Heavy Chain 1 | [20] |
| DYNLL1 | Cytoplasmic Dynein Light Polypeptide | Dynein Light Chain LC8-Type 1 | [1] |
| DYNLL2 | DYNLL2 | Dynein light chain 2, cytoplasmic | [2] |
| DYRK3 | DYRK3 | Dual Specificity Tyrosine Phosphorylation Regulated Kinase 3 | [22] |
| DZIP1 | DZIP1 | DAZ Interacting Zinc Finger Protein 1 | [23] |
| DZIP3 | DZIP3 | DAZ Interacting Zinc Finger Protein 3 | [6] |
| EDC3 | EDC3 | Enhancer of mRNA Decapping 3 | [1][4][6] |
| EDC4 | EDC4 | Enhancer of mRNA-Decapping protein 4 | [1][2][4] |
| EIF1 | EIF1 | Eukaryotic Translation Initiation Factor 1 | [1] |
| EIF2A | EIF2A | Eukaryotic Translation Initiation Factor 2A | [2][5][10] |
| EIF2AK2 | Protein Kinase R/PKR | Eukaryotic Translation Initiation Factor 2 Alpha Kinase 2 | [17] |
| EIF2B1-5 | EIF2B | Eukaryotic Translation Initiation Factor 2B | [24] |
| EIF2S1 | EIF2A subunit 1 | Eukaryotic Translation Initiation Factor 2 Subunit Alpha | [2] |
| EIF2S2 | EIF2A subunit 2 | Eukaryotic Translation Initiation Factor 2 Subunit Beta | [2] |
| EIF3A | EIF3A | Eukaryotic Translation Initiation Factor 3 Subunit A | [1][2][4] |
| EIF3B | EIF3B | Eukaryotic Translation Initiation Factor 3 Subunit B | [2][10][15] |
| EIF3C | EIF3C | Eukaryotic Translation Initiation Factor 3 Subunit C | [1] |
| EIF3D | EIF3D | Eukaryotic translation initiation factor 3 subunit D | [1][2] |
| EIF3E | EIF3E | Eukaryotic translation initiation factor 3 subunit E | [1][2] |
| EIF3F | EIF3F | Eukaryotic translation initiation factor 3 subunit F | [2] |
| EIF3G | EIF3G | Eukaryotic translation initiation factor 3 subunit G | [1][2][4] |
| EIF3H | EIF3H | Eukaryotic translation initiation factor 3 subunit H | [1][2][4] |
| EIF3I | EIF3I | Eukaryotic translation initiation factor 3 subunit I | [2][4] |
| EIF3J | EIF3J | Eukaryotic translation initiation factor 3 subunit J | [1][2] |
| EIF3K | EIF3K | Eukaryotic translation initiation factor 3 subunit K | [2] |
| EIF3L | EIF3L | Eukaryotic translation initiation factor 3 subunit L | [1][2] |
| EIF3M | EIF3M | Eukaryotic translation initiation factor 3 subunit M | [2] |
| EIF4A1 | EIF4A1 | Eukaryotic Translation Initiation Factor 4A1 | [1][2][4] |
| EIF4A2 | EIF4A2 | Eukaryotic Translation Initiation Factor 4A2 | [1][4] |
| EIF4A3 | EIF4A3 | Eukaryotic Translation Initiation Factor 4A3 | [1] |
| EIF4B | EIF4B | Eukaryotic translation Initiation factor 4B | [1][2][4] |
| EIF4E | EIF4E | Eukaryotic Translation Initiation Factor 4E | [2][10] |
| EIF4E2 | EIF4E2 | Eukaryotic Translation Initiation Factor 4E Family Member 2 | [6] |
| EIF4E3 | EIF4E3 | Eukaryotic Translation Initiation Factor 4E Family Member 3 | [25] |
| EIF4ENIF1 | EIF4ENIF1 | Eukaryotic Translation Initiation Factor 4E Nuclear Import Factor 1 | [1][6] |
| EIF4G1 | EIF4G1 | Eukaryotic Translation Initiation Factor 4G1 | [1][2][4][15][24] |
| EIF4G2 | EIF4G2 | Eukaryotic Translation Initiation Factor 4G2 | [2][6] |
| EIF4G3 | EIF4G3 | Eukaryotic Translation Initiation Factor 4G3 | [1] |
| EIF4H | EIF4H | Eukaryotic translation Initiation factor 4H | [1][2][4] |
| EIF5A | EIF5A | Eukaryotic Translation Initiation Factor 5A | [26] |
| ELAVL1 | HuR | ELAV Like RNA Binding Protein 1 | [1][2][4][15] |
| ELAVL2 | ELAVL2 | ELAV-like protein 2 | [1][2] |
| ELAVL3 | ELAVL3/HuC | ELAV Like RNA Binding Protein 3 | [1] |
| ELAVL4 | HuD | ELAV Like RNA Binding Protein 4 | [1] |
| ENC1 | ENC1 | Ectodermal-Neural Cortex 1 | [4] |
| ENDOV | EndoV | Endonuclease V | [27] |
| ENTPD1 | ENTPD1 | Ectonucleoside Triphosphate Diphosphohydrolase 1 | [1] |
| EP400 | EP400 | E1A Binding Protein P400 | [4] |
| EPPK1 | EPPK1 | Epiplakin | [2] |
| ETF1 | ETF1 | Eukaryotic peptide chain release factor subunit 1 | [2] |
| EWSR1 | EWSR1 | EWS RNA Binding Protein 1 | [4] |
| FABP5 | FABP5 | Fatty Acid Binding Protein 5 | [1] |
| FAM83H | FAM83H | Family With Sequence Similarity 83 Member H | [4] |
| FAM98A | FAM98A | Family With Sequence Similarity 98 Member A | [1][2][4] |
| FAM98C | FAM98C | Family With Sequence Similarity 98 Member C | [4] |
| FAM120A | FAM120A/OSSA | Constitutive coactivator of PPAR-gamma-like protein 1 | [1][2][6] |
| FAM120C | FAM120C | Family With Sequence Similarity 120C | [1][6] |
| FAM168A | FAM168A | Family With Sequence Similarity 168 Member A | [4] |
| FAM168B | FAM168B/MANI | Family With Sequence Similarity 168 Member B | [1] |
| FASTK | FAST | Fas Activated Serine/Threonine Kinase | [10] |
| FBL | FBL | rRNA 2-O-methyltransferase fibrillarin | [2] |
| FBRSL1 | Fibrosin Like 1 | Fibrosin Like 1 | [6] |
| FHL1 | FHL1 | Four and a half LIM domains protein 1 | [2] |
| FKBP1A | FKBP1A | FKBP Prolyl Isomerase 1A | [4] |
| FLNB | FLNB | Filamin-B | [2] |
| FMR1 | FMRP | Fragile X Mental Retardation 1 | [1][2][4][6][20][21] |
| FNDC3B | FNDC3B | Fibronectin type III domain-containing protein 3B | [2][4][6] |
| FSCN1 | FSCN1 | Fascin | [2] |
| FTSJ3 | FTSJ3 | pre-rRNA processing protein FTSJ3 | [2] |
| FUBP1 | FUBP1 | Far Upstream Element Binding Protein 1 | [1][4] |
| FUBP3 | FUBP3 | Far upstream element-binding protein 3 | [1][2][4][6] |
| FUS | FUS | FUS RNA Binding Protein | [1][2][4] |
| FXR1 | FXR1 | FMR1 Autosomal Homolog 1 | [1][2][4][6] |
| FXR2 | FXR2 | FMR1 Autosomal Homolog 2 | [1][2][4][6] |
| G3BP1 | G3BP1 | G3BP Stress Granule Assembly Factor 1 | [1][2][4][6] |
| G3BP2 | G3BP2 | G3BP Stress Granule Assembly Factor 2 | [1][2][4][6] |
| GABARAPL2 | GABARAPL2/GEF2/ATG8 | GABA Type A Receptor Associated Protein Like 2 | [1] |
| GAK | GAK | Cyclin G Associated Kinase | [4] |
| GAR1 | GAR1 | H/ACA Ribonucleoprotein Complex Subunit 1 | [28] |
| GCA | Grancalcin | Grancalcin | [1] |
| GEMIN5 | Gemin-5 | Gem Nuclear Organelle Associated Protein 5 | [29] |
| GFPT1 | GFPT1 | Glutamine—fructose-6-phosphate aminotransferase [isomerizing] 1 | [2] |
| GIGYF1 | GIGYF1/PERQ1 | GRB10 Interacting GYF Protein 1 | [1] |
| GIGYF2 | GIGYF2/TNRC15/PARK11/PERQ2 | GRB10 Interacting GYF Protein 2 | [1][6] |
| GLE1 | GLE1 | GLE1, RNA Export Mediator | [6] |
| GLO1 | Glyoxalase | Glyoxalase | [1] |
| GLRX3 | GLRX3/Glutaredoxin 3/TNLX2 | Glutaredoxin 3 | [1] |
| GLUD1 | GLUD1 | Glutamate Dehydrogenase 1 | [4] |
| GNB2 | GNB2 | Guanine nucleotide-binding protein G(I)/G(S)/G(T) subunit beta-2 | [2] |
| GOLGA2 | Golgin A2 | Golgin A2 | [1] |
| GPAT3 | GPAT3 | Glycerol-3-Phosphate Acyltransferase 3 | [4] |
| GRB2 | GRB2/ASH | Growth Factor Receptor Bound Protein 2 | [1] |
| GRB7 | GRB7 | Growth Factor Receptor Bound Protein 7 | [30] |
| GRSF1 | GRSF1 | G-Rich RNA Sequence Binding Factor 1 | [1][6] |
| GSPT1 | eRF3 | G1 To S Phase Transition 1 | [1] |
| GTF2I | GTF2I | General Transcription Factor IIi | [4] |
| GTF3C1 | GTF3C1 | General Transcription Factor IIIC Subunit 1 | [4] |
| GTF3C4 | GTF3C4 | General Transcription Factor IIIC Subunit 4 | [4] |
| H1F0 | H1F0 | Histone H1.0 | [2] |
| H1FX | H1FX | Histone H1x | [2] |
| H2AFV | H2AFV | Histone H2A.V | [2] |
| HABP4 | Ki-1/57 | Hyaluronan Binding Protein 4 | [31] |
| HDAC6 | HDAC6 | Histone Deacetylase 6 | [32] |
| HDLBP | HDL-Binding Protein/VGL/Vigilin | High Density Lipoprotein Binding Protein | [1] |
| HELZ | HELZ | Probable helicase with zinc finger domain | [1][2][6] |
| HELZ2 | HELZ2 | Helicase with zinc finger domain 2 | [2] |
| HMGA1 | HMGA1 | High mobility group protein HMG-I/HMG-Y | [2] |
| HMGB3 | HMGB3 | High mobility group protein B3 | [2] |
| HMGN1 | HMGN1 | Non-histone chromosomal protein HMG-14 | [2] |
| HNRNPA1 | HnRNPA1 | Heterogeneous Nuclear Ribonucleoprotein A1 | [1][2] |
| HNRNPA2B1 | HnRNPA2/B1 | Heterogeneous Nuclear Ribonucleoprotein A2/B1 | [1][2] |
| HNRNPA3 | HNRNPA3 | Heterogeneous nuclear ribonucleoprotein A3 | [1][2] |
| HNRNPAB | HNRNPAB | Heterogeneous nuclear ribonucleoprotein A/B | [1][2][6] |
| HNRNPD | HNRNPD | Heterogeneous nuclear ribonucleoprotein D | [1] |
| HNRNPDL | HNRNPDL | Heterogeneous nuclear ribonucleoprotein D-like | [1] |
| HNRNPF | HNRNPF | Heterogeneous nuclear ribonucleoprotein F | [1] |
| HNRNPH1 | HNRNPH1 | Heterogeneous nuclear ribonucleoprotein H1 | [1] |
| HNRNPH2 | HNRNPH2 | Heterogeneous nuclear ribonucleoprotein H2 | [2] |
| HNRNPH3 | HNRNPH3 | Heterogeneous nuclear ribonucleoprotein H3 | [1] |
| HNRNPK | HNRNPK | Heterogeneous Nuclear Ribonucleoprotein K | [2] |
| HNRNPUL1 | HNRNPUL1 | Heterogeneous nuclear ribonucleoprotein U-like protein 2 | [2] |
| HSBP1 | HSBP1 | Heat Shock Factor Binding Protein 1 | [1] |
| HSP90AA1 | HSP90 | Heat shock protein HSP 90-alpha | [2] |
| HSPA4 | HSP70 RY | Heat shock 70 kDa protein 4 | [2] |
| HSPA9 | HSP70 9B | Stress-70 protein, mitochondrial | [2] |
| HSPB1 | HSP27 | Heat Shock Protein Family B (Small) Member 1 | [2] |
| HSPB8 | HSPB8 | Heat Shock Protein Family B (Small) Member 8 | [33] |
| HSPBP1 | HSPBP1 | HSPA (Hsp70) Binding Protein 1 | [34] |
| HSPD1 | HSPD1 | 60 kDa heat shock protein, mitochondrial | [1][2] |
| HTT | Huntingtin | Huntingtin | [35] |
| IBTK | IBTK | Inhibitor Of Bruton Tyrosine Kinase | [6] |
| IFIH1 | MDA5 | Interferon Induced With Helicase C Domain 1 | [17] |
| IGF2BP1 | IGF2BP1 | Insulin-like Growth Factor 2 mRNA-binding protein 1 | [1][2][6] |
| IGF2BP2 | IGF2BP2 | Insulin-like Growth Factor 2 mRNA-binding protein 2 | [1][2][6] |
| IGF2BP3 | IGF2BP3 | Insulin-like Growth Factor 2 mRNA Binding Protein 3 | [1][2][6] |
| IK | IK | Protein Red | [2] |
| ILF3 | NF90 | Interleukin Enhancer Binding Factor 3 | [36] |
| IPO7 | IPO7 | Importin-7 | [2] |
| IPPK | IP5K | Inositol-Pentakisphosphate 2-Kinase | [37] |
| ITGB1 | ITGB1 | Integrin beta-1 | [2] |
| JMJD6 | JMJD6 | Arginine Demethylase and Lysine Hydroxylase | [38] |
| KANK2 | KANK2 | KN motif and ankyrin repeat domain-containing protein 2 | [2] |
| KEAP1 | KEAP1/KLHL19 | Kelch Like ECH Associated Protein 1 | [1] |
| KHDRBS1 | Sam68 | KH RNA Binding Domain Containing, Signal Transduction Associated 1 | [2] |
| KHDRBS3 | KHDRBS3 | KH domain-containing, RNA-binding, signal transduction-associated protein 3 | [2] |
| KHSRP | KSRP/FBP2 | KH-Type Splicing Regulatory Protein | [1][2] |
| KIAA0232 | KIAA0232 | KIAA0232 | [6] |
| KIAA1524 | CIP2A | Protein CIP2A | [2] |
| KIF1B | KIF1B | Kinesin Family Member 1B | [6] |
| KIF2A | Kinesin Heavy Chain Member 2 | Kinesin Family Member 2A | [20] |
| KIF13B | KIF13B/GAKIN | Kinesin Family Member 13B | [1] |
| KIF23 | KIF23 | Kinesin-like protein KIF23 | [2] |
| KLC1 | Kinesin Light Chain 1 | Kinesin Light Chain 1 | [20] |
| KPNA1 | Importin-ɑ5 | Karyopherin Subunit Alpha 1 | [1][2] |
| KPNA2 | Importin-ɑ1 | Karyopherin Subunit Alpha 2 | [1][2] |
| KPNA3 | Importin-ɑ4 | Karyopherin Subunit Alpha 3 | [1] |
| KPNA6 | Importin-ɑ7 | Importin subunit alpha | [2] |
| KPNB1 | Importin-β1 | Karyopherin Subunit Beta 1 | [2] |
| L1RE1 | LINE1 ORF1p | LINE1 ORF1 protein | [2] |
| LANCL1 | LanC Like 1 | LanC Like 1 | [1] |
| LARP1 | LARP1 | La-related protein 1 | [2] |
| LARP1B | LARP1B | La-related protein 1b | [6] |
| LARP4 | La-Related protein 4 | La Ribonucleoprotein Domain Family Member 4 | [2][1][6] |
| LARP4B | LARP4B | La Ribonucleoprotein Domain Family Member 4B | [1][6] |
| LASP1 | LIM And SH3 Protein 1/MLN50 | LIM And SH3 Protein 1 | [1] |
| LBR | LBR | Lamin-B receptor | [2] |
| LEMD3 | LEMD3 | Inner nuclear membrane protein Man1 | [2] |
| LIG3 | DNA Ligase 3 | DNA Ligase 3 | [1] |
| LIN28A | LIN28A | Lin-28 Homolog A | [1] |
| LIN28B | LIN28B | Lin-28 Homolog B | [1] |
| LMNA | LMNA | Prelamin-A/C | [2] |
| LPP | LPP | Lipoma-preferred partner | [2] |
| LSM1 | LSM1 | LSM1 Homolog, mRNA Degradation Associated | [1] |
| LSM3 | LSM3 | U6 snRNA-associated Sm-like protein LSm3 | [2] |
| LSM12 | LSM12 | LSM12 Homolog | [1][6] |
| LSM14A | RAP55 | LSM14A, mRNA Processing Body Assembly Factor | [1][2][6] |
| LSM14B | LSM14B | Protein LSM14 homolog B | [1][2][6] |
| LUC7L | LUC7L | Putative RNA-binding protein Luc7-like 1 | [2] |
| LUZP1 | LUZP1 | Leucine zipper protein 1 | [2][6] |
| MACF1 | MACF1 | Microtubule-actin cross-linking factor 1, isoforms 1/2/3/5 | [2] |
| MAEL | MAEL | Maelstrom Spermatogenic Transposon Silencer | [39] |
| MAGEA4 | MAGEA4 | Melanoma-associated antigen 4 | [2] |
| MAGED1 | MAGED1 | Melanoma-associated antigen D1 | [1][2][6] |
| MAGED2 | MAGED2 | Melanoma-associated antigen D2 | [2] |
| MAGOHB | MAGOHB | Protein mago nashi homolog 2 | [2] |
| MAP1LC3A | LC3-I | Microtubule Associated Protein 1 Light Chain 3 Alpha | [40] |
| MAP4 | MAP4 | Microtubule-associated protein 4 | [2] |
| MAP4K4 | MAP4K4 | Mitogen-activated protein kinase kinase kinase kinase 4 | [2] |
| MAPK1IP1L | MAPK1IP1L | Mitogen-Activated Protein Kinase 1 Interacting Protein 1 Like | [1] |
| MAPK8 | JNK1 | Mitogen-Activated Protein Kinase 8 | [41] |
| MAPRE1 | MAPRE1 | Microtubule-associated protein RP/EB family member 1 | [2] |
| MAPRE2 | MAPRE2 | Microtubule Associated Protein RP/EB Family Member 2 | [1] |
| MARF1 | MARF1 | Meiosis Regulator And mRNA Stability Factor 1 | [6] |
| MARS | MARS | Methionine—tRNA ligase, cytoplasmic | [2] |
| MBNL1 | MBNL1 | Muscleblind Like Splicing Regulator 1 | [42] |
| MBNL2 | MBNL2 | Muscleblind Like Splicing Regulator 2 | [6] |
| MCM4 | MCM4 | DNA replication licensing factor MCM4 | [2] |
| MCM5 | MCM5 | DNA replication licensing factor MCM5 | [2] |
| MCM7 | MCM7 | DNA replication licensing factor MCM7 | [2] |
| MCRIP1 | FAM195B/GRAN2 | Granulin-2 | [1][6] |
| MCRIP2 | FAM195A/GRAN1 | Granulin-1 | [6] |
| METAP1 | METAP1 | Methionine aminopeptidase | [2] |
| METAP2 | METAP2 | Methionyl Aminopeptidase 2 | [1] |
| MEX3A | MEX3A | RNA-binding protein MEX3A | [2] |
| MEX3B | MEX3B | Mex-3 RNA Binding Family Member B | [1] |
| MEX3C | MEX3C | Mex-3 RNA Binding Family Member C | [1] |
| MEX3D | MEX3D | Mex-3 RNA Binding Family Member D | [6] |
| MFAP1 | MFAP1 | Microfibrillar-associated protein 1 | [2] |
| MKI67 | MKI67 | Antigen KI-67 | [2] |
| MKRN2 | MKRN2 | Makorin Ring Finger Protein 2 | [1][6] |
| MOV10 | MOV-10 | Mov10 RISC Complex RNA Helicase | [2][6][8] |
| MSH6 | MSH6 | DNA mismatch repair protein Msh6 | [2] |
| MSI1 | Musashi-1 | Musashi RNA Binding Protein 1 | [1] |
| MSI2 | MSI2 | RNA-binding protein Musashi homolog 2 | [1][2] |
| MTHFD1 | MTHFD1 | C-1-tetrahydrofolate synthase, cytoplasmic | [2] |
| MTHFSD | MTHFSD | Methenyltetrahydrofolate Synthetase Domain Containing | [43] |
| MTOR | MTOR | Mechanistic Target Of Rapamycin | [22] |
| MYO6 | MYO6 | Unconventional myosin-VI | [2] |
| NCOA3 | SRC-3 | Nuclear Receptor Coactivator 3 | [44] |
| NDEL1 | NUDEL/MITAP1/EOPA | NudE Neurodevelopment Protein 1 Like 1 | [1] |
| NELFE | NELF-E/RD | Negative Elongation Factor Complex Member E | [1] |
| NEXN | NEXN | Nexilin | [2] |
| NKRF | NRF | NFK-B Repressing Factor | [1] |
| NOLC1 | Nucleolar And Coiled-Body Phosphoprotein 1/NOPP140 | Nucleolar And Coiled-Body Phosphoprotein 1 | [1] |
| NONO | NonO | Non-POU Domain Containing Octamer Binding | [2] |
| NOP58 | NOP58 | Nucleolar protein 58 | [2] |
| NOSIP | NOSIP | Nitric oxide synthase-interacting protein | [2] |
| NOVA2 | NOVA2 | NOVA Alternative Splicing Regulator 2 | [1] |
| NRG2 | Neuregulin-2 | Neuregulin-2 | [45] |
| NSUN2 | NSUN2 | tRNA (cytosine(34)-C(5))-methyltransferase | [2] |
| NTMT1 | NTMT1 | N-terminal Xaa-Pro-Lys N-methyltransferase 1 | [2] |
| NUDC | NUDC | Nuclear migration protein nudC | [2] |
| NUFIP1 | NUFIP | NUFIP1, FMR1 Interacting Protein 1 | [46] |
| NUFIP2 | NUFIP2 | Nuclear fragile X mental retardation-interacting protein 2 | [1][2][6] |
| NUP50 | NUP50 | Nucleoporin 50 | [47] |
| NUP58 | NUP58/NUPL1 | Nucleoporin 58 | [47] |
| NUP85 | NUP85 | Nucleoporin 85 | [47] |
| NUP88 | NUP88 | Nucleoporin 88 | [47] |
| NUP98 | NUP98/NUP96 | Nuclear pore complex protein Nup98-Nup96 | [2][47] |
| NUP153 | NUP153 | Nucleoporin 153 | [1] |
| NUP205 | NUP205 | Nuclear pore complex protein Nup205 | [2][47] |
| NUP210 | NUP210/GP210 | Nucleoporin 210 | [47] |
| NUP214 | NUP214 | Nucleoporin 214 | [47] |
| NUPL2 | NUPL2 | Nucleoporin Like 2 | [47] |
| NXF1 | NXF1/MEX67/TAP | Nuclear RNA Export Factor 1 | [6] |
| OAS1 | OAS | 2′–5′ oligoadenylate synthetase | [17] |
| OAS2 | OAS2 | 2'-5'-Oligoadenylate Synthetase 2 | [48] |
| OASL | OASL/OASL1 | 2'-5'-Oligoadenylate Synthetase Like | [49] |
| OGFOD1 | TPA1 | 2-Oxoglutarate And Iron Dependent Oxygenase Domain Containing 1 | [50] |
| OGG1 | OGG1 | 8-Oxoguanine DNA Glycosylase | [51] |
| OSBPL9 | Oxysterol Binding Protein Like 9 | Oxysterol Binding Protein Like 9 | [1] |
| OTUD4 | OTUD4/HIN1 | OTU Deubiquitinase 4 | [1][6] |
| P4HB | Prolyl 4-Hydroxylase Subunit Beta | Prolyl 4-Hydroxylase Subunit Beta | [1] |
| PABPC1 | PABP1 | Poly(A) Binding Protein Cytoplasmic 1 | [1][2][6][46] |
| PABPC4 | PABPC4 | Polyadenylate-binding protein 4 | [1][2][6] |
| PAK4 | PAK4 | Serine/threonine-protein kinase PAK 4 | [1][2] |
| PALLD | Palladin | Palladin | [2] |
| PARG | PARG/PARG99/PARG102 | Poly(ADP-Ribose) Glycohydrolase | [52] |
| PARK7 | PARK7/DJ-1 | Parkinsonism Associated Deglycase | [53] |
| PARN | PARN/DAN | Poly(A)-Specific Ribonuclease | [1] |
| PARP12 | PARP-12/ARTD12 | Poly(ADP-Ribose) Polymerase Family Member 12 | [6][52] |
| PARP14 | PARP-14 | Poly(ADP-Ribose) Polymerase Family Member 14 | [52] |
| PARP15 | PARP-15 | Poly(ADP-Ribose) Polymerase Family Member 15 | [52] |
| PATL1 | PATL1 | PAT1 Homolog 1, Processing Body mRNA Decay Factor | [1][6] |
| PAWR | PAWR | PRKC apoptosis WT1 regulator protein | [2] |
| PCBP1 | PCBP1/HNRNPE1 | Poly(RC) Binding Protein 1 | [1][6] |
| PCBP2 | PCBP2/HNRNPE2 | Poly(RC) Binding Protein 2 | [1][2][6] |
| PCNA | PCNA | Proliferating cell nuclear antigen | [2] |
| PDAP1 | PDAP1 | PDGFA Associated Protein 1 | [1] |
| PDCD4 | PDCD4 | Programmed Cell Death 4 | [54] |
| PDCD6IP | PDCD6IP | Programmed cell death 6-interacting protein | [2] |
| PDIA3 | PDIA3 | Protein Disulfide Isomerase Family A Member 3 | [1] |
| PDLIM1 | PDLIM1 | PDZ and LIM domain protein 1 | [2] |
| PDLIM4 | PDLIM4 | PDZ and LIM domain protein 4 | [2] |
| PDLIM5 | PDLIM5 | PDZ and LIM domain protein 5 | [2] |
| PDS5B | PDS5B | Sister chromatid cohesion protein PDS5 homolog B | [2] |
| PEF1 | PEF1 | Penta-EF-Hand Domain Containing 1 | [1] |
| PEG10 | PEG10 | Paternally Expressed 10 | [6] |
| PELO | PELO | Protein pelota homolog | [2] |
| PEPD | Peptidase D | Peptidase D | [1] |
| PEX11B | PEX11B | Peroxisomal Biogenesis Factor 11 Beta | [1] |
| PFDN4 | PFDN4 | Prefoldin subunit 4 | [2] |
| PFN1 | Profilin 1 | Profilin 1 | [2] |
| PFN2 | Profilin 2 | Profilin 2 | [2] |
| PGAM5 | PGAM5 | Serine/threonine-protein phosphatase PGAM5, mitochondrial | [2] |
| PGP | PGP/G3PP | Phosphoglycolate Phosphatase | [1] |
| PHB2 | Prohibitin 2 | Prohibitin 2 | [19] |
| PHLDB2 | PHLDB2 | Pleckstrin homology-like domain family B member 2 | [2] |
| PKP1 | Plakophilin 1 | Plakophilin 1 | [55] |
| PKP2 | Plakophilin 2 | Plakophilin 2 | [2] |
| PKP3 | Plakophilin 3 | Plakophilin 3 | [55] |
| PNPT1 | PNPase I | Polyribonucleotide Nucleotidyltransferase 1 | [1] |
| POLR2B | POLR2B | DNA-directed RNA polymerase | [2] |
| POM121 | POM121 | POM121 Transmembrane Nucleoporin | [47] |
| POP7 | RPP20 | POP7 Homolog, Ribonuclease P/MRP Subunit | [56] |
| PPME1 | PPME1 | Protein phosphatase methylesterase 1 | [2] |
| PPP1R8 | PPP1R8 | Protein Phosphatase 1 Regulatory Subunit 8 | [1] |
| PPP1R10 | PPP1R10 | Serine/threonine-protein phosphatase 1 regulatory subunit 10 | [2] |
| PPP1R18 | PPP1R18 | Phostensin | [2] |
| PPP2R1A | PPP2R1A | Serine/threonine-protein phosphatase 2A 65 kDa regulatory subunit A alpha isoform | [2] |
| PPP2R1B | PPP2R1B | Serine/threonine-protein phosphatase 2A 65 kDa regulatory subunit A beta isoform | [1] |
| PQBP1 | PQBP-1 | Polyglutamine Binding Protein 1 | [57] |
| PRDX1 | PRDX1 | Peroxiredoxin-1 | [1][2] |
| PRDX6 | PRDX6 | Peroxiredoxin-6 | [2] |
| PRKAA2 | AMPK-a2 | Protein Kinase AMP-Activated Catalytic Subunit Alpha 2 | [18] |
| PRKCA | PKC-ɑ | Protein Kinase C Alpha | [58] |
| PRKRA | PACT | Protein Activator Of Interferon Induced Protein Kinase EIF2AK2 | [2][3] |
| PRMT1 | PRMT1 | Protein arginine N-methyltransferase 1 | [2] |
| PRMT5 | PRMT5 | Protein arginine N-methyltransferase 5 | [2] |
| PRRC2A | PRRC2A | Proline Rich Coiled-Coil 2A | [1][2][6] |
| PRRC2B | PRRC2B | Proline Rich Coiled-Coil 2B | [1][6] |
| PRRC2C | PRRC2C | Proline Rich Coiled-Coil 2C | [1][2][6] |
| PSMD2 | PSMD2 | 26S proteasome non-ATPase regulatory subunit 2 | [2] |
| PSPC1 | PSP1 | Paraspeckle Component 1 | [1] |
| PTBP1 | PTBP1 | Polypyrimidine tract-binding protein 1 | [1] |
| PTBP3 | PTBP3 | Polypyrimidine tract-binding protein 3 | [1][2][6] |
| PTGES3 | PTGES3 | Prostaglandin E synthase 3 | [2] |
| PTK2 | FAK | Protein Tyrosine Kinase 2 | [30] |
| PUM1 | Pumilio-1 | Pumilio homolog 1 | [1][2][6] |
| PUM2 | Pumilio-2 | Pumilio RNA Binding Family Member 2 | [1][6] |
| PURA | PURA | Transcriptional activator protein Pur-alpha | [1][2] |
| PURB | PURB | Transcriptional activator protein Pur-beta | [1][2] |
| PWP1 | PWP1 | PWP1 Homolog, Endonuclein | [1] |
| PXDNL | PMR1 | Peroxidasin Like | [59] |
| PYCR1 | PYCR1 | Pyrroline-5-carboxylate reductase | [2] |
| QKI | QKI/HQK | QKI, KH Domain Containing RNA Binding | [1] |
| R3HDM1 | R3HDM1 | R3H Domain Containing 1 | [1][6] |
| R3HDM2 | R3HDM2 | R3H Domain Containing 2 | [6] |
| RAB1A | RAB1A | Ras-related protein Rab-1A | [2][57] |
| RACGAP1 | RACGAP1 | Rac GTPase-activating protein 1 | [2] |
| RACK1 | RACK1 | Receptor For Activated C Kinase 1 | [19] |
| RAD21 | RAD21 | Double-strand-break repair protein rad21 homolog | [2] |
| RAE1 | RAE1 | Ribonucleic Acid Export 1 | [47] |
| RAN | RAN | RAN, Member RAS Oncogene Family | [47] |
| RANBP1 | RANBP1 | Ran-specific GTPase-activating protein | [2] |
| RANBP2 | RANBP2/NUP358 | RAN Binding Protein 2 | [47] |
| RBBP4 | RBBP4 | Histone-binding protein RBBP4 | [2] |
| RBFOX1 | RBFOX1 | RNA binding protein fox-1 homolog | [2] |
| RBFOX2 | RBFOX2 | RNA binding protein fox-1 homolog 2 | [60] |
| RBFOX3 | RBFOX3 | RNA binding protein fox-1 homolog 3 | [60] |
| RBM3 | RBM3 | RNA-binding protein 3 | [1] |
| RBM4 | RBM4 | RNA Binding Motif Protein 4 | [1] |
| RBM4B | RBM4B | RNA Binding Motif Protein 4B | [1] |
| RBM12B | RBM12B | RNA-binding protein 12B | [2] |
| RBM15 | RBM15 | RNA-binding protein 15 | [1] |
| RBM17 | RBM17 | RNA-binding protein 17 | [1] |
| RBM25 | RBM25 | RNA-binding protein 25 | [1] |
| RBM26 | RBM26 | RNA-binding protein 26 | [2] |
| RBM38 | RBM38 | RNA-binding protein 38 | [1] |
| RBM42 | RBM42 | RNA Binding Motif Protein 42 | [61] |
| RBM45 | RBM45 | RNA Binding Motif Protein 45 | [62] |
| RBM47 | RBM47 | RNA Binding Motif Protein 47 | [6] |
| RBMS1 | RBMS1 | RNA-binding motif, single-stranded-interacting protein 1 | [1][2][6] |
| RBMS2 | RBMS2 | RNA-binding motif, single-stranded-interacting protein 2 | [1][2][6] |
| RBMX | RBMX | RNA Binding Motif Protein, X-Linked | [6] |
| RBPMS | RBPMS | RNA-binding protein with multiple splicing | [63] |
| RC3H1 | Roquin-1 | Ring Finger And CCCH-Type Domains 1 | [1][6] |
| RC3H2 | MNAB | Ring Finger And CCCH-Type Domains 2 | [6] |
| RCC1 | RCC1 | Regulator of chromosome condensation | [2] |
| RCC2 | RCC2 | Protein RCC2 | [2] |
| RECQL | RECQL1 | RecQ Like Helicase | [1] |
| RFC3 | RFC3 | Replication factor C subunit 3 | [2] |
| RFC4 | RFC4 | Replication factor C subunit 4 | [2] |
| RGPD3 | RGPD3 | RanBP2-like and GRIP domain-containing protein 3 | [2] |
| RHOA | RhoA | Ras Homolog Family Member A | [20] |
| RNASEL | RNAse L | Ribonuclease L | [17] |
| RNF25 | RNF25 | Ring Finger Protein 25 | [1] |
| RNF214 | RNF214 | RING finger protein 214 | [1][2] |
| RNF219 | RNF219 | RING finger protein 219 | [6] |
| RNH1 | RNH1 | Ribonuclease inhibitor | [2][7] |
| ROCK1 | ROCK1 | Rho Associated Coiled-Coil Containing Protein Kinase 1 | [20] |
| RPS3 | 40S Ribosomal Protein S3 | 40S Ribosomal Protein S3 | [24] |
| RPS6 | Ribosomal Protein S6 | Ribosomal Protein S6 | [2][24][46] |
| RPS6KA3 | RSK2 | Ribosomal Protein S6 Kinase A3 | [64] |
| RPS6KB1 | S6K1 | Ribosomal Protein S6 Kinase B1 | [65] |
| RPS6KB2 | S6K2 | Ribosomal Protein S6 Kinase B2 | [65] |
| RPS11 | Ribosomal Protein S11 | Ribosomal Protein S11 | [1] |
| RPS19 | Ribosomal Protein S19 | Ribosomal Protein S19 | [66] |
| RPS24 | Ribosomal Protein S24 | Ribosomal Protein S24 | [1] |
| RPTOR | RAPTOR | Regulatory Associated Protein of mTOR Complex 1 | [22][65] |
| RSL1D1 | RSL1D1 | Ribosomal L1 domain-containing protein 1 | [2] |
| RTCB | RTCB | tRNA-splicing ligase RtcB homolog, formerly C22orf28 | [1][2] |
| RTRAF | RTRAF (formerly C14orf166) | RNA Transcription, Translation And Transport Factor | [1] |
| S100A7A | S100A7A | Protein S100-A7A | [2] |
| S100A9 | S100A9 | Protein S100-A9 | [2] |
| SAFB2 | SAFB2 | Scaffold attachment factor B2 | [1][2] |
| SAMD4A | SMAUG1 | Sterile Alpha Motif Domain Containing 4A | [67] |
| SAMD4B | SMAUG2 | Sterile Alpha Motif Domain Containing 4B | [1] |
| SCAPER | SCAPER | S-Phase Cyclin A Associated Protein In The ER | [6] |
| SEC24C | SEC24C | Protein transport protein Sec24C | [1][2] |
| SECISBP2 | SECIS Binding Protein 2 | SECIS Binding Protein 2 | [1][6] |
| SERBP1 | PAI-RBP1/SERBP1 | SERPINE1 mRNA Binding Protein 1 | [46] |
| SERPINE1 | PAI-1/Serpin E1 | Serpine Family E Member 1 | [68] |
| SF1 | SF1 | Splicing Factor 1 | [1] |
| SFN | SFN | 14-3-3 protein sigma | [2] |
| SFPQ | PSF | Splicing Factor Proline And Glutamine Rich | [2] |
| SFRS3 | SFRS3 | Serine/arginine-rich splicing factor 3 | [2] |
| SIPA1L1 | SIPA1L1 | Signal-induced proliferation-associated 1-like protein 1 | [2] |
| SIRT6 | Sirtuin 6 | Sirtuin 6 | [69] |
| SLBP | Stem-Loop Binding Protein | Stem-Loop Binding Protein | [1] |
| SMAP2 | SMAP2 | Small ArfGAP2 | [6] |
| SMARCA1 | SMARCA1/SNF2L1 | Probable global transcription activator SNF2L1 | [2] |
| SMC4 | SMC4 | Structural maintenance of chromosomes protein | [2] |
| SMG1 | SMG-1 | SMG1, Nonsense Mediated mRNA Decay Associated PI3K Related Kinase | [67] |
| SMG6 | SMG6 | SMG6, Nonsense Mediated mRNA Decay Factor | [6] |
| SMG7 | SMG7 | SMG7, Nonsense Mediated mRNA Decay Factor | [6] |
| SMN1 | Survival of Motor Neuron | Survival Of Motor Neuron 1, Telomeric | [56] |
| SMU1 | SMU1 | WD40 repeat-containing protein SMU1 | [2] |
| SMYD5 | SMYD5 | SMYD Family Member 5 | [1] |
| SND1 | Tudor-SN | Staphylococcal Nuclease And Tudor Domain Containing 1 | [1][6][44] |
| SNRPF | SNRPF | Small nuclear ribonucleoprotein F | [2] |
| SNTB2 | SNTB2 | Beta-2-syntrophin | [2] |
| SOGA3 | SOGA3 | SOGA Family Member 3 | [1] |
| SORBS1 | SORBS1 | Sorbin and SH3 domain-containing protein 1 | [2] |
| SORBS3 | Vinexin | Sorbin And SH3 Domain Containing 3 | [70] |
| SOX3 | SOX3 | SRY-Box 3 | [1] |
| SPAG5 | Astrin | Sperm Associated Antigen 5 | [65] |
| SPATS2 | SPATS2/SPATA10/SCR59 | Spermatogenesis Associated Serine Rich 2 | [1] |
| SPATS2L | SGNP | Spermatogenesis Associated Serine Rich 2 Like | [2] |
| SPECC1L | SPECC1L | Cytospin-A | [2] |
| SQSTM1 | SQSTM1/p62 | Sequestosome 1 | [11] |
| SRI | SRI | Sorcin | [1][2] |
| SRP9 | SRP9 | Signal Recognition Particle 9 | [71] |
| SRP68 | Signal Recognition Particle 68 | Signal Recognition Particle 68 | [1][8] |
| SRRT | SRRT | Serrate RNA effector molecule homolog | [2] |
| SRSF1 | ASF/SF2 | Serine And Arginine Rich Splicing Factor 1 | [1] |
| SRSF3 | SRp20 | Serine And Arginine Rich Splicing Factor 3 | [57] |
| SRSF4 | SRSF4 | Serine/arginine-rich splicing factor 4 | [2] |
| SRSF5 | SRSF5/SRP40 | Serine/arginine-rich splicing factor 5 | [1] |
| SRSF7 | 9G8 | Serine And Arginine Rich Splicing Factor 7 | [46] |
| SRSF9 | SRSF9/SRP30C | Serine/arginine-rich splicing factor 9 | [1] |
| SS18L1 | SS18L1/CREST | SS18L1, nBAF Chromatin Remodeling Complex Subunit | [72] |
| ST7 | ST7/FAM4A1/HELG/RAY1/TSG7 | Suppression Of Tumorigenicity 7 | [6] |
| STAT1 | STAT1 | Signal transducer and activator of transcription 1-alpha/beta | [2] |
| STAU1 | Staufen 1 | Staufen Double-Stranded RNA Binding Protein 1 | [2][1][67] |
| STAU2 | Staufen 2 | Staufen Double-Stranded RNA Binding Protein 2 | [1][2][6] |
| STIP1 | STIP1/HOP | Stress-induced-phosphoprotein 1 | [2][3] |
| STRAP | STRAP | Serine-threonine kinase receptor-associated protein | [1][2] |
| SUGP2 | SUGP2 | SURP and G-patch domain-containing protein 2 | [2] |
| SUGT1 | SUGT1 | SGT1 Homolog, MIS12 Kinetochore Complex Assembly Cochaperone | [6] |
| SUN1 | SUN1 | SUN domain-containing protein 1 | [2] |
| SYCP3 | SYCP3 | Synaptonemal complex protein 3 | [2] |
| SYK | SYK | Spleen Associated Tyrosine Kinase | [73] |
| SYNCRIP | SYNCRIP | Heterogeneous nuclear ribonucleoprotein Q | [1][2][6] |
| TAF15 | TAF15 | TATA-Box Binding Protein Associated Factor 15 | [1][2][57] |
| TAGLN3 | Transgelin 3 | Transgelin 3 | [1] |
| TARDBP | TDP-43 | TAR DNA Binding Protein | [2] |
| TBRG1 | TBRG1 | Transforming Growth Factor Beta Regulator 1 | [1] |
| TCEA1 | TCEA1 | Transcription elongation factor A protein 1 | [2] |
| TCP1 | TCP1 | T-complex protein 1 subunit alpha | [2] |
| TDRD3 | Tudor Domain Containing 3 | Tudor Domain Containing 3 | [1][6] |
| TDRD7 | Tudor Domain Containing 7 | Tudor Domain Containing 7 | [6] |
| TERT | TERT | Telomerase Reverse Transcriptase | [74] |
| THOC2 | THOC2 | THO Complex 2 | [47] |
| THRAP3 | THRAP3 | Thyroid Hormone Receptor Associated Protein 3 | [1] |
| TIA1 | TIA-1 | TIA1 Cytotoxic Granule Associated RNA Binding Protein | [1][2][15][31][32][46][53][57][67] |
| TIAL1 | TIAR | TIA1 Cytotoxic Granule Associated RNA Binding Protein Like 1 | [1][2][6][46][62][67][72] |
| TMEM131 | TMEM131 | Transmembrane Protein 131 | [6] |
| TMOD3 | TMOD3 | Tropomodulin-3 | [2] |
| TNKS | PARP-5a | Tankyrase | [52] |
| TNKS1BP1 | TNKS1BP1 | 182 kDa tankyrase-1-binding protein | [2][6] |
| TNPO1 | Transportin-1 | Transportin-1/Karyopherin (Importin) Beta 2 | [1][2][47] |
| TNPO2 | Transportin-2 | Transportin-2 | [2][6] |
| TNRC6A | TNRC6A | Trinucleotide repeat-containing gene 6A protein | [1][6] |
| TNRC6B | TNRC6B | Trinucleotide repeat-containing gene 6B protein | [1][2][6] |
| TNRC6C | TNRC6C | Trinucleotide repeat-containing gene 6C protein | [1][6] |
| TOMM34 | TOMM34 | Mitochondrial import receptor subunit TOM34 | [2] |
| TOP3B | Topoisomerase (DNA) III Beta | Topoisomerase (DNA) III Beta | [6] |
| TPM1 | TPM1 | Tropomyosin alpha-1 chain | [2] |
| TPM2 | TPM2 | Tropomyosin beta chain | [2] |
| TPR | TPR | Translocated Promoter Region, Nuclear Basket Protein | [47] |
| TRA2B | TRA2B | Transformer 2 Beta Homolog | [6] |
| TRAF2 | TRAF2 | TNF Receptor Associated Factor 2 | [75] |
| TRDMT1 | DNMT2 | tRNA Aspartic Acid Methyltransferase 1 | [76] |
| TRIM21 | TRIM21 | E3 ubiquitin-protein ligase TRIM21 | [2] |
| TRIM25 | TRIM25 | E3 ubiquitin/ISG15 ligase TRIM25 | [1][2][57] |
| TRIM56 | TRIM56 | E3 ubiquitin-protein ligase TRIM56 | [2][6][57] |
| TRIM71 | TRIM71 | E3 ubiquitin-protein ligase TRIM71 | [1] |
| TRIP6 | TRIP6 | Thyroid receptor-interacting protein 6 | [1][2] |
| TROVE2 | RORNP | TROVE Domain Family Member 2 | [1] |
| TTC17 | TTC17 | Tetratricopeptide Repeat Domain 17 | [6] |
| TUBA1C | TUBA1C | Tubulin alpha-1C chain | [2] |
| TUBA3C | TUBA3C | Tubulin alpha-3C/D chain | [2] |
| TUBA4A | TUBA4A | Tubulin alpha-4A chain | [2] |
| TUBB3 | TUBB3 | Tubulin beta-3 chain | [2] |
| TUBB8 | TUBB8 | Tubulin beta-8 chain | [2] |
| TUFM | TUFM | Elongation factor Tu, mitochondrial | [2] |
| TXN | TXN | Thioredoxin | [2] |
| TXNDC17 | TXNDC17 | Thioredoxin Domain Containing 17 | [1] |
| U2AF1 | U2AF1 | Splicing factor U2AF 35 kDa subunit | [2] |
| UBA1 | UBA1 | Ubiquitin-like modifier-activating enzyme 1 | [2] |
| UBAP2 | UBAP2 | Ubiquitin-associated protein 2 | [1][2][6][57] |
| UBAP2L | UBAP2L | Ubiquitin-associated protein 2-like | [1][2][6][57] |
| UBB | Ubiquitin | Ubiquitin | [32] |
| UBL5 | Ubiquitin Like 5 | Ubiquitin Like 5 | [1] |
| UBQLN2 | Ubiquilin 2 | Ubiquilin 2 | [77] |
| ULK1 | ULK1 | Unc-51 Like Autophagy Activating Kinase 1 | [78] |
| ULK2 | ULK2 | Unc-51 Like Autophagy Activating Kinase 2 | [78] |
| UPF1 | UPF1 | UPF1, RNA Helicase and ATPase | [1][2][6][57] |
| UPF2 | UPF2 | UPF2, RNA Helicase and ATPase | [79] |
| UPF3B | UPF3B | UPF3B, Regulator of Nonsense Mediated mRNA Decay | [1] |
| USP5 | USP5 | Ubiquitin carboxyl-terminal hydrolase 5 | [2] |
| USP9X | USP9X | Ubiquitin Specific Peptidase 9, X-Linked | [80] |
| USP10 | USP10 | Ubiquitin Specific Peptidase 10 | [1][2][6][31][57][64] |
| USP11 | USP11 | Ubiquitin Specific Peptidase 11 | [1] |
| USP13 | USP13 | Ubiquitin Specific Peptidase 13 | [81] |
| UTP18 | UTP18 | UTP18, Small Subunit Processome Component | [1] |
| VASP | VASP | Vasodilator-stimulated phosphoprotein | [2] |
| VBP1 | VBP1 | VHL Binding Protein 1 | [1] |
| VCP | VCP | Valosin Containing Protein | [2][78] |
| WBP2 | WBP2 | WW Domain Binding Protein 2 | [1] |
| WDR47 | WDR47 | WD Repeat Domain 47 | [1] |
| WDR62 | WDR62 | WD Repeat Domain 62 | [41] |
| XPO1 | XPO1/CRM1 | Exportin 1 | [47] |
| XRN1 | XRN1 | 5'-3' Exoribonuclease 1 | [1][6][10] |
| XRN2 | XRN2 | 5'-3' Exoribonuclease 2 | [1] |
| YARS | YARS | Tyrosine—tRNA ligase, cytoplasmic | [2] |
| YBX1 | YB-1 | Y-Box Binding Protein 1 | [1][2][8][21][42][46] |
| YBX3 | YBX3/ZONAB | Y-box-binding protein 3 | [1][2][6] |
| YES1 | YES1 | Tyrosine-protein kinase Yes | [2] |
| YLPM1 | YLPM1 | YLP Motif Containing 1 | [1] |
| YTHDF1 | YTHDF1 | YTH domain family protein 1 | [1][2][6] |
| YTHDF2 | YTHDF2 | YTH domain family protein 2 | [1][2][6] |
| YTHDF3 | YTHDF3 | YTH domain family protein 3 | [1][2][6][29] |
| YWHAB | 14/03/2003 | Tyrosine 3-Monooxygenase/Tryptophan 5-Monooxygenase Activation Protein Beta | [2] |
| YWHAH | 14/03/2003 | 14-3-3 protein eta | [2] |
| YWHAQ | 14/03/2003 | 14-3-3 protein theta | [2] |
| ZBP1 | ZBP1 | Z-DNA Binding Protein 1 | [82] |
| ZC3H7A | ZC3H7A | Zinc finger CCCH domain-containing protein 7A | [2] |
| ZC3H7B | ZC3H7B | Zinc finger CCCH domain-containing protein 7B | [1][2] |
| ZC3H11A | ZC3H11A | Zinc finger CCCH domain-containing protein 11a | [1] |
| ZC3H14 | ZC3H14 | Zinc finger CCCH domain-containing protein 14 | [2] |
| ZC3HAV1 | PARP-13.1/PARP-13.2/ARTD13 | Zinc Finger CCCH-Type Containing, Antiviral 1 | [2][6][52] |
| ZCCHC2 | ZCCHC2 | Zinc finger CCCH domain-containing protein 2 | [6] |
| ZCCHC3 | ZCCHC3 | Zinc finger CCCH domain-containing protein 3 | [6] |
| ZCCHC11 | ZCCHC11 | Zinc finger CCCH domain-containing protein 11 | [6] |
| ZCCHC14 | ZCCHC14 | Zinc finger CCCH domain-containing protein 14 | [6] |
| ZFAND1 | ZFAND1 | Zinc Finger AN1-Type Containing 1 | [83] |
| ZFP36 | TTP/TIS11 | ZFP36 Ring Finger Protein/Trisetrapolin | [1][10][41] |
| ZNF598 | ZNF598 | Zinc finger protein 598 | [6] |
| ZNF638 | ZNF638 | Zinc finger protein 638 | [2] |

1. Markmiller, S. *et al.* Context-Dependent and Disease-Specific Diversity in Protein Interactions within Stress Granules. *Cell* **172**, 590-604.e13 (2018).

2. Pare, J. M. *et al.* Hsp90 Regulates the Function of Argonaute 2 and Its Recruitment to Stress Granules and P-Bodies. *Mol Biol Cell* **20**, 3273–3284 (2009).

3. Jain, S. *et al.* ATPase modulated stress granules contain a diverse proteome and substructure. *Cell* **164**, 487–498 (2016).

4. Marmor-Kollet, H. *et al.* Spatiotemporal Proteomic Analysis of Stress Granule Disassembly Using APEX Reveals Regulation by SUMOylation and Links to ALS Pathogenesis. *Mol Cell* **80**, 876-891.e6 (2020).

5. Kolobova, E. *et al.* Microtubule-dependent association of AKAP350A and CCAR1 with RNA stress granules. *Exp Cell Res* **315**, 542–555 (2009).

6. Youn, J.-Y. *et al.* High-Density Proximity Mapping Reveals the Subcellular Organization of mRNA-Associated Granules and Bodies. *Molecular Cell* **69**, 517-532.e11 (2018).

7. Pizzo, E. *et al.* Ribonuclease/angiogenin inhibitor 1 regulates stress-induced subcellular localization of angiogenin to control growth and survival. *J Cell Sci* **126**, 4308–4319 (2013).

8. Gallois-Montbrun, S. *et al.* Antiviral Protein APOBEC3G Localizes to Ribonucleoprotein Complexes Found in P Bodies and Stress Granules. *J Virol* **81**, 2165–2178 (2007).

9. Kim, B. & Rhee, K. BOULE, a Deleted in Azoospermia Homolog, Is Recruited to Stress Granules in the Mouse Male Germ Cells. *PLoS One* **11**, e0163015 (2016).

10. Kedersha, N. *et al.* Stress granules and processing bodies are dynamically linked sites of mRNP remodeling. *J Cell Biol* **169**, 871–884 (2005).

11. Chitiprolu, M. *et al.* A complex of C9ORF72 and p62 uses arginine methylation to eliminate stress granules by autophagy. *Nat Commun* **9**, 2794 (2018).

12. Maharjan, N., Künzli, C., Buthey, K. & Saxena, S. C9ORF72 Regulates Stress Granule Formation and Its Deficiency Impairs Stress Granule Assembly, Hypersensitizing Cells to Stress. *Mol Neurobiol* **54**, 3062–3077 (2017).

13. Fathinajafabadi, A., Pérez-Jiménez, E., Riera, M., Knecht, E. & Gonzàlez-Duarte, R. CERKL, a Retinal Disease Gene, Encodes an mRNA-Binding Protein That Localizes in Compact and Untranslated mRNPs Associated with Microtubules. *PLoS One* **9**, e87898 (2014).

14. Reineke, L. C. *et al.* Casein Kinase 2 Is Linked to Stress Granule Dynamics through Phosphorylation of the Stress Granule Nucleating Protein G3BP1. *Mol Cell Biol* **37**, e00596-16 (2017).

15. Kim, J.-E. *et al.* Proline-Rich Transcript in Brain Protein Induces Stress Granule Formation. *Mol Cell Biol* **28**, 803–813 (2008).

16. Kim, B., Cooke, H. J. & Rhee, K. DAZL is essential for stress granule formation implicated in germ cell survival upon heat stress. *Development* **139**, 568–578 (2012).

17. Onomoto, K. *et al.* Critical Role of an Antiviral Stress Granule Containing RIG-I and PKR in Viral Detection and Innate Immunity. *PLoS One* **7**, e43031 (2012).

18. Salleron, L. *et al.* DERA is the human deoxyribose phosphate aldolase and is involved in stress response. *Biochimica et Biophysica Acta (BBA) - Molecular Cell Research* **1843**, 2913–2925 (2014).

19. Ogawa, F., Kasai, M. & Akiyama, T. A functional link between Disrupted-In-Schizophrenia 1 and the eukaryotic translation initiation factor 3. *Biochemical and Biophysical Research Communications* **338**, 771–776 (2005).

20. Loschi, M., Leishman, C. C., Berardone, N. & Boccaccio, G. L. Dynein and kinesin regulate stress-granule and P-body dynamics. *J Cell Sci* **122**, 3973–3982 (2009).

21. Geng, Q., Xhabija, B., Knuckle, C., Bonham, C. A. & Vacratsis, P. O. The Atypical Dual Specificity Phosphatase hYVH1 Associates with Multiple Ribonucleoprotein Particles. *J Biol Chem* **292**, 539–550 (2017).

22. Wippich, F. *et al.* Dual Specificity Kinase DYRK3 Couples Stress Granule Condensation/Dissolution to mTORC1 Signaling. *Cell* **152**, 791–805 (2013).

23. ShigunovShigunov, P. *et al.* Ribonomic analysis of human DZIP1 reveals its involvement in ribonucleoprotein complexes and stress granules. *BMC Mol Biol* **15**, 12 (2014).

24. Kimball, S. R., Horetsky, R. L., Ron, D., Jefferson, L. S. & Harding, H. P. Mammalian stress granules represent sites of accumulation of stalled translation initiation complexes. *American Journal of Physiology-Cell Physiology* **284**, C273–C284 (2003).

25. Frydryskova, K. *et al.* Distinct recruitment of human eIF4E isoforms to processing bodies and stress granules. *BMC Mol Biol* **17**, 21 (2016).

26. Li, C. H., Ohn, T., Ivanov, P., Tisdale, S. & Anderson, P. eIF5A Promotes Translation Elongation, Polysome Disassembly and Stress Granule Assembly. *PLoS One* **5**, e9942 (2010).

27. Nawaz, M. S. *et al.* Regulation of Human Endonuclease V Activity and Relocalization to Cytoplasmic Stress Granules. *J Biol Chem* **291**, 21786–21801 (2016).

28. Belli, V. *et al.* A dynamic link between H/ACA snoRNP components and cytoplasmic stress granules. *Biochimica et Biophysica Acta (BBA) - Molecular Cell Research* **1866**, 118529 (2019).

29. Battle, D. J., Kasim, M., Wang, J. & Dreyfuss, G. SMN-independent Subunits of the SMN Complex: IDENTIFICATION OF A SMALL NUCLEAR RIBONUCLEOPROTEIN ASSEMBLY INTERMEDIATE *. *Journal of Biological Chemistry* **282**, 27953–27959 (2007).

30. Tsai, N.-P., Ho, P.-C. & Wei, L.-N. Regulation of stress granule dynamics by Grb7 and FAK signalling pathway. *EMBO J* **27**, 715–726 (2008).

31. de Almeida Gonçalves, K. *et al.* Evidence for the association of the human regulatory protein Ki-1/57 with the translational machinery. *FEBS Letters* **585**, 2556–2560 (2011).

32. Kwon, S., Zhang, Y. & Matthias, P. The deacetylase HDAC6 is a novel critical component of stress granules involved in the stress response. *Genes Dev* **21**, 3381–3394 (2007).

33. A Surveillance Function of the HSPB8-BAG3-HSP70 Chaperone Complex Ensures Stress Granule Integrity and Dynamism: Molecular Cell. https://www.cell.com/molecular-cell/fulltext/S1097-2765(16)30374-4?_returnURL=https%3A%2F%2Flinkinghub.elsevier.com%2Fretrieve%2Fpii%2FS1097276516303744%3Fshowall%3Dtrue.

34. Mahboubi, H., Moujaber, O., Kodiha, M. & Stochaj, U. The Co-Chaperone HspBP1 Is a Novel Component of Stress Granules that Regulates Their Formation. *Cells* **9**, 825 (2020).

35. Ratovitski, T. *et al.* Huntingtin protein interactions altered by polyglutamine expansion as determined by quantitative proteomic analysis. *Cell Cycle* **11**, 2006–2021 (2012).

36. Wen, X. *et al.* NF90 Exerts Antiviral Activity through Regulation of PKR Phosphorylation and Stress Granules in Infected Cells. *The Journal of Immunology* **192**, 3753–3764 (2014).

37. Brehm, M. A. *et al.* Intracellular localization of human Ins(1,3,4,5,6)P5 2-kinase. *Biochem J* **408**, 335–345 (2007).

38. Tsai, W.-C., Reineke, L. C., Jain, A., Jung, S. Y. & Lloyd, R. E. Histone arginine demethylase JMJD6 is linked to stress granule assembly through demethylation of the stress granule–nucleating protein G3BP1. *J Biol Chem* **292**, 18886–18896 (2017).

39. Proteomic analysis reveals that MAEL, a component of nuage, interacts with stress granule proteins in cancer cells. https://www.spandidos-publications.com/10.3892/or.2013.2836.

40. Ryu, H.-H. *et al.* Autophagy regulates amyotrophic lateral sclerosis-linked fused in sarcoma-positive stress granules in neurons. *Neurobiology of Aging* **35**, 2822–2831 (2014).

41. Wasserman, T. *et al.* A Novel c-Jun N-terminal Kinase (JNK)-binding Protein WDR62 Is Recruited to Stress Granules and Mediates a Nonclassical JNK Activation. *Mol Biol Cell* **21**, 117–130 (2010).

42. Onishi, H. *et al.* MBNL1 associates with YB-1 in cytoplasmic stress granules. *Journal of Neuroscience Research* **86**, 1994–2002 (2008).

43. MacNair, L. *et al.* MTHFSD and DDX58 are novel RNA-binding proteins abnormally regulated in amyotrophic lateral sclerosis. *Brain* **139**, 86–100 (2016).

44. Yu, C. *et al.* An Essential Function of the SRC-3 Coactivator in Suppression of Cytokine mRNA Translation and Inflammatory Response. *Mol Cell* **25**, 765–778 (2007).

45. Kim, J. A. *et al.* Identification of Neuregulin-2 as a novel stress granule component. *BMB Rep* **49**, 449–454 (2016).

46. Kim, S. H., Dong, W. K., Weiler, I. J. & Greenough, W. T. Fragile X Mental Retardation Protein Shifts between Polyribosomes and Stress Granules after Neuronal Injury by Arsenite Stress or In Vivo Hippocampal Electrode Insertion. *J Neurosci* **26**, 2413–2418 (2006).

47. Zhang, K. *et al.* Stress granule assembly disrupts nucleocytoplasmic transport. *Cell* **173**, 958-971.e17 (2018).

48. Reineke, L. C. & Lloyd, R. E. The Stress Granule Protein G3BP1 Recruits Protein Kinase R To Promote Multiple Innate Immune Antiviral Responses. *J Virol* **89**, 2575–2589 (2014).

49. Kang, J.-S. *et al.* OASL1 Traps Viral RNAs in Stress Granules to Promote Antiviral Responses. *Mol Cells* **41**, 214–223 (2018).

50. Wehner, K. A., Schütz, S. & Sarnow, P. OGFOD1, a Novel Modulator of Eukaryotic Translation Initiation Factor 2α Phosphorylation and the Cellular Response to Stress. *Mol Cell Biol* **30**, 2006–2016 (2010).

51. Bravard, A. *et al.* Inactivation by oxidation and recruitment into stress granules of hOGG1 but not APE1 in human cells exposed to sub-lethal concentrations of cadmium. *Mutation Research/Fundamental and Molecular Mechanisms of Mutagenesis* **685**, 61–69 (2010).

52. Leung, A. K. L. *et al.* Poly(ADP-ribose) Regulates Stress Responses and microRNA Activity in the Cytoplasm. *Mol Cell* **42**, 489–499 (2011).

53. Repici, M. *et al.* The Parkinson’s Disease-Linked Protein DJ-1 Associates with Cytoplasmic mRNP Granules During Stress and Neurodegeneration. *Mol Neurobiol* **56**, 61–77 (2019).

54. Bai, Y. *et al.* Pdcd4 Is Involved in the Formation of Stress Granule in Response to Oxidized Low-Density Lipoprotein or High-Fat Diet. *PLoS One* **11**, e0159568 (2016).

55. Hofmann, I. *et al.* Identification of the Junctional Plaque Protein Plakophilin 3 in Cytoplasmic Particles Containing RNA-binding Proteins and the Recruitment of Plakophilins 1 and 3 to Stress Granules. *Mol Biol Cell* **17**, 1388–1398 (2006).

56. Hua, Y. & Zhou, J. Rpp20 interacts with SMN and is re-distributed into SMN granules in response to stress. *Biochemical and Biophysical Research Communications* **314**, 268–276 (2004).

57. Kunde, S. A. *et al.* The X-chromosome-linked intellectual disability protein PQBP1 is a component of neuronal RNA granules and regulates the appearance of stress granules. *Human Molecular Genetics* **20**, 4916–4931 (2011).

58. Kobayashi, T., Winslow, S., Sunesson, L., Hellman, U. & Larsson, C. PKCα Binds G3BP2 and Regulates Stress Granule Formation Following Cellular Stress. *PLoS One* **7**, e35820 (2012).

59. Yang, F. *et al.* Polysome-Bound Endonuclease PMR1 Is Targeted to Stress Granules via Stress-Specific Binding to TIA-1. *Mol Cell Biol* **26**, 8803–8813 (2006).

60. Park, C. *et al.* Stress Granules Contain Rbfox2 with Cell Cycle-related mRNAs. *Sci Rep* **7**, 11211 (2017).

61. Fukuda, T., Naiki, T., Saito, M. & Irie, K. hnRNP K interacts with RNA binding motif protein 42 and functions in the maintenance of cellular ATP level during stress conditions. *Genes to Cells* **14**, 113–128 (2009).

62. Bakkar, N., Kousari, A., Kovalik, T., Li, Y. & Bowser, R. RBM45 Modulates the Antioxidant Response in Amyotrophic Lateral Sclerosis through Interactions with KEAP1. *Mol Cell Biol* **35**, 2385–2399 (2015).

63. Farazi, T. A. *et al.* Identification of the RNA recognition element of the RBPMS family of RNA-binding proteins and their transcriptome-wide mRNA targets. *RNA* **20**, 1090–1102 (2014).

64. Eisinger-Mathason, T. S. K. *et al.* Co-dependent functions of RSK2 and the apoptosis promoting factor, TIA-1, in stress granule assembly and cell survival. *Mol Cell* **31**, 722–736 (2008).

65. Sfakianos, A. P. *et al.* The mTOR-S6 kinase pathway promotes stress granule assembly. *Cell Death Differ* **25**, 1766–1780 (2018).

66. Kedersha, N. *et al.* Evidence That Ternary Complex (eIF2-GTP-tRNAiMet)–Deficient Preinitiation Complexes Are Core Constituents of Mammalian Stress Granules. *Mol Biol Cell* **13**, 195–210 (2002).

67. Baez, M. V. & Boccaccio, G. L. Mammalian Smaug Is a Translational Repressor That Forms Cytoplasmic Foci Similar to Stress Granules *. *Journal of Biological Chemistry* **280**, 43131–43140 (2005).

68. Omer, A. *et al.* Stress granules counteract senescence by sequestration of PAI‐1. *EMBO Rep* **19**, e44722 (2018).

69. Jedrusik-Bode, M. *et al.* The sirtuin SIRT6 regulates stress granule formation in C. elegans and mammals. *Journal of Cell Science* **126**, 5166–5177 (2013).

70. Chang, Y.-W. & Huang, Y.-S. Arsenite-Activated JNK Signaling Enhances CPEB4-Vinexin Interaction to Facilitate Stress Granule Assembly and Cell Survival. *PLoS One* **9**, e107961 (2014).

71. Berger, A. *et al.* Direct binding of the Alu binding protein dimer SRP9/14 to 40S ribosomal subunits promotes stress granule formation and is regulated by Alu RNA. *Nucleic Acids Res* **42**, 11203–11217 (2014).

72. Kukharsky, M. S. *et al.* Calcium-responsive transactivator (CREST) protein shares a set of structural and functional traits with other proteins associated with amyotrophic lateral sclerosis. *Mol Neurodegener* **10**, 20 (2015).

73. Krisenko, M. O. *et al.* Syk Is Recruited to Stress Granules and Promotes Their Clearance through Autophagy. *J Biol Chem* **290**, 27803–27815 (2015).

74. Iannilli, F., Zalfa, F., Gartner, A., Bagni, C. & Dotti, C. G. Cytoplasmic TERT Associates to RNA Granules in Fully Mature Neurons: Role in the Translational Control of the Cell Cycle Inhibitor p15INK4B. *PLoS One* **8**, e66602 (2013).

75. Kim, W. J., Back, S. H., Kim, V., Ryu, I. & Jang, S. K. Sequestration of TRAF2 into Stress Granules Interrupts Tumor Necrosis Factor Signaling under Stress Conditions. *Mol Cell Biol* **25**, 2450–2462 (2005).

76. Schaefer, M. *et al.* RNA methylation by Dnmt2 protects transfer RNAs against stress-induced cleavage. *Genes Dev* **24**, 1590–1595 (2010).

77. Dao, T. P. *et al.* Ubiquitin modulates liquid-liquid phase separation of UBQLN2 via disruption of multivalent interactions. *Mol Cell* **69**, 965-978.e6 (2018).

78. Wang, B. *et al.* ULK1/2 Regulates Stress Granule Disassembly Through Phosphorylation and Activation of VCP/p97. *Mol Cell* **74**, 742-757.e8 (2019).

79. Brown, J. A. L. *et al.* A Novel Role for hSMG-1 in Stress Granule Formation ▿. *Mol Cell Biol* **31**, 4417–4429 (2011).

80. Narayanan, N., Wang, Z., Li, L. & Yang, Y. Arginine methylation of USP9X promotes its interaction with TDRD3 and its anti-apoptotic activities in breast cancer cells. *Cell Discov* **3**, 16048 (2017).

81. Xie, X. *et al.* Deubiquitylases USP5 and USP13 are recruited to and regulate heat-induced stress granules through their deubiquitylating activities. *Journal of Cell Science* **131**, jcs210856 (2018).

82. Deigendesch, N., Koch-Nolte, F. & Rothenburg, S. ZBP1 subcellular localization and association with stress granules is controlled by its Z-DNA binding domains. *Nucleic Acids Res* **34**, 5007–5020 (2006).

83. Turakhiya, A. *et al.* ZFAND1 Recruits p97 and the 26S Proteasome to Promote the Clearance of Arsenite-Induced Stress Granules. *Molecular Cell* **70**, 906-919.e7 (2018).
