# Supplementary material for: Targeting NUPR1-dependent stress granules formation to induce synthetic lethality in KrasG12D-driven tumors
Source: EMBO Mol Med. 2024 Feb 15;16(3):4. doi: 10.1038/s44321-024-00032-2 (PMC10940650; doi:10.1038/s44321-024-00032-2)
Supplement: Supplementary file 5 — Dataset EV4 [file 44321_2024_32_MOESM5_ESM.docx]

**DatasetEV4: NUPR1-Flag interactors upon Thapsigargin-treatment.** In green, interactors identified to localize to stress granules

| **Gene ID** | **Description** |
| --- | --- |
| AAAS | Aladin OS=Homo sapiens GN=AAAS PE=1 SV=1 - [AAAS_HUMAN] |
| ABCF1 | ATP-binding cassette sub-family F member 1 OS=Homo sapiens GN=ABCF1 PE=1 SV=2 - [ABCF1_HUMAN] |
| ACIN1 | Apoptotic chromatin condensation inducer in the nucleus OS=Homo sapiens GN=ACIN1 PE=1 SV=2 - [ACINU_HUMAN] |
| ACOT9 | Acyl-coenzyme A thioesterase 9, mitochondrial OS=Homo sapiens GN=ACOT9 PE=1 SV=2 - [ACOT9_HUMAN] |
| ACTB | Actin, cytoplasmic 1 OS=Homo sapiens GN=ACTB PE=1 SV=1 - [ACTB_HUMAN] |
| ACTBL2 | Beta-actin-like protein 2 OS=Homo sapiens GN=ACTBL2 PE=1 SV=2 - [ACTBL_HUMAN] |
| ACTC1 | Actin, alpha cardiac muscle 1 OS=Homo sapiens GN=ACTC1 PE=1 SV=1 - [ACTC_HUMAN] |
| ACTG1 | Actin, cytoplasmic 2 OS=Homo sapiens GN=ACTG1 PE=1 SV=1 - [ACTG_HUMAN] |
| ACTN1 | Alpha-actinin-1 OS=Homo sapiens GN=ACTN1 PE=1 SV=2 - [ACTN1_HUMAN] |
| ACTN4 | Alpha-actinin-4 OS=Homo sapiens GN=ACTN4 PE=1 SV=2 - [ACTN4_HUMAN] |
| ACTR2 | Actin-related protein 2 OS=Homo sapiens GN=ACTR2 PE=1 SV=1 - [ARP2_HUMAN] |
| ACTR3 | Actin-related protein 3 OS=Homo sapiens GN=ACTR3 PE=1 SV=3 - [ARP3_HUMAN] |
| ACTR3B | Actin-related protein 3B OS=Homo sapiens GN=ACTR3B PE=2 SV=1 - [ARP3B_HUMAN] |
| ADAR | Double-stranded RNA-specific adenosine deaminase OS=Homo sapiens GN=ADAR PE=1 SV=4 - [DSRAD_HUMAN] |
| ADRM1 | Proteasomal ubiquitin receptor ADRM1 OS=Homo sapiens GN=ADRM1 PE=1 SV=2 - [ADRM1_HUMAN] |
| AFAP1 | Actin filament-associated protein 1 OS=Homo sapiens GN=AFAP1 PE=1 SV=2 - [AFAP1_HUMAN] |
| AHNAK | Neuroblast differentiation-associated protein AHNAK OS=Homo sapiens GN=AHNAK PE=1 SV=2 - [AHNK_HUMAN] |
| AIF1L | Allograft inflammatory factor 1-like OS=Homo sapiens GN=AIF1L PE=1 SV=1 - [AIF1L_HUMAN] |
| ALDOA | Fructose-bisphosphate aldolase A OS=Homo sapiens GN=ALDOA PE=1 SV=2 - [ALDOA_HUMAN] |
| ALYREF | THO complex subunit 4 OS=Homo sapiens GN=ALYREF PE=1 SV=3 - [THOC4_HUMAN] |
| ANXA2 | Annexin A2 OS=Homo sapiens GN=ANXA2 PE=1 SV=2 - [ANXA2_HUMAN] |
| AP2A1 | AP-2 complex subunit alpha-1 OS=Homo sapiens GN=AP2A1 PE=1 SV=3 - [AP2A1_HUMAN] |
| AP2A2 | AP-2 complex subunit alpha-2 OS=Homo sapiens GN=AP2A2 PE=1 SV=2 - [AP2A2_HUMAN] |
| AP2B1 | AP-2 complex subunit beta OS=Homo sapiens GN=AP2B1 PE=1 SV=1 - [AP2B1_HUMAN] |
| AP2M1 | AP-2 complex subunit mu OS=Homo sapiens GN=AP2M1 PE=1 SV=2 - [AP2M1_HUMAN] |
| AP2S1 | AP-2 complex subunit sigma OS=Homo sapiens GN=AP2S1 PE=1 SV=2 - [AP2S1_HUMAN] |
| API5 | Apoptosis inhibitor 5 OS=Homo sapiens GN=API5 PE=1 SV=3 - [API5_HUMAN] |
| APOBEC3B | DNA dC->dU-editing enzyme APOBEC-3B OS=Homo sapiens GN=APOBEC3B PE=1 SV=1 - [ABC3B_HUMAN] |
| APOBEC3C | DNA dC->dU-editing enzyme APOBEC-3C OS=Homo sapiens GN=APOBEC3C PE=1 SV=2 - [ABC3C_HUMAN] |
| AQR | Intron-binding protein aquarius OS=Homo sapiens GN=AQR PE=1 SV=4 - [AQR_HUMAN] |
| ARF3 | ADP-ribosylation factor 3 OS=Homo sapiens GN=ARF3 PE=1 SV=2 - [ARF3_HUMAN] |
| ARF6 | ADP-ribosylation factor 6 OS=Homo sapiens GN=ARF6 PE=1 SV=2 - [ARF6_HUMAN] |
| ARHGEF11 | Rho guanine nucleotide exchange factor 11 OS=Homo sapiens GN=ARHGEF11 PE=1 SV=1 - [ARHGB_HUMAN] |
| ARL6IP4 | ADP-ribosylation factor-like protein 6-interacting protein 4 OS=Homo sapiens GN=ARL6IP4 PE=1 SV=2 - [AR6P4_HUMAN] |
| ARPC1A | Actin-related protein 2/3 complex subunit 1A OS=Homo sapiens GN=ARPC1A PE=1 SV=2 - [ARC1A_HUMAN] |
| ARPC1B | Actin-related protein 2/3 complex subunit 1B OS=Homo sapiens GN=ARPC1B PE=1 SV=3 - [ARC1B_HUMAN] |
| ARPC2 | Actin-related protein 2/3 complex subunit 2 OS=Homo sapiens GN=ARPC2 PE=1 SV=1 - [ARPC2_HUMAN] |
| ARPC3 | Actin-related protein 2/3 complex subunit 3 OS=Homo sapiens GN=ARPC3 PE=1 SV=3 - [ARPC3_HUMAN] |
| ARPC4 | Actin-related protein 2/3 complex subunit 4 OS=Homo sapiens GN=ARPC4 PE=1 SV=3 - [ARPC4_HUMAN] |
| ARPC5 | Actin-related protein 2/3 complex subunit 5 OS=Homo sapiens GN=ARPC5 PE=1 SV=3 - [ARPC5_HUMAN] |
| ARPC5L | Actin-related protein 2/3 complex subunit 5-like protein OS=Homo sapiens GN=ARPC5L PE=1 SV=1 - [ARP5L_HUMAN] |
| ARRB2 | Beta-arrestin-2 OS=Homo sapiens GN=ARRB2 PE=1 SV=2 - [ARRB2_HUMAN] |
| ATAD3A | ATPase family AAA domain-containing protein 3A OS=Homo sapiens GN=ATAD3A PE=1 SV=2 - [ATD3A_HUMAN] |
| ATP6V1A | V-type proton ATPase catalytic subunit A OS=Homo sapiens GN=ATP6V1A PE=1 SV=2 - [VATA_HUMAN] |
| ATP6V1B2 | V-type proton ATPase subunit B, brain isoform OS=Homo sapiens GN=ATP6V1B2 PE=1 SV=3 - [VATB2_HUMAN] |
| ATXN2L | Ataxin-2-like protein OS=Homo sapiens GN=ATXN2L PE=1 SV=2 - [ATX2L_HUMAN] |
| BAIAP2 | Brain-specific angiogenesis inhibitor 1-associated protein 2 OS=Homo sapiens GN=BAIAP2 PE=1 SV=1 - [BAIP2_HUMAN] |
| BAZ1B | Tyrosine-protein kinase BAZ1B OS=Homo sapiens GN=BAZ1B PE=1 SV=2 - [BAZ1B_HUMAN] |
| BCLAF1 | Bcl-2-associated transcription factor 1 OS=Homo sapiens GN=BCLAF1 PE=1 SV=2 - [BCLF1_HUMAN] |
| BEND3 | BEN domain-containing protein 3 OS=Homo sapiens GN=BEND3 PE=1 SV=1 - [BEND3_HUMAN] |
| BOP1 | Ribosome biogenesis protein BOP1 OS=Homo sapiens GN=BOP1 PE=1 SV=2 - [BOP1_HUMAN] |
| BRD2 | Bromodomain-containing protein 2 OS=Homo sapiens GN=BRD2 PE=1 SV=2 - [BRD2_HUMAN] |
| BRD3 | Bromodomain-containing protein 3 OS=Homo sapiens GN=BRD3 PE=1 SV=1 - [BRD3_HUMAN] |
| BRD4 | Bromodomain-containing protein 4 OS=Homo sapiens GN=BRD4 PE=1 SV=2 - [BRD4_HUMAN] |
| BRIX1 | Ribosome biogenesis protein BRX1 homolog OS=Homo sapiens GN=BRIX1 PE=1 SV=2 - [BRX1_HUMAN] |
| BUB3 | Mitotic checkpoint protein BUB3 OS=Homo sapiens GN=BUB3 PE=1 SV=1 - [BUB3_HUMAN] |
| BUD31 | Protein BUD31 homolog OS=Homo sapiens GN=BUD31 PE=1 SV=2 - [BUD31_HUMAN] |
| BYSL | Bystin OS=Homo sapiens GN=BYSL PE=1 SV=3 - [BYST_HUMAN] |
| C1QBP | Complement component 1 Q subcomponent-binding protein, mitochondrial OS=Homo sapiens GN=C1QBP PE=1 SV=1 - [C1QBP_HUMAN] |
| CAD | CAD protein OS=Homo sapiens GN=CAD PE=1 SV=3 - [PYR1_HUMAN] |
| CALD1 | Caldesmon OS=Homo sapiens GN=CALD1 PE=1 SV=3 - [CALD1_HUMAN] |
| CALM1 | Calmodulin OS=Homo sapiens GN=CALM1 PE=1 SV=2 - [CALM_HUMAN] |
| CAPRIN1 | Caprin-1 OS=Homo sapiens GN=CAPRIN1 PE=1 SV=2 - [CAPR1_HUMAN] |
| CAPZA1 | F-actin-capping protein subunit alpha-1 OS=Homo sapiens GN=CAPZA1 PE=1 SV=3 - [CAZA1_HUMAN] |
| CAPZA2 | F-actin-capping protein subunit alpha-2 OS=Homo sapiens GN=CAPZA2 PE=1 SV=3 - [CAZA2_HUMAN] |
| CAPZB | F-actin-capping protein subunit beta OS=Homo sapiens GN=CAPZB PE=1 SV=4 - [CAPZB_HUMAN] |
| CASK | Peripheral plasma membrane protein CASK OS=Homo sapiens GN=CASK PE=1 SV=3 - [CSKP_HUMAN] |
| CAV1 | Caveolin-1 OS=Homo sapiens GN=CAV1 PE=1 SV=4 - [CAV1_HUMAN] |
| CAVIN1 | Polymerase I and transcript release factor OS=Homo sapiens GN=PTRF PE=1 SV=1 - [PTRF_HUMAN] |
| CAVIN2 | Serum deprivation-response protein OS=Homo sapiens GN=SDPR PE=1 SV=3 - [SDPR_HUMAN] |
| CBX1 | Chromobox protein homolog 1 OS=Homo sapiens GN=CBX1 PE=1 SV=1 - [CBX1_HUMAN] |
| CBX3 | Chromobox protein homolog 3 OS=Homo sapiens GN=CBX3 PE=1 SV=4 - [CBX3_HUMAN] |
| CBX5 | Chromobox protein homolog 5 OS=Homo sapiens GN=CBX5 PE=1 SV=1 - [CBX5_HUMAN] |
| CCAR1 | Cell division cycle and apoptosis regulator protein 1 OS=Homo sapiens GN=CCAR1 PE=1 SV=2 - [CCAR1_HUMAN] |
| CCAR2 | Cell cycle and apoptosis regulator protein 2 OS=Homo sapiens GN=CCAR2 PE=1 SV=2 - [CCAR2_HUMAN] |
| CCDC124 | Coiled-coil domain-containing protein 124 OS=Homo sapiens GN=CCDC124 PE=1 SV=1 - [CC124_HUMAN] |
| CCDC50 | Coiled-coil domain-containing protein 50 OS=Homo sapiens GN=CCDC50 PE=1 SV=1 - [CCD50_HUMAN] |
| CCDC86 | Coiled-coil domain-containing protein 86 OS=Homo sapiens GN=CCDC86 PE=1 SV=1 - [CCD86_HUMAN] |
| CCNY | Cyclin-Y OS=Homo sapiens GN=CCNY PE=1 SV=2 - [CCNY_HUMAN] |
| CCT2 | T-complex protein 1 subunit beta OS=Homo sapiens GN=CCT2 PE=1 SV=4 - [TCPB_HUMAN] |
| CCT3 | T-complex protein 1 subunit gamma OS=Homo sapiens GN=CCT3 PE=1 SV=4 - [TCPG_HUMAN] |
| CCT4 | T-complex protein 1 subunit delta OS=Homo sapiens GN=CCT4 PE=1 SV=4 - [TCPD_HUMAN] |
| CCT5 | T-complex protein 1 subunit epsilon OS=Homo sapiens GN=CCT5 PE=1 SV=1 - [TCPE_HUMAN] |
| CCT7 | T-complex protein 1 subunit eta OS=Homo sapiens GN=CCT7 PE=1 SV=2 - [TCPH_HUMAN] |
| CCT8 | T-complex protein 1 subunit theta OS=Homo sapiens GN=CCT8 PE=1 SV=4 - [TCPQ_HUMAN] |
| CD109 | CD109 antigen OS=Homo sapiens GN=CD109 PE=1 SV=2 - [CD109_HUMAN] |
| CD2BP2 | CD2 antigen cytoplasmic tail-binding protein 2 OS=Homo sapiens GN=CD2BP2 PE=1 SV=1 - [CD2B2_HUMAN] |
| CD44 | CD44 antigen OS=Homo sapiens GN=CD44 PE=1 SV=3 - [CD44_HUMAN] |
| CD59 | CD59 glycoprotein OS=Homo sapiens GN=CD59 PE=1 SV=1 - [CD59_HUMAN] |
| CDC40 | Pre-mRNA-processing factor 17 OS=Homo sapiens GN=CDC40 PE=1 SV=1 - [PRP17_HUMAN] |
| CDC42 | Cell division control protein 42 homolog OS=Homo sapiens GN=CDC42 PE=1 SV=2 - [CDC42_HUMAN] |
| CDC5L | Cell division cycle 5-like protein OS=Homo sapiens GN=CDC5L PE=1 SV=2 - [CDC5L_HUMAN] |
| CDC73 | Parafibromin OS=Homo sapiens GN=CDC73 PE=1 SV=1 - [CDC73_HUMAN] |
| CDK11A | Cyclin-dependent kinase 11A OS=Homo sapiens GN=CDK11A PE=1 SV=4 - [CD11A_HUMAN] |
| CDK6 | Cyclin-dependent kinase 6 OS=Homo sapiens GN=CDK6 PE=1 SV=1 - [CDK6_HUMAN] |
| CDK9 | Cyclin-dependent kinase 9 OS=Homo sapiens GN=CDK9 PE=1 SV=3 - [CDK9_HUMAN] |
| CENPV | Centromere protein V OS=Homo sapiens GN=CENPV PE=1 SV=1 - [CENPV_HUMAN] |
| CFAP20 | UPF0468 protein C16orf80 OS=Homo sapiens GN=C16orf80 PE=1 SV=1 - [CP080_HUMAN] |
| CFL1 | Cofilin-1 OS=Homo sapiens GN=CFL1 PE=1 SV=3 - [COF1_HUMAN] |
| CFL2 | Cofilin-2 OS=Homo sapiens GN=CFL2 PE=1 SV=1 - [COF2_HUMAN] |
| CHCHD3 | Coiled-coil-helix-coiled-coil-helix domain-containing protein 3, mitochondrial OS=Homo sapiens GN=CHCHD3 PE=1 SV=1 - [CHCH3_HUMAN] |
| CHERP | Calcium homeostasis endoplasmic reticulum protein OS=Homo sapiens GN=CHERP PE=1 SV=3 - [CHERP_HUMAN] |
| CLIC1 | Chloride intracellular channel protein 1 OS=Homo sapiens GN=CLIC1 PE=1 SV=4 - [CLIC1_HUMAN] |
| CLINT1 | Clathrin interactor 1 OS=Homo sapiens GN=CLINT1 PE=1 SV=1 - [EPN4_HUMAN] |
| CLTC | Clathrin heavy chain 1 OS=Homo sapiens GN=CLTC PE=1 SV=5 - [CLH1_HUMAN] |
| CNP | 2',3'-cyclic-nucleotide 3'-phosphodiesterase OS=Homo sapiens GN=CNP PE=1 SV=2 - [CN37_HUMAN] |
| COASY | Bifunctional coenzyme A synthase OS=Homo sapiens GN=COASY PE=1 SV=4 - [COASY_HUMAN] |
| COIL | Coilin OS=Homo sapiens GN=COIL PE=1 SV=1 - [COIL_HUMAN] |
| COLGALT1 | Procollagen galactosyltransferase 1 OS=Homo sapiens GN=COLGALT1 PE=1 SV=1 - [GT251_HUMAN] |
| CORO1C | Coronin-1C OS=Homo sapiens GN=CORO1C PE=1 SV=1 - [COR1C_HUMAN] |
| CPSF1 | Cleavage and polyadenylation specificity factor subunit 1 OS=Homo sapiens GN=CPSF1 PE=1 SV=2 - [CPSF1_HUMAN] |
| CPSF2 | Cleavage and polyadenylation specificity factor subunit 2 OS=Homo sapiens GN=CPSF2 PE=1 SV=2 - [CPSF2_HUMAN] |
| CPSF3 | Cleavage and polyadenylation specificity factor subunit 3 OS=Homo sapiens GN=CPSF3 PE=1 SV=1 - [CPSF3_HUMAN] |
| CROCC | Rootletin OS=Homo sapiens GN=CROCC PE=1 SV=1 - [CROCC_HUMAN] |
| CRTAP | Cartilage-associated protein OS=Homo sapiens GN=CRTAP PE=1 SV=1 - [CRTAP_HUMAN] |
| CSNK1A1 | Casein kinase I isoform alpha OS=Homo sapiens GN=CSNK1A1 PE=1 SV=2 - [KC1A_HUMAN] |
| CSNK1G1 | Casein kinase I isoform gamma-1 OS=Homo sapiens GN=CSNK1G1 PE=1 SV=1 - [KC1G1_HUMAN] |
| CSNK2A1 | Casein kinase II subunit alpha OS=Homo sapiens GN=CSNK2A1 PE=1 SV=1 - [CSK21_HUMAN] |
| CSNK2A2 | Casein kinase II subunit alpha' OS=Homo sapiens GN=CSNK2A2 PE=1 SV=1 - [CSK22_HUMAN] |
| CSNK2B | Casein kinase II subunit beta OS=Homo sapiens GN=CSNK2B PE=1 SV=1 - [CSK2B_HUMAN] |
| CSTF1 | Cleavage stimulation factor subunit 1 OS=Homo sapiens GN=CSTF1 PE=1 SV=1 - [CSTF1_HUMAN] |
| CSTF2 | Cleavage stimulation factor subunit 2 OS=Homo sapiens GN=CSTF2 PE=1 SV=1 - [CSTF2_HUMAN] |
| CSTF3 | Cleavage stimulation factor subunit 3 OS=Homo sapiens GN=CSTF3 PE=1 SV=1 - [CSTF3_HUMAN] |
| CTBP1 | C-terminal-binding protein 1 OS=Homo sapiens GN=CTBP1 PE=1 SV=2 - [CTBP1_HUMAN] |
| CTBP2 | C-terminal-binding protein 2 OS=Homo sapiens GN=CTBP2 PE=1 SV=1 - [CTBP2_HUMAN] |
| CTDSPL2 | CTD small phosphatase-like protein 2 OS=Homo sapiens GN=CTDSPL2 PE=1 SV=2 - [CTSL2_HUMAN] |
| CTNNBL1 | Beta-catenin-like protein 1 OS=Homo sapiens GN=CTNNBL1 PE=1 SV=1 - [CTBL1_HUMAN] |
| CTNND1 | Catenin delta-1 OS=Homo sapiens GN=CTNND1 PE=1 SV=1 - [CTND1_HUMAN] |
| CTR9 | RNA polymerase-associated protein CTR9 homolog OS=Homo sapiens GN=CTR9 PE=1 SV=1 - [CTR9_HUMAN] |
| CTSA | Lysosomal protective protein OS=Homo sapiens GN=CTSA PE=1 SV=2 - [PPGB_HUMAN] |
| CTTN | Src substrate cortactin OS=Homo sapiens GN=CTTN PE=1 SV=2 - [SRC8_HUMAN] |
| CWC22 | Pre-mRNA-splicing factor CWC22 homolog OS=Homo sapiens GN=CWC22 PE=1 SV=3 - [CWC22_HUMAN] |
| CWC27 | Peptidyl-prolyl cis-trans isomerase CWC27 homolog OS=Homo sapiens GN=CWC27 PE=1 SV=1 - [CWC27_HUMAN] |
| CYBRD1 | Cytochrome b reductase 1 OS=Homo sapiens GN=CYBRD1 PE=1 SV=1 - [CYBR1_HUMAN] |
| DAPK3 | Death-associated protein kinase 3 OS=Homo sapiens GN=DAPK3 PE=1 SV=1 - [DAPK3_HUMAN] |
| DAZAP1 | DAZ-associated protein 1 OS=Homo sapiens GN=DAZAP1 PE=1 SV=1 - [DAZP1_HUMAN] |
| DBN1 | Drebrin OS=Homo sapiens GN=DBN1 PE=1 SV=4 - [DREB_HUMAN] |
| DDB1 | DNA damage-binding protein 1 OS=Homo sapiens GN=DDB1 PE=1 SV=1 - [DDB1_HUMAN] |
| DDX1 | ATP-dependent RNA helicase DDX1 OS=Homo sapiens GN=DDX1 PE=1 SV=2 - [DDX1_HUMAN] |
| DDX17 | Probable ATP-dependent RNA helicase DDX17 OS=Homo sapiens GN=DDX17 PE=1 SV=2 - [DDX17_HUMAN] |
| DDX18 | ATP-dependent RNA helicase DDX18 OS=Homo sapiens GN=DDX18 PE=1 SV=2 - [DDX18_HUMAN] |
| DDX21 | Nucleolar RNA helicase 2 OS=Homo sapiens GN=DDX21 PE=1 SV=5 - [DDX21_HUMAN] |
| DDX23 | Probable ATP-dependent RNA helicase DDX23 OS=Homo sapiens GN=DDX23 PE=1 SV=3 - [DDX23_HUMAN] |
| DDX24 | ATP-dependent RNA helicase DDX24 OS=Homo sapiens GN=DDX24 PE=1 SV=1 - [DDX24_HUMAN] |
| DDX39A | ATP-dependent RNA helicase DDX39A OS=Homo sapiens GN=DDX39A PE=1 SV=2 - [DX39A_HUMAN] |
| DDX39B | Spliceosome RNA helicase DDX39B OS=Homo sapiens GN=DDX39B PE=1 SV=1 - [DX39B_HUMAN] |
| DDX3X | ATP-dependent RNA helicase DDX3X OS=Homo sapiens GN=DDX3X PE=1 SV=3 - [DDX3X_HUMAN] |
| DDX41 | Probable ATP-dependent RNA helicase DDX41 OS=Homo sapiens GN=DDX41 PE=1 SV=2 - [DDX41_HUMAN] |
| DDX42 | ATP-dependent RNA helicase DDX42 OS=Homo sapiens GN=DDX42 PE=1 SV=1 - [DDX42_HUMAN] |
| DDX46 | Probable ATP-dependent RNA helicase DDX46 OS=Homo sapiens GN=DDX46 PE=1 SV=2 - [DDX46_HUMAN] |
| DDX47 | Probable ATP-dependent RNA helicase DDX47 OS=Homo sapiens GN=DDX47 PE=1 SV=1 - [DDX47_HUMAN] |
| DDX5 | Probable ATP-dependent RNA helicase DDX5 OS=Homo sapiens GN=DDX5 PE=1 SV=1 - [DDX5_HUMAN] |
| DDX56 | Probable ATP-dependent RNA helicase DDX56 OS=Homo sapiens GN=DDX56 PE=1 SV=1 - [DDX56_HUMAN] |
| DDX6 | Probable ATP-dependent RNA helicase DDX6 OS=Homo sapiens GN=DDX6 PE=1 SV=2 - [DDX6_HUMAN] |
| DECR1 | 2,4-dienoyl-CoA reductase, mitochondrial OS=Homo sapiens GN=DECR1 PE=1 SV=1 - [DECR_HUMAN] |
| DEK | Protein DEK OS=Homo sapiens GN=DEK PE=1 SV=1 - [DEK_HUMAN] |
| DES | Desmin OS=Homo sapiens GN=DES PE=1 SV=3 - [DESM_HUMAN] |
| DHX15 | Putative pre-mRNA-splicing factor ATP-dependent RNA helicase DHX15 OS=Homo sapiens GN=DHX15 PE=1 SV=2 - [DHX15_HUMAN] |
| DHX30 | Putative ATP-dependent RNA helicase DHX30 OS=Homo sapiens GN=DHX30 PE=1 SV=1 - [DHX30_HUMAN] |
| DHX38 | Pre-mRNA-splicing factor ATP-dependent RNA helicase PRP16 OS=Homo sapiens GN=DHX38 PE=1 SV=2 - [PRP16_HUMAN] |
| DHX8 | ATP-dependent RNA helicase DHX8 OS=Homo sapiens GN=DHX8 PE=1 SV=1 - [DHX8_HUMAN] |
| DHX9 | ATP-dependent RNA helicase A OS=Homo sapiens GN=DHX9 PE=1 SV=4 - [DHX9_HUMAN] |
| DIEXF | Digestive organ expansion factor homolog OS=Homo sapiens GN=DIEXF PE=1 SV=2 - [DIEXF_HUMAN] |
| DIS3 | Exosome complex exonuclease RRP44 OS=Homo sapiens GN=DIS3 PE=1 SV=2 - [RRP44_HUMAN] |
| DKC1 | H/ACA ribonucleoprotein complex subunit 4 OS=Homo sapiens GN=DKC1 PE=1 SV=3 - [DKC1_HUMAN] |
| DLD | Dihydrolipoyl dehydrogenase, mitochondrial OS=Homo sapiens GN=DLD PE=1 SV=2 - [DLDH_HUMAN] |
| DLG1 | Disks large homolog 1 OS=Homo sapiens GN=DLG1 PE=1 SV=2 - [DLG1_HUMAN] |
| DLST | Dihydrolipoyllysine-residue succinyltransferase component of 2-oxoglutarate dehydrogenase complex, mitochondrial OS=Homo sapiens GN=DLST PE=1 SV=4 - [ODO2_HUMAN] |
| DNAJA2 | DnaJ homolog subfamily A member 2 OS=Homo sapiens GN=DNAJA2 PE=1 SV=1 - [DNJA2_HUMAN] |
| DNAJB6 | DnaJ homolog subfamily B member 6 OS=Homo sapiens GN=DNAJB6 PE=1 SV=2 - [DNJB6_HUMAN] |
| DNAJB7 | DnaJ homolog subfamily B member 7 OS=Homo sapiens GN=DNAJB7 PE=2 SV=2 - [DNJB7_HUMAN] |
| DNAJC8 | DnaJ homolog subfamily C member 8 OS=Homo sapiens GN=DNAJC8 PE=1 SV=2 - [DNJC8_HUMAN] |
| DNMT1 | DNA (cytosine-5)-methyltransferase 1 OS=Homo sapiens GN=DNMT1 PE=1 SV=2 - [DNMT1_HUMAN] |
| DNTTIP2 | Deoxynucleotidyltransferase terminal-interacting protein 2 OS=Homo sapiens GN=DNTTIP2 PE=1 SV=2 - [TDIF2_HUMAN] |
| DOCK7 | Dedicator of cytokinesis protein 7 OS=Homo sapiens GN=DOCK7 PE=1 SV=4 - [DOCK7_HUMAN] |
| DSG2 | Desmoglein-2 OS=Homo sapiens GN=DSG2 PE=1 SV=2 - [DSG2_HUMAN] |
| DYNC1H1 | Cytoplasmic dynein 1 heavy chain 1 OS=Homo sapiens GN=DYNC1H1 PE=1 SV=5 - [DYHC1_HUMAN] |
| DYNLL1 | Dynein light chain 1, cytoplasmic OS=Homo sapiens GN=DYNLL1 PE=1 SV=1 - [DYL1_HUMAN] |
| DYNLL2 | Dynein light chain 2, cytoplasmic OS=Homo sapiens GN=DYNLL2 PE=1 SV=1 - [DYL2_HUMAN] |
| EBNA1BP2 | Probable rRNA-processing protein EBP2 OS=Homo sapiens GN=EBNA1BP2 PE=1 SV=2 - [EBP2_HUMAN] |
| EED | Polycomb protein EED OS=Homo sapiens GN=EED PE=1 SV=2 - [EED_HUMAN] |
| EFHD2 | EF-hand domain-containing protein D2 OS=Homo sapiens GN=EFHD2 PE=1 SV=1 - [EFHD2_HUMAN] |
| EFR3A | Protein EFR3 homolog A OS=Homo sapiens GN=EFR3A PE=1 SV=2 - [EFR3A_HUMAN] |
| EFTUD2 | 116 kDa U5 small nuclear ribonucleoprotein component OS=Homo sapiens GN=EFTUD2 PE=1 SV=1 - [U5S1_HUMAN] |
| EIF1AX | Eukaryotic translation initiation factor 1A, X-chromosomal OS=Homo sapiens GN=EIF1AX PE=1 SV=2 - [IF1AX_HUMAN] |
| EIF2AK2 | Interferon-induced, double-stranded RNA-activated protein kinase OS=Homo sapiens GN=EIF2AK2 PE=1 SV=2 - [E2AK2_HUMAN] |
| EIF2S1 | Eukaryotic translation initiation factor 2 subunit 1 OS=Homo sapiens GN=EIF2S1 PE=1 SV=3 - [IF2A_HUMAN] |
| EIF2S2 | Eukaryotic translation initiation factor 2 subunit 2 OS=Homo sapiens GN=EIF2S2 PE=1 SV=2 - [IF2B_HUMAN] |
| EIF2S3 | Eukaryotic translation initiation factor 2 subunit 3 OS=Homo sapiens GN=EIF2S3 PE=1 SV=3 - [IF2G_HUMAN] |
| EIF3A | Eukaryotic translation initiation factor 3 subunit A OS=Homo sapiens GN=EIF3A PE=1 SV=1 - [EIF3A_HUMAN] |
| EIF3B | Eukaryotic translation initiation factor 3 subunit B OS=Homo sapiens GN=EIF3B PE=1 SV=3 - [EIF3B_HUMAN] |
| EIF3C | Eukaryotic translation initiation factor 3 subunit C OS=Homo sapiens GN=EIF3C PE=1 SV=1 - [EIF3C_HUMAN] |
| EIF3D | Eukaryotic translation initiation factor 3 subunit D OS=Homo sapiens GN=EIF3D PE=1 SV=1 - [EIF3D_HUMAN] |
| EIF3E | Eukaryotic translation initiation factor 3 subunit E OS=Homo sapiens GN=EIF3E PE=1 SV=1 - [EIF3E_HUMAN] |
| EIF3F | Eukaryotic translation initiation factor 3 subunit F OS=Homo sapiens GN=EIF3F PE=1 SV=1 - [EIF3F_HUMAN] |
| EIF3I | Eukaryotic translation initiation factor 3 subunit I OS=Homo sapiens GN=EIF3I PE=1 SV=1 - [EIF3I_HUMAN] |
| EIF3L | Eukaryotic translation initiation factor 3 subunit L OS=Homo sapiens GN=EIF3L PE=1 SV=1 - [EIF3L_HUMAN] |
| EIF3M | Eukaryotic translation initiation factor 3 subunit M OS=Homo sapiens GN=EIF3M PE=1 SV=1 - [EIF3M_HUMAN] |
| EIF4A1 | Eukaryotic initiation factor 4A-I OS=Homo sapiens GN=EIF4A1 PE=1 SV=1 - [IF4A1_HUMAN] |
| EIF4A3 | Eukaryotic initiation factor 4A-III OS=Homo sapiens GN=EIF4A3 PE=1 SV=4 - [IF4A3_HUMAN] |
| EIF4G1 | Eukaryotic translation initiation factor 4 gamma 1 OS=Homo sapiens GN=EIF4G1 PE=1 SV=4 - [IF4G1_HUMAN] |
| EIF5A2 | Eukaryotic translation initiation factor 5A-2 OS=Homo sapiens GN=EIF5A2 PE=1 SV=3 - [IF5A2_HUMAN] |
| EIF5B | Eukaryotic translation initiation factor 5B OS=Homo sapiens GN=EIF5B PE=1 SV=4 - [IF2P_HUMAN] |
| EIF6 | Eukaryotic translation initiation factor 6 OS=Homo sapiens GN=EIF6 PE=1 SV=1 - [IF6_HUMAN] |
| ELAVL1 | ELAV-like protein 1 OS=Homo sapiens GN=ELAVL1 PE=1 SV=2 - [ELAV1_HUMAN] |
| EMG1 | Ribosomal RNA small subunit methyltransferase NEP1 OS=Homo sapiens GN=EMG1 PE=1 SV=4 - [NEP1_HUMAN] |
| EMP3 | Epithelial membrane protein 3 OS=Homo sapiens GN=EMP3 PE=2 SV=1 - [EMP3_HUMAN] |
| ENO1 | Alpha-enolase OS=Homo sapiens GN=ENO1 PE=1 SV=2 - [ENOA_HUMAN] |
| EPB41L2 | Band 4.1-like protein 2 OS=Homo sapiens GN=EPB41L2 PE=1 SV=1 - [E41L2_HUMAN] |
| EPHA2 | Ephrin type-A receptor 2 OS=Homo sapiens GN=EPHA2 PE=1 SV=2 - [EPHA2_HUMAN] |
| EPS8 | Epidermal growth factor receptor kinase substrate 8 OS=Homo sapiens GN=EPS8 PE=1 SV=1 - [EPS8_HUMAN] |
| ERCC2 | TFIIH basal transcription factor complex helicase XPD subunit OS=Homo sapiens GN=ERCC2 PE=1 SV=1 - [ERCC2_HUMAN] |
| ERCC3 | TFIIH basal transcription factor complex helicase XPB subunit OS=Homo sapiens GN=ERCC3 PE=1 SV=1 - [ERCC3_HUMAN] |
| ERH | Enhancer of rudimentary homolog OS=Homo sapiens GN=ERH PE=1 SV=1 - [ERH_HUMAN] |
| ERLIN1 | Erlin-1 OS=Homo sapiens GN=ERLIN1 PE=1 SV=1 - [ERLN1_HUMAN] |
| ERLIN2 | Erlin-2 OS=Homo sapiens GN=ERLIN2 PE=1 SV=1 - [ERLN2_HUMAN] |
| ESF1 | ESF1 homolog OS=Homo sapiens GN=ESF1 PE=1 SV=1 - [ESF1_HUMAN] |
| EXOSC10 | Exosome component 10 OS=Homo sapiens GN=EXOSC10 PE=1 SV=2 - [EXOSX_HUMAN] |
| EXOSC2 | Exosome complex component RRP4 OS=Homo sapiens GN=EXOSC2 PE=1 SV=2 - [EXOS2_HUMAN] |
| EXOSC7 | Exosome complex component RRP42 OS=Homo sapiens GN=EXOSC7 PE=1 SV=3 - [EXOS7_HUMAN] |
| EXOSC9 | Exosome complex component RRP45 OS=Homo sapiens GN=EXOSC9 PE=1 SV=3 - [EXOS9_HUMAN] |
| EZH2 | Histone-lysine N-methyltransferase EZH2 OS=Homo sapiens GN=EZH2 PE=1 SV=2 - [EZH2_HUMAN] |
| EZR | Ezrin OS=Homo sapiens GN=EZR PE=1 SV=4 - [EZRI_HUMAN] |
| F2 | Prothrombin OS=Homo sapiens GN=F2 PE=1 SV=2 - [THRB_HUMAN] |
| F5 | Coagulation factor V OS=Homo sapiens GN=F5 PE=1 SV=4 - [FA5_HUMAN] |
| FAM120A | Constitutive coactivator of PPAR-gamma-like protein 1 OS=Homo sapiens GN=FAM120A PE=1 SV=2 - [F120A_HUMAN] |
| FAM126A | Hyccin OS=Homo sapiens GN=FAM126A PE=1 SV=2 - [HYCCI_HUMAN] |
| FBL | rRNA 2'-O-methyltransferase fibrillarin OS=Homo sapiens GN=FBL PE=1 SV=2 - [FBRL_HUMAN] |
| FLII | Protein flightless-1 homolog OS=Homo sapiens GN=FLII PE=1 SV=2 - [FLII_HUMAN] |
| FLNA | Filamin-A OS=Homo sapiens GN=FLNA PE=1 SV=4 - [FLNA_HUMAN] |
| FLNB | Filamin-B OS=Homo sapiens GN=FLNB PE=1 SV=2 - [FLNB_HUMAN] |
| FLOT1 | Flotillin-1 OS=Homo sapiens GN=FLOT1 PE=1 SV=3 - [FLOT1_HUMAN] |
| FLOT2 | Flotillin-2 OS=Homo sapiens GN=FLOT2 PE=1 SV=2 - [FLOT2_HUMAN] |
| FMNL2 | Formin-like protein 2 OS=Homo sapiens GN=FMNL2 PE=1 SV=3 - [FMNL2_HUMAN] |
| FMR1 | Fragile X mental retardation protein 1 OS=Homo sapiens GN=FMR1 PE=1 SV=1 - [FMR1_HUMAN] |
| FTH1 | Ferritin heavy chain OS=Homo sapiens GN=FTH1 PE=1 SV=2 - [FRIH_HUMAN] |
| FUS | RNA-binding protein FUS OS=Homo sapiens GN=FUS PE=1 SV=1 - [FUS_HUMAN] |
| FXR1 | Fragile X mental retardation syndrome-related protein 1 OS=Homo sapiens GN=FXR1 PE=1 SV=3 - [FXR1_HUMAN] |
| FXR2 | Fragile X mental retardation syndrome-related protein 2 OS=Homo sapiens GN=FXR2 PE=1 SV=2 - [FXR2_HUMAN] |
| G3BP1 | Ras GTPase-activating protein-binding protein 1 OS=Homo sapiens GN=G3BP1 PE=1 SV=1 - [G3BP1_HUMAN] |
| G3BP2 | Ras GTPase-activating protein-binding protein 2 OS=Homo sapiens GN=G3BP2 PE=1 SV=2 - [G3BP2_HUMAN] |
| GAR1 | H/ACA ribonucleoprotein complex subunit 1 OS=Homo sapiens GN=GAR1 PE=1 SV=1 - [GAR1_HUMAN] |
| GART | Trifunctional purine biosynthetic protein adenosine-3 OS=Homo sapiens GN=GART PE=1 SV=1 - [PUR2_HUMAN] |
| GIPC1 | PDZ domain-containing protein GIPC1 OS=Homo sapiens GN=GIPC1 PE=1 SV=2 - [GIPC1_HUMAN] |
| GJC1 | Gap junction gamma-1 protein OS=Homo sapiens GN=GJC1 PE=1 SV=2 - [CXG1_HUMAN] |
| GLB1 | Beta-galactosidase OS=Homo sapiens GN=GLB1 PE=1 SV=2 - [BGAL_HUMAN] |
| GNA11 | Guanine nucleotide-binding protein subunit alpha-11 OS=Homo sapiens GN=GNA11 PE=1 SV=2 - [GNA11_HUMAN] |
| GNAI2 | Guanine nucleotide-binding protein G(i) subunit alpha-2 OS=Homo sapiens GN=GNAI2 PE=1 SV=3 - [GNAI2_HUMAN] |
| GNAI3 | Guanine nucleotide-binding protein G(k) subunit alpha OS=Homo sapiens GN=GNAI3 PE=1 SV=3 - [GNAI3_HUMAN] |
| GNAL | Guanine nucleotide-binding protein G(olf) subunit alpha OS=Homo sapiens GN=GNAL PE=1 SV=1 - [GNAL_HUMAN] |
| GNAS | Guanine nucleotide-binding protein G(s) subunit alpha isoforms short OS=Homo sapiens GN=GNAS PE=1 SV=1 - [GNAS2_HUMAN] |
| GNB1 | Guanine nucleotide-binding protein G(I)/G(S)/G(T) subunit beta-1 OS=Homo sapiens GN=GNB1 PE=1 SV=3 - [GBB1_HUMAN] |
| GNB2 | Guanine nucleotide-binding protein G(I)/G(S)/G(T) subunit beta-2 OS=Homo sapiens GN=GNB2 PE=1 SV=3 - [GBB2_HUMAN] |
| GNG12 | Guanine nucleotide-binding protein G(I)/G(S)/G(O) subunit gamma-12 OS=Homo sapiens GN=GNG12 PE=1 SV=3 - [GBG12_HUMAN] |
| GNL3 | Guanine nucleotide-binding protein-like 3 OS=Homo sapiens GN=GNL3 PE=1 SV=2 - [GNL3_HUMAN] |
| GPC1 | Glypican-1 OS=Homo sapiens GN=GPC1 PE=1 SV=2 - [GPC1_HUMAN] |
| GPRC5A | Retinoic acid-induced protein 3 OS=Homo sapiens GN=GPRC5A PE=1 SV=2 - [RAI3_HUMAN] |
| GRWD1 | Glutamate-rich WD repeat-containing protein 1 OS=Homo sapiens GN=GRWD1 PE=1 SV=1 - [GRWD1_HUMAN] |
| GSN | Gelsolin OS=Homo sapiens GN=GSN PE=1 SV=1 - [GELS_HUMAN] |
| GTF2H1 | General transcription factor IIH subunit 1 OS=Homo sapiens GN=GTF2H1 PE=1 SV=1 - [TF2H1_HUMAN] |
| GTF2H2 | General transcription factor IIH subunit 2 OS=Homo sapiens GN=GTF2H2 PE=1 SV=1 - [TF2H2_HUMAN] |
| GTF2H3 | General transcription factor IIH subunit 3 OS=Homo sapiens GN=GTF2H3 PE=1 SV=2 - [TF2H3_HUMAN] |
| GTF2H4 | General transcription factor IIH subunit 4 OS=Homo sapiens GN=GTF2H4 PE=2 SV=1 - [TF2H4_HUMAN] |
| GTF2I | General transcription factor II-I OS=Homo sapiens GN=GTF2I PE=1 SV=2 - [GTF2I_HUMAN] |
| GTPBP4 | Nucleolar GTP-binding protein 1 OS=Homo sapiens GN=GTPBP4 PE=1 SV=3 - [NOG1_HUMAN] |
| H1F0 | Histone H1.0 OS=Homo sapiens GN=H1F0 PE=1 SV=3 - [H10_HUMAN] |
| H2AFX | Histone H2AX OS=Homo sapiens GN=H2AFX PE=1 SV=2 - [H2AX_HUMAN] |
| H2AFY | Core histone macro-H2A.1 OS=Homo sapiens GN=H2AFY PE=1 SV=4 - [H2AY_HUMAN] |
| H2AFZ | Histone H2A.Z OS=Homo sapiens GN=H2AFZ PE=1 SV=2 - [H2AZ_HUMAN] |
| HADHA | Trifunctional enzyme subunit alpha, mitochondrial OS=Homo sapiens GN=HADHA PE=1 SV=2 - [ECHA_HUMAN] |
| HADHB | Trifunctional enzyme subunit beta, mitochondrial OS=Homo sapiens GN=HADHB PE=1 SV=3 - [ECHB_HUMAN] |
| HCFC1 | Host cell factor 1 OS=Homo sapiens GN=HCFC1 PE=1 SV=2 - [HCFC1_HUMAN] |
| HDAC1 | Histone deacetylase 1 OS=Homo sapiens GN=HDAC1 PE=1 SV=1 - [HDAC1_HUMAN] |
| HDAC2 | Histone deacetylase 2 OS=Homo sapiens GN=HDAC2 PE=1 SV=2 - [HDAC2_HUMAN] |
| HDGFL2 | Hepatoma-derived growth factor-related protein 2 OS=Homo sapiens GN=HDGFRP2 PE=1 SV=1 - [HDGR2_HUMAN] |
| HEATR1 | HEAT repeat-containing protein 1 OS=Homo sapiens GN=HEATR1 PE=1 SV=3 - [HEAT1_HUMAN] |
| HIP1 | Huntingtin-interacting protein 1 OS=Homo sapiens GN=HIP1 PE=1 SV=5 - [HIP1_HUMAN] |
| HIST1H1B | Histone H1.5 OS=Homo sapiens GN=HIST1H1B PE=1 SV=3 - [H15_HUMAN] |
| HIST1H1C | Histone H1.2 OS=Homo sapiens GN=HIST1H1C PE=1 SV=2 - [H12_HUMAN] |
| HIST1H1D | Histone H1.3 OS=Homo sapiens GN=HIST1H1D PE=1 SV=2 - [H13_HUMAN] |
| HIST1H1E | Histone H1.4 OS=Homo sapiens GN=HIST1H1E PE=1 SV=2 - [H14_HUMAN] |
| HIST1H2AC | Histone H2A type 1-C OS=Homo sapiens GN=HIST1H2AC PE=1 SV=3 - [H2A1C_HUMAN] |
| HIST1H2AH | Histone H2A type 1-H OS=Homo sapiens GN=HIST1H2AH PE=1 SV=3 - [H2A1H_HUMAN] |
| HIST1H2BJ | Histone H2B type 1-J OS=Homo sapiens GN=HIST1H2BJ PE=1 SV=3 - [H2B1J_HUMAN] |
| HIST1H2BK | Histone H2B type 1-K OS=Homo sapiens GN=HIST1H2BK PE=1 SV=3 - [H2B1K_HUMAN] |
| HIST1H3A | Histone H3.1 OS=Homo sapiens GN=HIST1H3A PE=1 SV=2 - [H31_HUMAN] |
| HIST1H4A | Histone H4 OS=Homo sapiens GN=HIST1H4A PE=1 SV=2 - [H4_HUMAN] |
| HNRNPA0 | Heterogeneous nuclear ribonucleoprotein A0 OS=Homo sapiens GN=HNRNPA0 PE=1 SV=1 - [ROA0_HUMAN] |
| HNRNPA1 | Heterogeneous nuclear ribonucleoprotein A1 OS=Homo sapiens GN=HNRNPA1 PE=1 SV=5 - [ROA1_HUMAN] |
| HNRNPA2B1 | Heterogeneous nuclear ribonucleoproteins A2/B1 OS=Homo sapiens GN=HNRNPA2B1 PE=1 SV=2 - [ROA2_HUMAN] |
| HNRNPA3 | Heterogeneous nuclear ribonucleoprotein A3 OS=Homo sapiens GN=HNRNPA3 PE=1 SV=2 - [ROA3_HUMAN] |
| HNRNPAB | Heterogeneous nuclear ribonucleoprotein A/B OS=Homo sapiens GN=HNRNPAB PE=1 SV=2 - [ROAA_HUMAN] |
| HNRNPC | Heterogeneous nuclear ribonucleoproteins C1/C2 OS=Homo sapiens GN=HNRNPC PE=1 SV=4 - [HNRPC_HUMAN] |
| HNRNPD | Heterogeneous nuclear ribonucleoprotein D0 OS=Homo sapiens GN=HNRNPD PE=1 SV=1 - [HNRPD_HUMAN] |
| HNRNPDL | Heterogeneous nuclear ribonucleoprotein D-like OS=Homo sapiens GN=HNRNPDL PE=1 SV=3 - [HNRDL_HUMAN] |
| HNRNPF | Heterogeneous nuclear ribonucleoprotein F OS=Homo sapiens GN=HNRNPF PE=1 SV=3 - [HNRPF_HUMAN] |
| HNRNPH1 | Heterogeneous nuclear ribonucleoprotein H OS=Homo sapiens GN=HNRNPH1 PE=1 SV=4 - [HNRH1_HUMAN] |
| HNRNPH2 | Heterogeneous nuclear ribonucleoprotein H2 OS=Homo sapiens GN=HNRNPH2 PE=1 SV=1 - [HNRH2_HUMAN] |
| HNRNPH3 | Heterogeneous nuclear ribonucleoprotein H3 OS=Homo sapiens GN=HNRNPH3 PE=1 SV=2 - [HNRH3_HUMAN] |
| HNRNPK | Heterogeneous nuclear ribonucleoprotein K OS=Homo sapiens GN=HNRNPK PE=1 SV=1 - [HNRPK_HUMAN] |
| HNRNPL | Heterogeneous nuclear ribonucleoprotein L OS=Homo sapiens GN=HNRNPL PE=1 SV=2 - [HNRPL_HUMAN] |
| HNRNPLL | Heterogeneous nuclear ribonucleoprotein L-like OS=Homo sapiens GN=HNRNPLL PE=1 SV=1 - [HNRLL_HUMAN] |
| HNRNPM | Heterogeneous nuclear ribonucleoprotein M OS=Homo sapiens GN=HNRNPM PE=1 SV=3 - [HNRPM_HUMAN] |
| HNRNPR | Heterogeneous nuclear ribonucleoprotein R OS=Homo sapiens GN=HNRNPR PE=1 SV=1 - [HNRPR_HUMAN] |
| HNRNPU | Heterogeneous nuclear ribonucleoprotein U OS=Homo sapiens GN=HNRNPU PE=1 SV=6 - [HNRPU_HUMAN] |
| HNRNPUL1 | Heterogeneous nuclear ribonucleoprotein U-like protein 1 OS=Homo sapiens GN=HNRNPUL1 PE=1 SV=2 - [HNRL1_HUMAN] |
| HNRNPUL2 | Heterogeneous nuclear ribonucleoprotein U-like protein 2 OS=Homo sapiens GN=HNRNPUL2 PE=1 SV=1 - [HNRL2_HUMAN] |
| HP1BP3 | Heterochromatin protein 1-binding protein 3 OS=Homo sapiens GN=HP1BP3 PE=1 SV=1 - [HP1B3_HUMAN] |
| HSP90AA1 | Heat shock protein HSP 90-alpha OS=Homo sapiens GN=HSP90AA1 PE=1 SV=5 - [HS90A_HUMAN] |
| HSP90AB1 | Heat shock protein HSP 90-beta OS=Homo sapiens GN=HSP90AB1 PE=1 SV=4 - [HS90B_HUMAN] |
| HSPA4 | Heat shock 70 kDa protein 4 OS=Homo sapiens GN=HSPA4 PE=1 SV=4 - [HSP74_HUMAN] |
| HSPB1 | Heat shock protein beta-1 OS=Homo sapiens GN=HSPB1 PE=1 SV=2 - [HSPB1_HUMAN] |
| HSPD1 | 60 kDa heat shock protein, mitochondrial OS=Homo sapiens GN=HSPD1 PE=1 SV=2 - [CH60_HUMAN] |
| IGF2BP3 | Insulin-like growth factor 2 mRNA-binding protein 3 OS=Homo sapiens GN=IGF2BP3 PE=1 SV=2 - [IF2B3_HUMAN] |
| IGHG2 | Ig gamma-2 chain C region OS=Homo sapiens GN=IGHG2 PE=1 SV=2 - [IGHG2_HUMAN] |
| IK | Protein Red OS=Homo sapiens GN=IK PE=1 SV=3 - [RED_HUMAN] |
| ILF2 | Interleukin enhancer-binding factor 2 OS=Homo sapiens GN=ILF2 PE=1 SV=2 - [ILF2_HUMAN] |
| ILF3 | Interleukin enhancer-binding factor 3 OS=Homo sapiens GN=ILF3 PE=1 SV=3 - [ILF3_HUMAN] |
| IMMT | Mitochondrial inner membrane protein OS=Homo sapiens GN=IMMT PE=1 SV=1 - [IMMT_HUMAN] |
| IMPDH2 | Inosine-5'-monophosphate dehydrogenase 2 OS=Homo sapiens GN=IMPDH2 PE=1 SV=2 - [IMDH2_HUMAN] |
| INF2 | Inverted formin-2 OS=Homo sapiens GN=INF2 PE=1 SV=2 - [INF2_HUMAN] |
| IPO7 | Importin-7 OS=Homo sapiens GN=IPO7 PE=1 SV=1 - [IPO7_HUMAN] |
| IQGAP1 | Ras GTPase-activating-like protein IQGAP1 OS=Homo sapiens GN=IQGAP1 PE=1 SV=1 - [IQGA1_HUMAN] |
| IST1 | IST1 homolog OS=Homo sapiens GN=IST1 PE=1 SV=1 - [IST1_HUMAN] |
| ITPR3 | Inositol 1,4,5-trisphosphate receptor type 3 OS=Homo sapiens GN=ITPR3 PE=1 SV=2 - [ITPR3_HUMAN] |
| IWS1 | Protein IWS1 homolog OS=Homo sapiens GN=IWS1 PE=1 SV=2 - [IWS1_HUMAN] |
| JADE1 | Protein Jade-1 OS=Homo sapiens GN=JADE1 PE=1 SV=1 - [JADE1_HUMAN] |
| KAT7 | Histone acetyltransferase KAT7 OS=Homo sapiens GN=KAT7 PE=1 SV=1 - [KAT7_HUMAN] |
| KDM1A | Lysine-specific histone demethylase 1A OS=Homo sapiens GN=KDM1A PE=1 SV=2 - [KDM1A_HUMAN] |
| KDM2A | Lysine-specific demethylase 2A OS=Homo sapiens GN=KDM2A PE=1 SV=3 - [KDM2A_HUMAN] |
| KEAP1 | Kelch-like ECH-associated protein 1 OS=Homo sapiens GN=KEAP1 PE=1 SV=2 - [KEAP1_HUMAN] |
| KHDRBS1 | KH domain-containing, RNA-binding, signal transduction-associated protein 1 OS=Homo sapiens GN=KHDRBS1 PE=1 SV=1 - [KHDR1_HUMAN] |
| KIAA1671 | Uncharacterized protein KIAA1671 OS=Homo sapiens GN=KIAA1671 PE=1 SV=2 - [K1671_HUMAN] |
| KIF21B | Kinesin-like protein KIF21B OS=Homo sapiens GN=KIF21B PE=1 SV=2 - [KI21B_HUMAN] |
| KIF22 | Kinesin-like protein KIF22 OS=Homo sapiens GN=KIF22 PE=1 SV=5 - [KIF22_HUMAN] |
| KIF23 | Kinesin-like protein KIF23 OS=Homo sapiens GN=KIF23 PE=1 SV=3 - [KIF23_HUMAN] |
| KIF2A | Kinesin-like protein KIF2A OS=Homo sapiens GN=KIF2A PE=1 SV=3 - [KIF2A_HUMAN] |
| KIF5C | Kinesin heavy chain isoform 5C OS=Homo sapiens GN=KIF5C PE=1 SV=1 - [KIF5C_HUMAN] |
| KPNA1 | Importin subunit alpha-5 OS=Homo sapiens GN=KPNA1 PE=1 SV=3 - [IMA5_HUMAN] |
| KPNA2 | Importin subunit alpha-1 OS=Homo sapiens GN=KPNA2 PE=1 SV=1 - [IMA1_HUMAN] |
| KPNA3 | Importin subunit alpha-4 OS=Homo sapiens GN=KPNA3 PE=1 SV=2 - [IMA4_HUMAN] |
| KPNA4 | Importin subunit alpha-3 OS=Homo sapiens GN=KPNA4 PE=1 SV=1 - [IMA3_HUMAN] |
| KPNA6 | Importin subunit alpha-7 OS=Homo sapiens GN=KPNA6 PE=1 SV=1 - [IMA7_HUMAN] |
| KPNB1 | Importin subunit beta-1 OS=Homo sapiens GN=KPNB1 PE=1 SV=2 - [IMB1_HUMAN] |
| KRI1 | Protein KRI1 homolog OS=Homo sapiens GN=KRI1 PE=1 SV=3 - [KRI1_HUMAN] |
| KRR1 | KRR1 small subunit processome component homolog OS=Homo sapiens GN=KRR1 PE=1 SV=4 - [KRR1_HUMAN] |
| KRT12 | Keratin, type I cytoskeletal 12 OS=Homo sapiens GN=KRT12 PE=1 SV=1 - [K1C12_HUMAN] |
| KRT15 | Keratin, type I cytoskeletal 15 OS=Homo sapiens GN=KRT15 PE=1 SV=3 - [K1C15_HUMAN] |
| KRT17 | Keratin, type I cytoskeletal 17 OS=Homo sapiens GN=KRT17 PE=1 SV=2 - [K1C17_HUMAN] |
| KRT18 | Keratin, type I cytoskeletal 18 OS=Homo sapiens GN=KRT18 PE=1 SV=2 - [K1C18_HUMAN] |
| KRT19 | Keratin, type I cytoskeletal 19 OS=Homo sapiens GN=KRT19 PE=1 SV=4 - [K1C19_HUMAN] |
| KRT4 | Keratin, type II cytoskeletal 4 OS=Homo sapiens GN=KRT4 PE=1 SV=4 - [K2C4_HUMAN] |
| KRT6A | Keratin, type II cytoskeletal 6A OS=Homo sapiens GN=KRT6A PE=1 SV=3 - [K2C6A_HUMAN] |
| KRT6B | Keratin, type II cytoskeletal 6B OS=Homo sapiens GN=KRT6B PE=1 SV=5 - [K2C6B_HUMAN] |
| KRT8 | Keratin, type II cytoskeletal 8 OS=Homo sapiens GN=KRT8 PE=1 SV=7 - [K2C8_HUMAN] |
| KRT80 | Keratin, type II cytoskeletal 80 OS=Homo sapiens GN=KRT80 PE=1 SV=2 - [K2C80_HUMAN] |
| KRT86 | Keratin, type II cuticular Hb6 OS=Homo sapiens GN=KRT86 PE=1 SV=1 - [KRT86_HUMAN] |
| LARS | Leucine--tRNA ligase, cytoplasmic OS=Homo sapiens GN=LARS PE=1 SV=2 - [SYLC_HUMAN] |
| LASP1 | LIM and SH3 domain protein 1 OS=Homo sapiens GN=LASP1 PE=1 SV=2 - [LASP1_HUMAN] |
| LEO1 | RNA polymerase-associated protein LEO1 OS=Homo sapiens GN=LEO1 PE=1 SV=1 - [LEO1_HUMAN] |
| LGALS8 | Galectin-8 OS=Homo sapiens GN=LGALS8 PE=1 SV=4 - [LEG8_HUMAN] |
| LIG3 | DNA ligase 3 OS=Homo sapiens GN=LIG3 PE=1 SV=2 - [DNLI3_HUMAN] |
| LIMA1 | LIM domain and actin-binding protein 1 OS=Homo sapiens GN=LIMA1 PE=1 SV=1 - [LIMA1_HUMAN] |
| LMNA | Prelamin-A/C OS=Homo sapiens GN=LMNA PE=1 SV=1 - [LMNA_HUMAN] |
| LMNB1 | Lamin-B1 OS=Homo sapiens GN=LMNB1 PE=1 SV=2 - [LMNB1_HUMAN] |
| LMNB2 | Lamin-B2 OS=Homo sapiens GN=LMNB2 PE=1 SV=4 - [LMNB2_HUMAN] |
| LONP1 | Lon protease homolog, mitochondrial OS=Homo sapiens GN=LONP1 PE=1 SV=2 - [LONM_HUMAN] |
| LRCH3 | Leucine-rich repeat and calponin homology domain-containing protein 3 OS=Homo sapiens GN=LRCH3 PE=1 SV=2 - [LRCH3_HUMAN] |
| LRWD1 | Leucine-rich repeat and WD repeat-containing protein 1 OS=Homo sapiens GN=LRWD1 PE=1 SV=2 - [LRWD1_HUMAN] |
| LSM12 | Protein LSM12 homolog OS=Homo sapiens GN=LSM12 PE=1 SV=2 - [LSM12_HUMAN] |
| LSM2 | U6 snRNA-associated Sm-like protein LSm2 OS=Homo sapiens GN=LSM2 PE=1 SV=1 - [LSM2_HUMAN] |
| LSM3 | U6 snRNA-associated Sm-like protein LSm3 OS=Homo sapiens GN=LSM3 PE=1 SV=2 - [LSM3_HUMAN] |
| LSM6 | U6 snRNA-associated Sm-like protein LSm6 OS=Homo sapiens GN=LSM6 PE=1 SV=1 - [LSM6_HUMAN] |
| LSM7 | U6 snRNA-associated Sm-like protein LSm7 OS=Homo sapiens GN=LSM7 PE=1 SV=1 - [LSM7_HUMAN] |
| LSM8 | U6 snRNA-associated Sm-like protein LSm8 OS=Homo sapiens GN=LSM8 PE=1 SV=3 - [LSM8_HUMAN] |
| LUC7L2 | Putative RNA-binding protein Luc7-like 2 OS=Homo sapiens GN=LUC7L2 PE=1 SV=2 - [LC7L2_HUMAN] |
| LUC7L3 | Luc7-like protein 3 OS=Homo sapiens GN=LUC7L3 PE=1 SV=2 - [LC7L3_HUMAN] |
| LUZP1 | Leucine zipper protein 1 OS=Homo sapiens GN=LUZP1 PE=1 SV=2 - [LUZP1_HUMAN] |
| LYAR | Cell growth-regulating nucleolar protein OS=Homo sapiens GN=LYAR PE=1 SV=2 - [LYAR_HUMAN] |
| LYN | Tyrosine-protein kinase Lyn OS=Homo sapiens GN=LYN PE=1 SV=3 - [LYN_HUMAN] |
| MACF1 | Microtubule-actin cross-linking factor 1, isoforms 1/2/3/5 OS=Homo sapiens GN=MACF1 PE=1 SV=4 - [MACF1_HUMAN] |
| MAGOH | Protein mago nashi homolog OS=Homo sapiens GN=MAGOH PE=1 SV=1 - [MGN_HUMAN] |
| MAGOHB | Protein mago nashi homolog 2 OS=Homo sapiens GN=MAGOHB PE=1 SV=1 - [MGN2_HUMAN] |
| MAK16 | Protein MAK16 homolog OS=Homo sapiens GN=MAK16 PE=1 SV=2 - [MAK16_HUMAN] |
| MAT2A | S-adenosylmethionine synthase isoform type-2 OS=Homo sapiens GN=MAT2A PE=1 SV=1 - [METK2_HUMAN] |
| MATR3 | Matrin-3 OS=Homo sapiens GN=MATR3 PE=1 SV=2 - [MATR3_HUMAN] |
| MCM3 | DNA replication licensing factor MCM3 OS=Homo sapiens GN=MCM3 PE=1 SV=3 - [MCM3_HUMAN] |
| MCM3AP | Germinal-center associated nuclear protein OS=Homo sapiens GN=MCM3AP PE=1 SV=2 - [GANP_HUMAN] |
| MCM4 | DNA replication licensing factor MCM4 OS=Homo sapiens GN=MCM4 PE=1 SV=5 - [MCM4_HUMAN] |
| MCM5 | DNA replication licensing factor MCM5 OS=Homo sapiens GN=MCM5 PE=1 SV=5 - [MCM5_HUMAN] |
| MCM6 | DNA replication licensing factor MCM6 OS=Homo sapiens GN=MCM6 PE=1 SV=1 - [MCM6_HUMAN] |
| MCM7 | DNA replication licensing factor MCM7 OS=Homo sapiens GN=MCM7 PE=1 SV=4 - [MCM7_HUMAN] |
| MDC1 | Mediator of DNA damage checkpoint protein 1 OS=Homo sapiens GN=MDC1 PE=1 SV=3 - [MDC1_HUMAN] |
| MELTF | Melanotransferrin OS=Homo sapiens GN=MFI2 PE=1 SV=2 - [TRFM_HUMAN] |
| MEN1 | Menin OS=Homo sapiens GN=MEN1 PE=1 SV=4 - [MEN1_HUMAN] |
| METAP1 | Methionine aminopeptidase 1 OS=Homo sapiens GN=METAP1 PE=1 SV=2 - [MAP11_HUMAN] |
| MFAP1 | Microfibrillar-associated protein 1 OS=Homo sapiens GN=MFAP1 PE=1 SV=2 - [MFAP1_HUMAN] |
| MFGE8 | Lactadherin OS=Homo sapiens GN=MFGE8 PE=1 SV=2 - [MFGM_HUMAN] |
| MISP | Mitotic interactor and substrate of PLK1 OS=Homo sapiens GN=MISP PE=1 SV=1 - [MISP_HUMAN] |
| MITD1 | MIT domain-containing protein 1 OS=Homo sapiens GN=MITD1 PE=1 SV=1 - [MITD1_HUMAN] |
| MKI67 | Antigen KI-67 OS=Homo sapiens GN=MKI67 PE=1 SV=2 - [KI67_HUMAN] |
| MPRIP | Myosin phosphatase Rho-interacting protein OS=Homo sapiens GN=MPRIP PE=1 SV=3 - [MPRIP_HUMAN] |
| MRM3 | RNA methyltransferase-like protein 1 OS=Homo sapiens GN=RNMTL1 PE=1 SV=2 - [RMTL1_HUMAN] |
| MRTO4 | mRNA turnover protein 4 homolog OS=Homo sapiens GN=MRTO4 PE=1 SV=2 - [MRT4_HUMAN] |
| MSH2 | DNA mismatch repair protein Msh2 OS=Homo sapiens GN=MSH2 PE=1 SV=1 - [MSH2_HUMAN] |
| MSH3 | DNA mismatch repair protein Msh3 OS=Homo sapiens GN=MSH3 PE=1 SV=4 - [MSH3_HUMAN] |
| MSN | Moesin OS=Homo sapiens GN=MSN PE=1 SV=3 - [MOES_HUMAN] |
| MTREX | Superkiller viralicidic activity 2-like 2 OS=Homo sapiens GN=SKIV2L2 PE=1 SV=3 - [SK2L2_HUMAN] |
| MYADM | Myeloid-associated differentiation marker OS=Homo sapiens GN=MYADM PE=1 SV=2 - [MYADM_HUMAN] |
| MYBBP1A | Myb-binding protein 1A OS=Homo sapiens GN=MYBBP1A PE=1 SV=2 - [MBB1A_HUMAN] |
| MYH10 | Myosin-10 OS=Homo sapiens GN=MYH10 PE=1 SV=3 - [MYH10_HUMAN] |
| MYH9 | Myosin-9 OS=Homo sapiens GN=MYH9 PE=1 SV=4 - [MYH9_HUMAN] |
| MYL12A | Myosin regulatory light chain 12A OS=Homo sapiens GN=MYL12A PE=1 SV=2 - [ML12A_HUMAN] |
| MYL6 | Myosin light polypeptide 6 OS=Homo sapiens GN=MYL6 PE=1 SV=2 - [MYL6_HUMAN] |
| MYO18A | Unconventional myosin-XVIIIa OS=Homo sapiens GN=MYO18A PE=1 SV=3 - [MY18A_HUMAN] |
| MYO1C | Unconventional myosin-Ic OS=Homo sapiens GN=MYO1C PE=1 SV=4 - [MYO1C_HUMAN] |
| MYO5A | Unconventional myosin-Va OS=Homo sapiens GN=MYO5A PE=1 SV=2 - [MYO5A_HUMAN] |
| MYO6 | Unconventional myosin-VI OS=Homo sapiens GN=MYO6 PE=1 SV=4 - [MYO6_HUMAN] |
| MYOF | Myoferlin OS=Homo sapiens GN=MYOF PE=1 SV=1 - [MYOF_HUMAN] |
| NACA | Nascent polypeptide-associated complex subunit alpha OS=Homo sapiens GN=NACA PE=1 SV=1 - [NACA_HUMAN] |
| NAT10 | N-acetyltransferase 10 OS=Homo sapiens GN=NAT10 PE=1 SV=2 - [NAT10_HUMAN] |
| NCBP1 | Nuclear cap-binding protein subunit 1 OS=Homo sapiens GN=NCBP1 PE=1 SV=1 - [NCBP1_HUMAN] |
| NCBP2 | Nuclear cap-binding protein subunit 2 OS=Homo sapiens GN=NCBP2 PE=1 SV=1 - [NCBP2_HUMAN] |
| NCBP3 | Uncharacterized protein C17orf85 OS=Homo sapiens GN=C17orf85 PE=1 SV=2 - [CQ085_HUMAN] |
| NES | Nestin OS=Homo sapiens GN=NES PE=1 SV=2 - [NEST_HUMAN] |
| NEXN | Nexilin OS=Homo sapiens GN=NEXN PE=1 SV=1 - [NEXN_HUMAN] |
| NF2 | Merlin OS=Homo sapiens GN=NF2 PE=1 SV=1 - [MERL_HUMAN] |
| NFYC | Nuclear transcription factor Y subunit gamma OS=Homo sapiens GN=NFYC PE=1 SV=3 - [NFYC_HUMAN] |
| NHEJ1 | Non-homologous end-joining factor 1 OS=Homo sapiens GN=NHEJ1 PE=1 SV=1 - [NHEJ1_HUMAN] |
| NHP2 | H/ACA ribonucleoprotein complex subunit 2 OS=Homo sapiens GN=NHP2 PE=1 SV=1 - [NHP2_HUMAN] |
| NIP7 | 60S ribosome subunit biogenesis protein NIP7 homolog OS=Homo sapiens GN=NIP7 PE=1 SV=1 - [NIP7_HUMAN] |
| NKRF | NF-kappa-B-repressing factor OS=Homo sapiens GN=NKRF PE=1 SV=2 - [NKRF_HUMAN] |
| NLE1 | Notchless protein homolog 1 OS=Homo sapiens GN=NLE1 PE=1 SV=4 - [NLE1_HUMAN] |
| NMNAT1 | Nicotinamide mononucleotide adenylyltransferase 1 OS=Homo sapiens GN=NMNAT1 PE=1 SV=1 - [NMNA1_HUMAN] |
| NOC2L | Nucleolar complex protein 2 homolog OS=Homo sapiens GN=NOC2L PE=1 SV=4 - [NOC2L_HUMAN] |
| NOL11 | Nucleolar protein 11 OS=Homo sapiens GN=NOL11 PE=1 SV=1 - [NOL11_HUMAN] |
| NOLC1 | Nucleolar and coiled-body phosphoprotein 1 OS=Homo sapiens GN=NOLC1 PE=1 SV=2 - [NOLC1_HUMAN] |
| NONO | Non-POU domain-containing octamer-binding protein OS=Homo sapiens GN=NONO PE=1 SV=4 - [NONO_HUMAN] |
| NOP10 | H/ACA ribonucleoprotein complex subunit 3 OS=Homo sapiens GN=NOP10 PE=1 SV=1 - [NOP10_HUMAN] |
| NOP2 | Putative ribosomal RNA methyltransferase NOP2 OS=Homo sapiens GN=NOP2 PE=1 SV=2 - [NOP2_HUMAN] |
| NOP56 | Nucleolar protein 56 OS=Homo sapiens GN=NOP56 PE=1 SV=4 - [NOP56_HUMAN] |
| NOP58 | Nucleolar protein 58 OS=Homo sapiens GN=NOP58 PE=1 SV=1 - [NOP58_HUMAN] |
| NOSIP | Nitric oxide synthase-interacting protein OS=Homo sapiens GN=NOSIP PE=1 SV=1 - [NOSIP_HUMAN] |
| NPM1 | Nucleophosmin OS=Homo sapiens GN=NPM1 PE=1 SV=2 - [NPM_HUMAN] |
| NPM3 | Nucleoplasmin-3 OS=Homo sapiens GN=NPM3 PE=1 SV=3 - [NPM3_HUMAN] |
| NQO1 | NAD(P)H dehydrogenase [quinone] 1 OS=Homo sapiens GN=NQO1 PE=1 SV=1 - [NQO1_HUMAN] |
| NSF | Vesicle-fusing ATPase OS=Homo sapiens GN=NSF PE=1 SV=3 - [NSF_HUMAN] |
| NSUN2 | tRNA (cytosine(34)-C(5))-methyltransferase OS=Homo sapiens GN=NSUN2 PE=1 SV=2 - [NSUN2_HUMAN] |
| NSUN5 | Putative methyltransferase NSUN5 OS=Homo sapiens GN=NSUN5 PE=1 SV=2 - [NSUN5_HUMAN] |
| NT5E | 5'-nucleotidase OS=Homo sapiens GN=NT5E PE=1 SV=1 - [5NTD_HUMAN] |
| NUDT21 | Cleavage and polyadenylation specificity factor subunit 5 OS=Homo sapiens GN=NUDT21 PE=1 SV=1 - [CPSF5_HUMAN] |
| NUFIP2 | Nuclear fragile X mental retardation-interacting protein 2 OS=Homo sapiens GN=NUFIP2 PE=1 SV=1 - [NUFP2_HUMAN] |
| NUMA1 | Nuclear mitotic apparatus protein 1 OS=Homo sapiens GN=NUMA1 PE=1 SV=2 - [NUMA1_HUMAN] |
| NUP107 | Nuclear pore complex protein Nup107 OS=Homo sapiens GN=NUP107 PE=1 SV=1 - [NU107_HUMAN] |
| NUP133 | Nuclear pore complex protein Nup133 OS=Homo sapiens GN=NUP133 PE=1 SV=2 - [NU133_HUMAN] |
| NUP153 | Nuclear pore complex protein Nup153 OS=Homo sapiens GN=NUP153 PE=1 SV=2 - [NU153_HUMAN] |
| NUP155 | Nuclear pore complex protein Nup155 OS=Homo sapiens GN=NUP155 PE=1 SV=1 - [NU155_HUMAN] |
| NUP160 | Nuclear pore complex protein Nup160 OS=Homo sapiens GN=NUP160 PE=1 SV=3 - [NU160_HUMAN] |
| NUP188 | Nucleoporin NUP188 homolog OS=Homo sapiens GN=NUP188 PE=1 SV=1 - [NU188_HUMAN] |
| NUP205 | Nuclear pore complex protein Nup205 OS=Homo sapiens GN=NUP205 PE=1 SV=3 - [NU205_HUMAN] |
| NUP214 | Nuclear pore complex protein Nup214 OS=Homo sapiens GN=NUP214 PE=1 SV=2 - [NU214_HUMAN] |
| NUP35 | Nucleoporin NUP53 OS=Homo sapiens GN=NUP35 PE=1 SV=1 - [NUP53_HUMAN] |
| NUP37 | Nucleoporin Nup37 OS=Homo sapiens GN=NUP37 PE=1 SV=1 - [NUP37_HUMAN] |
| NUP43 | Nucleoporin Nup43 OS=Homo sapiens GN=NUP43 PE=1 SV=1 - [NUP43_HUMAN] |
| NUP50 | Nuclear pore complex protein Nup50 OS=Homo sapiens GN=NUP50 PE=1 SV=2 - [NUP50_HUMAN] |
| NUP54 | Nucleoporin p54 OS=Homo sapiens GN=NUP54 PE=1 SV=2 - [NUP54_HUMAN] |
| NUP58 | Nucleoporin p58/p45 OS=Homo sapiens GN=NUPL1 PE=1 SV=1 - [NUPL1_HUMAN] |
| NUP62 | Nuclear pore glycoprotein p62 OS=Homo sapiens GN=NUP62 PE=1 SV=3 - [NUP62_HUMAN] |
| NUP85 | Nuclear pore complex protein Nup85 OS=Homo sapiens GN=NUP85 PE=1 SV=1 - [NUP85_HUMAN] |
| NUP88 | Nuclear pore complex protein Nup88 OS=Homo sapiens GN=NUP88 PE=1 SV=2 - [NUP88_HUMAN] |
| NUP93 | Nuclear pore complex protein Nup93 OS=Homo sapiens GN=NUP93 PE=1 SV=2 - [NUP93_HUMAN] |
| NUP98 | Nuclear pore complex protein Nup98-Nup96 OS=Homo sapiens GN=NUP98 PE=1 SV=4 - [NUP98_HUMAN] |
| NVL | Nuclear valosin-containing protein-like OS=Homo sapiens GN=NVL PE=1 SV=1 - [NVL_HUMAN] |
| NXF1 | Nuclear RNA export factor 1 OS=Homo sapiens GN=NXF1 PE=1 SV=1 - [NXF1_HUMAN] |
| OGDH | 2-oxoglutarate dehydrogenase, mitochondrial OS=Homo sapiens GN=OGDH PE=1 SV=3 - [ODO1_HUMAN] |
| OGT | UDP-N-acetylglucosamine--peptide N-acetylglucosaminyltransferase 110 kDa subunit OS=Homo sapiens GN=OGT PE=1 SV=3 - [OGT1_HUMAN] |
| ORC3 | Origin recognition complex subunit 3 OS=Homo sapiens GN=ORC3 PE=1 SV=1 - [ORC3_HUMAN] |
| ORC5 | Origin recognition complex subunit 5 OS=Homo sapiens GN=ORC5 PE=1 SV=1 - [ORC5_HUMAN] |
| P3H1 | Prolyl 3-hydroxylase 1 OS=Homo sapiens GN=LEPRE1 PE=1 SV=2 - [P3H1_HUMAN] |
| P4HB | Protein disulfide-isomerase OS=Homo sapiens GN=P4HB PE=1 SV=3 - [PDIA1_HUMAN] |
| PA2G4 | Proliferation-associated protein 2G4 OS=Homo sapiens GN=PA2G4 PE=1 SV=3 - [PA2G4_HUMAN] |
| PABPC1 | Polyadenylate-binding protein 1 OS=Homo sapiens GN=PABPC1 PE=1 SV=2 - [PABP1_HUMAN] |
| PABPC4 | Polyadenylate-binding protein 4 OS=Homo sapiens GN=PABPC4 PE=1 SV=1 - [PABP4_HUMAN] |
| PABPN1 | Polyadenylate-binding protein 2 OS=Homo sapiens GN=PABPN1 PE=1 SV=3 - [PABP2_HUMAN] |
| PACSIN3 | Protein kinase C and casein kinase substrate in neurons protein 3 OS=Homo sapiens GN=PACSIN3 PE=1 SV=2 - [PACN3_HUMAN] |
| PAF1 | RNA polymerase II-associated factor 1 homolog OS=Homo sapiens GN=PAF1 PE=1 SV=2 - [PAF1_HUMAN] |
| PAIP1 | Polyadenylate-binding protein-interacting protein 1 OS=Homo sapiens GN=PAIP1 PE=1 SV=1 - [PAIP1_HUMAN] |
| PAK1IP1 | p21-activated protein kinase-interacting protein 1 OS=Homo sapiens GN=PAK1IP1 PE=1 SV=2 - [PK1IP_HUMAN] |
| PARP1 | Poly [ADP-ribose] polymerase 1 OS=Homo sapiens GN=PARP1 PE=1 SV=4 - [PARP1_HUMAN] |
| PCBP2 | Poly(rC)-binding protein 2 OS=Homo sapiens GN=PCBP2 PE=1 SV=1 - [PCBP2_HUMAN] |
| PCBP3 | Poly(rC)-binding protein 3 OS=Homo sapiens GN=PCBP3 PE=2 SV=2 - [PCBP3_HUMAN] |
| PCID2 | PCI domain-containing protein 2 OS=Homo sapiens GN=PCID2 PE=1 SV=2 - [PCID2_HUMAN] |
| PCNA | Proliferating cell nuclear antigen OS=Homo sapiens GN=PCNA PE=1 SV=1 - [PCNA_HUMAN] |
| PDCD11 | Protein RRP5 homolog OS=Homo sapiens GN=PDCD11 PE=1 SV=3 - [RRP5_HUMAN] |
| PDCD6 | Programmed cell death protein 6 OS=Homo sapiens GN=PDCD6 PE=1 SV=1 - [PDCD6_HUMAN] |
| PDCD6IP | Programmed cell death 6-interacting protein OS=Homo sapiens GN=PDCD6IP PE=1 SV=1 - [PDC6I_HUMAN] |
| PDS5B | Sister chromatid cohesion protein PDS5 homolog B OS=Homo sapiens GN=PDS5B PE=1 SV=1 - [PDS5B_HUMAN] |
| PES1 | Pescadillo homolog OS=Homo sapiens GN=PES1 PE=1 SV=1 - [PESC_HUMAN] |
| PFKFB3 | 6-phosphofructo-2-kinase/fructose-2,6-bisphosphatase 3 OS=Homo sapiens GN=PFKFB3 PE=1 SV=1 - [F263_HUMAN] |
| PFN1 | Profilin-1 OS=Homo sapiens GN=PFN1 PE=1 SV=2 - [PROF1_HUMAN] |
| PFN2 | Profilin-2 OS=Homo sapiens GN=PFN2 PE=1 SV=3 - [PROF2_HUMAN] |
| PGAM5 | Serine/threonine-protein phosphatase PGAM5, mitochondrial OS=Homo sapiens GN=PGAM5 PE=1 SV=2 - [PGAM5_HUMAN] |
| PHF5A | PHD finger-like domain-containing protein 5A OS=Homo sapiens GN=PHF5A PE=1 SV=1 - [PHF5A_HUMAN] |
| PHF6 | PHD finger protein 6 OS=Homo sapiens GN=PHF6 PE=1 SV=1 - [PHF6_HUMAN] |
| PI4KA | Phosphatidylinositol 4-kinase alpha OS=Homo sapiens GN=PI4KA PE=1 SV=4 - [PI4KA_HUMAN] |
| PICALM | Phosphatidylinositol-binding clathrin assembly protein OS=Homo sapiens GN=PICALM PE=1 SV=2 - [PICAL_HUMAN] |
| PIK3C2A | Phosphatidylinositol 4-phosphate 3-kinase C2 domain-containing subunit alpha OS=Homo sapiens GN=PIK3C2A PE=1 SV=2 - [P3C2A_HUMAN] |
| PLD2 | Phospholipase D2 OS=Homo sapiens GN=PLD2 PE=1 SV=2 - [PLD2_HUMAN] |
| PLEC | Plectin OS=Homo sapiens GN=PLEC PE=1 SV=3 - [PLEC_HUMAN] |
| PLOD1 | Procollagen-lysine,2-oxoglutarate 5-dioxygenase 1 OS=Homo sapiens GN=PLOD1 PE=1 SV=2 - [PLOD1_HUMAN] |
| PLOD3 | Procollagen-lysine,2-oxoglutarate 5-dioxygenase 3 OS=Homo sapiens GN=PLOD3 PE=1 SV=1 - [PLOD3_HUMAN] |
| PLRG1 | Pleiotropic regulator 1 OS=Homo sapiens GN=PLRG1 PE=1 SV=1 - [PLRG1_HUMAN] |
| PLS1 | Plastin-1 OS=Homo sapiens GN=PLS1 PE=1 SV=2 - [PLSI_HUMAN] |
| PLS3 | Plastin-3 OS=Homo sapiens GN=PLS3 PE=1 SV=4 - [PLST_HUMAN] |
| PML | Protein PML OS=Homo sapiens GN=PML PE=1 SV=3 - [PML_HUMAN] |
| PNN | Pinin OS=Homo sapiens GN=PNN PE=1 SV=4 - [PININ_HUMAN] |
| POLB | DNA polymerase beta OS=Homo sapiens GN=POLB PE=1 SV=3 - [DPOLB_HUMAN] |
| POLDIP3 | Polymerase delta-interacting protein 3 OS=Homo sapiens GN=POLDIP3 PE=1 SV=2 - [PDIP3_HUMAN] |
| POLR1A | DNA-directed RNA polymerase I subunit RPA1 OS=Homo sapiens GN=POLR1A PE=1 SV=2 - [RPA1_HUMAN] |
| POLR1B | DNA-directed RNA polymerase I subunit RPA2 OS=Homo sapiens GN=POLR1B PE=1 SV=2 - [RPA2_HUMAN] |
| POLR1C | DNA-directed RNA polymerases I and III subunit RPAC1 OS=Homo sapiens GN=POLR1C PE=1 SV=1 - [RPAC1_HUMAN] |
| POLR2E | DNA-directed RNA polymerases I, II, and III subunit RPABC1 OS=Homo sapiens GN=POLR2E PE=1 SV=4 - [RPAB1_HUMAN] |
| POLR2H | DNA-directed RNA polymerases I, II, and III subunit RPABC3 OS=Homo sapiens GN=POLR2H PE=1 SV=4 - [RPAB3_HUMAN] |
| POLR2K | DNA-directed RNA polymerases I, II, and III subunit RPABC4 OS=Homo sapiens GN=POLR2K PE=2 SV=1 - [RPAB4_HUMAN] |
| POLR2L | DNA-directed RNA polymerases I, II, and III subunit RPABC5 OS=Homo sapiens GN=POLR2L PE=1 SV=1 - [RPAB5_HUMAN] |
| POTEE | POTE ankyrin domain family member E OS=Homo sapiens GN=POTEE PE=1 SV=3 - [POTEE_HUMAN] |
| POTEJ | POTE ankyrin domain family member J OS=Homo sapiens GN=POTEJ PE=3 SV=1 - [POTEJ_HUMAN] |
| PPIA | Peptidyl-prolyl cis-trans isomerase A OS=Homo sapiens GN=PPIA PE=1 SV=2 - [PPIA_HUMAN] |
| PPIB | Peptidyl-prolyl cis-trans isomerase B OS=Homo sapiens GN=PPIB PE=1 SV=2 - [PPIB_HUMAN] |
| PPIG | Peptidyl-prolyl cis-trans isomerase G OS=Homo sapiens GN=PPIG PE=1 SV=2 - [PPIG_HUMAN] |
| PPIH | Peptidyl-prolyl cis-trans isomerase H OS=Homo sapiens GN=PPIH PE=1 SV=1 - [PPIH_HUMAN] |
| PPM1B | Protein phosphatase 1B OS=Homo sapiens GN=PPM1B PE=1 SV=1 - [PPM1B_HUMAN] |
| PPP1CA | Serine/threonine-protein phosphatase PP1-alpha catalytic subunit OS=Homo sapiens GN=PPP1CA PE=1 SV=1 - [PP1A_HUMAN] |
| PPP1CB | Serine/threonine-protein phosphatase PP1-beta catalytic subunit OS=Homo sapiens GN=PPP1CB PE=1 SV=3 - [PP1B_HUMAN] |
| PPP1CC | Serine/threonine-protein phosphatase PP1-gamma catalytic subunit OS=Homo sapiens GN=PPP1CC PE=1 SV=1 - [PP1G_HUMAN] |
| PPP1R12A | Protein phosphatase 1 regulatory subunit 12A OS=Homo sapiens GN=PPP1R12A PE=1 SV=1 - [MYPT1_HUMAN] |
| PPP1R18 | Phostensin OS=Homo sapiens GN=PPP1R18 PE=1 SV=1 - [PPR18_HUMAN] |
| PPP1R8 | Nuclear inhibitor of protein phosphatase 1 OS=Homo sapiens GN=PPP1R8 PE=1 SV=2 - [PP1R8_HUMAN] |
| PPP1R9B | Neurabin-2 OS=Homo sapiens GN=PPP1R9B PE=1 SV=2 - [NEB2_HUMAN] |
| PPP2R1A | Serine/threonine-protein phosphatase 2A 65 kDa regulatory subunit A alpha isoform OS=Homo sapiens GN=PPP2R1A PE=1 SV=4 - [2AAA_HUMAN] |
| PPP2R5C | Serine/threonine-protein phosphatase 2A 56 kDa regulatory subunit gamma isoform OS=Homo sapiens GN=PPP2R5C PE=1 SV=3 - [2A5G_HUMAN] |
| PPWD1 | Peptidylprolyl isomerase domain and WD repeat-containing protein 1 OS=Homo sapiens GN=PPWD1 PE=1 SV=1 - [PPWD1_HUMAN] |
| PRKDC | DNA-dependent protein kinase catalytic subunit OS=Homo sapiens GN=PRKDC PE=1 SV=3 - [PRKDC_HUMAN] |
| PRNP | Major prion protein OS=Homo sapiens GN=PRNP PE=1 SV=1 - [PRIO_HUMAN] |
| PRPF18 | Pre-mRNA-splicing factor 18 OS=Homo sapiens GN=PRPF18 PE=1 SV=1 - [PRP18_HUMAN] |
| PRPF19 | Pre-mRNA-processing factor 19 OS=Homo sapiens GN=PRPF19 PE=1 SV=1 - [PRP19_HUMAN] |
| PRPF3 | U4/U6 small nuclear ribonucleoprotein Prp3 OS=Homo sapiens GN=PRPF3 PE=1 SV=2 - [PRPF3_HUMAN] |
| PRPF31 | U4/U6 small nuclear ribonucleoprotein Prp31 OS=Homo sapiens GN=PRPF31 PE=1 SV=2 - [PRP31_HUMAN] |
| PRPF38A | Pre-mRNA-splicing factor 38A OS=Homo sapiens GN=PRPF38A PE=1 SV=1 - [PR38A_HUMAN] |
| PRPF4 | U4/U6 small nuclear ribonucleoprotein Prp4 OS=Homo sapiens GN=PRPF4 PE=1 SV=2 - [PRP4_HUMAN] |
| PRPF40A | Pre-mRNA-processing factor 40 homolog A OS=Homo sapiens GN=PRPF40A PE=1 SV=2 - [PR40A_HUMAN] |
| PRPF4B | Serine/threonine-protein kinase PRP4 homolog OS=Homo sapiens GN=PRPF4B PE=1 SV=3 - [PRP4B_HUMAN] |
| PRPF6 | Pre-mRNA-processing factor 6 OS=Homo sapiens GN=PRPF6 PE=1 SV=1 - [PRP6_HUMAN] |
| PRPF8 | Pre-mRNA-processing-splicing factor 8 OS=Homo sapiens GN=PRPF8 PE=1 SV=2 - [PRP8_HUMAN] |
| PRPS1 | Ribose-phosphate pyrophosphokinase 1 OS=Homo sapiens GN=PRPS1 PE=1 SV=2 - [PRPS1_HUMAN] |
| PRPS2 | Ribose-phosphate pyrophosphokinase 2 OS=Homo sapiens GN=PRPS2 PE=1 SV=2 - [PRPS2_HUMAN] |
| PSMA4 | Proteasome subunit alpha type-4 OS=Homo sapiens GN=PSMA4 PE=1 SV=1 - [PSA4_HUMAN] |
| PSMA5 | Proteasome subunit alpha type-5 OS=Homo sapiens GN=PSMA5 PE=1 SV=3 - [PSA5_HUMAN] |
| PSMA6 | Proteasome subunit alpha type-6 OS=Homo sapiens GN=PSMA6 PE=1 SV=1 - [PSA6_HUMAN] |
| PSMB4 | Proteasome subunit beta type-4 OS=Homo sapiens GN=PSMB4 PE=1 SV=4 - [PSB4_HUMAN] |
| PSMC4 | 26S protease regulatory subunit 6B OS=Homo sapiens GN=PSMC4 PE=1 SV=2 - [PRS6B_HUMAN] |
| PSMD4 | 26S proteasome non-ATPase regulatory subunit 4 OS=Homo sapiens GN=PSMD4 PE=1 SV=1 - [PSMD4_HUMAN] |
| PSMD7 | 26S proteasome non-ATPase regulatory subunit 7 OS=Homo sapiens GN=PSMD7 PE=1 SV=2 - [PSMD7_HUMAN] |
| PSME3 | Proteasome activator complex subunit 3 OS=Homo sapiens GN=PSME3 PE=1 SV=1 - [PSME3_HUMAN] |
| PTBP1 | Polypyrimidine tract-binding protein 1 OS=Homo sapiens GN=PTBP1 PE=1 SV=1 - [PTBP1_HUMAN] |
| PUF60 | Poly(U)-binding-splicing factor PUF60 OS=Homo sapiens GN=PUF60 PE=1 SV=1 - [PUF60_HUMAN] |
| PUM3 | Pumilio domain-containing protein KIAA0020 OS=Homo sapiens GN=KIAA0020 PE=1 SV=3 - [K0020_HUMAN] |
| PWP1 | Periodic tryptophan protein 1 homolog OS=Homo sapiens GN=PWP1 PE=1 SV=1 - [PWP1_HUMAN] |
| QKI | Protein quaking OS=Homo sapiens GN=QKI PE=1 SV=1 - [QKI_HUMAN] |
| RAB10 | Ras-related protein Rab-10 OS=Homo sapiens GN=RAB10 PE=1 SV=1 - [RAB10_HUMAN] |
| RACGAP1 | Rac GTPase-activating protein 1 OS=Homo sapiens GN=RACGAP1 PE=1 SV=1 - [RGAP1_HUMAN] |
| RACK1 | Guanine nucleotide-binding protein subunit beta-2-like 1 OS=Homo sapiens GN=GNB2L1 PE=1 SV=3 - [GBLP_HUMAN] |
| RAD21 | Double-strand-break repair protein rad21 homolog OS=Homo sapiens GN=RAD21 PE=1 SV=2 - [RAD21_HUMAN] |
| RAE1 | mRNA export factor OS=Homo sapiens GN=RAE1 PE=1 SV=1 - [RAE1L_HUMAN] |
| RALY | RNA-binding protein Raly OS=Homo sapiens GN=RALY PE=1 SV=1 - [RALY_HUMAN] |
| RAN | GTP-binding nuclear protein Ran OS=Homo sapiens GN=RAN PE=1 SV=3 - [RAN_HUMAN] |
| RANBP2 | E3 SUMO-protein ligase RanBP2 OS=Homo sapiens GN=RANBP2 PE=1 SV=2 - [RBP2_HUMAN] |
| RANGAP1 | Ran GTPase-activating protein 1 OS=Homo sapiens GN=RANGAP1 PE=1 SV=1 - [RAGP1_HUMAN] |
| RAP2B | Ras-related protein Rap-2b OS=Homo sapiens GN=RAP2B PE=1 SV=1 - [RAP2B_HUMAN] |
| RBBP4 | Histone-binding protein RBBP4 OS=Homo sapiens GN=RBBP4 PE=1 SV=3 - [RBBP4_HUMAN] |
| RBBP7 | Histone-binding protein RBBP7 OS=Homo sapiens GN=RBBP7 PE=1 SV=1 - [RBBP7_HUMAN] |
| RBM10 | RNA-binding protein 10 OS=Homo sapiens GN=RBM10 PE=1 SV=3 - [RBM10_HUMAN] |
| RBM12B | RNA-binding protein 12B OS=Homo sapiens GN=RBM12B PE=1 SV=2 - [RB12B_HUMAN] |
| RBM14 | RNA-binding protein 14 OS=Homo sapiens GN=RBM14 PE=1 SV=2 - [RBM14_HUMAN] |
| RBM15 | Putative RNA-binding protein 15 OS=Homo sapiens GN=RBM15 PE=1 SV=2 - [RBM15_HUMAN] |
| RBM17 | Splicing factor 45 OS=Homo sapiens GN=RBM17 PE=1 SV=1 - [SPF45_HUMAN] |
| RBM25 | RNA-binding protein 25 OS=Homo sapiens GN=RBM25 PE=1 SV=3 - [RBM25_HUMAN] |
| RBM3 | Putative RNA-binding protein 3 OS=Homo sapiens GN=RBM3 PE=1 SV=1 - [RBM3_HUMAN] |
| RBM39 | RNA-binding protein 39 OS=Homo sapiens GN=RBM39 PE=1 SV=2 - [RBM39_HUMAN] |
| RBM4 | RNA-binding protein 4 OS=Homo sapiens GN=RBM4 PE=1 SV=1 - [RBM4_HUMAN] |
| RBM42 | RNA-binding protein 42 OS=Homo sapiens GN=RBM42 PE=1 SV=1 - [RBM42_HUMAN] |
| RBM8A | RNA-binding protein 8A OS=Homo sapiens GN=RBM8A PE=1 SV=1 - [RBM8A_HUMAN] |
| RBMX | RNA-binding motif protein, X chromosome OS=Homo sapiens GN=RBMX PE=1 SV=3 - [RBMX_HUMAN] |
| RCC1 | Regulator of chromosome condensation OS=Homo sapiens GN=RCC1 PE=1 SV=1 - [RCC1_HUMAN] |
| RCC2 | Protein RCC2 OS=Homo sapiens GN=RCC2 PE=1 SV=2 - [RCC2_HUMAN] |
| RDX | Radixin OS=Homo sapiens GN=RDX PE=1 SV=1 - [RADI_HUMAN] |
| RECQL | ATP-dependent DNA helicase Q1 OS=Homo sapiens GN=RECQL PE=1 SV=3 - [RECQ1_HUMAN] |
| RFC2 | Replication factor C subunit 2 OS=Homo sapiens GN=RFC2 PE=1 SV=3 - [RFC2_HUMAN] |
| RFC5 | Replication factor C subunit 5 OS=Homo sapiens GN=RFC5 PE=1 SV=1 - [RFC5_HUMAN] |
| RFLNB | Protein FAM101B OS=Homo sapiens GN=FAM101B PE=1 SV=1 - [F101B_HUMAN] |
| RGPD1 | RANBP2-like and GRIP domain-containing protein 1 OS=Homo sapiens GN=RGPD1 PE=2 SV=1 - [RGPD1_HUMAN] |
| RGPD5 | RANBP2-like and GRIP domain-containing protein 5/6 OS=Homo sapiens GN=RGPD5 PE=1 SV=3 - [RGPD5_HUMAN] |
| RNF2 | E3 ubiquitin-protein ligase RING2 OS=Homo sapiens GN=RNF2 PE=1 SV=1 - [RING2_HUMAN] |
| RNH1 | Ribonuclease inhibitor OS=Homo sapiens GN=RNH1 PE=1 SV=2 - [RINI_HUMAN] |
| RNPS1 | RNA-binding protein with serine-rich domain 1 OS=Homo sapiens GN=RNPS1 PE=1 SV=1 - [RNPS1_HUMAN] |
| RPA1 | Replication protein A 70 kDa DNA-binding subunit OS=Homo sapiens GN=RPA1 PE=1 SV=2 - [RFA1_HUMAN] |
| RPA2 | Replication protein A 32 kDa subunit OS=Homo sapiens GN=RPA2 PE=1 SV=1 - [RFA2_HUMAN] |
| RPF2 | Ribosome production factor 2 homolog OS=Homo sapiens GN=RPF2 PE=1 SV=2 - [RPF2_HUMAN] |
| RPL22 | 60S ribosomal protein L22 OS=Homo sapiens GN=RPL22 PE=1 SV=2 - [RL22_HUMAN] |
| RPL23 | 60S ribosomal protein L23 OS=Homo sapiens GN=RPL23 PE=1 SV=1 - [RL23_HUMAN] |
| RPL23A | 60S ribosomal protein L23a OS=Homo sapiens GN=RPL23A PE=1 SV=1 - [RL23A_HUMAN] |
| RPL30 | 60S ribosomal protein L30 OS=Homo sapiens GN=RPL30 PE=1 SV=2 - [RL30_HUMAN] |
| RPL32 | 60S ribosomal protein L32 OS=Homo sapiens GN=RPL32 PE=1 SV=2 - [RL32_HUMAN] |
| RPL34 | 60S ribosomal protein L34 OS=Homo sapiens GN=RPL34 PE=1 SV=3 - [RL34_HUMAN] |
| RPL5 | 60S ribosomal protein L5 OS=Homo sapiens GN=RPL5 PE=1 SV=3 - [RL5_HUMAN] |
| RPL9 | 60S ribosomal protein L9 OS=Homo sapiens GN=RPL9 PE=1 SV=1 - [RL9_HUMAN] |
| RPP30 | Ribonuclease P protein subunit p30 OS=Homo sapiens GN=RPP30 PE=1 SV=1 - [RPP30_HUMAN] |
| RPRD1A | Regulation of nuclear pre-mRNA domain-containing protein 1A OS=Homo sapiens GN=RPRD1A PE=1 SV=1 - [RPR1A_HUMAN] |
| RPRD1B | Regulation of nuclear pre-mRNA domain-containing protein 1B OS=Homo sapiens GN=RPRD1B PE=1 SV=1 - [RPR1B_HUMAN] |
| RPS10 | 40S ribosomal protein S10 OS=Homo sapiens GN=RPS10 PE=1 SV=1 - [RS10_HUMAN] |
| RPS11 | 40S ribosomal protein S11 OS=Homo sapiens GN=RPS11 PE=1 SV=3 - [RS11_HUMAN] |
| RPS12 | 40S ribosomal protein S12 OS=Homo sapiens GN=RPS12 PE=1 SV=3 - [RS12_HUMAN] |
| RPS15 | 40S ribosomal protein S15 OS=Homo sapiens GN=RPS15 PE=1 SV=2 - [RS15_HUMAN] |
| RPS15A | 40S ribosomal protein S15a OS=Homo sapiens GN=RPS15A PE=1 SV=2 - [RS15A_HUMAN] |
| RPS16 | 40S ribosomal protein S16 OS=Homo sapiens GN=RPS16 PE=1 SV=2 - [RS16_HUMAN] |
| RPS2 | 40S ribosomal protein S2 OS=Homo sapiens GN=RPS2 PE=1 SV=2 - [RS2_HUMAN] |
| RPS20 | 40S ribosomal protein S20 OS=Homo sapiens GN=RPS20 PE=1 SV=1 - [RS20_HUMAN] |
| RPS21 | 40S ribosomal protein S21 OS=Homo sapiens GN=RPS21 PE=1 SV=1 - [RS21_HUMAN] |
| RPS23 | 40S ribosomal protein S23 OS=Homo sapiens GN=RPS23 PE=1 SV=3 - [RS23_HUMAN] |
| RPS24 | 40S ribosomal protein S24 OS=Homo sapiens GN=RPS24 PE=1 SV=1 - [RS24_HUMAN] |
| RPS27 | 40S ribosomal protein S27 OS=Homo sapiens GN=RPS27 PE=1 SV=3 - [RS27_HUMAN] |
| RPS27A | Ubiquitin-40S ribosomal protein S27a OS=Homo sapiens GN=RPS27A PE=1 SV=2 - [RS27A_HUMAN] |
| RPS9 | 40S ribosomal protein S9 OS=Homo sapiens GN=RPS9 PE=1 SV=3 - [RS9_HUMAN] |
| RPSA | 40S ribosomal protein SA OS=Homo sapiens GN=RPSA PE=1 SV=4 - [RSSA_HUMAN] |
| RRP1 | Ribosomal RNA processing protein 1 homolog A OS=Homo sapiens GN=RRP1 PE=1 SV=1 - [RRP1_HUMAN] |
| RRP1B | Ribosomal RNA processing protein 1 homolog B OS=Homo sapiens GN=RRP1B PE=1 SV=3 - [RRP1B_HUMAN] |
| RRP9 | U3 small nucleolar RNA-interacting protein 2 OS=Homo sapiens GN=RRP9 PE=1 SV=1 - [U3IP2_HUMAN] |
| RSL1D1 | Ribosomal L1 domain-containing protein 1 OS=Homo sapiens GN=RSL1D1 PE=1 SV=3 - [RL1D1_HUMAN] |
| RSL24D1 | Probable ribosome biogenesis protein RLP24 OS=Homo sapiens GN=RSL24D1 PE=1 SV=1 - [RLP24_HUMAN] |
| RSPRY1 | RING finger and SPRY domain-containing protein 1 OS=Homo sapiens GN=RSPRY1 PE=2 SV=1 - [RSPRY_HUMAN] |
| RTCB | tRNA-splicing ligase RtcB homolog OS=Homo sapiens GN=RTCB PE=1 SV=1 - [RTCB_HUMAN] |
| RTRAF | UPF0568 protein C14orf166 OS=Homo sapiens GN=C14orf166 PE=1 SV=1 - [CN166_HUMAN] |
| RUVBL1 | RuvB-like 1 OS=Homo sapiens GN=RUVBL1 PE=1 SV=1 - [RUVB1_HUMAN] |
| RUVBL2 | RuvB-like 2 OS=Homo sapiens GN=RUVBL2 PE=1 SV=3 - [RUVB2_HUMAN] |
| SAC3D1 | SAC3 domain-containing protein 1 OS=Homo sapiens GN=SAC3D1 PE=1 SV=2 - [SAC31_HUMAN] |
| SAFB | Scaffold attachment factor B1 OS=Homo sapiens GN=SAFB PE=1 SV=4 - [SAFB1_HUMAN] |
| SAFB2 | Scaffold attachment factor B2 OS=Homo sapiens GN=SAFB2 PE=1 SV=1 - [SAFB2_HUMAN] |
| SAP18 | Histone deacetylase complex subunit SAP18 OS=Homo sapiens GN=SAP18 PE=1 SV=1 - [SAP18_HUMAN] |
| SAP30BP | SAP30-binding protein OS=Homo sapiens GN=SAP30BP PE=1 SV=1 - [S30BP_HUMAN] |
| SARNP | SAP domain-containing ribonucleoprotein OS=Homo sapiens GN=SARNP PE=1 SV=3 - [SARNP_HUMAN] |
| SART1 | U4/U6.U5 tri-snRNP-associated protein 1 OS=Homo sapiens GN=SART1 PE=1 SV=1 - [SNUT1_HUMAN] |
| SCRIB | Protein scribble homolog OS=Homo sapiens GN=SCRIB PE=1 SV=4 - [SCRIB_HUMAN] |
| SEC13 | Protein SEC13 homolog OS=Homo sapiens GN=SEC13 PE=1 SV=3 - [SEC13_HUMAN] |
| SEH1L | Nucleoporin SEH1 OS=Homo sapiens GN=SEH1L PE=1 SV=3 - [SEH1_HUMAN] |
| SEP9 | Septin-9 OS=Homo sapiens GN=SEPT9 PE=1 SV=2 - [SEPT9_HUMAN] |
| SEPT2 | Septin-2 OS=Homo sapiens GN=SEPT2 PE=1 SV=1 - [SEPT2_HUMAN] |
| SEPT7 | Septin-7 OS=Homo sapiens GN=SEPT7 PE=1 SV=2 - [SEPT7_HUMAN] |
| SF1 | Splicing factor 1 OS=Homo sapiens GN=SF1 PE=1 SV=4 - [SF01_HUMAN] |
| SF3A2 | Splicing factor 3A subunit 2 OS=Homo sapiens GN=SF3A2 PE=1 SV=2 - [SF3A2_HUMAN] |
| SF3A3 | Splicing factor 3A subunit 3 OS=Homo sapiens GN=SF3A3 PE=1 SV=1 - [SF3A3_HUMAN] |
| SF3B1 | Splicing factor 3B subunit 1 OS=Homo sapiens GN=SF3B1 PE=1 SV=3 - [SF3B1_HUMAN] |
| SF3B2 | Splicing factor 3B subunit 2 OS=Homo sapiens GN=SF3B2 PE=1 SV=2 - [SF3B2_HUMAN] |
| SF3B3 | Splicing factor 3B subunit 3 OS=Homo sapiens GN=SF3B3 PE=1 SV=4 - [SF3B3_HUMAN] |
| SF3B4 | Splicing factor 3B subunit 4 OS=Homo sapiens GN=SF3B4 PE=1 SV=1 - [SF3B4_HUMAN] |
| SF3B6 | Pre-mRNA branch site protein p14 OS=Homo sapiens GN=SF3B14 PE=1 SV=1 - [PM14_HUMAN] |
| SFPQ | Splicing factor, proline- and glutamine-rich OS=Homo sapiens GN=SFPQ PE=1 SV=2 - [SFPQ_HUMAN] |
| SFSWAP | Splicing factor, suppressor of white-apricot homolog OS=Homo sapiens GN=SFSWAP PE=1 SV=3 - [SFSWA_HUMAN] |
| SH3BGRL3 | SH3 domain-binding glutamic acid-rich-like protein 3 OS=Homo sapiens GN=SH3BGRL3 PE=1 SV=1 - [SH3L3_HUMAN] |
| SIPA1L3 | Signal-induced proliferation-associated 1-like protein 3 OS=Homo sapiens GN=SIPA1L3 PE=1 SV=3 - [SI1L3_HUMAN] |
| SKP1 | S-phase kinase-associated protein 1 OS=Homo sapiens GN=SKP1 PE=1 SV=2 - [SKP1_HUMAN] |
| SLC25A5 | ADP/ATP translocase 2 OS=Homo sapiens GN=SLC25A5 PE=1 SV=7 - [ADT2_HUMAN] |
| SLC25A6 | ADP/ATP translocase 3 OS=Homo sapiens GN=SLC25A6 PE=1 SV=4 - [ADT3_HUMAN] |
| SLTM | SAFB-like transcription modulator OS=Homo sapiens GN=SLTM PE=1 SV=2 - [SLTM_HUMAN] |
| SMARCA5 | SWI/SNF-related matrix-associated actin-dependent regulator of chromatin subfamily A member 5 OS=Homo sapiens GN=SMARCA5 PE=1 SV=1 - [SMCA5_HUMAN] |
| SMC1A | Structural maintenance of chromosomes protein 1A OS=Homo sapiens GN=SMC1A PE=1 SV=2 - [SMC1A_HUMAN] |
| SMC3 | Structural maintenance of chromosomes protein 3 OS=Homo sapiens GN=SMC3 PE=1 SV=2 - [SMC3_HUMAN] |
| SMCHD1 | Structural maintenance of chromosomes flexible hinge domain-containing protein 1 OS=Homo sapiens GN=SMCHD1 PE=1 SV=2 - [SMHD1_HUMAN] |
| SMU1 | WD40 repeat-containing protein SMU1 OS=Homo sapiens GN=SMU1 PE=1 SV=2 - [SMU1_HUMAN] |
| SND1 | Staphylococcal nuclease domain-containing protein 1 OS=Homo sapiens GN=SND1 PE=1 SV=1 - [SND1_HUMAN] |
| SNRNP200 | U5 small nuclear ribonucleoprotein 200 kDa helicase OS=Homo sapiens GN=SNRNP200 PE=1 SV=2 - [U520_HUMAN] |
| SNRNP27 | U4/U6.U5 small nuclear ribonucleoprotein 27 kDa protein OS=Homo sapiens GN=SNRNP27 PE=1 SV=1 - [SNR27_HUMAN] |
| SNRNP40 | U5 small nuclear ribonucleoprotein 40 kDa protein OS=Homo sapiens GN=SNRNP40 PE=1 SV=1 - [SNR40_HUMAN] |
| SNRNP70 | U1 small nuclear ribonucleoprotein 70 kDa OS=Homo sapiens GN=SNRNP70 PE=1 SV=2 - [RU17_HUMAN] |
| SNRPA | U1 small nuclear ribonucleoprotein A OS=Homo sapiens GN=SNRPA PE=1 SV=3 - [SNRPA_HUMAN] |
| SNRPA1 | U2 small nuclear ribonucleoprotein A' OS=Homo sapiens GN=SNRPA1 PE=1 SV=2 - [RU2A_HUMAN] |
| SNRPB | Small nuclear ribonucleoprotein-associated proteins B and B' OS=Homo sapiens GN=SNRPB PE=1 SV=2 - [RSMB_HUMAN] |
| SNRPB2 | U2 small nuclear ribonucleoprotein B'' OS=Homo sapiens GN=SNRPB2 PE=1 SV=1 - [RU2B_HUMAN] |
| SNRPC | U1 small nuclear ribonucleoprotein C OS=Homo sapiens GN=SNRPC PE=1 SV=1 - [RU1C_HUMAN] |
| SNRPD1 | Small nuclear ribonucleoprotein Sm D1 OS=Homo sapiens GN=SNRPD1 PE=1 SV=1 - [SMD1_HUMAN] |
| SNRPD2 | Small nuclear ribonucleoprotein Sm D2 OS=Homo sapiens GN=SNRPD2 PE=1 SV=1 - [SMD2_HUMAN] |
| SNRPD3 | Small nuclear ribonucleoprotein Sm D3 OS=Homo sapiens GN=SNRPD3 PE=1 SV=1 - [SMD3_HUMAN] |
| SNRPE | Small nuclear ribonucleoprotein E OS=Homo sapiens GN=SNRPE PE=1 SV=1 - [RUXE_HUMAN] |
| SNRPF | Small nuclear ribonucleoprotein F OS=Homo sapiens GN=SNRPF PE=1 SV=1 - [RUXF_HUMAN] |
| SNTB2 | Beta-2-syntrophin OS=Homo sapiens GN=SNTB2 PE=1 SV=1 - [SNTB2_HUMAN] |
| SNU13 | NHP2-like protein 1 OS=Homo sapiens GN=NHP2L1 PE=1 SV=3 - [NH2L1_HUMAN] |
| SPECC1L | Cytospin-A OS=Homo sapiens GN=SPECC1L PE=1 SV=2 - [CYTSA_HUMAN] |
| SPTAN1 | Spectrin alpha chain, non-erythrocytic 1 OS=Homo sapiens GN=SPTAN1 PE=1 SV=3 - [SPTN1_HUMAN] |
| SPTBN1 | Spectrin beta chain, non-erythrocytic 1 OS=Homo sapiens GN=SPTBN1 PE=1 SV=2 - [SPTB2_HUMAN] |
| SPTBN2 | Spectrin beta chain, non-erythrocytic 2 OS=Homo sapiens GN=SPTBN2 PE=1 SV=3 - [SPTN2_HUMAN] |
| SQSTM1 | Sequestosome-1 OS=Homo sapiens GN=SQSTM1 PE=1 SV=1 - [SQSTM_HUMAN] |
| SRGAP2 | SLIT-ROBO Rho GTPase-activating protein 2 OS=Homo sapiens GN=SRGAP2 PE=1 SV=3 - [SRGP2_HUMAN] |
| SRP14 | Signal recognition particle 14 kDa protein OS=Homo sapiens GN=SRP14 PE=1 SV=2 - [SRP14_HUMAN] |
| SRP9 | Signal recognition particle 9 kDa protein OS=Homo sapiens GN=SRP9 PE=1 SV=2 - [SRP09_HUMAN] |
| SRRM1 | Serine/arginine repetitive matrix protein 1 OS=Homo sapiens GN=SRRM1 PE=1 SV=2 - [SRRM1_HUMAN] |
| SRRM2 | Serine/arginine repetitive matrix protein 2 OS=Homo sapiens GN=SRRM2 PE=1 SV=2 - [SRRM2_HUMAN] |
| SRRT | Serrate RNA effector molecule homolog OS=Homo sapiens GN=SRRT PE=1 SV=1 - [SRRT_HUMAN] |
| SRSF1 | Serine/arginine-rich splicing factor 1 OS=Homo sapiens GN=SRSF1 PE=1 SV=2 - [SRSF1_HUMAN] |
| SRSF10 | Serine/arginine-rich splicing factor 10 OS=Homo sapiens GN=SRSF10 PE=1 SV=1 - [SRS10_HUMAN] |
| SRSF11 | Serine/arginine-rich splicing factor 11 OS=Homo sapiens GN=SRSF11 PE=1 SV=1 - [SRS11_HUMAN] |
| SRSF3 | Serine/arginine-rich splicing factor 3 OS=Homo sapiens GN=SRSF3 PE=1 SV=1 - [SRSF3_HUMAN] |
| SRSF4 | Serine/arginine-rich splicing factor 4 OS=Homo sapiens GN=SRSF4 PE=1 SV=2 - [SRSF4_HUMAN] |
| SRSF5 | Serine/arginine-rich splicing factor 5 OS=Homo sapiens GN=SRSF5 PE=1 SV=1 - [SRSF5_HUMAN] |
| SRSF6 | Serine/arginine-rich splicing factor 6 OS=Homo sapiens GN=SRSF6 PE=1 SV=2 - [SRSF6_HUMAN] |
| SRSF7 | Serine/arginine-rich splicing factor 7 OS=Homo sapiens GN=SRSF7 PE=1 SV=1 - [SRSF7_HUMAN] |
| SRSF9 | Serine/arginine-rich splicing factor 9 OS=Homo sapiens GN=SRSF9 PE=1 SV=1 - [SRSF9_HUMAN] |
| SSBP1 | Single-stranded DNA-binding protein, mitochondrial OS=Homo sapiens GN=SSBP1 PE=1 SV=1 - [SSBP_HUMAN] |
| SSFA2 | Sperm-specific antigen 2 OS=Homo sapiens GN=SSFA2 PE=1 SV=3 - [SSFA2_HUMAN] |
| SSH2 | Protein phosphatase Slingshot homolog 2 OS=Homo sapiens GN=SSH2 PE=1 SV=1 - [SSH2_HUMAN] |
| SSRP1 | FACT complex subunit SSRP1 OS=Homo sapiens GN=SSRP1 PE=1 SV=1 - [SSRP1_HUMAN] |
| STAG2 | Cohesin subunit SA-2 OS=Homo sapiens GN=STAG2 PE=1 SV=3 - [STAG2_HUMAN] |
| STIP1 | Stress-induced-phosphoprotein 1 OS=Homo sapiens GN=STIP1 PE=1 SV=1 - [STIP1_HUMAN] |
| STRAP | Serine-threonine kinase receptor-associated protein OS=Homo sapiens GN=STRAP PE=1 SV=1 - [STRAP_HUMAN] |
| SUB1 | Activated RNA polymerase II transcriptional coactivator p15 OS=Homo sapiens GN=SUB1 PE=1 SV=3 - [TCP4_HUMAN] |
| SUGP2 | SURP and G-patch domain-containing protein 2 OS=Homo sapiens GN=SUGP2 PE=1 SV=2 - [SUGP2_HUMAN] |
| SUMO1 | Small ubiquitin-related modifier 1 OS=Homo sapiens GN=SUMO1 PE=1 SV=1 - [SUMO1_HUMAN] |
| SUMO2 | Small ubiquitin-related modifier 2 OS=Homo sapiens GN=SUMO2 PE=1 SV=3 - [SUMO2_HUMAN] |
| SUPT16H | FACT complex subunit SPT16 OS=Homo sapiens GN=SUPT16H PE=1 SV=1 - [SP16H_HUMAN] |
| SUPT5H | Transcription elongation factor SPT5 OS=Homo sapiens GN=SUPT5H PE=1 SV=1 - [SPT5H_HUMAN] |
| SUPT6H | Transcription elongation factor SPT6 OS=Homo sapiens GN=SUPT6H PE=1 SV=2 - [SPT6H_HUMAN] |
| SUZ12 | Polycomb protein SUZ12 OS=Homo sapiens GN=SUZ12 PE=1 SV=3 - [SUZ12_HUMAN] |
| SVIL | Supervillin OS=Homo sapiens GN=SVIL PE=1 SV=2 - [SVIL_HUMAN] |
| SYMPK | Symplekin OS=Homo sapiens GN=SYMPK PE=1 SV=2 - [SYMPK_HUMAN] |
| SYNCRIP | Heterogeneous nuclear ribonucleoprotein Q OS=Homo sapiens GN=SYNCRIP PE=1 SV=2 - [HNRPQ_HUMAN] |
| SYNM | Synemin OS=Homo sapiens GN=SYNM PE=1 SV=2 - [SYNEM_HUMAN] |
| TARDBP | TAR DNA-binding protein 43 OS=Homo sapiens GN=TARDBP PE=1 SV=1 - [TADBP_HUMAN] |
| TBL3 | Transducin beta-like protein 3 OS=Homo sapiens GN=TBL3 PE=1 SV=2 - [TBL3_HUMAN] |
| TCERG1 | Transcription elongation regulator 1 OS=Homo sapiens GN=TCERG1 PE=1 SV=2 - [TCRG1_HUMAN] |
| TCP1 | T-complex protein 1 subunit alpha OS=Homo sapiens GN=TCP1 PE=1 SV=1 - [TCPA_HUMAN] |
| TFCP2 | Alpha-globin transcription factor CP2 OS=Homo sapiens GN=TFCP2 PE=1 SV=2 - [TFCP2_HUMAN] |
| TFIP11 | Tuftelin-interacting protein 11 OS=Homo sapiens GN=TFIP11 PE=1 SV=1 - [TFP11_HUMAN] |
| TFRC | Transferrin receptor protein 1 OS=Homo sapiens GN=TFRC PE=1 SV=2 - [TFR1_HUMAN] |
| TGM2 | Protein-glutamine gamma-glutamyltransferase 2 OS=Homo sapiens GN=TGM2 PE=1 SV=2 - [TGM2_HUMAN] |
| THOC1 | THO complex subunit 1 OS=Homo sapiens GN=THOC1 PE=1 SV=1 - [THOC1_HUMAN] |
| THOC2 | THO complex subunit 2 OS=Homo sapiens GN=THOC2 PE=1 SV=2 - [THOC2_HUMAN] |
| THOC3 | THO complex subunit 3 OS=Homo sapiens GN=THOC3 PE=1 SV=1 - [THOC3_HUMAN] |
| THOC5 | THO complex subunit 5 homolog OS=Homo sapiens GN=THOC5 PE=1 SV=2 - [THOC5_HUMAN] |
| THOC6 | THO complex subunit 6 homolog OS=Homo sapiens GN=THOC6 PE=1 SV=1 - [THOC6_HUMAN] |
| THOC7 | THO complex subunit 7 homolog OS=Homo sapiens GN=THOC7 PE=1 SV=3 - [THOC7_HUMAN] |
| THRAP3 | Thyroid hormone receptor-associated protein 3 OS=Homo sapiens GN=THRAP3 PE=1 SV=2 - [TR150_HUMAN] |
| TIAL1 | Nucleolysin TIAR OS=Homo sapiens GN=TIAL1 PE=1 SV=1 - [TIAR_HUMAN] |
| TJP1 | Tight junction protein ZO-1 OS=Homo sapiens GN=TJP1 PE=1 SV=3 - [ZO1_HUMAN] |
| TMOD3 | Tropomodulin-3 OS=Homo sapiens GN=TMOD3 PE=1 SV=1 - [TMOD3_HUMAN] |
| TMPO | Lamina-associated polypeptide 2, isoforms beta/gamma OS=Homo sapiens GN=TMPO PE=1 SV=2 - [LAP2B_HUMAN] |
| TMPO | Lamina-associated polypeptide 2, isoform alpha OS=Homo sapiens GN=TMPO PE=1 SV=2 - [LAP2A_HUMAN] |
| TNKS1BP1 | 182 kDa tankyrase-1-binding protein OS=Homo sapiens GN=TNKS1BP1 PE=1 SV=4 - [TB182_HUMAN] |
| TOP1 | DNA topoisomerase 1 OS=Homo sapiens GN=TOP1 PE=1 SV=2 - [TOP1_HUMAN] |
| TOP2A | DNA topoisomerase 2-alpha OS=Homo sapiens GN=TOP2A PE=1 SV=3 - [TOP2A_HUMAN] |
| TPM3 | Tropomyosin alpha-3 chain OS=Homo sapiens GN=TPM3 PE=1 SV=2 - [TPM3_HUMAN] |
| TPR | Nucleoprotein TPR OS=Homo sapiens GN=TPR PE=1 SV=3 - [TPR_HUMAN] |
| TPRN | Taperin OS=Homo sapiens GN=TPRN PE=1 SV=2 - [TPRN_HUMAN] |
| TRA2A | Transformer-2 protein homolog alpha OS=Homo sapiens GN=TRA2A PE=1 SV=1 - [TRA2A_HUMAN] |
| TRA2B | Transformer-2 protein homolog beta OS=Homo sapiens GN=TRA2B PE=1 SV=1 - [TRA2B_HUMAN] |
| TRIM28 | Transcription intermediary factor 1-beta OS=Homo sapiens GN=TRIM28 PE=1 SV=5 - [TIF1B_HUMAN] |
| TRIP12 | E3 ubiquitin-protein ligase TRIP12 OS=Homo sapiens GN=TRIP12 PE=1 SV=1 - [TRIPC_HUMAN] |
| TTC7B | Tetratricopeptide repeat protein 7B OS=Homo sapiens GN=TTC7B PE=1 SV=3 - [TTC7B_HUMAN] |
| TUBB2A | Tubulin beta-2A chain OS=Homo sapiens GN=TUBB2A PE=1 SV=1 - [TBB2A_HUMAN] |
| TUBB3 | Tubulin beta-3 chain OS=Homo sapiens GN=TUBB3 PE=1 SV=2 - [TBB3_HUMAN] |
| TUFM | Elongation factor Tu, mitochondrial OS=Homo sapiens GN=TUFM PE=1 SV=2 - [EFTU_HUMAN] |
| TWF1 | Twinfilin-1 OS=Homo sapiens GN=TWF1 PE=1 SV=3 - [TWF1_HUMAN] |
| TWF2 | Twinfilin-2 OS=Homo sapiens GN=TWF2 PE=1 SV=2 - [TWF2_HUMAN] |
| TWISTNB | DNA-directed RNA polymerase I subunit RPA43 OS=Homo sapiens GN=TWISTNB PE=1 SV=1 - [RPA43_HUMAN] |
| TXNDC5 | Thioredoxin domain-containing protein 5 OS=Homo sapiens GN=TXNDC5 PE=1 SV=2 - [TXND5_HUMAN] |
| TXNL4A | Thioredoxin-like protein 4A OS=Homo sapiens GN=TXNL4A PE=1 SV=1 - [TXN4A_HUMAN] |
| U2AF1 | Splicing factor U2AF 35 kDa subunit OS=Homo sapiens GN=U2AF1 PE=1 SV=3 - [U2AF1_HUMAN] |
| U2AF2 | Splicing factor U2AF 65 kDa subunit OS=Homo sapiens GN=U2AF2 PE=1 SV=4 - [U2AF2_HUMAN] |
| U2SURP | U2 snRNP-associated SURP motif-containing protein OS=Homo sapiens GN=U2SURP PE=1 SV=2 - [SR140_HUMAN] |
| UACA | Uveal autoantigen with coiled-coil domains and ankyrin repeats OS=Homo sapiens GN=UACA PE=1 SV=2 - [UACA_HUMAN] |
| UBAP2L | Ubiquitin-associated protein 2-like OS=Homo sapiens GN=UBAP2L PE=1 SV=2 - [UBP2L_HUMAN] |
| UBE2I | SUMO-conjugating enzyme UBC9 OS=Homo sapiens GN=UBE2I PE=1 SV=1 - [UBC9_HUMAN] |
| UBP1 | Upstream-binding protein 1 OS=Homo sapiens GN=UBP1 PE=2 SV=1 - [UBIP1_HUMAN] |
| UBTF | Nucleolar transcription factor 1 OS=Homo sapiens GN=UBTF PE=1 SV=1 - [UBF1_HUMAN] |
| UGGT1 | UDP-glucose:glycoprotein glucosyltransferase 1 OS=Homo sapiens GN=UGGT1 PE=1 SV=3 - [UGGG1_HUMAN] |
| UHRF1 | E3 ubiquitin-protein ligase UHRF1 OS=Homo sapiens GN=UHRF1 PE=1 SV=1 - [UHRF1_HUMAN] |
| UPF1 | Regulator of nonsense transcripts 1 OS=Homo sapiens GN=UPF1 PE=1 SV=2 - [RENT1_HUMAN] |
| URB1 | Nucleolar pre-ribosomal-associated protein 1 OS=Homo sapiens GN=URB1 PE=1 SV=4 - [NPA1P_HUMAN] |
| USP39 | U4/U6.U5 tri-snRNP-associated protein 2 OS=Homo sapiens GN=USP39 PE=1 SV=2 - [SNUT2_HUMAN] |
| USP6NL | USP6 N-terminal-like protein OS=Homo sapiens GN=USP6NL PE=1 SV=3 - [US6NL_HUMAN] |
| USP7 | Ubiquitin carboxyl-terminal hydrolase 7 OS=Homo sapiens GN=USP7 PE=1 SV=2 - [UBP7_HUMAN] |
| UTP15 | U3 small nucleolar RNA-associated protein 15 homolog OS=Homo sapiens GN=UTP15 PE=1 SV=3 - [UTP15_HUMAN] |
| UTP4 | Cirhin OS=Homo sapiens GN=CIRH1A PE=1 SV=1 - [CIR1A_HUMAN] |
| UTP6 | U3 small nucleolar RNA-associated protein 6 homolog OS=Homo sapiens GN=UTP6 PE=1 SV=2 - [UTP6_HUMAN] |
| VASP | Vasodilator-stimulated phosphoprotein OS=Homo sapiens GN=VASP PE=1 SV=3 - [VASP_HUMAN] |
| VCP | Transitional endoplasmic reticulum ATPase OS=Homo sapiens GN=VCP PE=1 SV=4 - [TERA_HUMAN] |
| VIM | Vimentin OS=Homo sapiens GN=VIM PE=1 SV=4 - [VIME_HUMAN] |
| VRK1 | Serine/threonine-protein kinase VRK1 OS=Homo sapiens GN=VRK1 PE=1 SV=1 - [VRK1_HUMAN] |
| VTA1 | Vacuolar protein sorting-associated protein VTA1 homolog OS=Homo sapiens GN=VTA1 PE=1 SV=1 - [VTA1_HUMAN] |
| WBP11 | WW domain-binding protein 11 OS=Homo sapiens GN=WBP11 PE=1 SV=1 - [WBP11_HUMAN] |
| WDR1 | WD repeat-containing protein 1 OS=Homo sapiens GN=WDR1 PE=1 SV=4 - [WDR1_HUMAN] |
| WDR3 | WD repeat-containing protein 3 OS=Homo sapiens GN=WDR3 PE=1 SV=1 - [WDR3_HUMAN] |
| WDR33 | pre-mRNA 3' end processing protein WDR33 OS=Homo sapiens GN=WDR33 PE=1 SV=2 - [WDR33_HUMAN] |
| WDR36 | WD repeat-containing protein 36 OS=Homo sapiens GN=WDR36 PE=1 SV=1 - [WDR36_HUMAN] |
| WDR43 | WD repeat-containing protein 43 OS=Homo sapiens GN=WDR43 PE=1 SV=3 - [WDR43_HUMAN] |
| WDR5 | WD repeat-containing protein 5 OS=Homo sapiens GN=WDR5 PE=1 SV=1 - [WDR5_HUMAN] |
| WDR55 | WD repeat-containing protein 55 OS=Homo sapiens GN=WDR55 PE=1 SV=2 - [WDR55_HUMAN] |
| WDR61 | WD repeat-containing protein 61 OS=Homo sapiens GN=WDR61 PE=1 SV=1 - [WDR61_HUMAN] |
| WDR75 | WD repeat-containing protein 75 OS=Homo sapiens GN=WDR75 PE=1 SV=1 - [WDR75_HUMAN] |
| WRNIP1 | ATPase WRNIP1 OS=Homo sapiens GN=WRNIP1 PE=1 SV=2 - [WRIP1_HUMAN] |
| XRCC1 | DNA repair protein XRCC1 OS=Homo sapiens GN=XRCC1 PE=1 SV=2 - [XRCC1_HUMAN] |
| XRCC5 | X-ray repair cross-complementing protein 5 OS=Homo sapiens GN=XRCC5 PE=1 SV=3 - [XRCC5_HUMAN] |
| XRCC6 | X-ray repair cross-complementing protein 6 OS=Homo sapiens GN=XRCC6 PE=1 SV=2 - [XRCC6_HUMAN] |
| XRN2 | 5'-3' exoribonuclease 2 OS=Homo sapiens GN=XRN2 PE=1 SV=1 - [XRN2_HUMAN] |
| YES1 | Tyrosine-protein kinase Yes OS=Homo sapiens GN=YES1 PE=1 SV=3 - [YES_HUMAN] |
| YLPM1 | YLP motif-containing protein 1 OS=Homo sapiens GN=YLPM1 PE=1 SV=3 - [YLPM1_HUMAN] |
| YTHDF2 | YTH domain family protein 2 OS=Homo sapiens GN=YTHDF2 PE=1 SV=2 - [YTHD2_HUMAN] |
| YWHAB | 14-3-3 protein beta/alpha OS=Homo sapiens GN=YWHAB PE=1 SV=3 - [1433B_HUMAN] |
| YWHAE | 14-3-3 protein epsilon OS=Homo sapiens GN=YWHAE PE=1 SV=1 - [1433E_HUMAN] |
| YWHAG | 14-3-3 protein gamma OS=Homo sapiens GN=YWHAG PE=1 SV=2 - [1433G_HUMAN] |
| YWHAH | 14-3-3 protein eta OS=Homo sapiens GN=YWHAH PE=1 SV=4 - [1433F_HUMAN] |
| YWHAQ | 14-3-3 protein theta OS=Homo sapiens GN=YWHAQ PE=1 SV=1 - [1433T_HUMAN] |
| YWHAZ | 14-3-3 protein zeta/delta OS=Homo sapiens GN=YWHAZ PE=1 SV=1 - [1433Z_HUMAN] |
| ZC3H11A | Zinc finger CCCH domain-containing protein 11A OS=Homo sapiens GN=ZC3H11A PE=1 SV=3 - [ZC11A_HUMAN] |
| ZC3H14 | Zinc finger CCCH domain-containing protein 14 OS=Homo sapiens GN=ZC3H14 PE=1 SV=1 - [ZC3HE_HUMAN] |
| ZC3H18 | Zinc finger CCCH domain-containing protein 18 OS=Homo sapiens GN=ZC3H18 PE=1 SV=2 - [ZCH18_HUMAN] |
| ZC3HAV1 | Zinc finger CCCH-type antiviral protein 1 OS=Homo sapiens GN=ZC3HAV1 PE=1 SV=3 - [ZCCHV_HUMAN] |
| ZC3HC1 | Nuclear-interacting partner of ALK OS=Homo sapiens GN=ZC3HC1 PE=1 SV=1 - [NIPA_HUMAN] |
| ZFR | Zinc finger RNA-binding protein OS=Homo sapiens GN=ZFR PE=1 SV=2 - [ZFR_HUMAN] |
| ZNF207 | BUB3-interacting and GLEBS motif-containing protein ZNF207 OS=Homo sapiens GN=ZNF207 PE=1 SV=1 - [ZN207_HUMAN] |
| ZNF326 | DBIRD complex subunit ZNF326 OS=Homo sapiens GN=ZNF326 PE=1 SV=2 - [ZN326_HUMAN] |
