# Supplementary material for: Targeting NUPR1-dependent stress granules formation to induce synthetic lethality in KrasG12D-driven tumors
Source: EMBO Mol Med. 2024 Feb 15;16(3):4. doi: 10.1038/s44321-024-00032-2 (PMC10940650; doi:10.1038/s44321-024-00032-2)
Supplement: Supplementary file 6 — Dataset EV5 [file 44321_2024_32_MOESM6_ESM.docx]

**DatasetEV5: NUPR1-GFP interactors in control condition.** In green, interactors identified to localize to stress granules.

| **Gene ID** | **Description** |
| --- | --- |
| ACIN1 | Apoptotic chromatin condensation inducer in the nucleus OS=Homo sapiens GN=ACIN1 PE=1 SV=2 - [ACINU_HUMAN] |
| ACTBL2 | Beta-actin-like protein 2 OS=Homo sapiens GN=ACTBL2 PE=1 SV=2 - [ACTBL_HUMAN] |
| ACTC1 | Actin, alpha cardiac muscle 1 OS=Homo sapiens GN=ACTC1 PE=1 SV=1 - [ACTC_HUMAN] |
| ACTN1 | Alpha-actinin-1 OS=Homo sapiens GN=ACTN1 PE=1 SV=2 - [ACTN1_HUMAN] |
| ACTN4 | Alpha-actinin-4 OS=Homo sapiens GN=ACTN4 PE=1 SV=2 - [ACTN4_HUMAN] |
| ACTR2 | Actin-related protein 2 OS=Homo sapiens GN=ACTR2 PE=1 SV=1 - [ARP2_HUMAN] |
| ADAR | Double-stranded RNA-specific adenosine deaminase OS=Homo sapiens GN=ADAR PE=1 SV=4 - [DSRAD_HUMAN] |
| AFAP1 | Actin filament-associated protein 1 OS=Homo sapiens GN=AFAP1 PE=1 SV=2 - [AFAP1_HUMAN] |
| AGO2 | Protein argonaute-2 OS=Homo sapiens GN=AGO2 PE=1 SV=3 - [AGO2_HUMAN] |
| ALYREF | THO complex subunit 4 OS=Homo sapiens GN=ALYREF PE=1 SV=3 - [THOC4_HUMAN] |
| AP2A1 | AP-2 complex subunit alpha-1 OS=Homo sapiens GN=AP2A1 PE=1 SV=3 - [AP2A1_HUMAN] |
| AP2A2 | AP-2 complex subunit alpha-2 OS=Homo sapiens GN=AP2A2 PE=1 SV=2 - [AP2A2_HUMAN] |
| AP2M1 | AP-2 complex subunit mu OS=Homo sapiens GN=AP2M1 PE=1 SV=2 - [AP2M1_HUMAN] |
| ARHGEF11 | Rho guanine nucleotide exchange factor 11 OS=Homo sapiens GN=ARHGEF11 PE=1 SV=1 - [ARHGB_HUMAN] |
| ARHGEF17 | Rho guanine nucleotide exchange factor 17 OS=Homo sapiens GN=ARHGEF17 PE=1 SV=1 - [ARHGH_HUMAN] |
| ARPC3 | Actin-related protein 2/3 complex subunit 3 OS=Homo sapiens GN=ARPC3 PE=1 SV=3 - [ARPC3_HUMAN] |
| ARPC5 | Actin-related protein 2/3 complex subunit 5 OS=Homo sapiens GN=ARPC5 PE=1 SV=3 - [ARPC5_HUMAN] |
| ARPC5L | Actin-related protein 2/3 complex subunit 5-like protein OS=Homo sapiens GN=ARPC5L PE=1 SV=1 - [ARP5L_HUMAN] |
| ATXN2L | Ataxin-2-like protein OS=Homo sapiens GN=ATXN2L PE=1 SV=2 - [ATX2L_HUMAN] |
| BAIAP2 | Brain-specific angiogenesis inhibitor 1-associated protein 2 OS=Homo sapiens GN=BAIAP2 PE=1 SV=1 - [BAIP2_HUMAN] |
| BCLAF1 | Bcl-2-associated transcription factor 1 OS=Homo sapiens GN=BCLAF1 PE=1 SV=2 - [BCLF1_HUMAN] |
| BRD2 | Bromodomain-containing protein 2 OS=Homo sapiens GN=BRD2 PE=1 SV=2 - [BRD2_HUMAN] |
| CALD1 | Caldesmon OS=Homo sapiens GN=CALD1 PE=1 SV=3 - [CALD1_HUMAN] |
| CAPRIN1 | Caprin-1 OS=Homo sapiens GN=CAPRIN1 PE=1 SV=2 - [CAPR1_HUMAN] |
| CASK | Peripheral plasma membrane protein CASK OS=Homo sapiens GN=CASK PE=1 SV=3 - [CSKP_HUMAN] |
| CAV1 | Caveolin-1 OS=Homo sapiens GN=CAV1 PE=1 SV=4 - [CAV1_HUMAN] |
| CAV2 | Caveolin-2 OS=Homo sapiens GN=CAV2 PE=1 SV=2 - [CAV2_HUMAN] |
| CAVIN2 | Serum deprivation-response protein OS=Homo sapiens GN=SDPR PE=1 SV=3 - [SDPR_HUMAN] |
| CCNY | Cyclin-Y OS=Homo sapiens GN=CCNY PE=1 SV=2 - [CCNY_HUMAN] |
| CDK16 | Cyclin-dependent kinase 16 OS=Homo sapiens GN=CDK16 PE=1 SV=1 - [CDK16_HUMAN] |
| CIT | Citron Rho-interacting kinase OS=Homo sapiens GN=CIT PE=1 SV=2 - [CTRO_HUMAN] |
| CSNK1G2 | Casein kinase I isoform gamma-2 OS=Homo sapiens GN=CSNK1G2 PE=1 SV=1 - [KC1G2_HUMAN] |
| DBN1 | Drebrin OS=Homo sapiens GN=DBN1 PE=1 SV=4 - [DREB_HUMAN] |
| DDX21 | Nucleolar RNA helicase 2 OS=Homo sapiens GN=DDX21 PE=1 SV=5 - [DDX21_HUMAN] |
| DDX39B | Spliceosome RNA helicase DDX39B OS=Homo sapiens GN=DDX39B PE=1 SV=1 - [DX39B_HUMAN] |
| DDX41 | Probable ATP-dependent RNA helicase DDX41 OS=Homo sapiens GN=DDX41 PE=1 SV=2 - [DDX41_HUMAN] |
| DDX47 | Probable ATP-dependent RNA helicase DDX47 OS=Homo sapiens GN=DDX47 PE=1 SV=1 - [DDX47_HUMAN] |
| DEF6 | Differentially expressed in FDCP 6 homolog OS=Homo sapiens GN=DEF6 PE=1 SV=1 - [DEFI6_HUMAN] |
| DEK | Protein DEK OS=Homo sapiens GN=DEK PE=1 SV=1 - [DEK_HUMAN] |
| DHX30 | Putative ATP-dependent RNA helicase DHX30 OS=Homo sapiens GN=DHX30 PE=1 SV=1 - [DHX30_HUMAN] |
| DHX36 | Probable ATP-dependent RNA helicase DHX36 OS=Homo sapiens GN=DHX36 PE=1 SV=2 - [DHX36_HUMAN] |
| DLAT | Dihydrolipoyllysine-residue acetyltransferase component of pyruvate dehydrogenase complex, mitochondrial OS=Homo sapiens GN=DLAT PE=1 SV=3 - [ODP2_HUMAN] |
| DLG1 | Disks large homolog 1 OS=Homo sapiens GN=DLG1 PE=1 SV=2 - [DLG1_HUMAN] |
| DLST | Dihydrolipoyllysine-residue succinyltransferase component of 2-oxoglutarate dehydrogenase complex, mitochondrial OS=Homo sapiens GN=DLST PE=1 SV=4 - [ODO2_HUMAN] |
| DNMT1 | DNA (cytosine-5)-methyltransferase 1 OS=Homo sapiens GN=DNMT1 PE=1 SV=2 - [DNMT1_HUMAN] |
| DNTTIP2 | Deoxynucleotidyltransferase terminal-interacting protein 2 OS=Homo sapiens GN=DNTTIP2 PE=1 SV=2 - [TDIF2_HUMAN] |
| DOCK7 | Dedicator of cytokinesis protein 7 OS=Homo sapiens GN=DOCK7 PE=1 SV=4 - [DOCK7_HUMAN] |
| DSG2 | Desmoglein-2 OS=Homo sapiens GN=DSG2 PE=1 SV=2 - [DSG2_HUMAN] |
| DST | Dystonin OS=Homo sapiens GN=DST PE=1 SV=4 - [DYST_HUMAN] |
| EFR3A | Protein EFR3 homolog A OS=Homo sapiens GN=EFR3A PE=1 SV=2 - [EFR3A_HUMAN] |
| EGFR | Epidermal growth factor receptor OS=Homo sapiens GN=EGFR PE=1 SV=2 - [EGFR_HUMAN] |
| EIF2S1 | Eukaryotic translation initiation factor 2 subunit 1 OS=Homo sapiens GN=EIF2S1 PE=1 SV=3 - [IF2A_HUMAN] |
| EIF3A | Eukaryotic translation initiation factor 3 subunit A OS=Homo sapiens GN=EIF3A PE=1 SV=1 - [EIF3A_HUMAN] |
| EIF3F | Eukaryotic translation initiation factor 3 subunit F OS=Homo sapiens GN=EIF3F PE=1 SV=1 - [EIF3F_HUMAN] |
| EIF4A2 | Eukaryotic initiation factor 4A-II OS=Homo sapiens GN=EIF4A2 PE=1 SV=2 - [IF4A2_HUMAN] |
| EIF4A3 | Eukaryotic initiation factor 4A-III OS=Homo sapiens GN=EIF4A3 PE=1 SV=4 - [IF4A3_HUMAN] |
| EIF5B | Eukaryotic translation initiation factor 5B OS=Homo sapiens GN=EIF5B PE=1 SV=4 - [IF2P_HUMAN] |
| ELAVL1 | ELAV-like protein 1 OS=Homo sapiens GN=ELAVL1 PE=1 SV=2 - [ELAV1_HUMAN] |
| EMP3 | Epithelial membrane protein 3 OS=Homo sapiens GN=EMP3 PE=2 SV=1 - [EMP3_HUMAN] |
| EPHA2 | Ephrin type-A receptor 2 OS=Homo sapiens GN=EPHA2 PE=1 SV=2 - [EPHA2_HUMAN] |
| EPS8 | Epidermal growth factor receptor kinase substrate 8 OS=Homo sapiens GN=EPS8 PE=1 SV=1 - [EPS8_HUMAN] |
| ERBIN | Protein LAP2 OS=Homo sapiens GN=ERBB2IP PE=1 SV=2 - [LAP2_HUMAN] |
| ERH | Enhancer of rudimentary homolog OS=Homo sapiens GN=ERH PE=1 SV=1 - [ERH_HUMAN] |
| ERLIN1 | Erlin-1 OS=Homo sapiens GN=ERLIN1 PE=1 SV=1 - [ERLN1_HUMAN] |
| FAM120A | Constitutive coactivator of PPAR-gamma-like protein 1 OS=Homo sapiens GN=FAM120A PE=1 SV=2 - [F120A_HUMAN] |
| FAM207A | Protein FAM207A OS=Homo sapiens GN=FAM207A PE=1 SV=2 - [F207A_HUMAN] |
| FARP2 | FERM, RhoGEF and pleckstrin domain-containing protein 2 OS=Homo sapiens GN=FARP2 PE=1 SV=3 - [FARP2_HUMAN] |
| FBL | rRNA 2'-O-methyltransferase fibrillarin OS=Homo sapiens GN=FBL PE=1 SV=2 - [FBRL_HUMAN] |
| FEM1B | Protein fem-1 homolog B OS=Homo sapiens GN=FEM1B PE=1 SV=1 - [FEM1B_HUMAN] |
| FLNA | Filamin-A OS=Homo sapiens GN=FLNA PE=1 SV=4 - [FLNA_HUMAN] |
| FLNB | Filamin-B OS=Homo sapiens GN=FLNB PE=1 SV=2 - [FLNB_HUMAN] |
| FLOT1 | Flotillin-1 OS=Homo sapiens GN=FLOT1 PE=1 SV=3 - [FLOT1_HUMAN] |
| FLOT2 | Flotillin-2 OS=Homo sapiens GN=FLOT2 PE=1 SV=2 - [FLOT2_HUMAN] |
| FMR1 | Fragile X mental retardation protein 1 OS=Homo sapiens GN=FMR1 PE=1 SV=1 - [FMR1_HUMAN] |
| FXR1 | Fragile X mental retardation syndrome-related protein 1 OS=Homo sapiens GN=FXR1 PE=1 SV=3 - [FXR1_HUMAN] |
| FXR2 | Fragile X mental retardation syndrome-related protein 2 OS=Homo sapiens GN=FXR2 PE=1 SV=2 - [FXR2_HUMAN] |
| G3BP1 | Ras GTPase-activating protein-binding protein 1 OS=Homo sapiens GN=G3BP1 PE=1 SV=1 - [G3BP1_HUMAN] |
| G3BP2 | Ras GTPase-activating protein-binding protein 2 OS=Homo sapiens GN=G3BP2 PE=1 SV=2 - [G3BP2_HUMAN] |
| GAR1 | H/ACA ribonucleoprotein complex subunit 1 OS=Homo sapiens GN=GAR1 PE=1 SV=1 - [GAR1_HUMAN] |
| GNA11 | Guanine nucleotide-binding protein subunit alpha-11 OS=Homo sapiens GN=GNA11 PE=1 SV=2 - [GNA11_HUMAN] |
| GNG12 | Guanine nucleotide-binding protein G(I)/G(S)/G(O) subunit gamma-12 OS=Homo sapiens GN=GNG12 PE=1 SV=3 - [GBG12_HUMAN] |
| GOLGA7 | Golgin subfamily A member 7 OS=Homo sapiens GN=GOLGA7 PE=1 SV=2 - [GOGA7_HUMAN] |
| GPC1 | Glypican-1 OS=Homo sapiens GN=GPC1 PE=1 SV=2 - [GPC1_HUMAN] |
| GPRC5A | Retinoic acid-induced protein 3 OS=Homo sapiens GN=GPRC5A PE=1 SV=2 - [RAI3_HUMAN] |
| GSDMA | Gasdermin-A OS=Homo sapiens GN=GSDMA PE=1 SV=4 - [GSDMA_HUMAN] |
| GTPBP4 | Nucleolar GTP-binding protein 1 OS=Homo sapiens GN=GTPBP4 PE=1 SV=3 - [NOG1_HUMAN] |
| H2AFY | Core histone macro-H2A.1 OS=Homo sapiens GN=H2AFY PE=1 SV=4 - [H2AY_HUMAN] |
| HDLBP | Vigilin OS=Homo sapiens GN=HDLBP PE=1 SV=2 - [VIGLN_HUMAN] |
| HEATR1 | HEAT repeat-containing protein 1 OS=Homo sapiens GN=HEATR1 PE=1 SV=3 - [HEAT1_HUMAN] |
| HEATR3 | HEAT repeat-containing protein 3 OS=Homo sapiens GN=HEATR3 PE=1 SV=2 - [HEAT3_HUMAN] |
| HIST1H1B | Histone H1.5 OS=Homo sapiens GN=HIST1H1B PE=1 SV=3 - [H15_HUMAN] |
| HIST1H1C | Histone H1.2 OS=Homo sapiens GN=HIST1H1C PE=1 SV=2 - [H12_HUMAN] |
| HIST1H2AH | Histone H2A type 1-H OS=Homo sapiens GN=HIST1H2AH PE=1 SV=3 - [H2A1H_HUMAN] |
| HMCES | Embryonic stem cell-specific 5-hydroxymethylcytosine-binding protein OS=Homo sapiens GN=HMCES PE=1 SV=1 - [HMCES_HUMAN] |
| HNRNPD | Heterogeneous nuclear ribonucleoprotein D0 OS=Homo sapiens GN=HNRNPD PE=1 SV=1 - [HNRPD_HUMAN] |
| HNRNPH3 | Heterogeneous nuclear ribonucleoprotein H3 OS=Homo sapiens GN=HNRNPH3 PE=1 SV=2 - [HNRH3_HUMAN] |
| HNRNPM | Heterogeneous nuclear ribonucleoprotein M OS=Homo sapiens GN=HNRNPM PE=1 SV=3 - [HNRPM_HUMAN] |
| HNRNPR | Heterogeneous nuclear ribonucleoprotein R OS=Homo sapiens GN=HNRNPR PE=1 SV=1 - [HNRPR_HUMAN] |
| HNRNPUL1 | Heterogeneous nuclear ribonucleoprotein U-like protein 1 OS=Homo sapiens GN=HNRNPUL1 PE=1 SV=2 - [HNRL1_HUMAN] |
| HRNR | Hornerin OS=Homo sapiens GN=HRNR PE=1 SV=2 - [HORN_HUMAN] |
| IGF2BP2 | Insulin-like growth factor 2 mRNA-binding protein 2 OS=Homo sapiens GN=IGF2BP2 PE=1 SV=2 - [IF2B2_HUMAN] |
| IGF2BP3 | Insulin-like growth factor 2 mRNA-binding protein 3 OS=Homo sapiens GN=IGF2BP3 PE=1 SV=2 - [IF2B3_HUMAN] |
| INF2 | Inverted formin-2 OS=Homo sapiens GN=INF2 PE=1 SV=2 - [INF2_HUMAN] |
| ITPR2 | Inositol 1,4,5-trisphosphate receptor type 2 OS=Homo sapiens GN=ITPR2 PE=1 SV=2 - [ITPR2_HUMAN] |
| ITPR3 | Inositol 1,4,5-trisphosphate receptor type 3 OS=Homo sapiens GN=ITPR3 PE=1 SV=2 - [ITPR3_HUMAN] |
| KDM1A | Lysine-specific histone demethylase 1A OS=Homo sapiens GN=KDM1A PE=1 SV=2 - [KDM1A_HUMAN] |
| KEAP1 | Kelch-like ECH-associated protein 1 OS=Homo sapiens GN=KEAP1 PE=1 SV=2 - [KEAP1_HUMAN] |
| KHDRBS1 | KH domain-containing, RNA-binding, signal transduction-associated protein 1 OS=Homo sapiens GN=KHDRBS1 PE=1 SV=1 - [KHDR1_HUMAN] |
| KIAA1671 | Uncharacterized protein KIAA1671 OS=Homo sapiens GN=KIAA1671 PE=1 SV=2 - [K1671_HUMAN] |
| KIF23 | Kinesin-like protein KIF23 OS=Homo sapiens GN=KIF23 PE=1 SV=3 - [KIF23_HUMAN] |
| KPNA3 | Importin subunit alpha-4 OS=Homo sapiens GN=KPNA3 PE=1 SV=2 - [IMA4_HUMAN] |
| LARP1 | La-related protein 1 OS=Homo sapiens GN=LARP1 PE=1 SV=2 - [LARP1_HUMAN] |
| LARP4B | La-related protein 4B OS=Homo sapiens GN=LARP4B PE=1 SV=3 - [LAR4B_HUMAN] |
| LIG3 | DNA ligase 3 OS=Homo sapiens GN=LIG3 PE=1 SV=2 - [DNLI3_HUMAN] |
| LRCH3 | Leucine-rich repeat and calponin homology domain-containing protein 3 OS=Homo sapiens GN=LRCH3 PE=1 SV=2 - [LRCH3_HUMAN] |
| LRRC59 | Leucine-rich repeat-containing protein 59 OS=Homo sapiens GN=LRRC59 PE=1 SV=1 - [LRC59_HUMAN] |
| LUZP1 | Leucine zipper protein 1 OS=Homo sapiens GN=LUZP1 PE=1 SV=2 - [LUZP1_HUMAN] |
| LYN | Tyrosine-protein kinase Lyn OS=Homo sapiens GN=LYN PE=1 SV=3 - [LYN_HUMAN] |
| MACF1 | Microtubule-actin cross-linking factor 1, isoforms 1/2/3/5 OS=Homo sapiens GN=MACF1 PE=1 SV=4 - [MACF1_HUMAN] |
| MAT2B | Methionine adenosyltransferase 2 subunit beta OS=Homo sapiens GN=MAT2B PE=1 SV=1 - [MAT2B_HUMAN] |
| MFGE8 | Lactadherin OS=Homo sapiens GN=MFGE8 PE=1 SV=2 - [MFGM_HUMAN] |
| MISP | Mitotic interactor and substrate of PLK1 OS=Homo sapiens GN=MISP PE=1 SV=1 - [MISP_HUMAN] |
| MKI67 | Antigen KI-67 OS=Homo sapiens GN=MKI67 PE=1 SV=2 - [KI67_HUMAN] |
| MPRIP | Myosin phosphatase Rho-interacting protein OS=Homo sapiens GN=MPRIP PE=1 SV=3 - [MPRIP_HUMAN] |
| MYO18A | Unconventional myosin-XVIIIa OS=Homo sapiens GN=MYO18A PE=1 SV=3 - [MY18A_HUMAN] |
| MYO19 | Unconventional myosin-XIX OS=Homo sapiens GN=MYO19 PE=2 SV=2 - [MYO19_HUMAN] |
| MYO1E | Unconventional myosin-Ie OS=Homo sapiens GN=MYO1E PE=1 SV=2 - [MYO1E_HUMAN] |
| MYO5A | Unconventional myosin-Va OS=Homo sapiens GN=MYO5A PE=1 SV=2 - [MYO5A_HUMAN] |
| MYO6 | Unconventional myosin-VI OS=Homo sapiens GN=MYO6 PE=1 SV=4 - [MYO6_HUMAN] |
| MYOF | Myoferlin OS=Homo sapiens GN=MYOF PE=1 SV=1 - [MYOF_HUMAN] |
| NASP | Nuclear autoantigenic sperm protein OS=Homo sapiens GN=NASP PE=1 SV=2 - [NASP_HUMAN] |
| NF2 | Merlin OS=Homo sapiens GN=NF2 PE=1 SV=1 - [MERL_HUMAN] |
| NIFK | MKI67 FHA domain-interacting nucleolar phosphoprotein OS=Homo sapiens GN=NIFK PE=1 SV=1 - [MK67I_HUMAN] |
| NME2 | Nucleoside diphosphate kinase B OS=Homo sapiens GN=NME2 PE=1 SV=1 - [NDKB_HUMAN] |
| NOC2L | Nucleolar complex protein 2 homolog OS=Homo sapiens GN=NOC2L PE=1 SV=4 - [NOC2L_HUMAN] |
| NOL10 | Nucleolar protein 10 OS=Homo sapiens GN=NOL10 PE=1 SV=1 - [NOL10_HUMAN] |
| NOL6 | Nucleolar protein 6 OS=Homo sapiens GN=NOL6 PE=1 SV=2 - [NOL6_HUMAN] |
| NOLC1 | Nucleolar and coiled-body phosphoprotein 1 OS=Homo sapiens GN=NOLC1 PE=1 SV=2 - [NOLC1_HUMAN] |
| NOP2 | Putative ribosomal RNA methyltransferase NOP2 OS=Homo sapiens GN=NOP2 PE=1 SV=2 - [NOP2_HUMAN] |
| NOP56 | Nucleolar protein 56 OS=Homo sapiens GN=NOP56 PE=1 SV=4 - [NOP56_HUMAN] |
| NUFIP2 | Nuclear fragile X mental retardation-interacting protein 2 OS=Homo sapiens GN=NUFIP2 PE=1 SV=1 - [NUFP2_HUMAN] |
| NUMA1 | Nuclear mitotic apparatus protein 1 OS=Homo sapiens GN=NUMA1 PE=1 SV=2 - [NUMA1_HUMAN] |
| NUP160 | Nuclear pore complex protein Nup160 OS=Homo sapiens GN=NUP160 PE=1 SV=3 - [NU160_HUMAN] |
| NUP205 | Nuclear pore complex protein Nup205 OS=Homo sapiens GN=NUP205 PE=1 SV=3 - [NU205_HUMAN] |
| NUP35 | Nucleoporin NUP53 OS=Homo sapiens GN=NUP35 PE=1 SV=1 - [NUP53_HUMAN] |
| NUP98 | Nuclear pore complex protein Nup98-Nup96 OS=Homo sapiens GN=NUP98 PE=1 SV=4 - [NUP98_HUMAN] |
| NUPR1 | Nuclear protein 1 OS=Homo sapiens GN=NUPR1 PE=1 SV=1 - [NUPR1_HUMAN] |
| PABPC4 | Polyadenylate-binding protein 4 OS=Homo sapiens GN=PABPC4 PE=1 SV=1 - [PABP4_HUMAN] |
| PACSIN3 | Protein kinase C and casein kinase substrate in neurons protein 3 OS=Homo sapiens GN=PACSIN3 PE=1 SV=2 - [PACN3_HUMAN] |
| PAK1IP1 | p21-activated protein kinase-interacting protein 1 OS=Homo sapiens GN=PAK1IP1 PE=1 SV=2 - [PK1IP_HUMAN] |
| PALM | Paralemmin-1 OS=Homo sapiens GN=PALM PE=1 SV=2 - [PALM_HUMAN] |
| PCBP3 | Poly(rC)-binding protein 3 OS=Homo sapiens GN=PCBP3 PE=2 SV=2 - [PCBP3_HUMAN] |
| PDCD11 | Protein RRP5 homolog OS=Homo sapiens GN=PDCD11 PE=1 SV=3 - [RRP5_HUMAN] |
| PI4KA | Phosphatidylinositol 4-kinase alpha OS=Homo sapiens GN=PI4KA PE=1 SV=4 - [PI4KA_HUMAN] |
| PKP4 | Plakophilin-4 OS=Homo sapiens GN=PKP4 PE=1 SV=2 - [PKP4_HUMAN] |
| PLD2 | Phospholipase D2 OS=Homo sapiens GN=PLD2 PE=1 SV=2 - [PLD2_HUMAN] |
| PLEC | Plectin OS=Homo sapiens GN=PLEC PE=1 SV=3 - [PLEC_HUMAN] |
| PLS1 | Plastin-1 OS=Homo sapiens GN=PLS1 PE=1 SV=2 - [PLSI_HUMAN] |
| PNN | Pinin OS=Homo sapiens GN=PNN PE=1 SV=4 - [PININ_HUMAN] |
| PPP1R12A | Protein phosphatase 1 regulatory subunit 12A OS=Homo sapiens GN=PPP1R12A PE=1 SV=1 - [MYPT1_HUMAN] |
| PPP1R18 | Phostensin OS=Homo sapiens GN=PPP1R18 PE=1 SV=1 - [PPR18_HUMAN] |
| PPP1R9B | Neurabin-2 OS=Homo sapiens GN=PPP1R9B PE=1 SV=2 - [NEB2_HUMAN] |
| PPP2R5C | Serine/threonine-protein phosphatase 2A 56 kDa regulatory subunit gamma isoform OS=Homo sapiens GN=PPP2R5C PE=1 SV=3 - [2A5G_HUMAN] |
| PRNP | Major prion protein OS=Homo sapiens GN=PRNP PE=1 SV=1 - [PRIO_HUMAN] |
| PRPF19 | Pre-mRNA-processing factor 19 OS=Homo sapiens GN=PRPF19 PE=1 SV=1 - [PRP19_HUMAN] |
| PRPF4 | U4/U6 small nuclear ribonucleoprotein Prp4 OS=Homo sapiens GN=PRPF4 PE=1 SV=2 - [PRP4_HUMAN] |
| PRPF6 | Pre-mRNA-processing factor 6 OS=Homo sapiens GN=PRPF6 PE=1 SV=1 - [PRP6_HUMAN] |
| PRPF8 | Pre-mRNA-processing-splicing factor 8 OS=Homo sapiens GN=PRPF8 PE=1 SV=2 - [PRP8_HUMAN] |
| PRRC2C | Protein PRRC2C OS=Homo sapiens GN=PRRC2C PE=1 SV=4 - [PRC2C_HUMAN] |
| PWP2 | Periodic tryptophan protein 2 homolog OS=Homo sapiens GN=PWP2 PE=1 SV=2 - [PWP2_HUMAN] |
| RAB35 | Ras-related protein Rab-35 OS=Homo sapiens GN=RAB35 PE=1 SV=1 - [RAB35_HUMAN] |
| RACGAP1 | Rac GTPase-activating protein 1 OS=Homo sapiens GN=RACGAP1 PE=1 SV=1 - [RGAP1_HUMAN] |
| RANBP2 | E3 SUMO-protein ligase RanBP2 OS=Homo sapiens GN=RANBP2 PE=1 SV=2 - [RBP2_HUMAN] |
| RCL1 | RNA 3'-terminal phosphate cyclase-like protein OS=Homo sapiens GN=RCL1 PE=1 SV=3 - [RCL1_HUMAN] |
| RDX | Radixin OS=Homo sapiens GN=RDX PE=1 SV=1 - [RADI_HUMAN] |
| RPA1 | Replication protein A 70 kDa DNA-binding subunit OS=Homo sapiens GN=RPA1 PE=1 SV=2 - [RFA1_HUMAN] |
| RPL10A | 60S ribosomal protein L10a OS=Homo sapiens GN=RPL10A PE=1 SV=2 - [RL10A_HUMAN] |
| RPL13 | 60S ribosomal protein L13 OS=Homo sapiens GN=RPL13 PE=1 SV=4 - [RL13_HUMAN] |
| RPL13A | 60S ribosomal protein L13a OS=Homo sapiens GN=RPL13A PE=1 SV=2 - [RL13A_HUMAN] |
| RPL15 | 60S ribosomal protein L15 OS=Homo sapiens GN=RPL15 PE=1 SV=2 - [RL15_HUMAN] |
| RPL18A | 60S ribosomal protein L18a OS=Homo sapiens GN=RPL18A PE=1 SV=2 - [RL18A_HUMAN] |
| RPL19 | 60S ribosomal protein L19 OS=Homo sapiens GN=RPL19 PE=1 SV=1 - [RL19_HUMAN] |
| RPL24 | 60S ribosomal protein L24 OS=Homo sapiens GN=RPL24 PE=1 SV=1 - [RL24_HUMAN] |
| RPL26 | 60S ribosomal protein L26 OS=Homo sapiens GN=RPL26 PE=1 SV=1 - [RL26_HUMAN] |
| RPL27 | 60S ribosomal protein L27 OS=Homo sapiens GN=RPL27 PE=1 SV=2 - [RL27_HUMAN] |
| RPL3 | 60S ribosomal protein L3 OS=Homo sapiens GN=RPL3 PE=1 SV=2 - [RL3_HUMAN] |
| RPL31 | 60S ribosomal protein L31 OS=Homo sapiens GN=RPL31 PE=1 SV=1 - [RL31_HUMAN] |
| RPL35A | 60S ribosomal protein L35a OS=Homo sapiens GN=RPL35A PE=1 SV=2 - [RL35A_HUMAN] |
| RPL4 | 60S ribosomal protein L4 OS=Homo sapiens GN=RPL4 PE=1 SV=5 - [RL4_HUMAN] |
| RPL5 | 60S ribosomal protein L5 OS=Homo sapiens GN=RPL5 PE=1 SV=3 - [RL5_HUMAN] |
| RPL6 | 60S ribosomal protein L6 OS=Homo sapiens GN=RPL6 PE=1 SV=3 - [RL6_HUMAN] |
| RPL7 | 60S ribosomal protein L7 OS=Homo sapiens GN=RPL7 PE=1 SV=1 - [RL7_HUMAN] |
| RPL7A | 60S ribosomal protein L7a OS=Homo sapiens GN=RPL7A PE=1 SV=2 - [RL7A_HUMAN] |
| RPL8 | 60S ribosomal protein L8 OS=Homo sapiens GN=RPL8 PE=1 SV=2 - [RL8_HUMAN] |
| RPL9 | 60S ribosomal protein L9 OS=Homo sapiens GN=RPL9 PE=1 SV=1 - [RL9_HUMAN] |
| RPP30 | Ribonuclease P protein subunit p30 OS=Homo sapiens GN=RPP30 PE=1 SV=1 - [RPP30_HUMAN] |
| RPS28 | 40S ribosomal protein S28 OS=Homo sapiens GN=RPS28 PE=1 SV=1 - [RS28_HUMAN] |
| RPS6 | 40S ribosomal protein S6 OS=Homo sapiens GN=RPS6 PE=1 SV=1 - [RS6_HUMAN] |
| RPS9 | 40S ribosomal protein S9 OS=Homo sapiens GN=RPS9 PE=1 SV=3 - [RS9_HUMAN] |
| RRAS2 | Ras-related protein R-Ras2 OS=Homo sapiens GN=RRAS2 PE=1 SV=1 - [RRAS2_HUMAN] |
| RRP9 | U3 small nucleolar RNA-interacting protein 2 OS=Homo sapiens GN=RRP9 PE=1 SV=1 - [U3IP2_HUMAN] |
| RSPRY1 | RING finger and SPRY domain-containing protein 1 OS=Homo sapiens GN=RSPRY1 PE=2 SV=1 - [RSPRY_HUMAN] |
| RTCB | tRNA-splicing ligase RtcB homolog OS=Homo sapiens GN=RTCB PE=1 SV=1 - [RTCB_HUMAN] |
| S100A8 | Protein S100-A8 OS=Homo sapiens GN=S100A8 PE=1 SV=1 - [S10A8_HUMAN] |
| S100A9 | Protein S100-A9 OS=Homo sapiens GN=S100A9 PE=1 SV=1 - [S10A9_HUMAN] |
| SAFB | Scaffold attachment factor B1 OS=Homo sapiens GN=SAFB PE=1 SV=4 - [SAFB1_HUMAN] |
| SAFB2 | Scaffold attachment factor B2 OS=Homo sapiens GN=SAFB2 PE=1 SV=1 - [SAFB2_HUMAN] |
| SCRIB | Protein scribble homolog OS=Homo sapiens GN=SCRIB PE=1 SV=4 - [SCRIB_HUMAN] |
| SEMA3A | Semaphorin-3A OS=Homo sapiens GN=SEMA3A PE=1 SV=1 - [SEM3A_HUMAN] |
| SF3A3 | Splicing factor 3A subunit 3 OS=Homo sapiens GN=SF3A3 PE=1 SV=1 - [SF3A3_HUMAN] |
| SF3B1 | Splicing factor 3B subunit 1 OS=Homo sapiens GN=SF3B1 PE=1 SV=3 - [SF3B1_HUMAN] |
| SF3B2 | Splicing factor 3B subunit 2 OS=Homo sapiens GN=SF3B2 PE=1 SV=2 - [SF3B2_HUMAN] |
| SF3B6 | Pre-mRNA branch site protein p14 OS=Homo sapiens GN=SF3B14 PE=1 SV=1 - [PM14_HUMAN] |
| SFPQ | Splicing factor, proline- and glutamine-rich OS=Homo sapiens GN=SFPQ PE=1 SV=2 - [SFPQ_HUMAN] |
| SIPA1L3 | Signal-induced proliferation-associated 1-like protein 3 OS=Homo sapiens GN=SIPA1L3 PE=1 SV=3 - [SI1L3_HUMAN] |
| SMCHD1 | Structural maintenance of chromosomes flexible hinge domain-containing protein 1 OS=Homo sapiens GN=SMCHD1 PE=1 SV=2 - [SMHD1_HUMAN] |
| SND1 | Staphylococcal nuclease domain-containing protein 1 OS=Homo sapiens GN=SND1 PE=1 SV=1 - [SND1_HUMAN] |
| SNRPB | Small nuclear ribonucleoprotein-associated proteins B and B' OS=Homo sapiens GN=SNRPB PE=1 SV=2 - [RSMB_HUMAN] |
| SNRPB2 | U2 small nuclear ribonucleoprotein B'' OS=Homo sapiens GN=SNRPB2 PE=1 SV=1 - [RU2B_HUMAN] |
| SNRPD2 | Small nuclear ribonucleoprotein Sm D2 OS=Homo sapiens GN=SNRPD2 PE=1 SV=1 - [SMD2_HUMAN] |
| SNRPD3 | Small nuclear ribonucleoprotein Sm D3 OS=Homo sapiens GN=SNRPD3 PE=1 SV=1 - [SMD3_HUMAN] |
| SNX1 | Sorting nexin-1 OS=Homo sapiens GN=SNX1 PE=1 SV=3 - [SNX1_HUMAN] |
| SPATS2L | SPATS2-like protein OS=Homo sapiens GN=SPATS2L PE=1 SV=2 - [SPS2L_HUMAN] |
| SPECC1 | Cytospin-B OS=Homo sapiens GN=SPECC1 PE=1 SV=1 - [CYTSB_HUMAN] |
| SPECC1L | Cytospin-A OS=Homo sapiens GN=SPECC1L PE=1 SV=2 - [CYTSA_HUMAN] |
| SPTAN1 | Spectrin alpha chain, non-erythrocytic 1 OS=Homo sapiens GN=SPTAN1 PE=1 SV=3 - [SPTN1_HUMAN] |
| SPTBN1 | Spectrin beta chain, non-erythrocytic 1 OS=Homo sapiens GN=SPTBN1 PE=1 SV=2 - [SPTB2_HUMAN] |
| SQSTM1 | Sequestosome-1 OS=Homo sapiens GN=SQSTM1 PE=1 SV=1 - [SQSTM_HUMAN] |
| SRGAP2 | SLIT-ROBO Rho GTPase-activating protein 2 OS=Homo sapiens GN=SRGAP2 PE=1 SV=3 - [SRGP2_HUMAN] |
| SRSF1 | Serine/arginine-rich splicing factor 1 OS=Homo sapiens GN=SRSF1 PE=1 SV=2 - [SRSF1_HUMAN] |
| SRSF7 | Serine/arginine-rich splicing factor 7 OS=Homo sapiens GN=SRSF7 PE=1 SV=1 - [SRSF7_HUMAN] |
| SSFA2 | Sperm-specific antigen 2 OS=Homo sapiens GN=SSFA2 PE=1 SV=3 - [SSFA2_HUMAN] |
| SSH2 | Protein phosphatase Slingshot homolog 2 OS=Homo sapiens GN=SSH2 PE=1 SV=1 - [SSH2_HUMAN] |
| SUN1 | SUN domain-containing protein 1 OS=Homo sapiens GN=SUN1 PE=1 SV=3 - [SUN1_HUMAN] |
| SVIL | Supervillin OS=Homo sapiens GN=SVIL PE=1 SV=2 - [SVIL_HUMAN] |
| SYNCRIP | Heterogeneous nuclear ribonucleoprotein Q OS=Homo sapiens GN=SYNCRIP PE=1 SV=2 - [HNRPQ_HUMAN] |
| TBL3 | Transducin beta-like protein 3 OS=Homo sapiens GN=TBL3 PE=1 SV=2 - [TBL3_HUMAN] |
| THOC3 | THO complex subunit 3 OS=Homo sapiens GN=THOC3 PE=1 SV=1 - [THOC3_HUMAN] |
| THRAP3 | Thyroid hormone receptor-associated protein 3 OS=Homo sapiens GN=THRAP3 PE=1 SV=2 - [TR150_HUMAN] |
| TJP1 | Tight junction protein ZO-1 OS=Homo sapiens GN=TJP1 PE=1 SV=3 - [ZO1_HUMAN] |
| TMOD3 | Tropomodulin-3 OS=Homo sapiens GN=TMOD3 PE=1 SV=1 - [TMOD3_HUMAN] |
| TOP1 | DNA topoisomerase 1 OS=Homo sapiens GN=TOP1 PE=1 SV=2 - [TOP1_HUMAN] |
| TOP2A | DNA topoisomerase 2-alpha OS=Homo sapiens GN=TOP2A PE=1 SV=3 - [TOP2A_HUMAN] |
| TOP3A | DNA topoisomerase 3-alpha OS=Homo sapiens GN=TOP3A PE=1 SV=1 - [TOP3A_HUMAN] |
| TPR | Nucleoprotein TPR OS=Homo sapiens GN=TPR PE=1 SV=3 - [TPR_HUMAN] |
| TPRN | Taperin OS=Homo sapiens GN=TPRN PE=1 SV=2 - [TPRN_HUMAN] |
| TRA2B | Transformer-2 protein homolog beta OS=Homo sapiens GN=TRA2B PE=1 SV=1 - [TRA2B_HUMAN] |
| TUBA1A | Tubulin alpha-1A chain OS=Homo sapiens GN=TUBA1A PE=1 SV=1 - [TBA1A_HUMAN] |
| TWF1 | Twinfilin-1 OS=Homo sapiens GN=TWF1 PE=1 SV=3 - [TWF1_HUMAN] |
| UACA | Uveal autoantigen with coiled-coil domains and ankyrin repeats OS=Homo sapiens GN=UACA PE=1 SV=2 - [UACA_HUMAN] |
| UBE2O | Ubiquitin-conjugating enzyme E2 O OS=Homo sapiens GN=UBE2O PE=1 SV=3 - [UBE2O_HUMAN] |
| UBTF | Nucleolar transcription factor 1 OS=Homo sapiens GN=UBTF PE=1 SV=1 - [UBF1_HUMAN] |
| UPF1 | Regulator of nonsense transcripts 1 OS=Homo sapiens GN=UPF1 PE=1 SV=2 - [RENT1_HUMAN] |
| USP6NL | USP6 N-terminal-like protein OS=Homo sapiens GN=USP6NL PE=1 SV=3 - [US6NL_HUMAN] |
| UTP14A | U3 small nucleolar RNA-associated protein 14 homolog A OS=Homo sapiens GN=UTP14A PE=1 SV=1 - [UT14A_HUMAN] |
| UTP4 | Cirhin OS=Homo sapiens GN=CIRH1A PE=1 SV=1 - [CIR1A_HUMAN] |
| VRK2 | Serine/threonine-protein kinase VRK2 OS=Homo sapiens GN=VRK2 PE=1 SV=3 - [VRK2_HUMAN] |
| WASF2 | Wiskott-Aldrich syndrome protein family member 2 OS=Homo sapiens GN=WASF2 PE=1 SV=3 - [WASF2_HUMAN] |
| WDR1 | WD repeat-containing protein 1 OS=Homo sapiens GN=WDR1 PE=1 SV=4 - [WDR1_HUMAN] |
| WDR12 | Ribosome biogenesis protein WDR12 OS=Homo sapiens GN=WDR12 PE=1 SV=2 - [WDR12_HUMAN] |
| WDR3 | WD repeat-containing protein 3 OS=Homo sapiens GN=WDR3 PE=1 SV=1 - [WDR3_HUMAN] |
| WDR45B | WD repeat domain phosphoinositide-interacting protein 3 OS=Homo sapiens GN=WDR45B PE=2 SV=2 - [WIPI3_HUMAN] |
| WDR75 | WD repeat-containing protein 75 OS=Homo sapiens GN=WDR75 PE=1 SV=1 - [WDR75_HUMAN] |
| XRN2 | 5'-3' exoribonuclease 2 OS=Homo sapiens GN=XRN2 PE=1 SV=1 - [XRN2_HUMAN] |
| YBX1 | Nuclease-sensitive element-binding protein 1 OS=Homo sapiens GN=YBX1 PE=1 SV=3 - [YBOX1_HUMAN] |
| YTHDF2 | YTH domain family protein 2 OS=Homo sapiens GN=YTHDF2 PE=1 SV=2 - [YTHD2_HUMAN] |
| ZC3H14 | Zinc finger CCCH domain-containing protein 14 OS=Homo sapiens GN=ZC3H14 PE=1 SV=1 - [ZC3HE_HUMAN] |
| ZDHHC5 | Palmitoyltransferase ZDHHC5 OS=Homo sapiens GN=ZDHHC5 PE=1 SV=2 - [ZDHC5_HUMAN] |
| ZFR | Zinc finger RNA-binding protein OS=Homo sapiens GN=ZFR PE=1 SV=2 - [ZFR_HUMAN] |
| ZNF326 | DBIRD complex subunit ZNF326 OS=Homo sapiens GN=ZNF326 PE=1 SV=2 - [ZN326_HUMAN] |
