# Supplementary figures and images for: Targeting NUPR1-dependent stress granules formation to induce synthetic lethality in KrasG12D-driven tumors (part 1 of 3)
Source: EMBO Mol Med. 2024 Feb 15;16(3):4. doi: 10.1038/s44321-024-00032-2 (PMC10940650; doi:10.1038/s44321-024-00032-2)

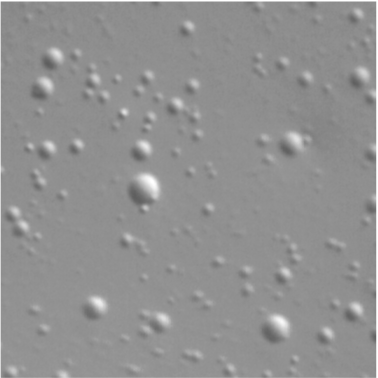

Supplement: Supplementary file 9 — Source Data Fig. 1 [file 44321_2024_32_MOESM9_ESM.zip › Figure 1/Figure 1B rNUPR1wt.tif]

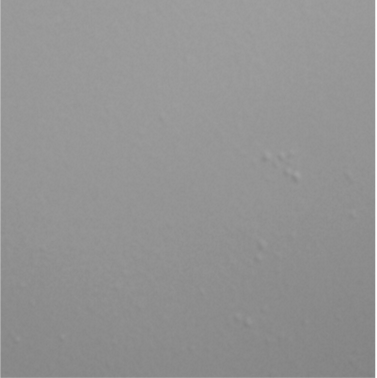

Supplement: Supplementary file 9 — Source Data Fig. 1 [file 44321_2024_32_MOESM9_ESM.zip › Figure 1/Figure 1B rNUPR1T68Qt.tif]

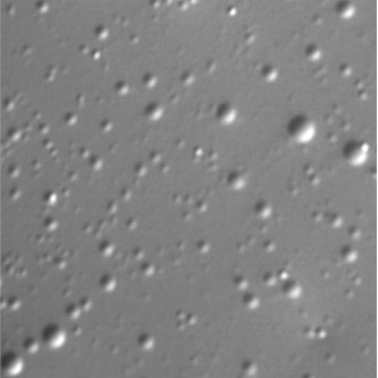

Supplement: Supplementary file 9 — Source Data Fig. 1 [file 44321_2024_32_MOESM9_ESM.zip › Figure 1/Figure 1C rNUPR1wt.tif]

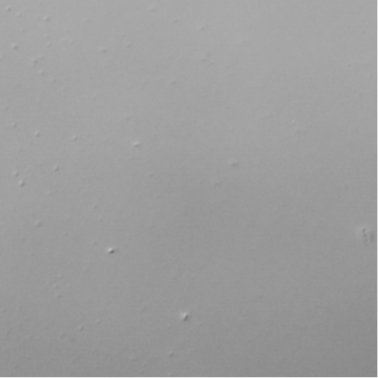

Supplement: Supplementary file 9 — Source Data Fig. 1 [file 44321_2024_32_MOESM9_ESM.zip › Figure 1/Figure 1A 0 ┬╡M.tif]

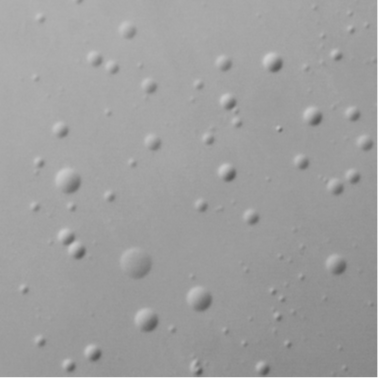

Supplement: Supplementary file 9 — Source Data Fig. 1 [file 44321_2024_32_MOESM9_ESM.zip › Figure 1/Figure 1A 25 ┬╡M.tif]

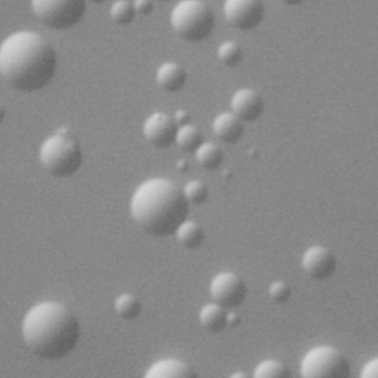

Supplement: Supplementary file 9 — Source Data Fig. 1 [file 44321_2024_32_MOESM9_ESM.zip › Figure 1/Figure 1D rNUPR1wt+PAR.tif]

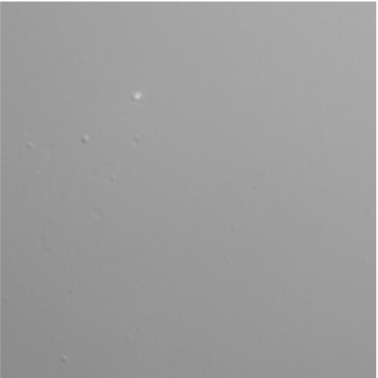

Supplement: Supplementary file 9 — Source Data Fig. 1 [file 44321_2024_32_MOESM9_ESM.zip › Figure 1/Figure 1B rNUPR1T68Q.A33Q.tif]

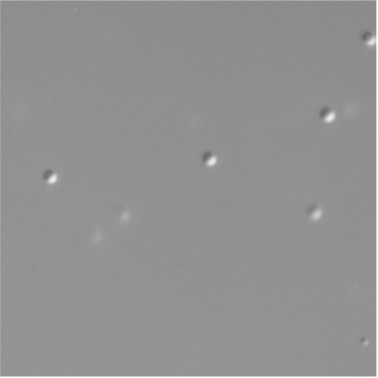

Supplement: Supplementary file 9 — Source Data Fig. 1 [file 44321_2024_32_MOESM9_ESM.zip › Figure 1/Figure 1C rNUPR1wt +ZZW-115.tif]

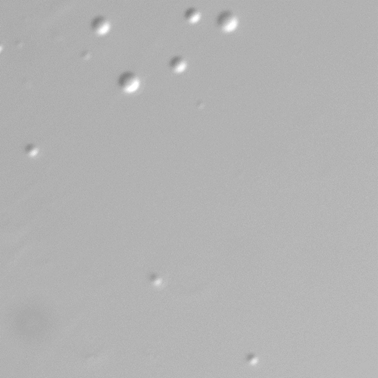

Supplement: Supplementary file 9 — Source Data Fig. 1 [file 44321_2024_32_MOESM9_ESM.zip › Figure 1/Figure 1E rNUPR1wt+RNA+ZZW-115.tif]

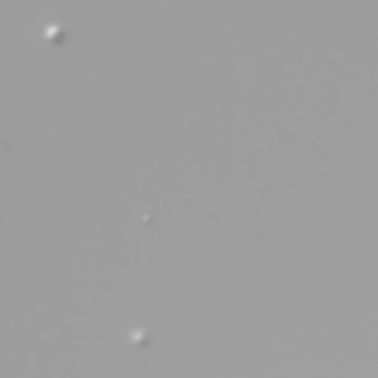

Supplement: Supplementary file 9 — Source Data Fig. 1 [file 44321_2024_32_MOESM9_ESM.zip › Figure 1/Figure 1D rNUPR1wt+PAR+ZZW-115.tif]

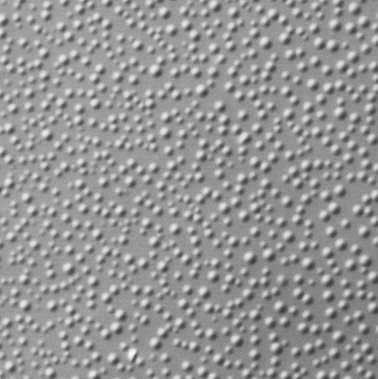

Supplement: Supplementary file 9 — Source Data Fig. 1 [file 44321_2024_32_MOESM9_ESM.zip › Figure 1/Figure 1A100 ┬╡M.tif]

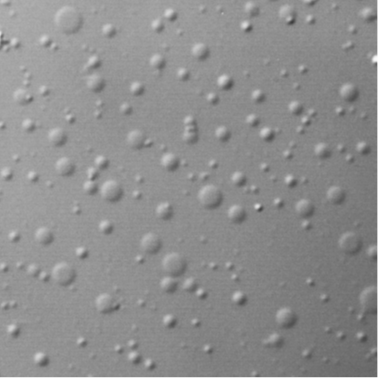

Supplement: Supplementary file 9 — Source Data Fig. 1 [file 44321_2024_32_MOESM9_ESM.zip › Figure 1/Figure 1A 50 ┬╡M.tif]

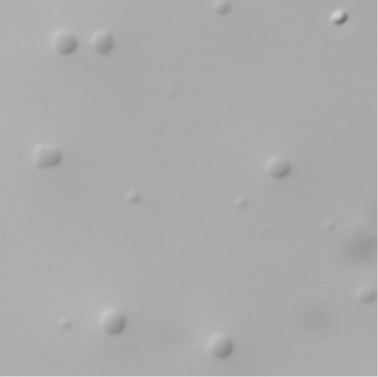

Supplement: Supplementary file 9 — Source Data Fig. 1 [file 44321_2024_32_MOESM9_ESM.zip › Figure 1/Figure 1F rNUPR1wt+ 100 ng.┬╡l RNA.tif]

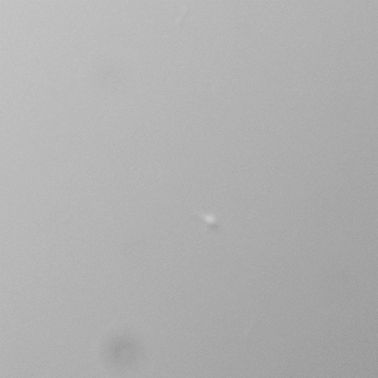

Supplement: Supplementary file 9 — Source Data Fig. 1 [file 44321_2024_32_MOESM9_ESM.zip › Figure 1/Figure 1E rNUPR1A33Q.T68Q+RNA.tif]

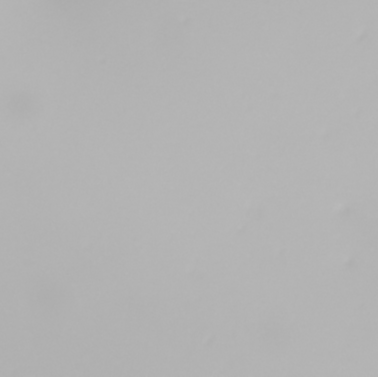

Supplement: Supplementary file 9 — Source Data Fig. 1 [file 44321_2024_32_MOESM9_ESM.zip › Figure 1/Figure 1F 200 ng ul RNA.tif]

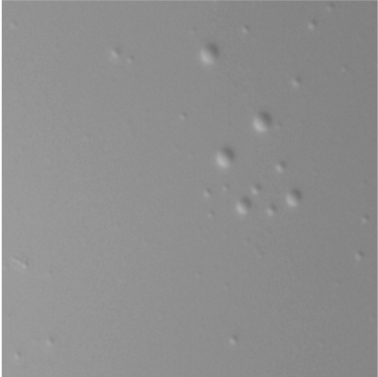

Supplement: Supplementary file 9 — Source Data Fig. 1 [file 44321_2024_32_MOESM9_ESM.zip › Figure 1/Figure 1B rNUPR1A33Q.tif]

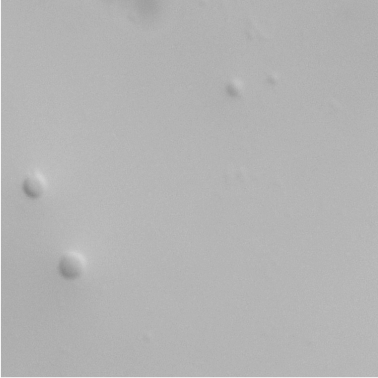

Supplement: Supplementary file 9 — Source Data Fig. 1 [file 44321_2024_32_MOESM9_ESM.zip › Figure 1/Figure 1F rNUPR1wt+ 50 ng.┬╡l RNA.tif]

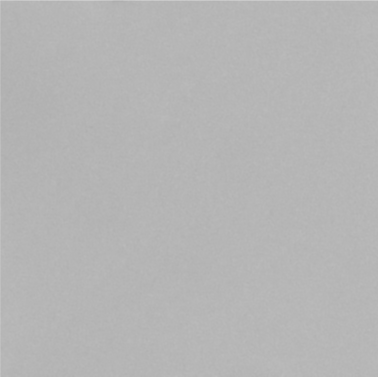

Supplement: Supplementary file 9 — Source Data Fig. 1 [file 44321_2024_32_MOESM9_ESM.zip › Figure 1/Figure 1F rNUPR1wt.tif]

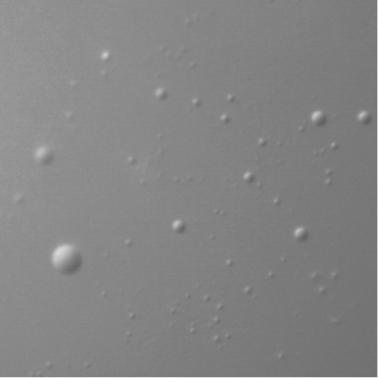

Supplement: Supplementary file 9 — Source Data Fig. 1 [file 44321_2024_32_MOESM9_ESM.zip › Figure 1/Figure 1A 5 ┬╡M.tif]

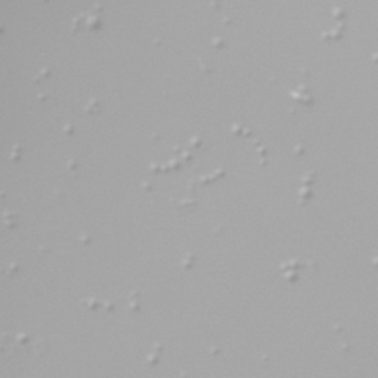

Supplement: Supplementary file 9 — Source Data Fig. 1 [file 44321_2024_32_MOESM9_ESM.zip › Figure 1/Figure 1D rNUPR1A33Q+PAR.tif]

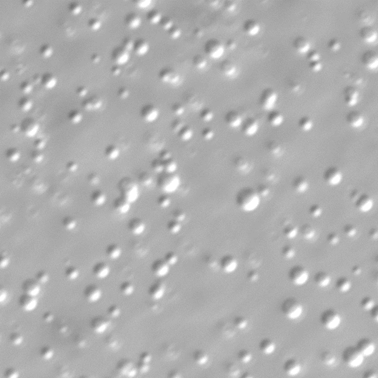

Supplement: Supplementary file 9 — Source Data Fig. 1 [file 44321_2024_32_MOESM9_ESM.zip › Figure 1/Figure 1E rNUPR1wt+RNA.tif]

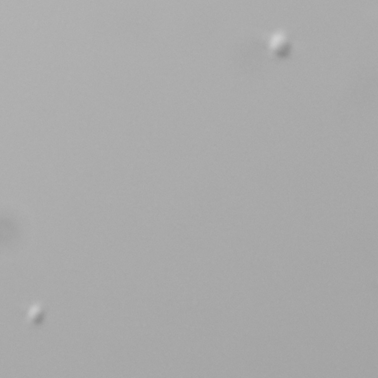

Supplement: Supplementary file 9 — Source Data Fig. 1 [file 44321_2024_32_MOESM9_ESM.zip › Figure 1/Figure 1E rNUPR1T68Q+RNA.tif]

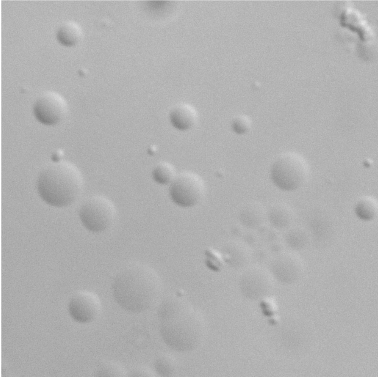

Supplement: Supplementary file 9 — Source Data Fig. 1 [file 44321_2024_32_MOESM9_ESM.zip › Figure 1/Figure 1F rNUPR1wt+ 200 ng.┬╡l RNA.tif]

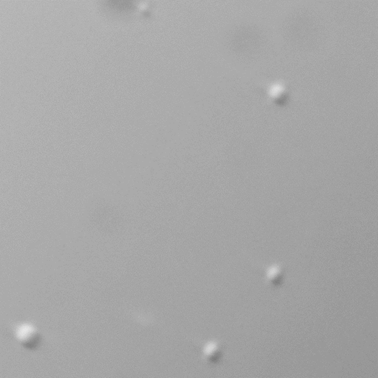

Supplement: Supplementary file 9 — Source Data Fig. 1 [file 44321_2024_32_MOESM9_ESM.zip › Figure 1/Figure 1E rNUPR1A33Q+RNA.tif]

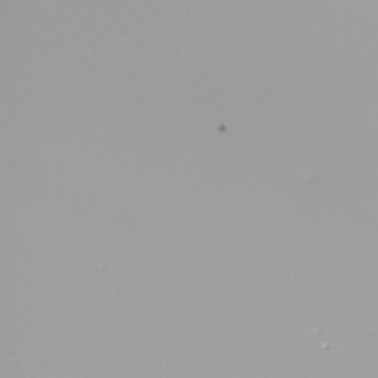

Supplement: Supplementary file 9 — Source Data Fig. 1 [file 44321_2024_32_MOESM9_ESM.zip › Figure 1/Figure 1D rNUPR1A33Q.T68Q+PAR.tif]

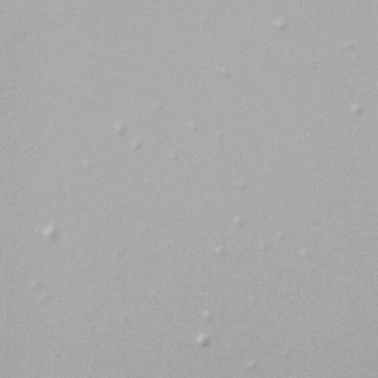

Supplement: Supplementary file 9 — Source Data Fig. 1 [file 44321_2024_32_MOESM9_ESM.zip › Figure 1/Figure 1D rNUPR1T68Q+PAR.tif]

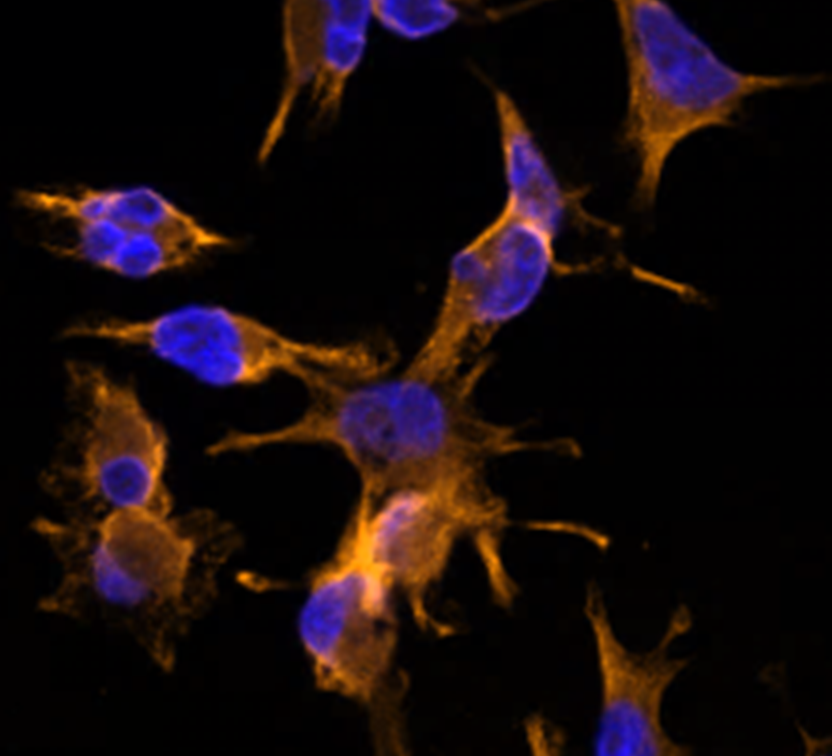

Supplement: Supplementary file 10 — Source Data Fig. 2 [file 44321_2024_32_MOESM10_ESM.zip › Figure 2/Figure 2C siNUPR1.tif]

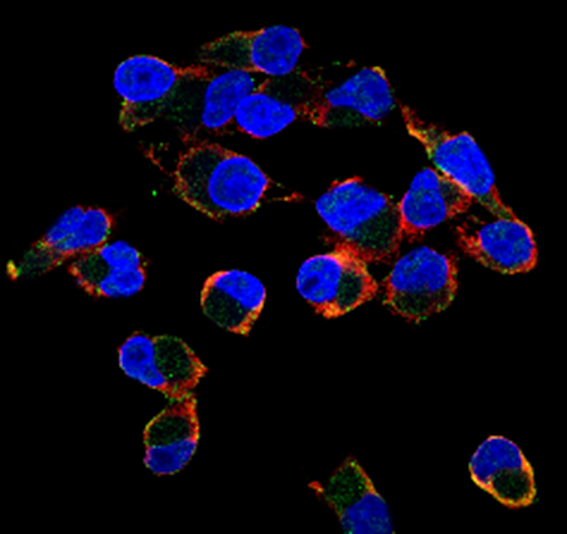

Supplement: Supplementary file 10 — Source Data Fig. 2 [file 44321_2024_32_MOESM10_ESM.zip › Figure 2/Figure 2D Merge ZZW-115.tif]

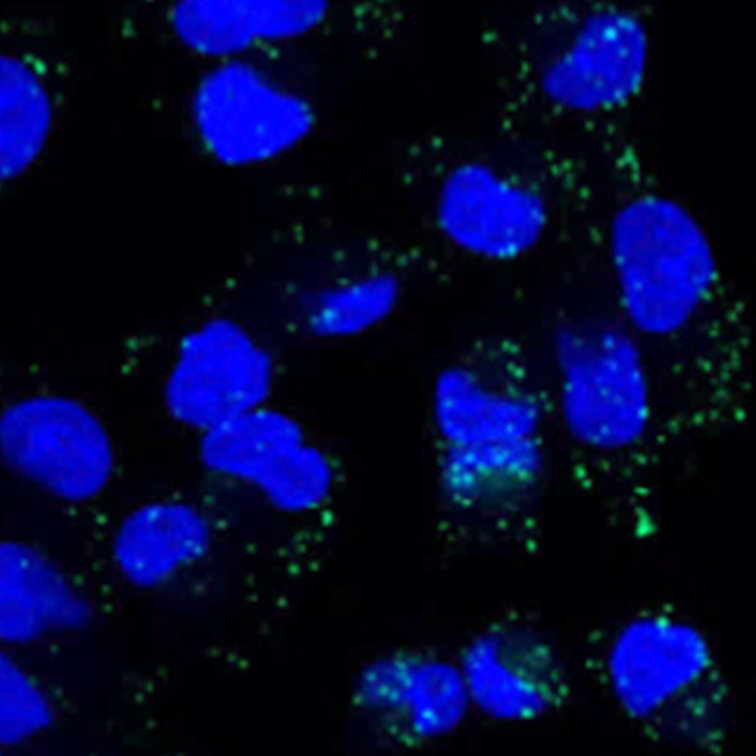

Supplement: Supplementary file 10 — Source Data Fig. 2 [file 44321_2024_32_MOESM10_ESM.zip › Figure 2/Figure 2B Ar.tif]

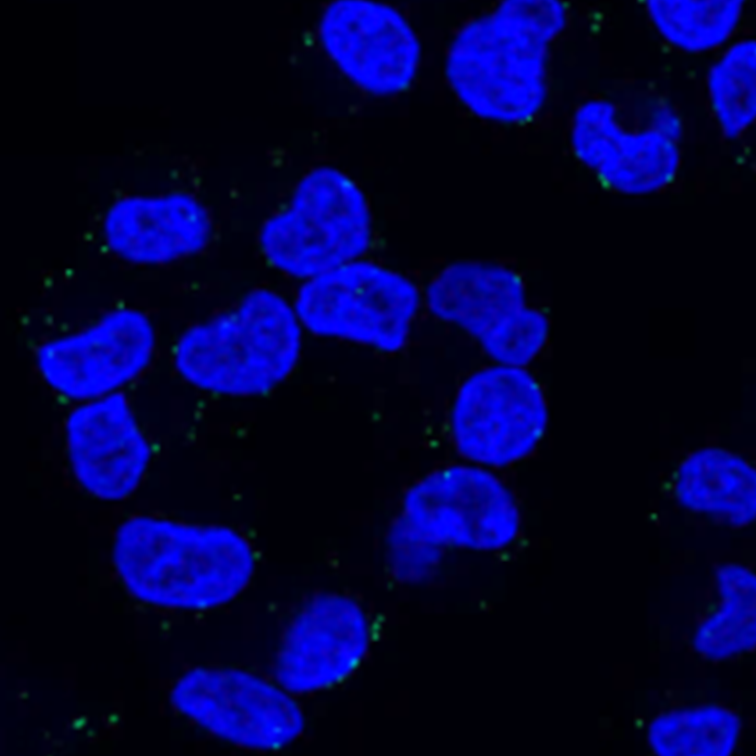

Supplement: Supplementary file 10 — Source Data Fig. 2 [file 44321_2024_32_MOESM10_ESM.zip › Figure 2/Figure 2B ZZW115 Ar.tif]

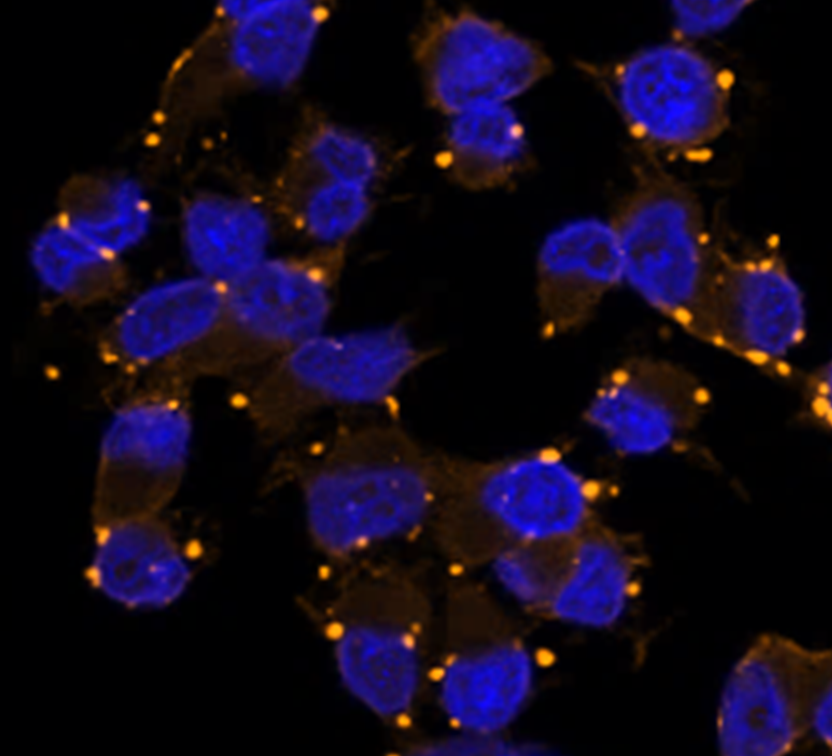

Supplement: Supplementary file 10 — Source Data Fig. 2 [file 44321_2024_32_MOESM10_ESM.zip › Figure 2/Figure 2C Ar sicontrol.tif]

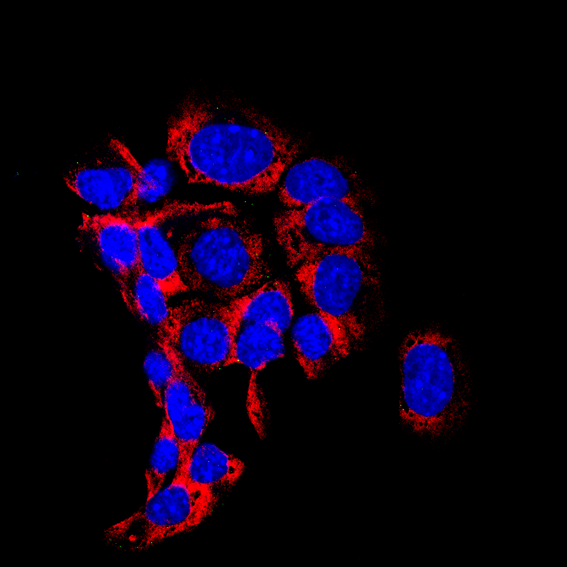

Supplement: Supplementary file 10 — Source Data Fig. 2 [file 44321_2024_32_MOESM10_ESM.zip › Figure 2/Figure 2E Merge KO Vehicle.tif]

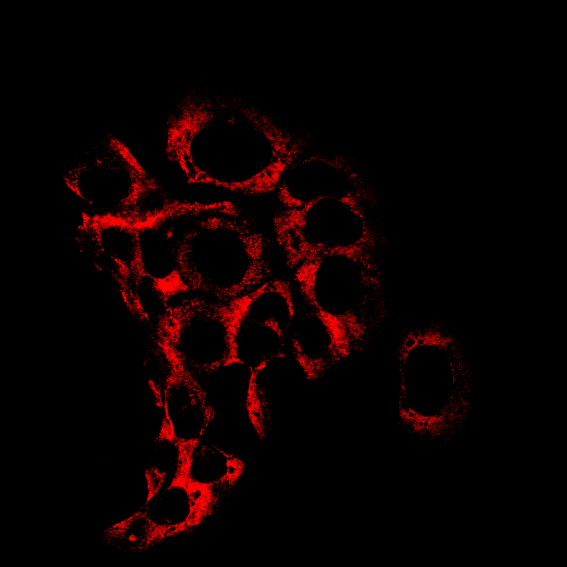

Supplement: Supplementary file 10 — Source Data Fig. 2 [file 44321_2024_32_MOESM10_ESM.zip › Figure 2/Figure 2E G3BP1 KO Vehicle.tif]

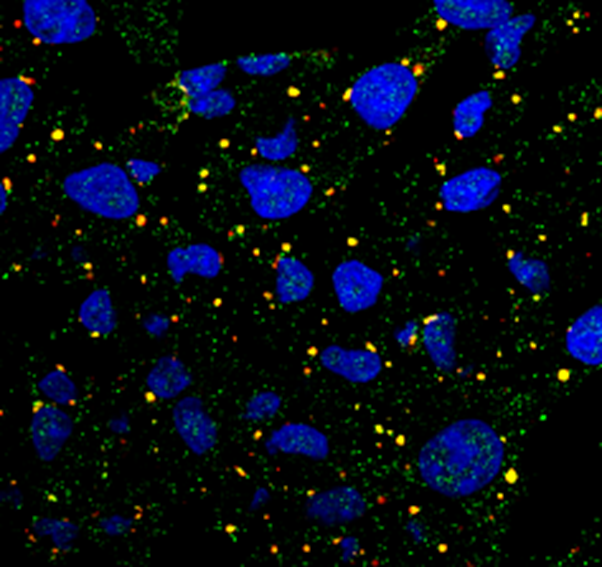

Supplement: Supplementary file 10 — Source Data Fig. 2 [file 44321_2024_32_MOESM10_ESM.zip › Figure 2/Figure 2D Merge Ar.tif]

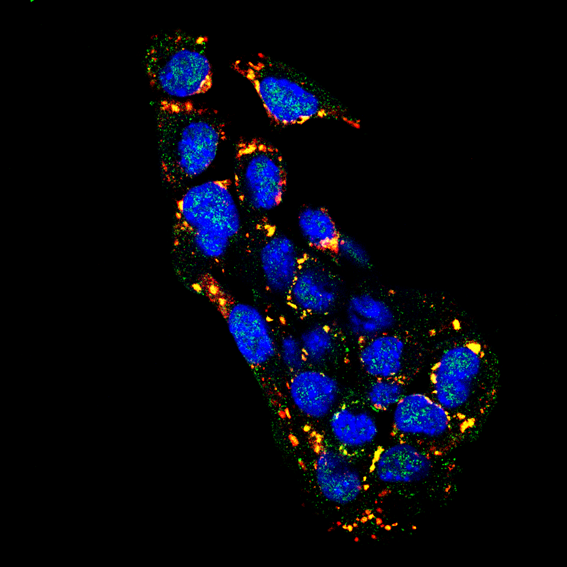

Supplement: Supplementary file 10 — Source Data Fig. 2 [file 44321_2024_32_MOESM10_ESM.zip › Figure 2/Figure 2E Merge WT Ar.tif]

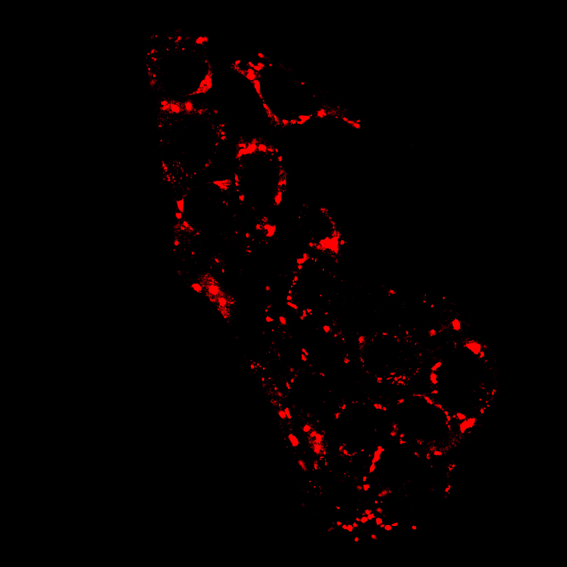

Supplement: Supplementary file 10 — Source Data Fig. 2 [file 44321_2024_32_MOESM10_ESM.zip › Figure 2/Figure 2E G3BP1 WT Ar.tif]

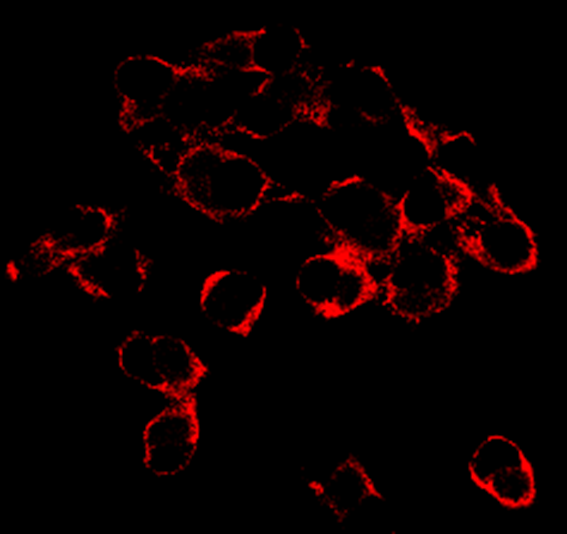

Supplement: Supplementary file 10 — Source Data Fig. 2 [file 44321_2024_32_MOESM10_ESM.zip › Figure 2/Figure 2D G3BP1 ZZW-115.tif]

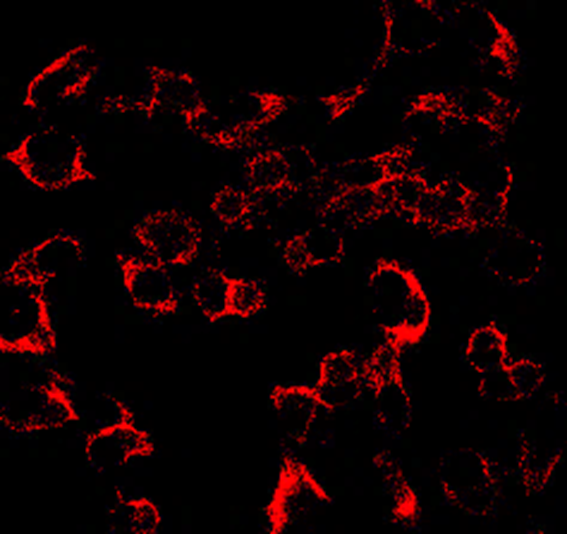

Supplement: Supplementary file 10 — Source Data Fig. 2 [file 44321_2024_32_MOESM10_ESM.zip › Figure 2/Figure 2D G3BP1 Control.tif]

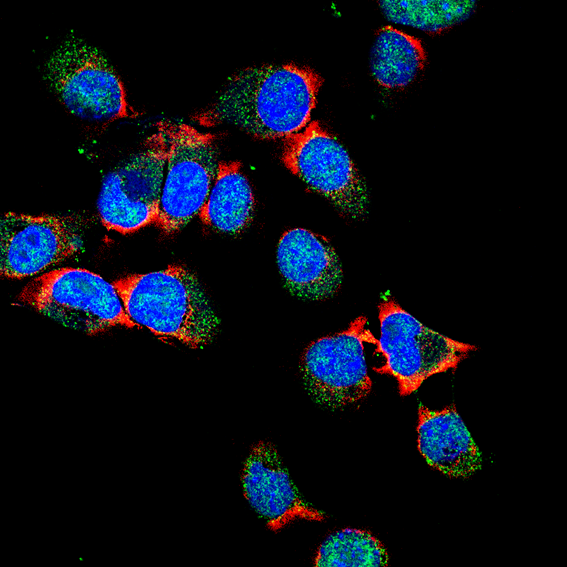

Supplement: Supplementary file 10 — Source Data Fig. 2 [file 44321_2024_32_MOESM10_ESM.zip › Figure 2/Figure 2E Merge WT Vehicle.tif]

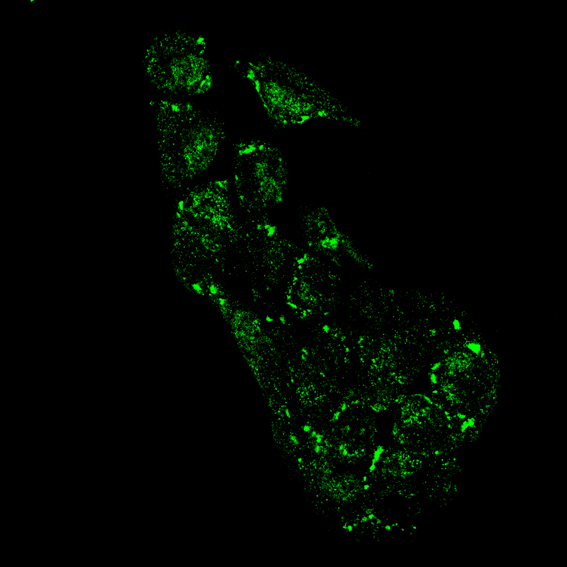

Supplement: Supplementary file 10 — Source Data Fig. 2 [file 44321_2024_32_MOESM10_ESM.zip › Figure 2/Figure 2E NUPR1 WT Ar.tif]

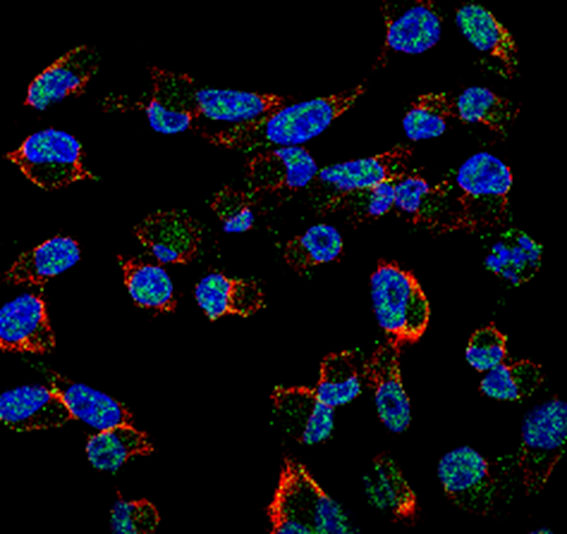

Supplement: Supplementary file 10 — Source Data Fig. 2 [file 44321_2024_32_MOESM10_ESM.zip › Figure 2/Figure 2D Merge Control.tif]

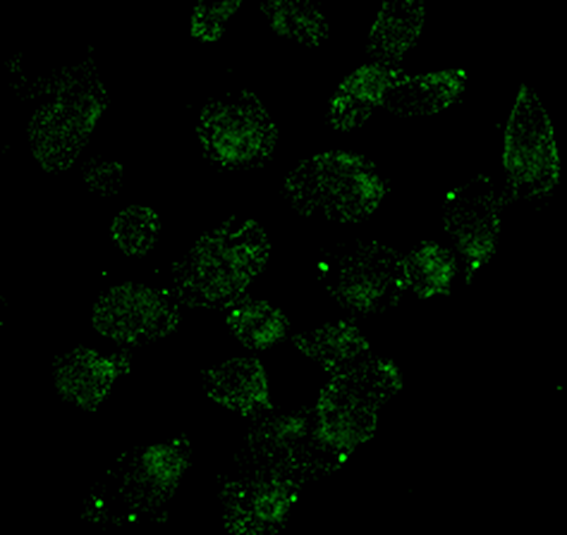

Supplement: Supplementary file 10 — Source Data Fig. 2 [file 44321_2024_32_MOESM10_ESM.zip › Figure 2/Figure 2D NUPR1 ZZW-115+Ar.tif]

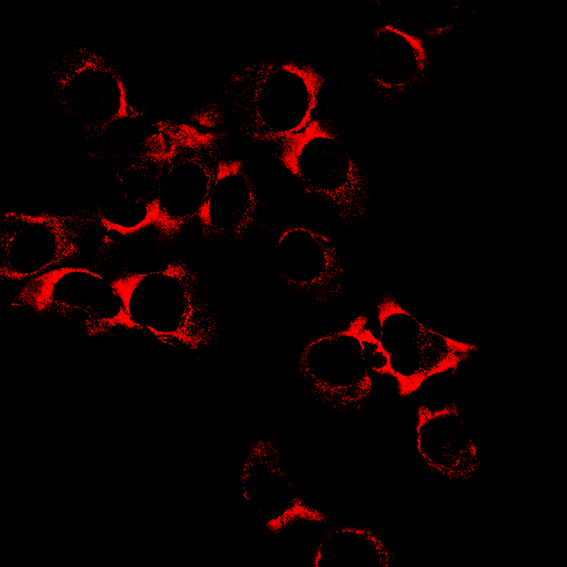

Supplement: Supplementary file 10 — Source Data Fig. 2 [file 44321_2024_32_MOESM10_ESM.zip › Figure 2/Figure 2E G3BP1 WT Vehicle.tif]

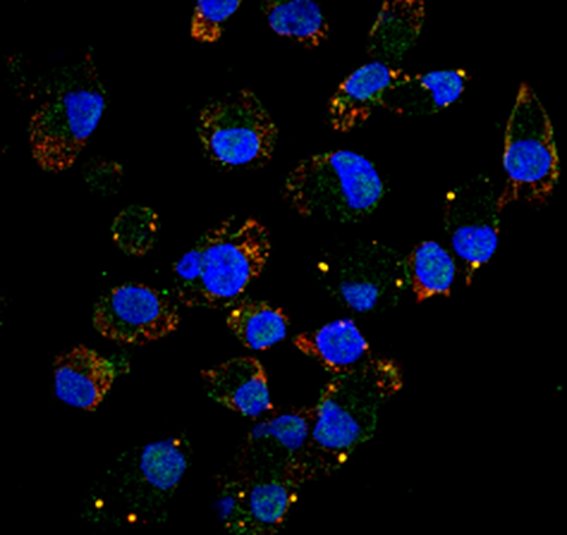

Supplement: Supplementary file 10 — Source Data Fig. 2 [file 44321_2024_32_MOESM10_ESM.zip › Figure 2/Figure 2D Merge ZZW-115+Ar.tif]

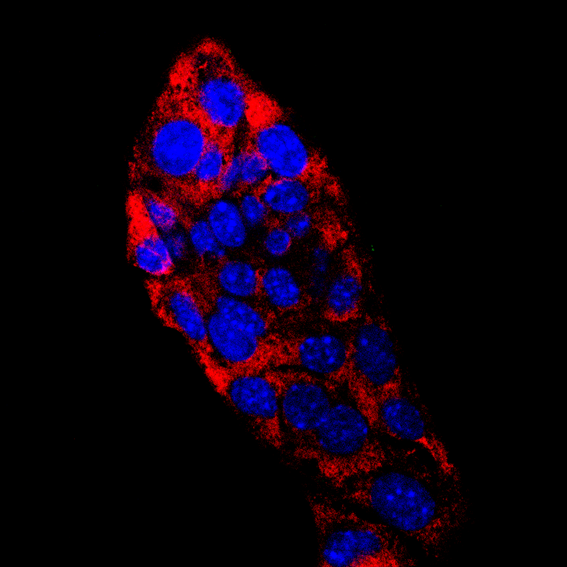

Supplement: Supplementary file 10 — Source Data Fig. 2 [file 44321_2024_32_MOESM10_ESM.zip › Figure 2/Figure 2E Merge KO Ar.tif]

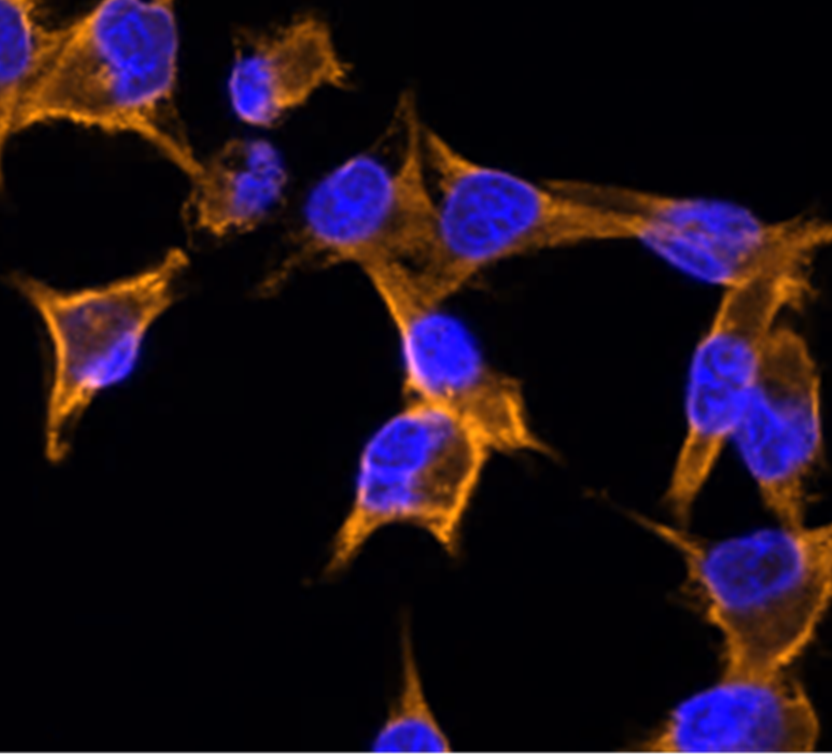

Supplement: Supplementary file 10 — Source Data Fig. 2 [file 44321_2024_32_MOESM10_ESM.zip › Figure 2/Figure 2C Ar.tif]

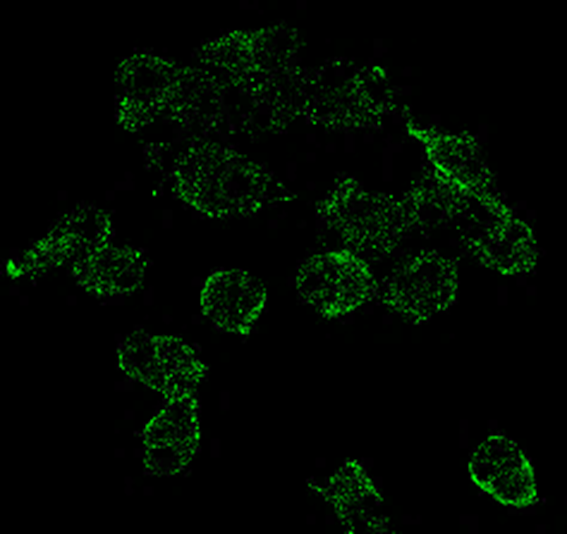

Supplement: Supplementary file 10 — Source Data Fig. 2 [file 44321_2024_32_MOESM10_ESM.zip › Figure 2/Figure 2D NUPR1 ZZW-115.tif]

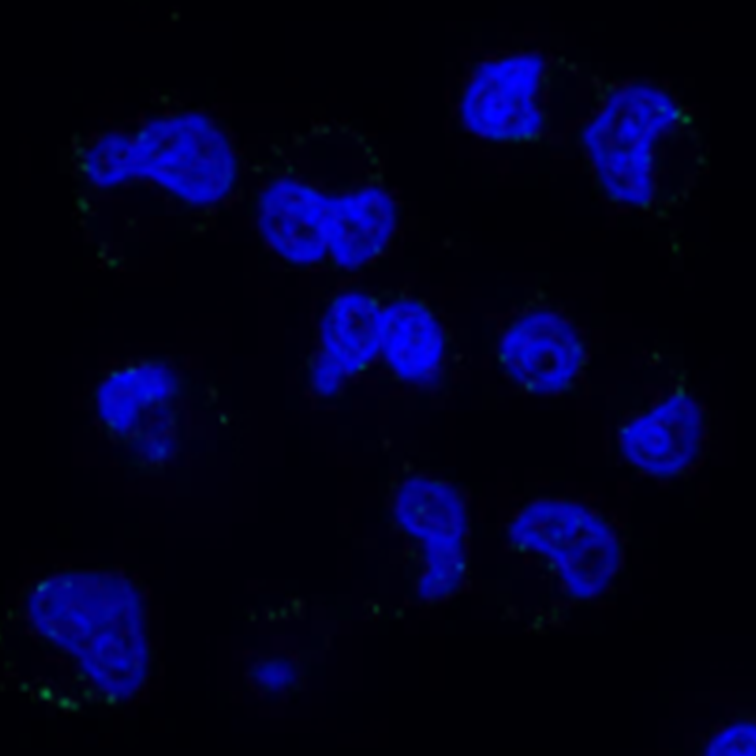

Supplement: Supplementary file 10 — Source Data Fig. 2 [file 44321_2024_32_MOESM10_ESM.zip › Figure 2/Figure 2B ZZW-115.tif]

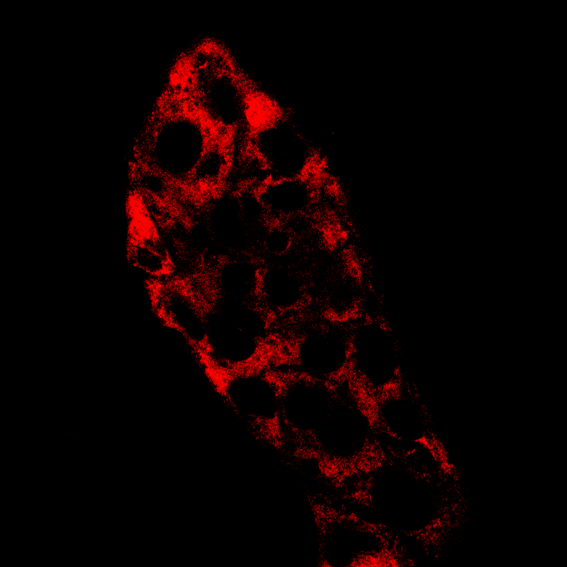

Supplement: Supplementary file 10 — Source Data Fig. 2 [file 44321_2024_32_MOESM10_ESM.zip › Figure 2/Figure 2E G3BP1 KO Ar.tif]

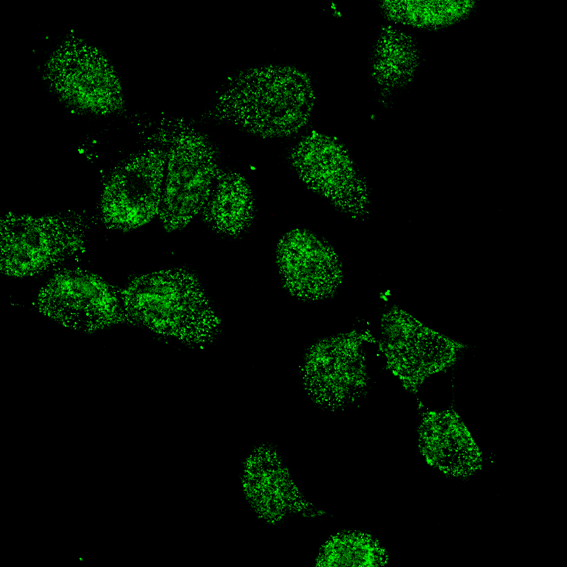

Supplement: Supplementary file 10 — Source Data Fig. 2 [file 44321_2024_32_MOESM10_ESM.zip › Figure 2/Figure 2E NUPR1 WT Vehicle.tif]

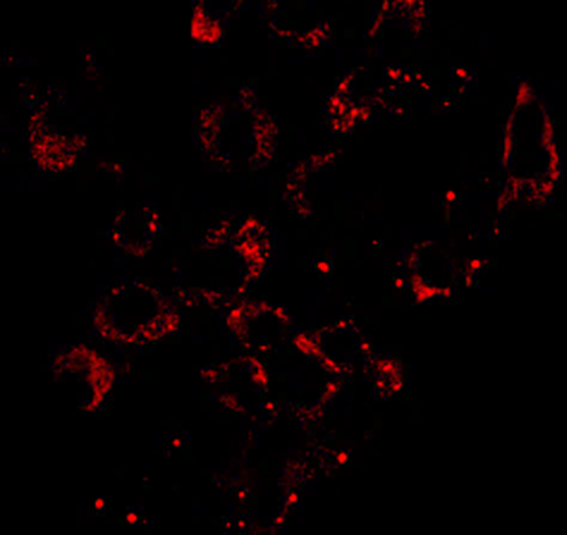

Supplement: Supplementary file 10 — Source Data Fig. 2 [file 44321_2024_32_MOESM10_ESM.zip › Figure 2/Figure 2D G3BP1 ZZW-115+Ar.tif]

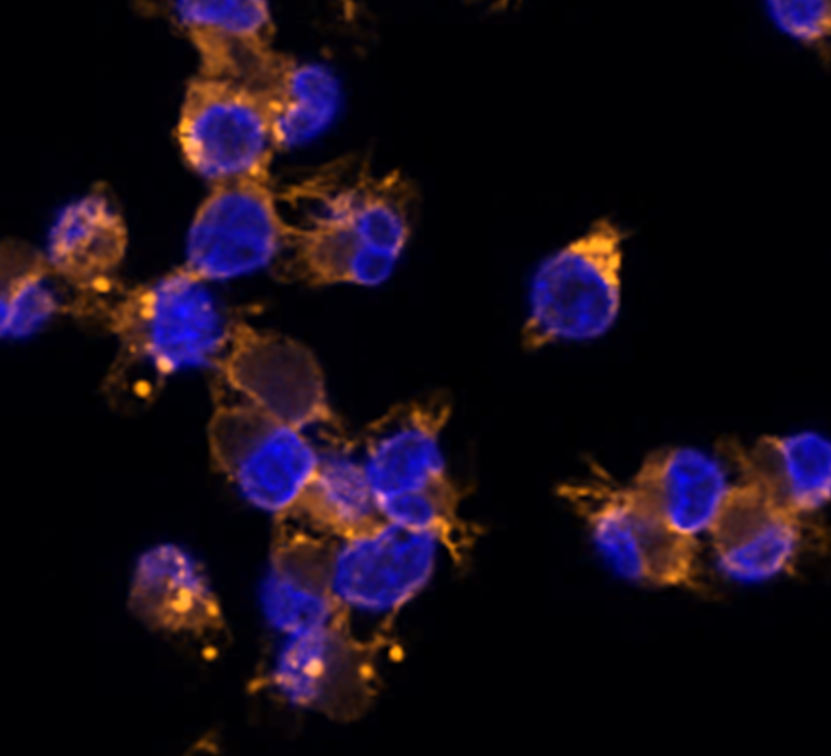

Supplement: Supplementary file 10 — Source Data Fig. 2 [file 44321_2024_32_MOESM10_ESM.zip › Figure 2/Figure 2C Ar siNUPR1.tif]

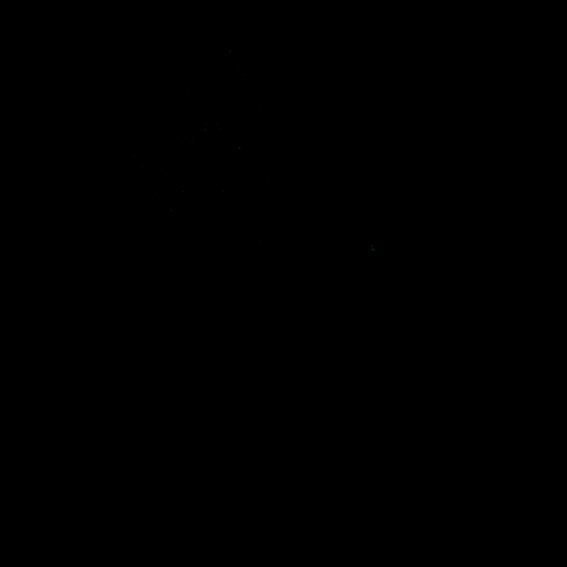

Supplement: Supplementary file 10 — Source Data Fig. 2 [file 44321_2024_32_MOESM10_ESM.zip › Figure 2/Figure 2E NUPR1 KO Ar.tif]

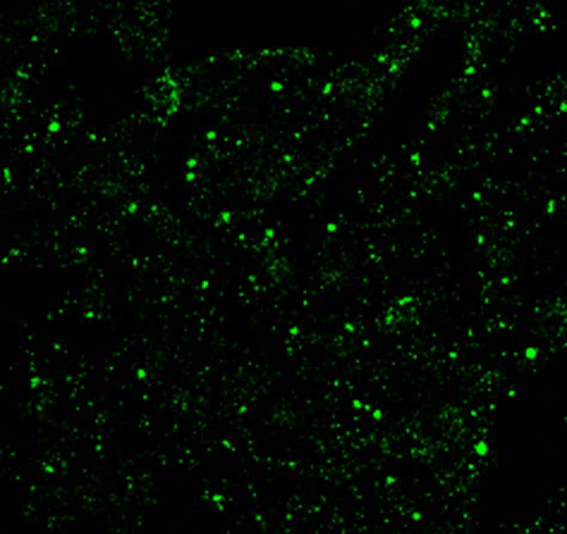

Supplement: Supplementary file 10 — Source Data Fig. 2 [file 44321_2024_32_MOESM10_ESM.zip › Figure 2/Figure 2D NUPR1 Ar.tif]

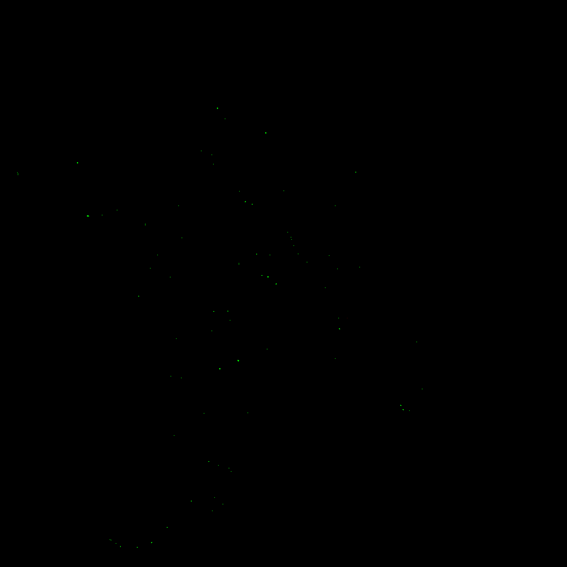

Supplement: Supplementary file 10 — Source Data Fig. 2 [file 44321_2024_32_MOESM10_ESM.zip › Figure 2/Figure 2E NUPR1 KO Vehicle.tif]

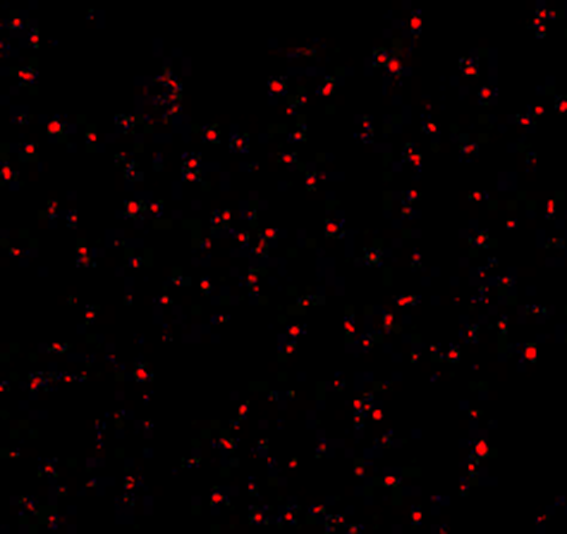

Supplement: Supplementary file 10 — Source Data Fig. 2 [file 44321_2024_32_MOESM10_ESM.zip › Figure 2/Figure 2D G3BP1 Ar.tif]

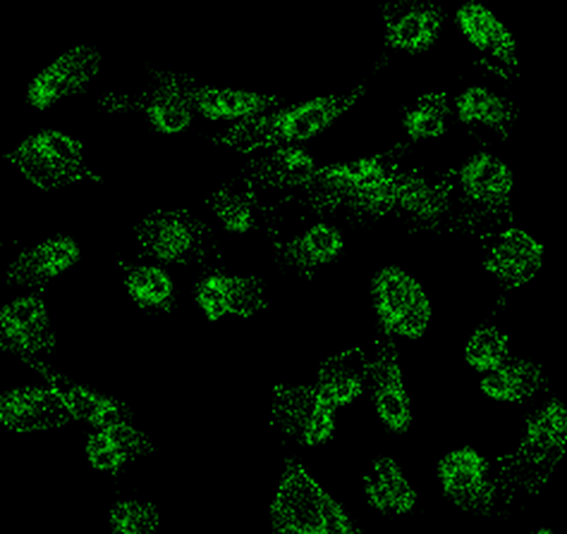

Supplement: Supplementary file 10 — Source Data Fig. 2 [file 44321_2024_32_MOESM10_ESM.zip › Figure 2/Figure 2D NUPR1 Control.tif]

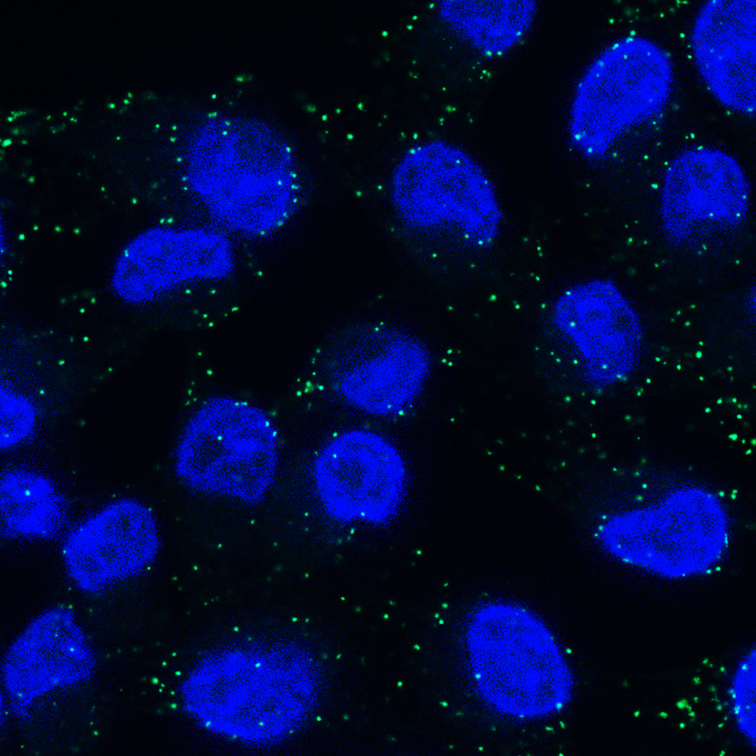

Supplement: Supplementary file 10 — Source Data Fig. 2 [file 44321_2024_32_MOESM10_ESM.zip › Figure 2/Figure 2B Control.tif]

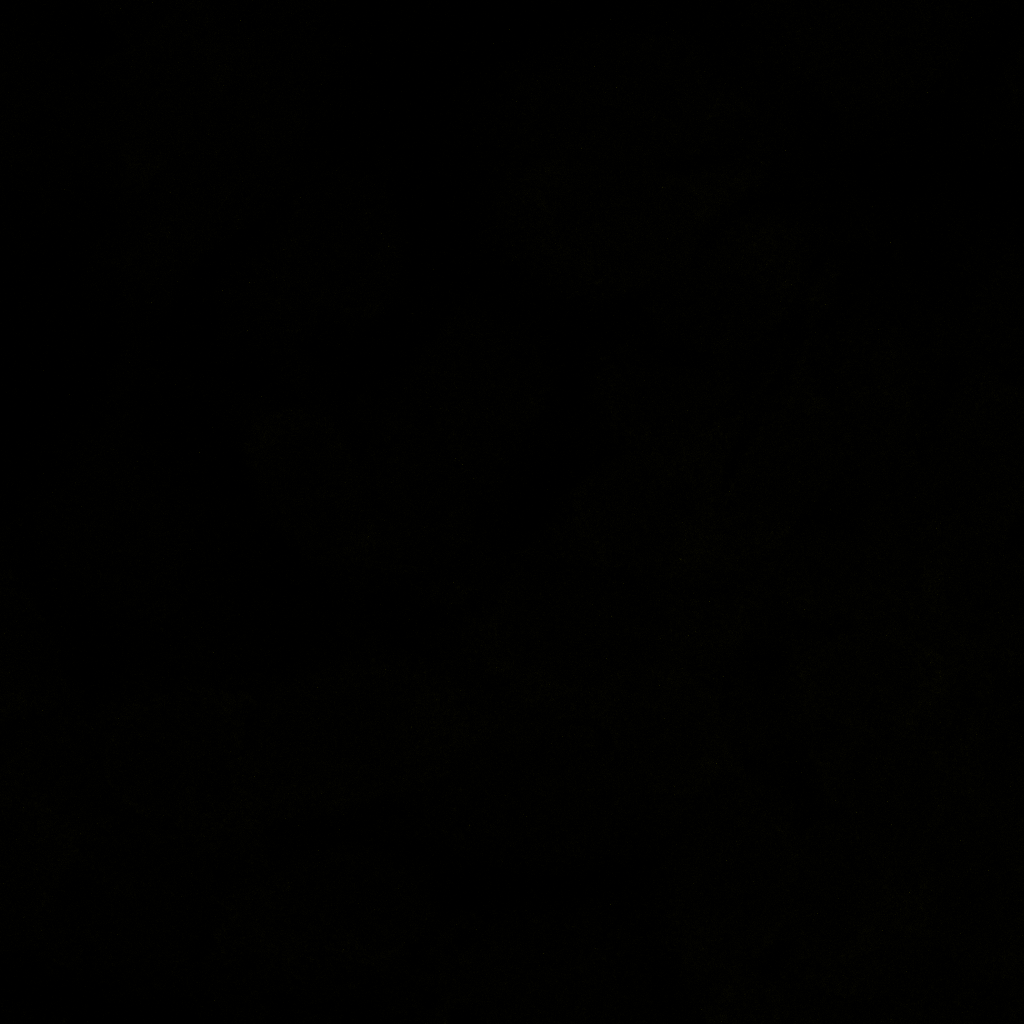

Supplement: Supplementary file 11 — Source Data Fig. 3 [file 44321_2024_32_MOESM11_ESM.zip › Figure 3/Figure 3C Untrated Flag GFP Dox.tif]

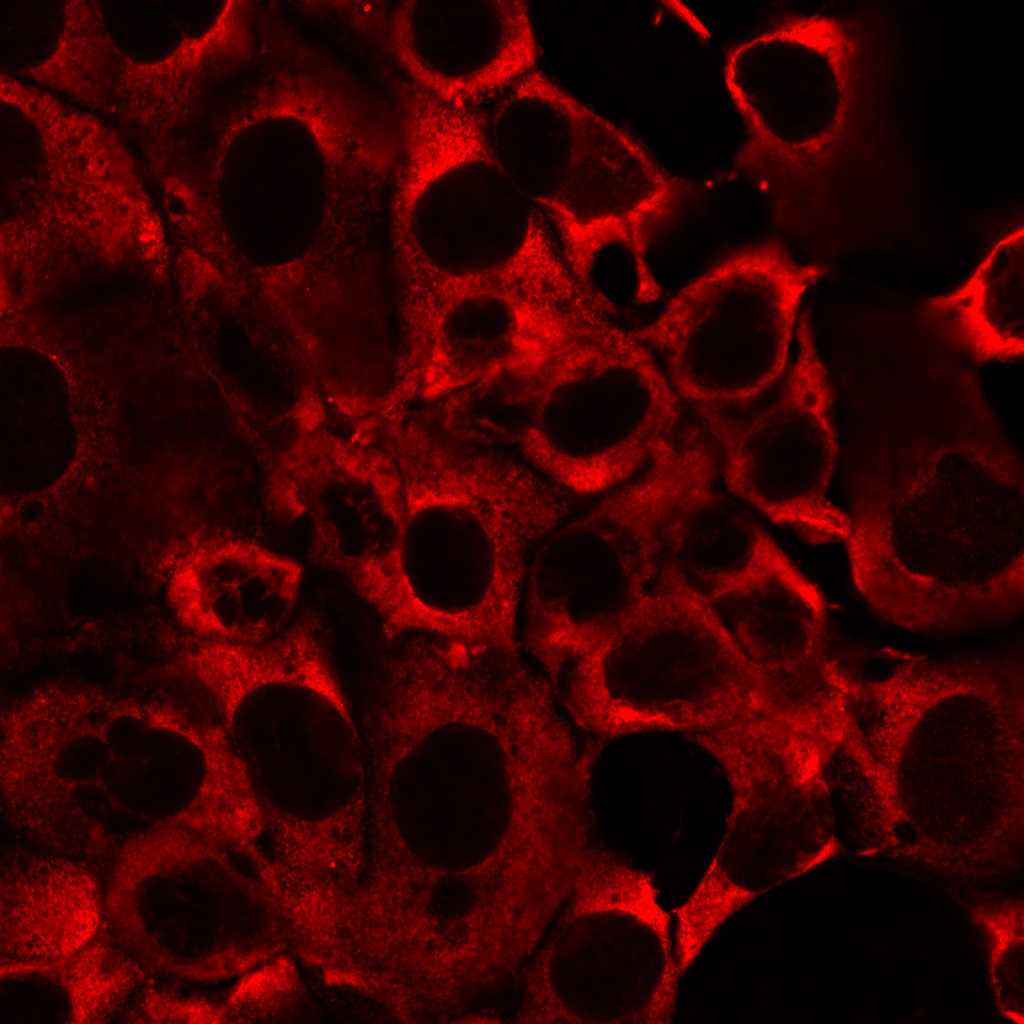

Supplement: Supplementary file 11 — Source Data Fig. 3 [file 44321_2024_32_MOESM11_ESM.zip › Figure 3/Figure 3C Untrated G3BP1 GFP Vehicle.tif]

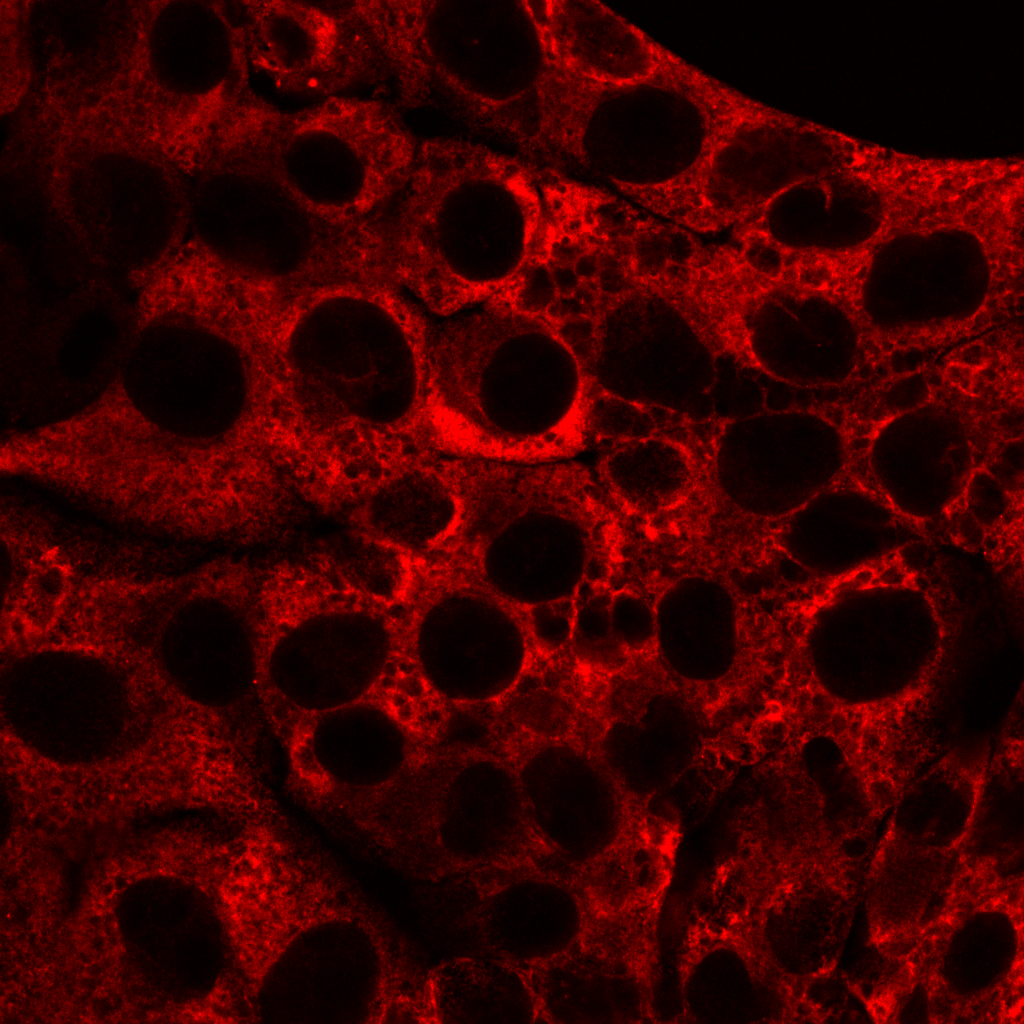

Supplement: Supplementary file 11 — Source Data Fig. 3 [file 44321_2024_32_MOESM11_ESM.zip › Figure 3/Figure 3C Untrated G3BP1 NUPR1mut-Flag Vehicle.tif]

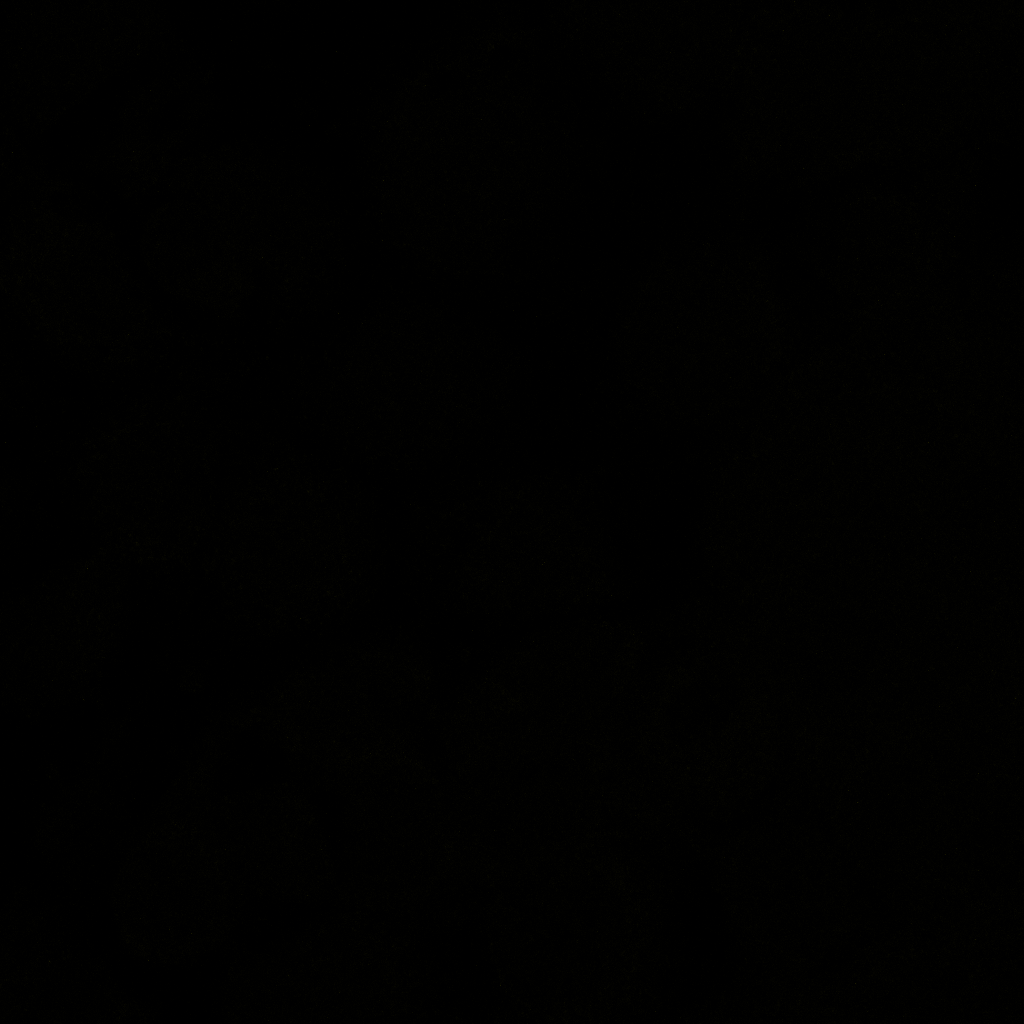

Supplement: Supplementary file 11 — Source Data Fig. 3 [file 44321_2024_32_MOESM11_ESM.zip › Figure 3/Figure 3C ZZW-115+Arsenate Flag GFP Vehicle.tif]

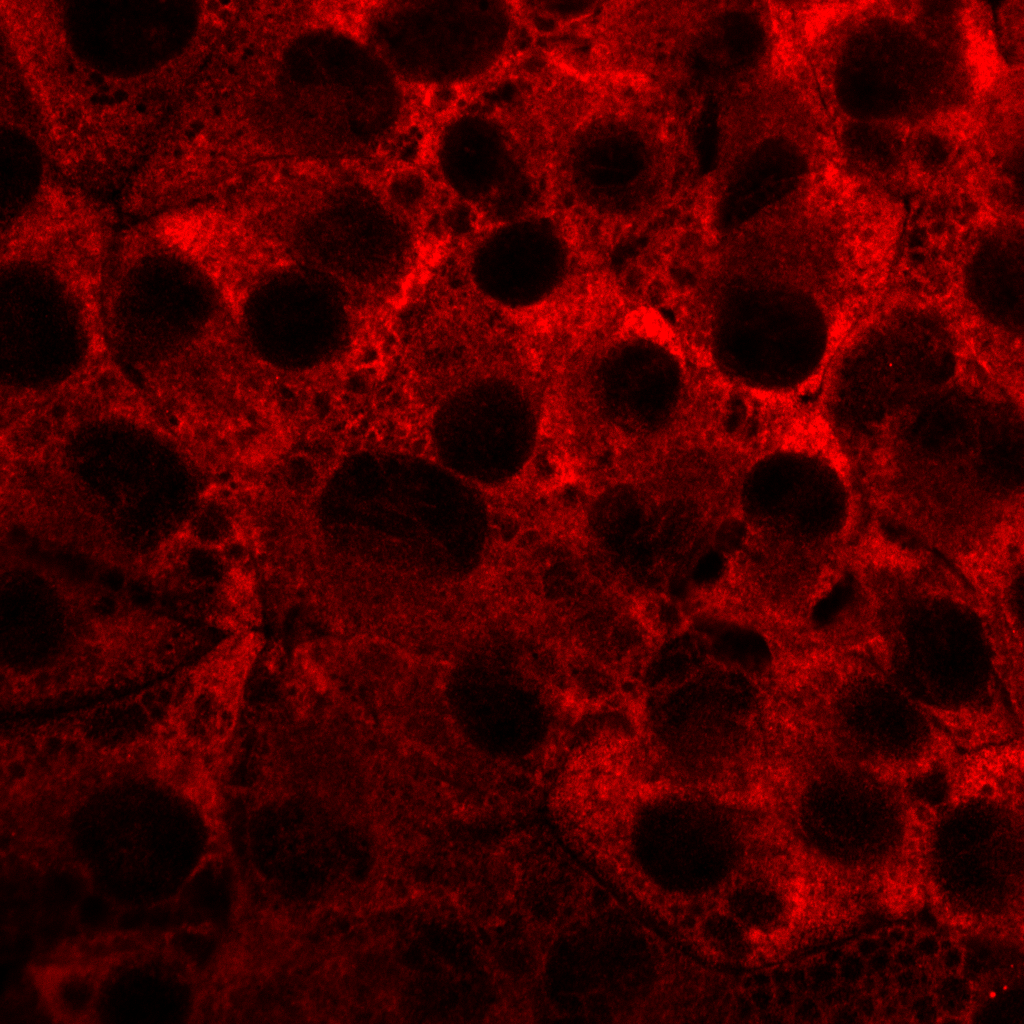

Supplement: Supplementary file 11 — Source Data Fig. 3 [file 44321_2024_32_MOESM11_ESM.zip › Figure 3/Figure 3C ZZW-115+Arsenate G3BP1 NUPR1mut-Flag Vehicle.tif]

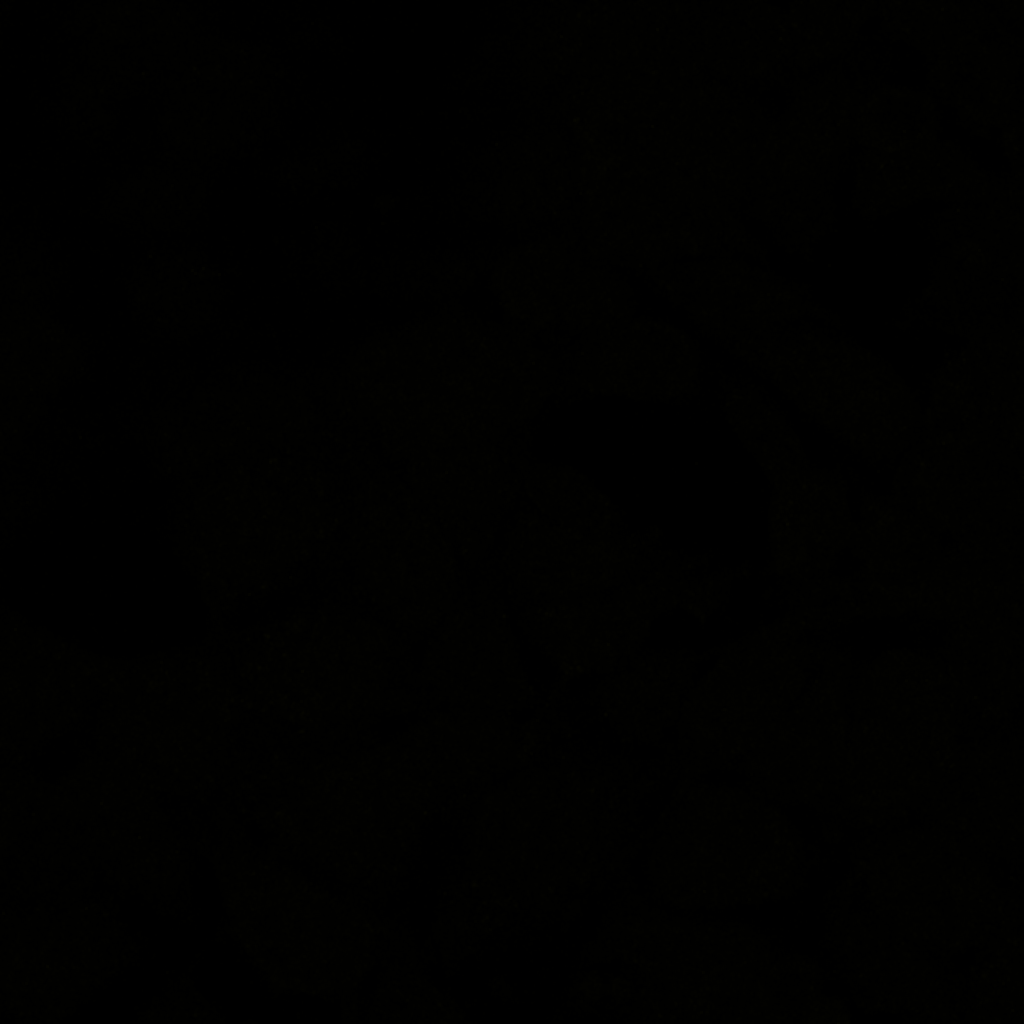

Supplement: Supplementary file 11 — Source Data Fig. 3 [file 44321_2024_32_MOESM11_ESM.zip › Figure 3/Figure 3CArsenate Flag GFP Vehicle.tif]

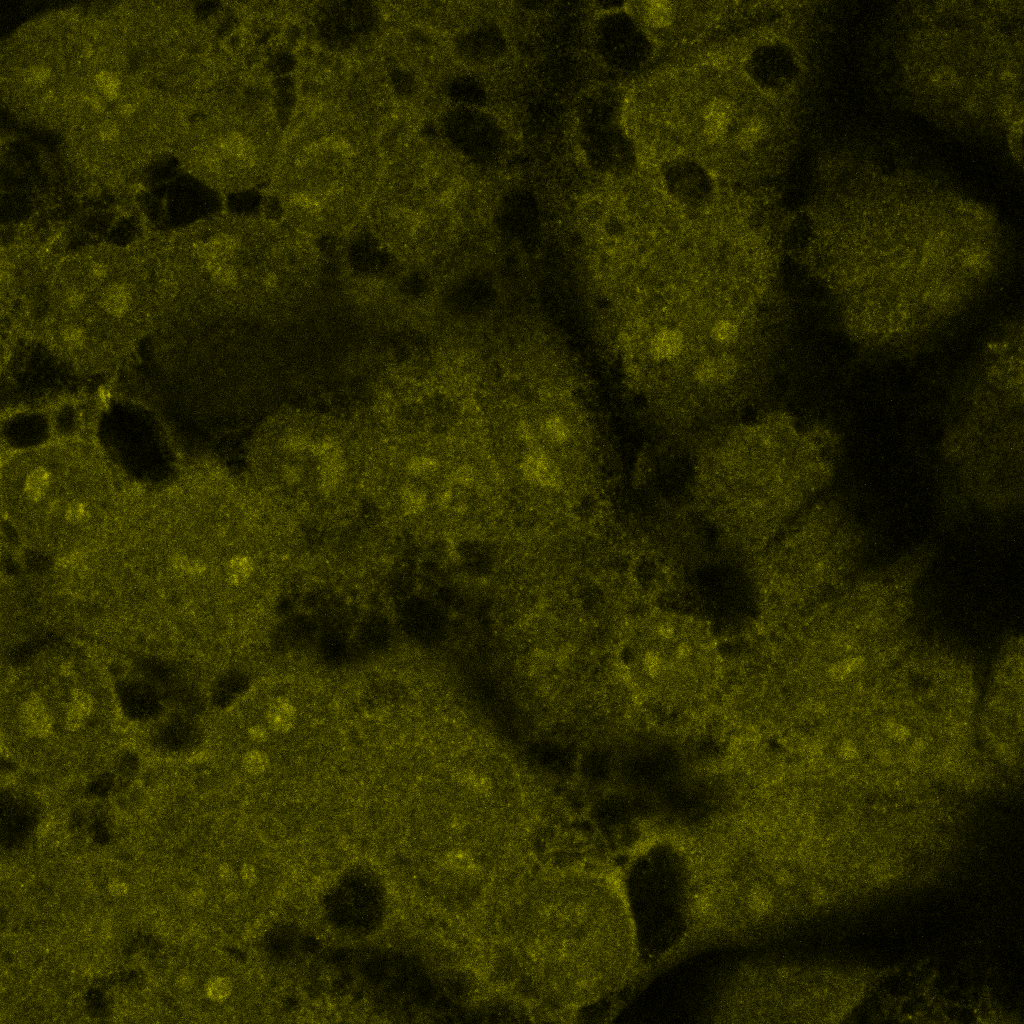

Supplement: Supplementary file 11 — Source Data Fig. 3 [file 44321_2024_32_MOESM11_ESM.zip › Figure 3/Figure 3C ZZW-115+Arsenate Flag NUPR1mut-Flag Dox.tif]

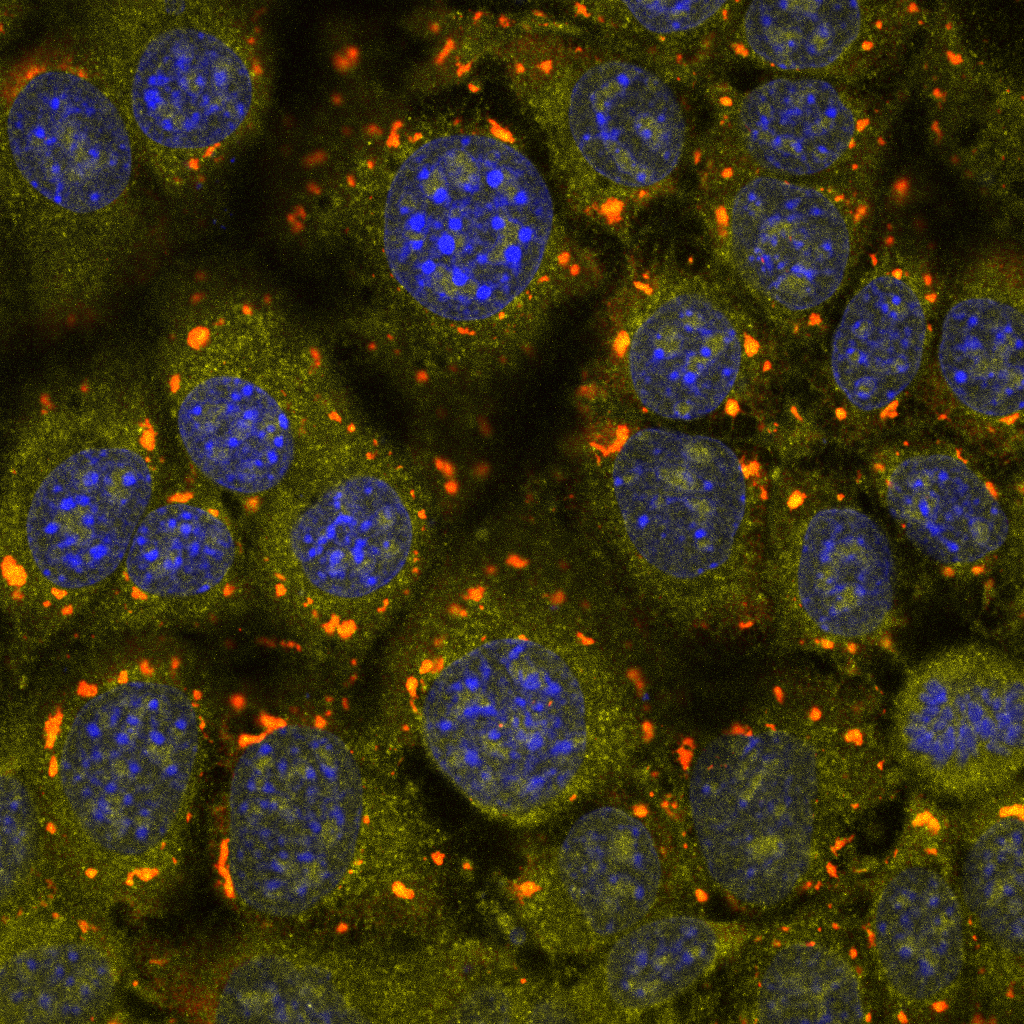

Supplement: Supplementary file 11 — Source Data Fig. 3 [file 44321_2024_32_MOESM11_ESM.zip › Figure 3/Figure 3CArsenate Merge NUPR1-Flag Vehicle.tif]

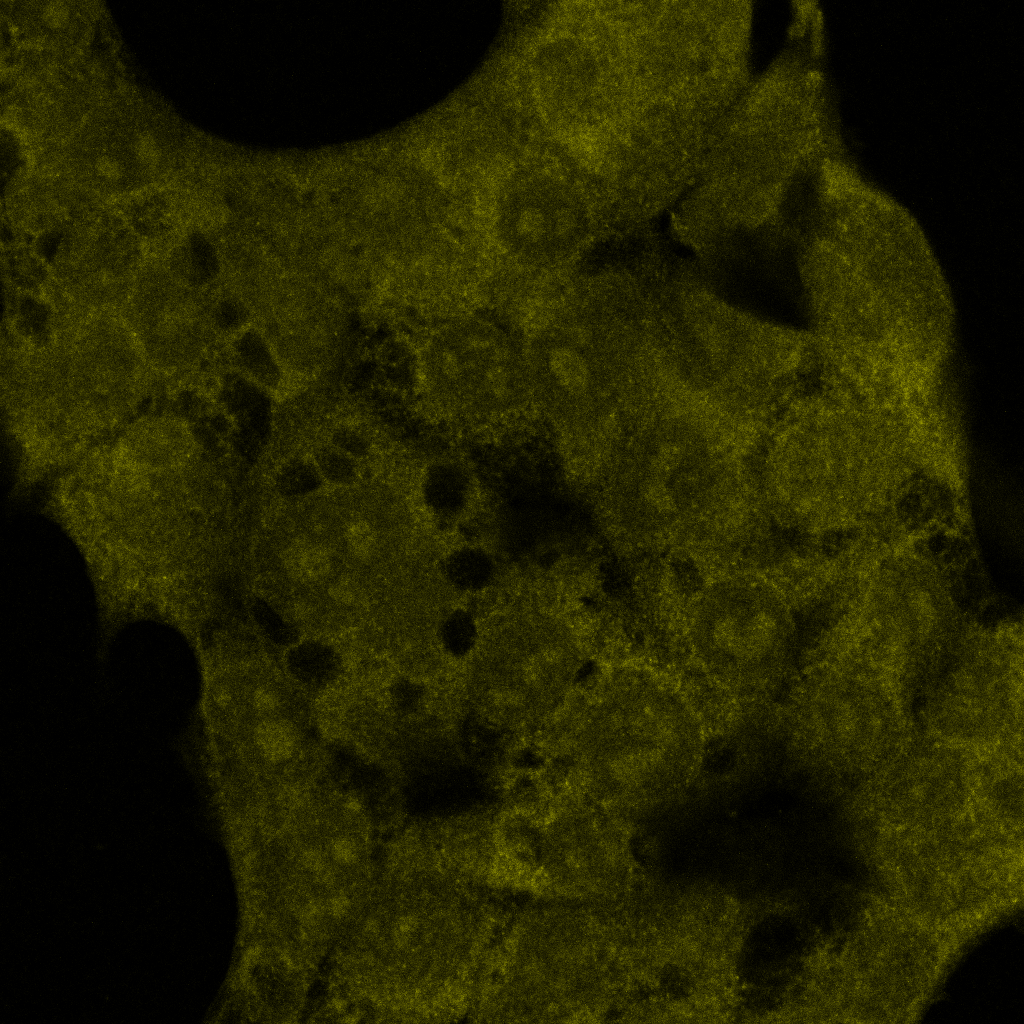

Supplement: Supplementary file 11 — Source Data Fig. 3 [file 44321_2024_32_MOESM11_ESM.zip › Figure 3/Figure 3C Arsenate Flag NUPR1mut-Flag Vehicle.tif]

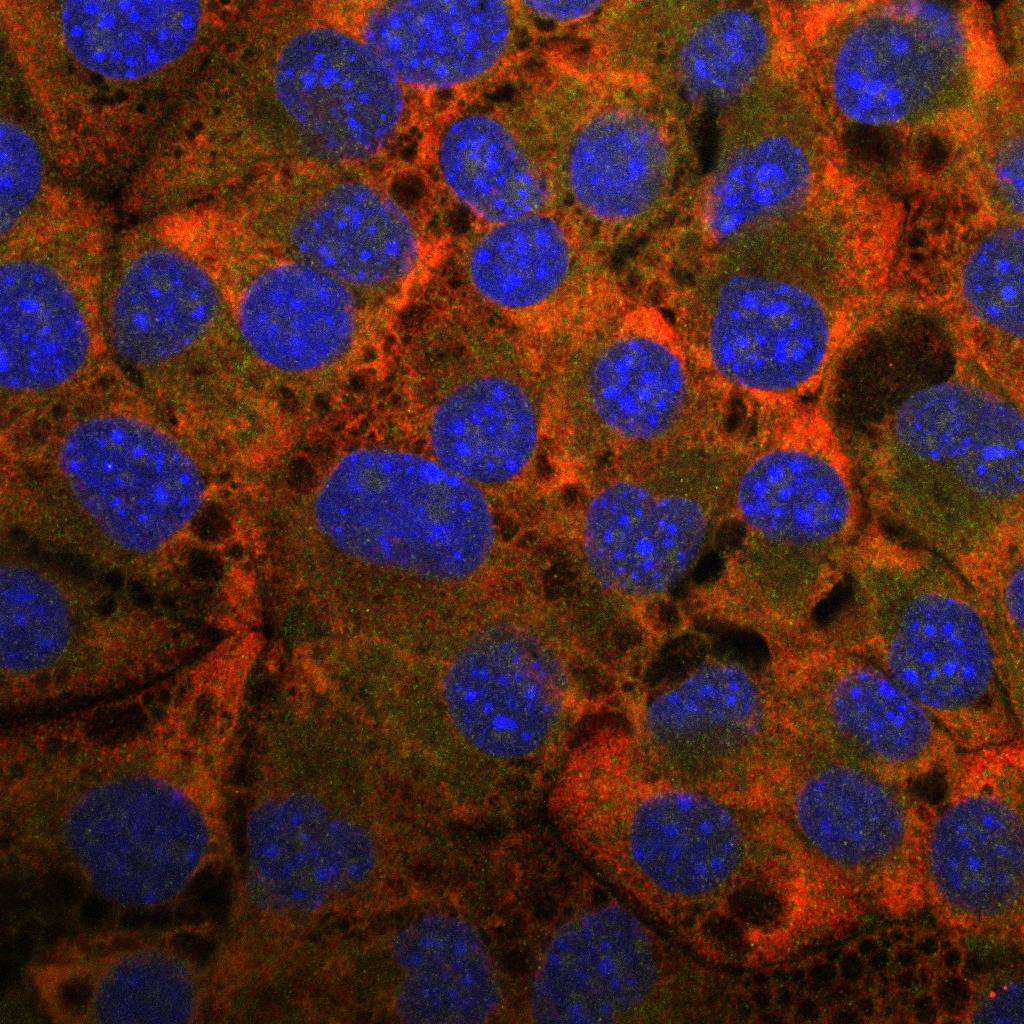

Supplement: Supplementary file 11 — Source Data Fig. 3 [file 44321_2024_32_MOESM11_ESM.zip › Figure 3/Figure 3C ZZW-115+Arsenate Merge NUPR1mut-Flag Vehicle.tif]

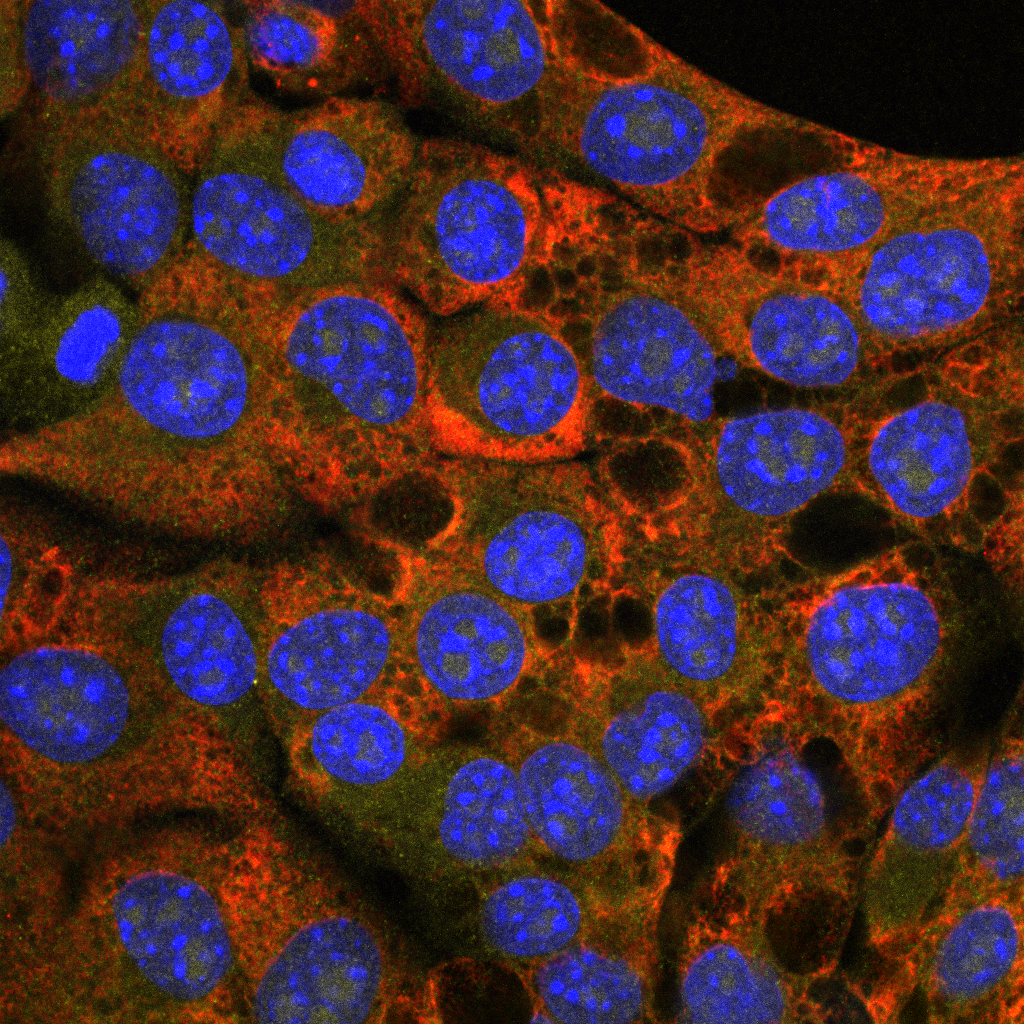

Supplement: Supplementary file 11 — Source Data Fig. 3 [file 44321_2024_32_MOESM11_ESM.zip › Figure 3/Figure 3C Untrated Merge NUPR1mut-Flag Vehicle.tif]

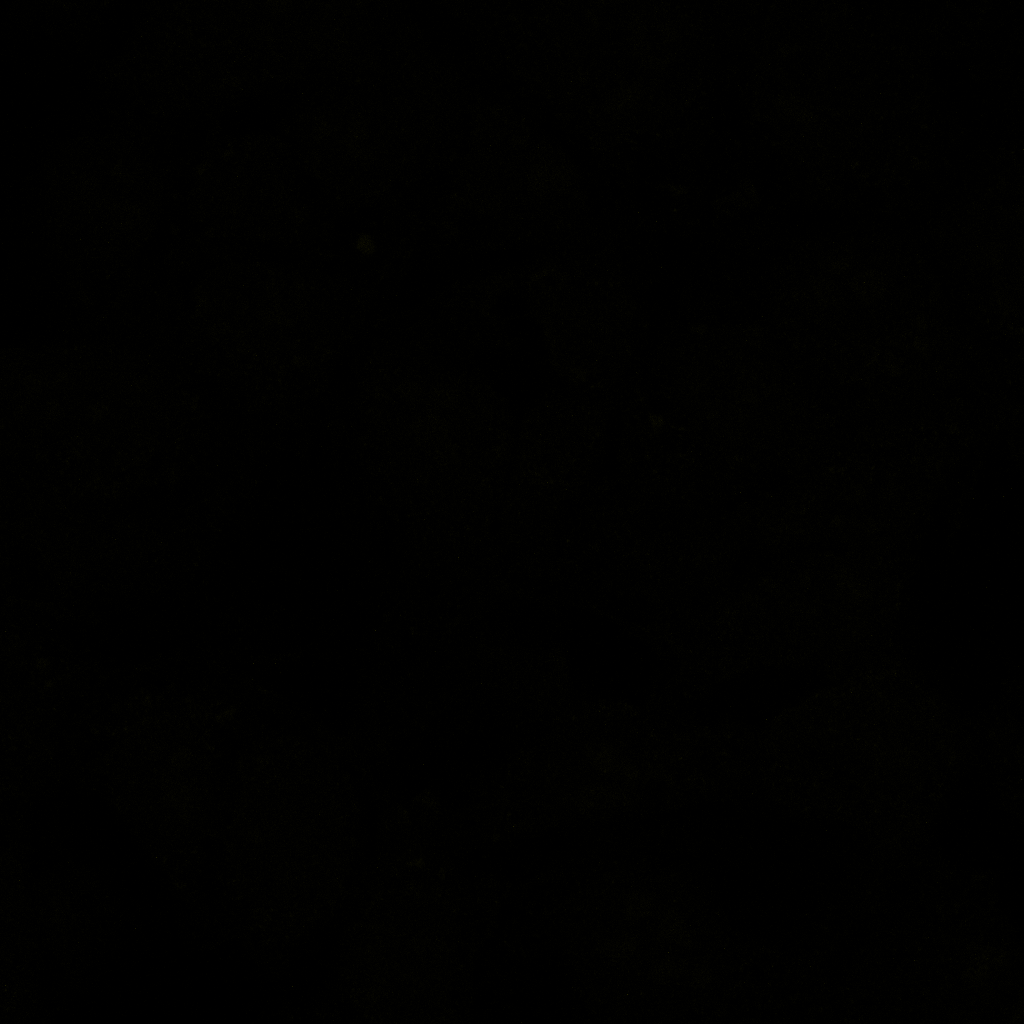

Supplement: Supplementary file 11 — Source Data Fig. 3 [file 44321_2024_32_MOESM11_ESM.zip › Figure 3/Figure 3C Arsenate Flag GFP Dox.tif]

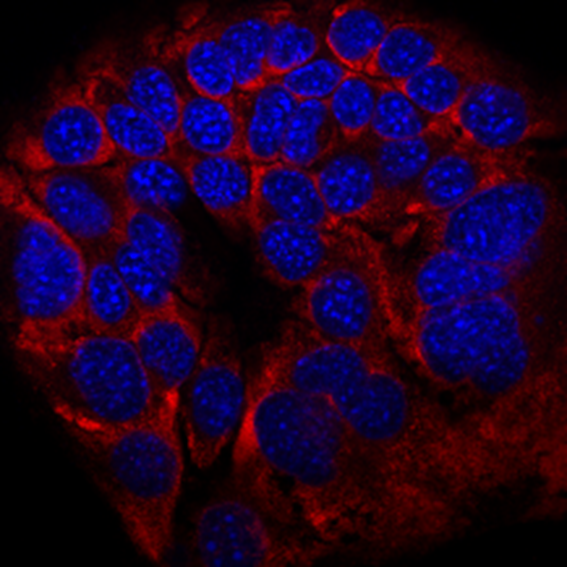

Supplement: Supplementary file 11 — Source Data Fig. 3 [file 44321_2024_32_MOESM11_ESM.zip › Figure 3/Figure 3A 4668 iKras Control Vehicle.tif]

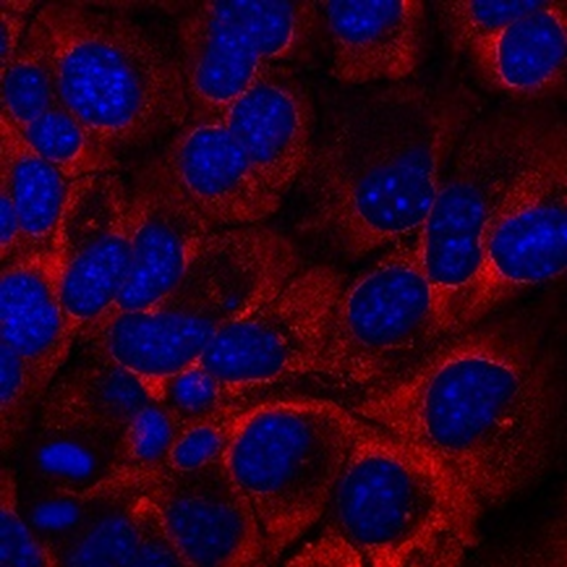

Supplement: Supplementary file 11 — Source Data Fig. 3 [file 44321_2024_32_MOESM11_ESM.zip › Figure 3/Figure 3A 4668 iKras Control Dox.tif]

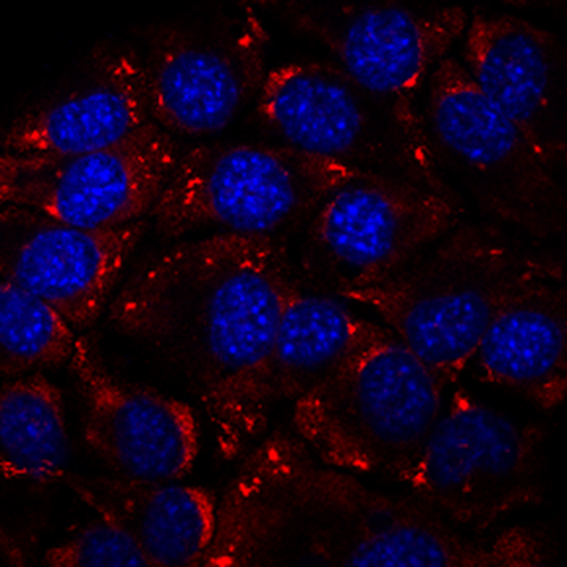

Supplement: Supplementary file 11 — Source Data Fig. 3 [file 44321_2024_32_MOESM11_ESM.zip › Figure 3/Figure 3A 4668 iKras Ar Dox.tif]

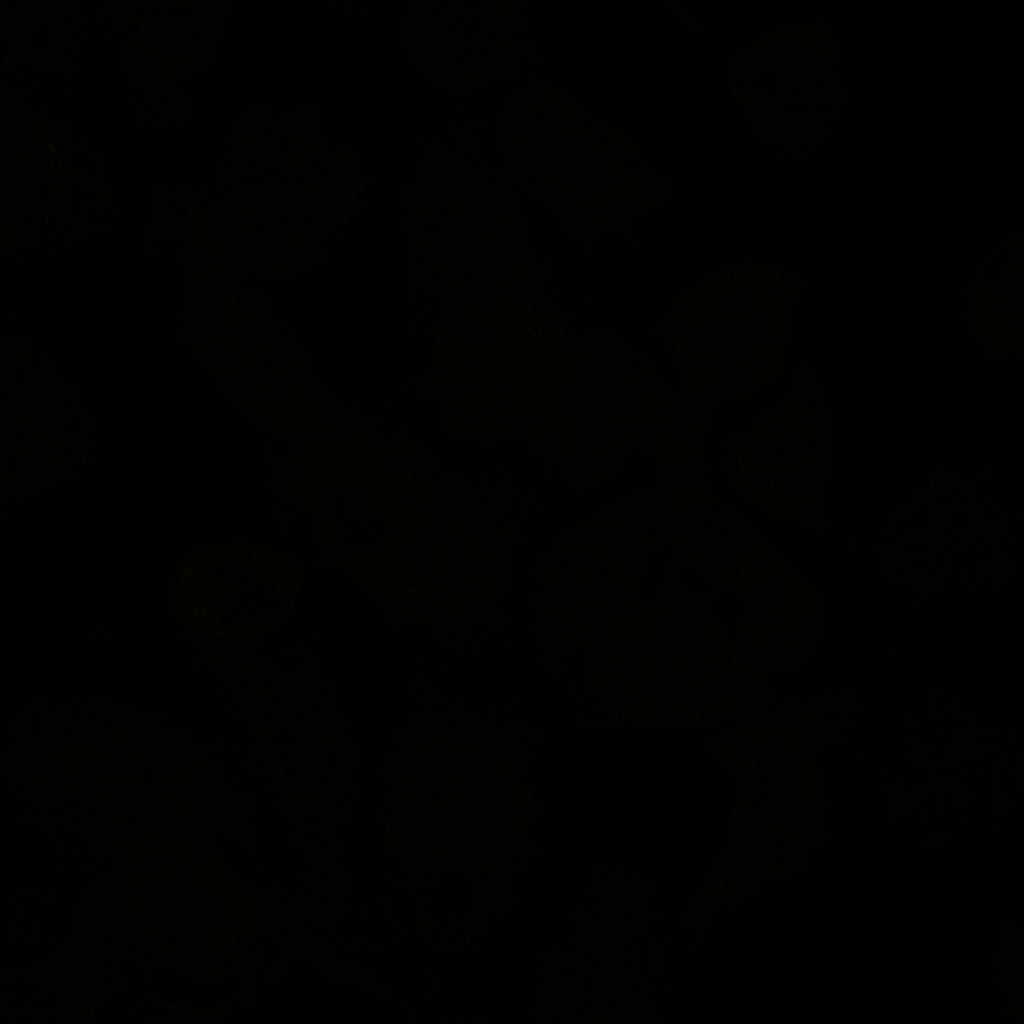

Supplement: Supplementary file 11 — Source Data Fig. 3 [file 44321_2024_32_MOESM11_ESM.zip › Figure 3/Figure 3C Untrated Flag GFP Vehicle.tif]

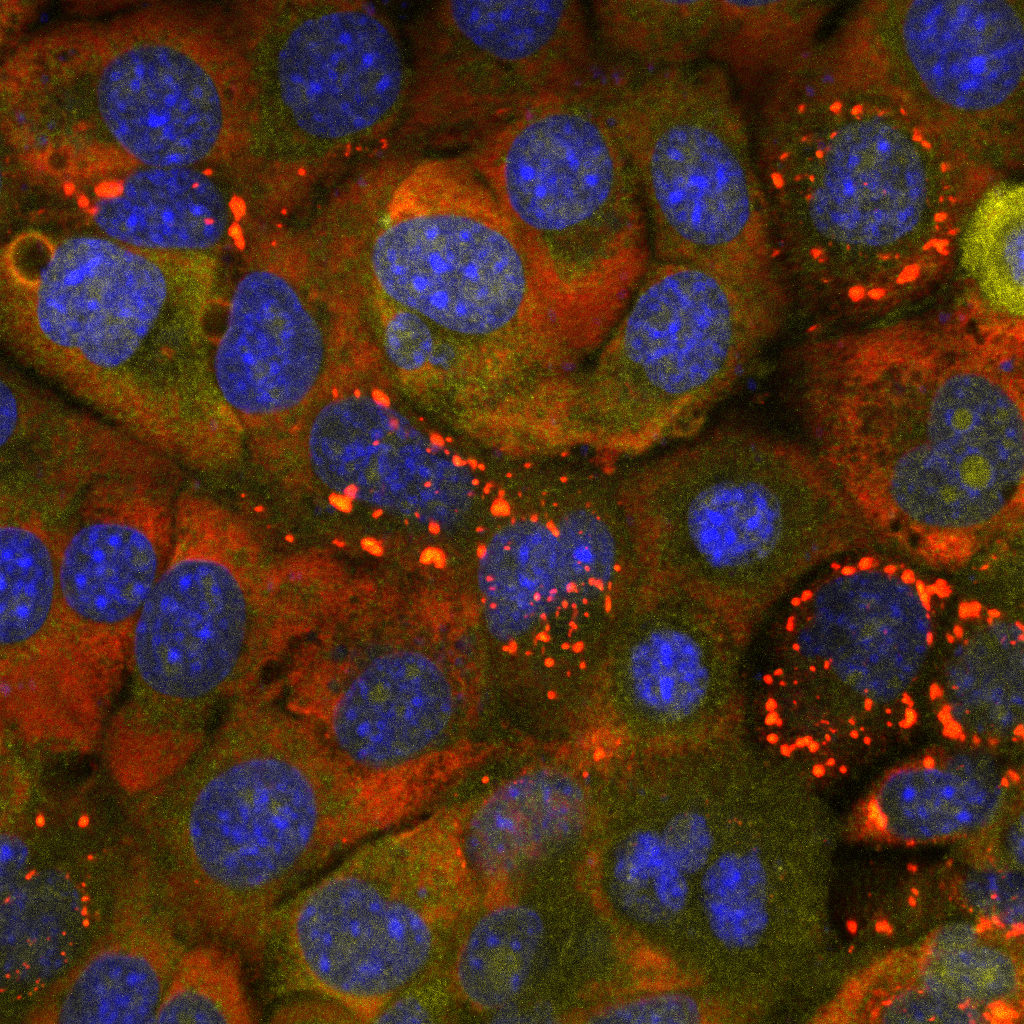

Supplement: Supplementary file 11 — Source Data Fig. 3 [file 44321_2024_32_MOESM11_ESM.zip › Figure 3/Figure 3C Untrated Merge NUPR1-Flag Vehicle.tif]

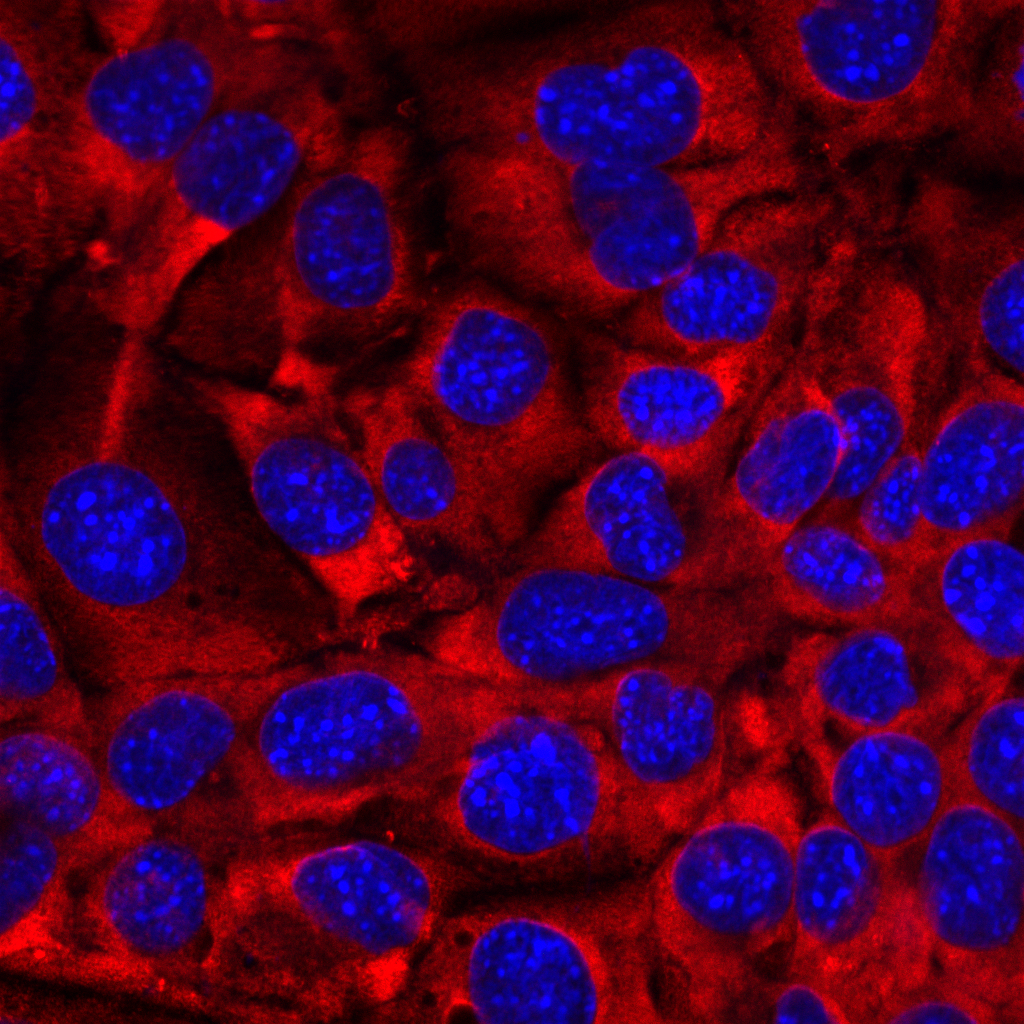

Supplement: Supplementary file 11 — Source Data Fig. 3 [file 44321_2024_32_MOESM11_ESM.zip › Figure 3/Figure 3C Untrated Merge GFP Dox.tif]

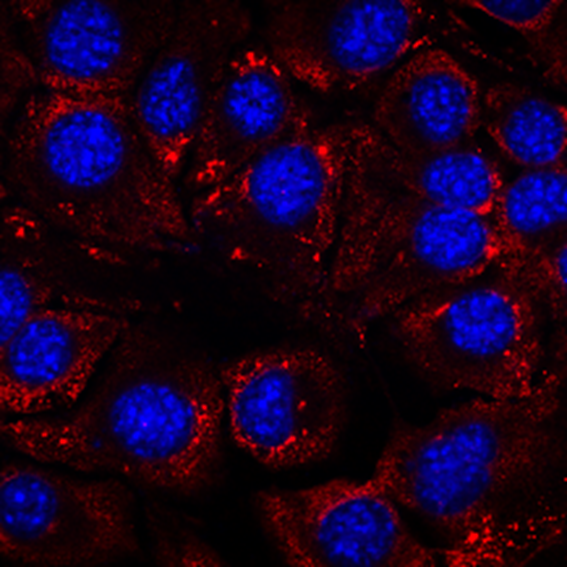

Supplement: Supplementary file 11 — Source Data Fig. 3 [file 44321_2024_32_MOESM11_ESM.zip › Figure 3/Figure 3A 4292 iKras Ar Dox.tif]

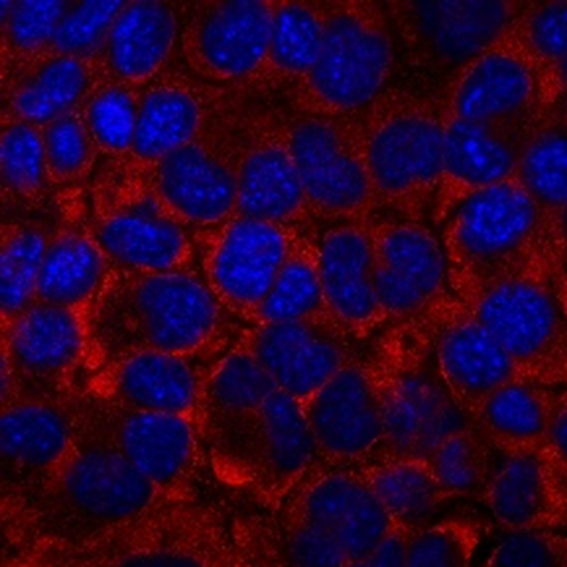

Supplement: Supplementary file 11 — Source Data Fig. 3 [file 44321_2024_32_MOESM11_ESM.zip › Figure 3/Figure 3A 9805 iKras Control Dox.tif]

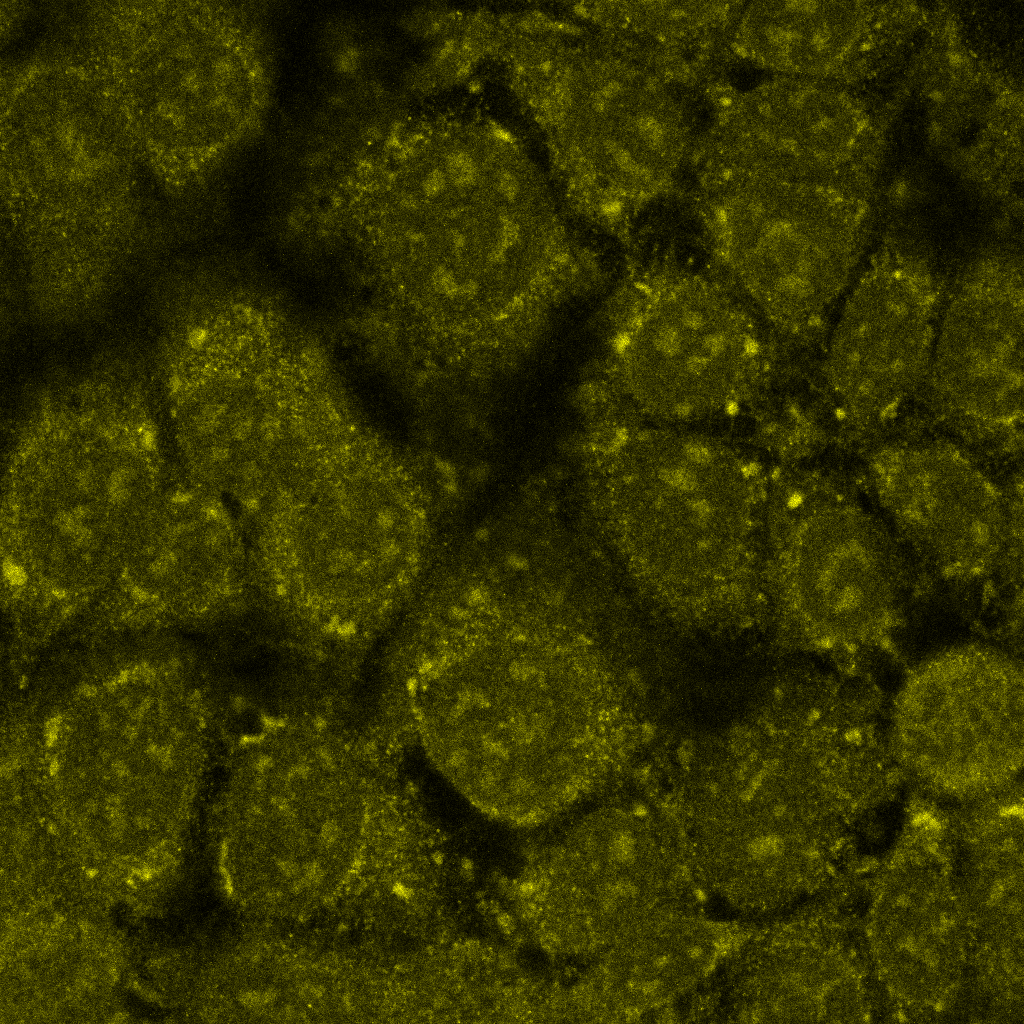

Supplement: Supplementary file 11 — Source Data Fig. 3 [file 44321_2024_32_MOESM11_ESM.zip › Figure 3/Figure 3C Arsenate Flag NUPR1-Flag Vehicle.tif]

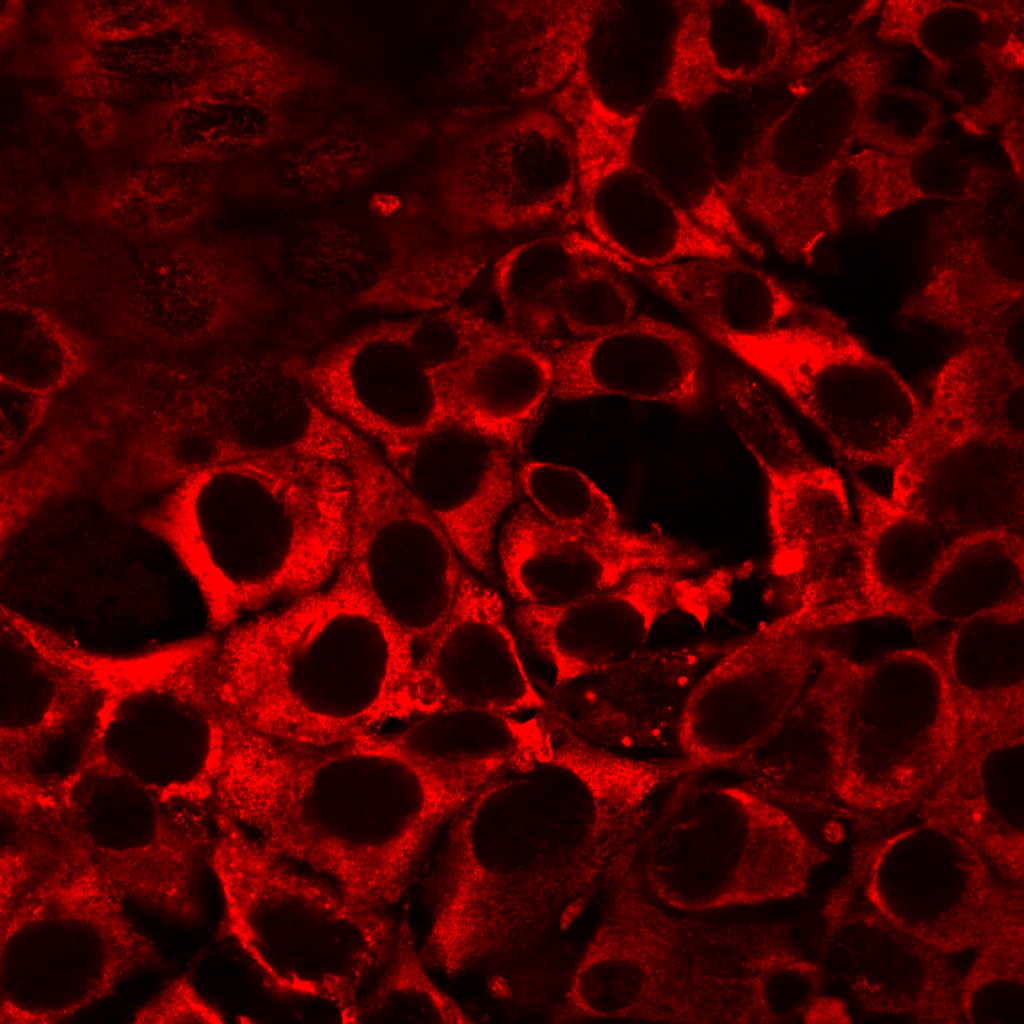

Supplement: Supplementary file 11 — Source Data Fig. 3 [file 44321_2024_32_MOESM11_ESM.zip › Figure 3/Figure 3C Arsenate G3BP1 GFP Vehicle.tif]

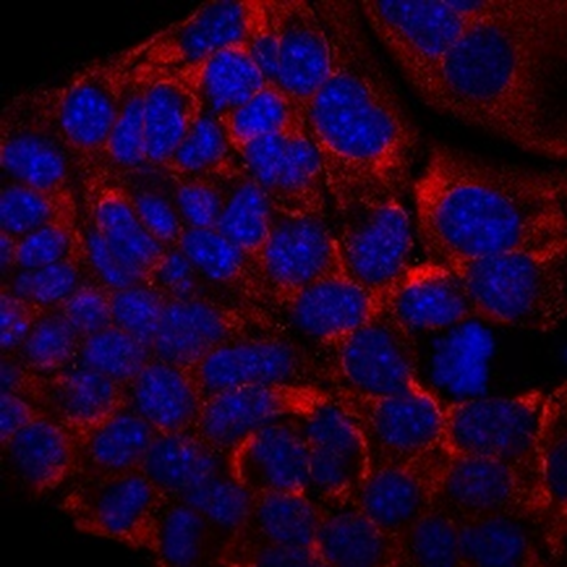

Supplement: Supplementary file 11 — Source Data Fig. 3 [file 44321_2024_32_MOESM11_ESM.zip › Figure 3/Figure 3A 4668 iKras Ar Vehicle.tif]

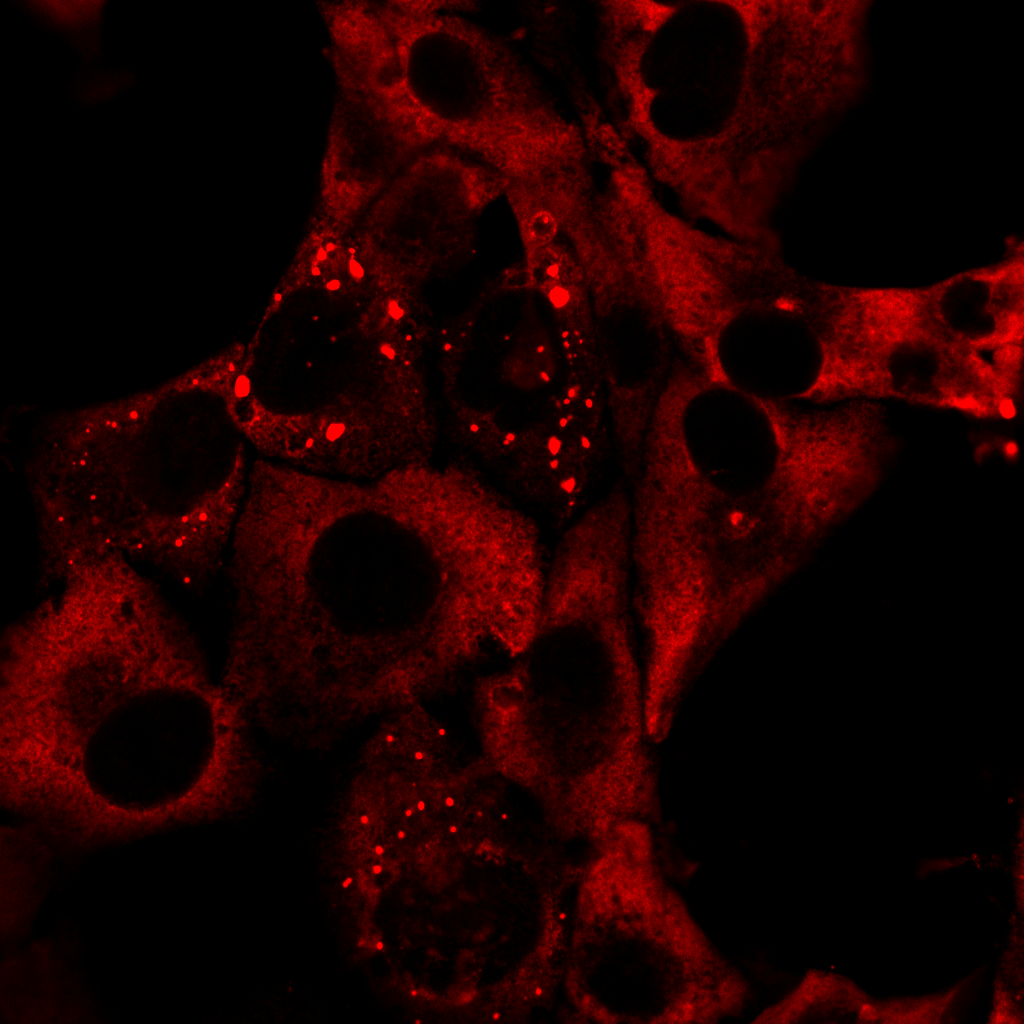

Supplement: Supplementary file 11 — Source Data Fig. 3 [file 44321_2024_32_MOESM11_ESM.zip › Figure 3/Figure 3C Untrated G3BP1 NUPR1-Flag Dox.tif]

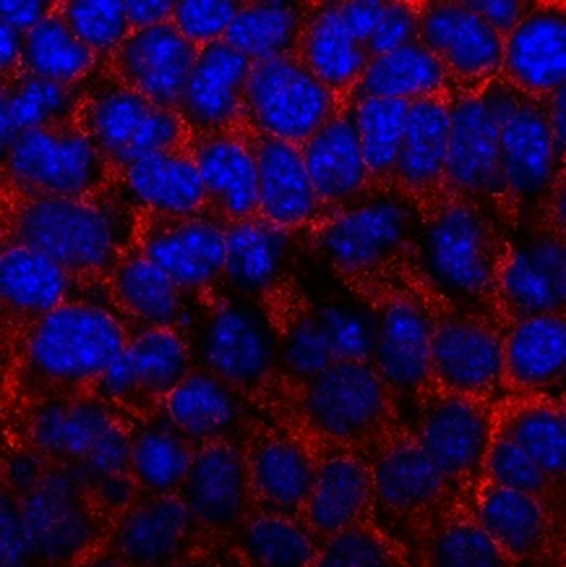

Supplement: Supplementary file 11 — Source Data Fig. 3 [file 44321_2024_32_MOESM11_ESM.zip › Figure 3/Figure 3A 9805 iKras Control Vehicle.tif]

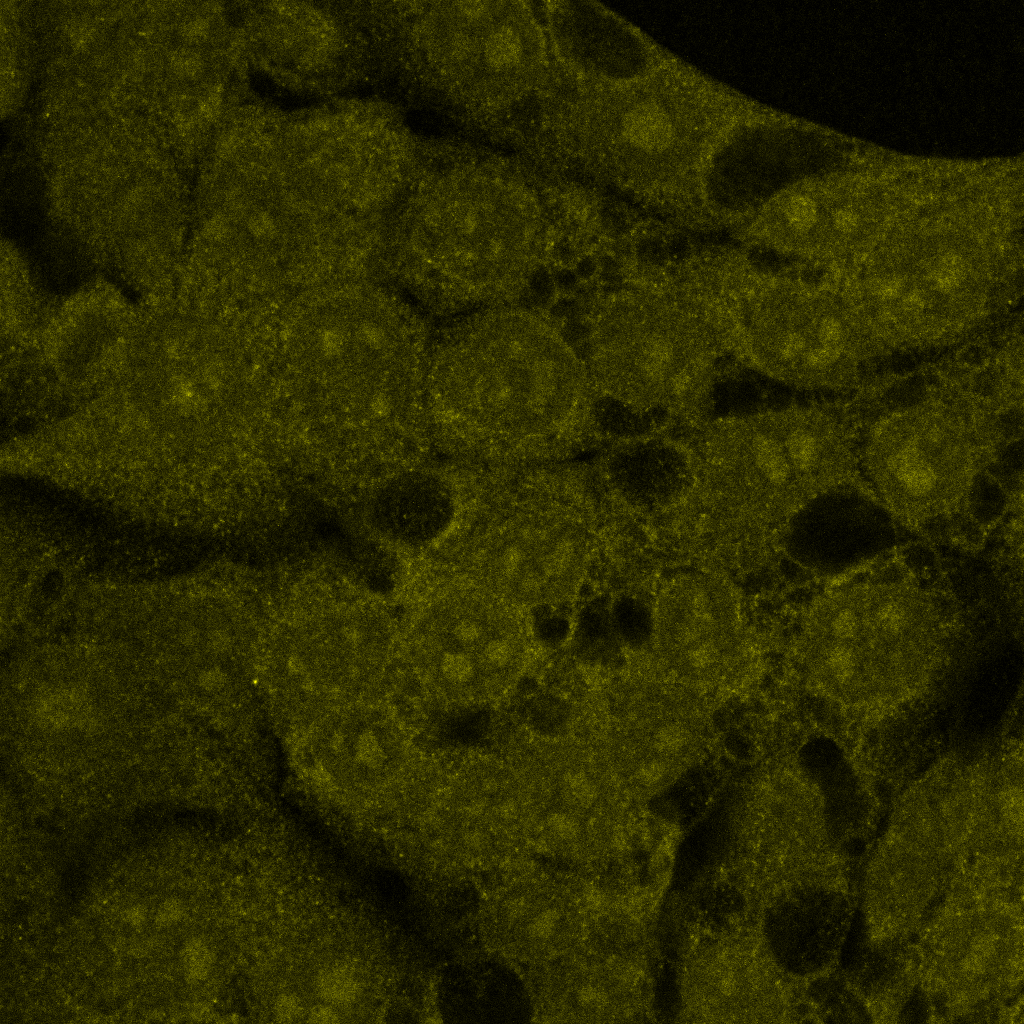

Supplement: Supplementary file 11 — Source Data Fig. 3 [file 44321_2024_32_MOESM11_ESM.zip › Figure 3/Figure 3C Untrated Flag NUPR1mut-Flag Vehicle.tif]

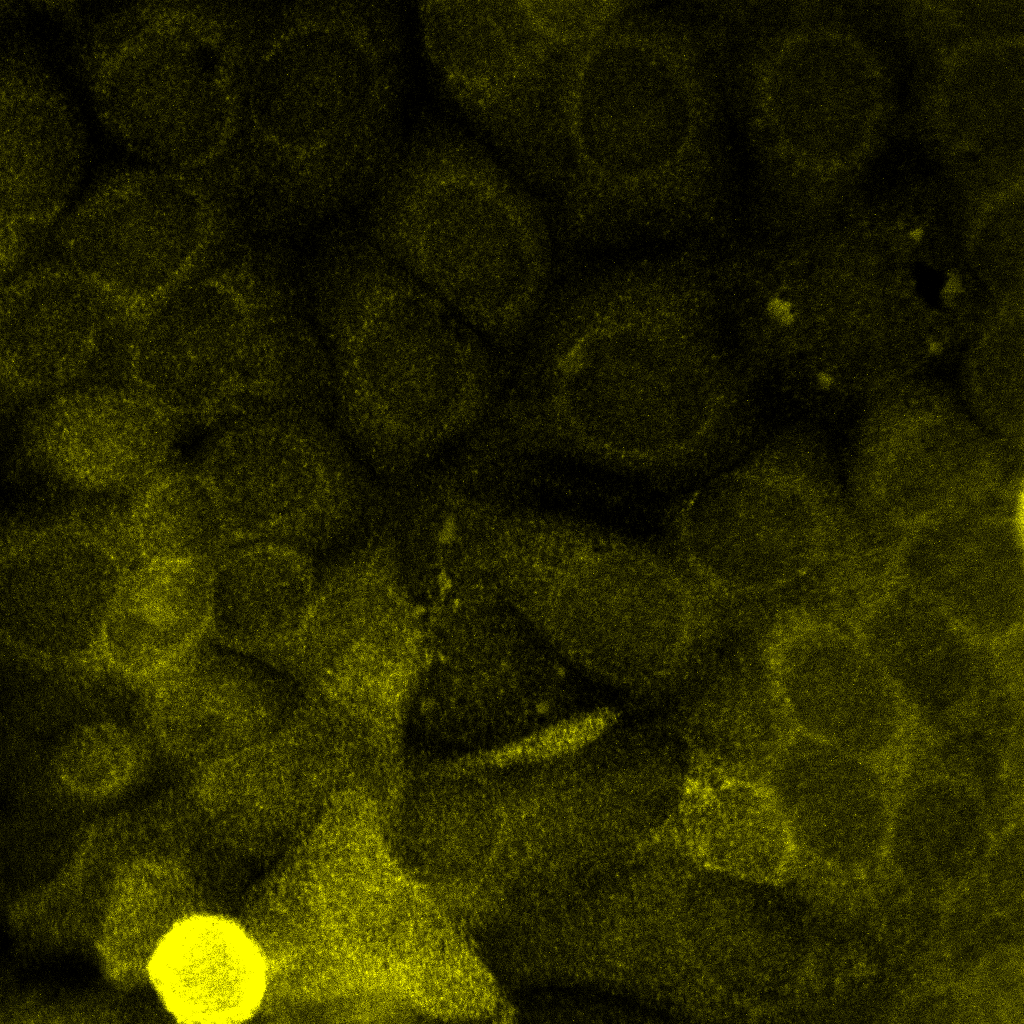

Supplement: Supplementary file 11 — Source Data Fig. 3 [file 44321_2024_32_MOESM11_ESM.zip › Figure 3/Figure 3C ZZW-115+Arsenate Flag NUPR1-Flag Dox.tif]

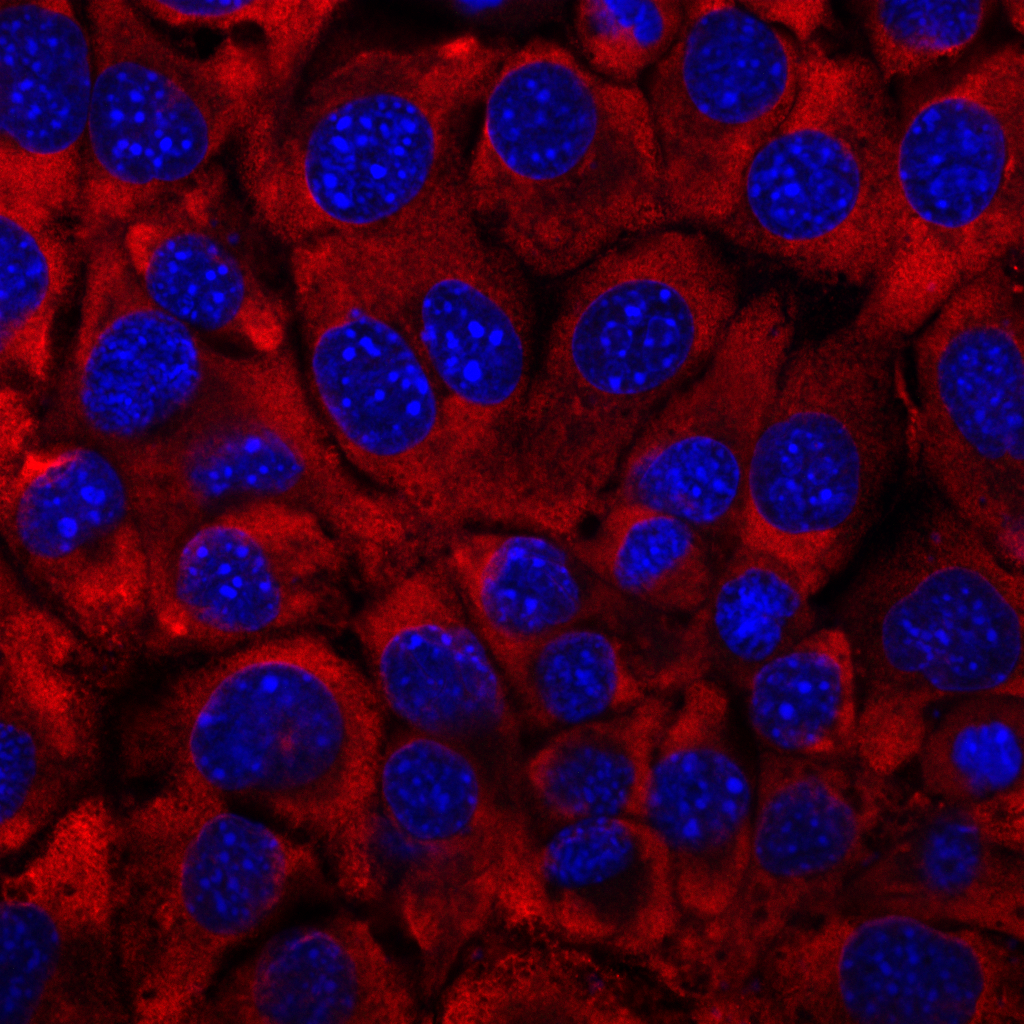

Supplement: Supplementary file 11 — Source Data Fig. 3 [file 44321_2024_32_MOESM11_ESM.zip › Figure 3/Figure 3C ZZW-115+Arsenate Merge GFP Dox.tif]

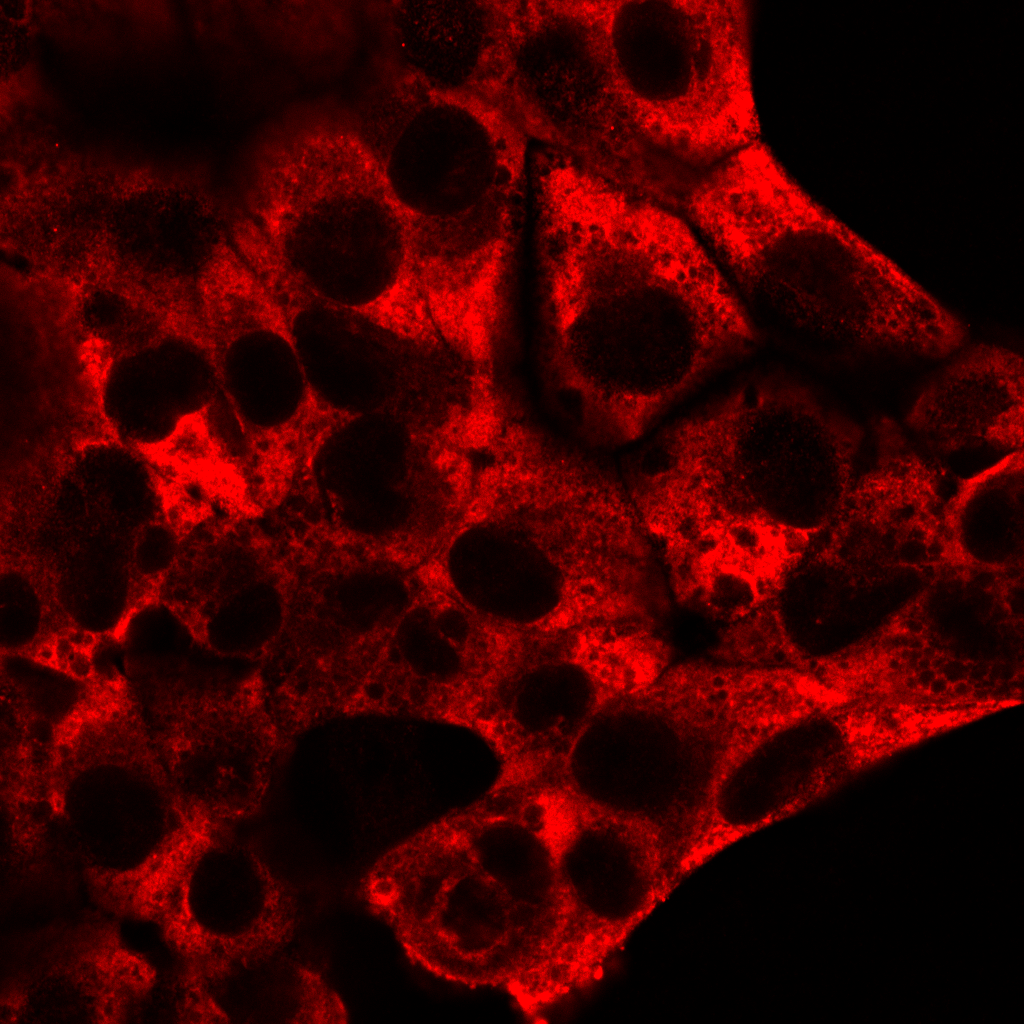

Supplement: Supplementary file 11 — Source Data Fig. 3 [file 44321_2024_32_MOESM11_ESM.zip › Figure 3/Figure 3C Arsenate G3BP1 NUPR1mut-Flag Dox.tif]

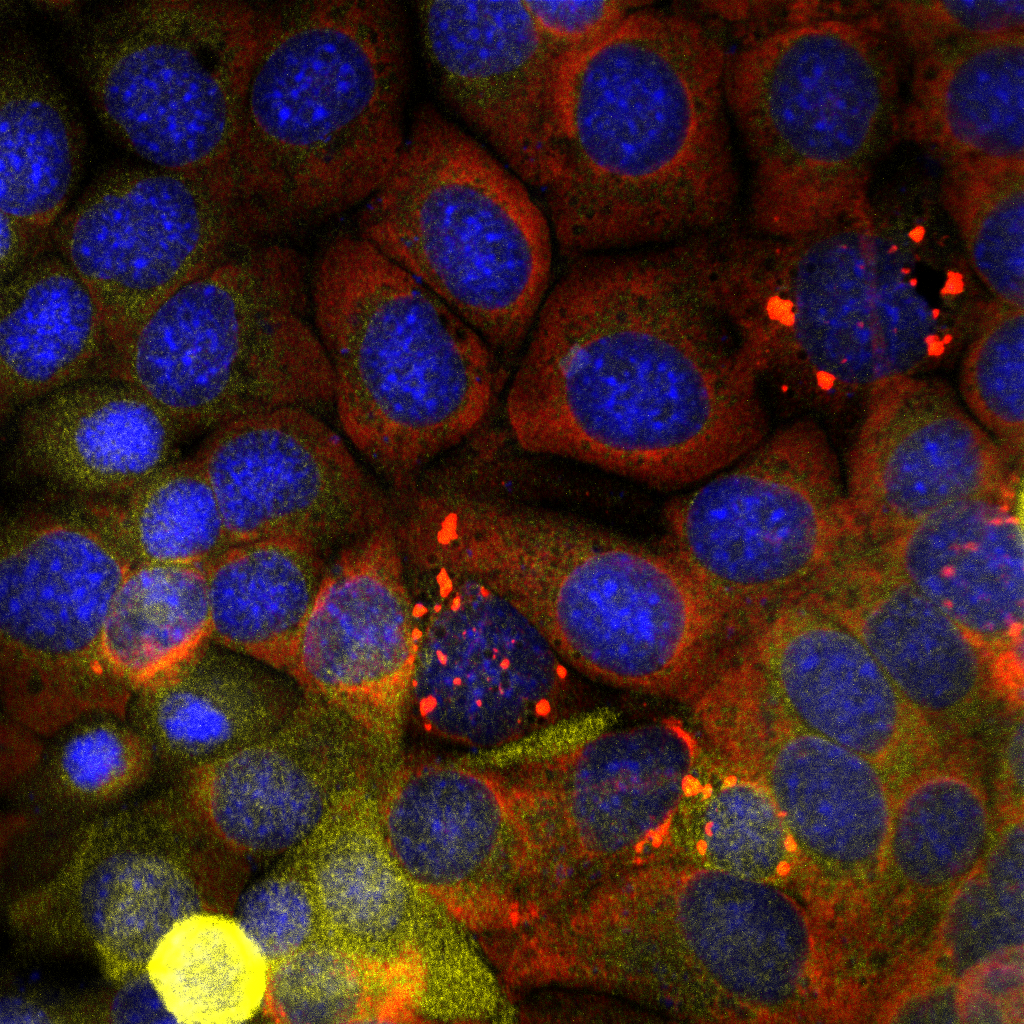

Supplement: Supplementary file 11 — Source Data Fig. 3 [file 44321_2024_32_MOESM11_ESM.zip › Figure 3/Figure 3C ZZW-115+Arsenate Merge NUPR1-Flag Dox.tif]

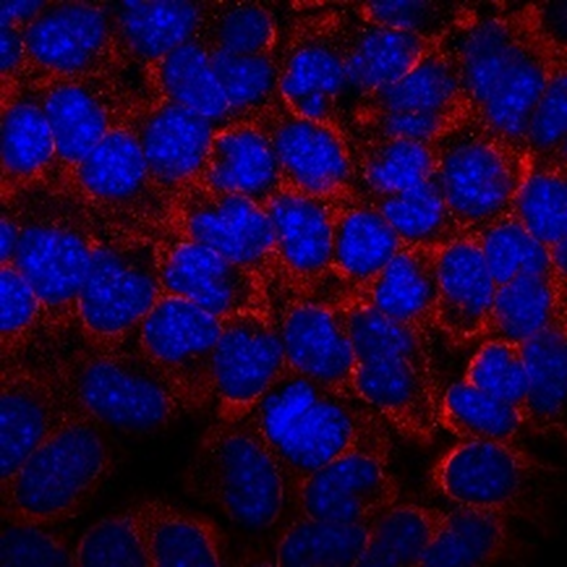

Supplement: Supplementary file 11 — Source Data Fig. 3 [file 44321_2024_32_MOESM11_ESM.zip › Figure 3/Figure 3A 9805 iKras Ar Vehicle.tif]

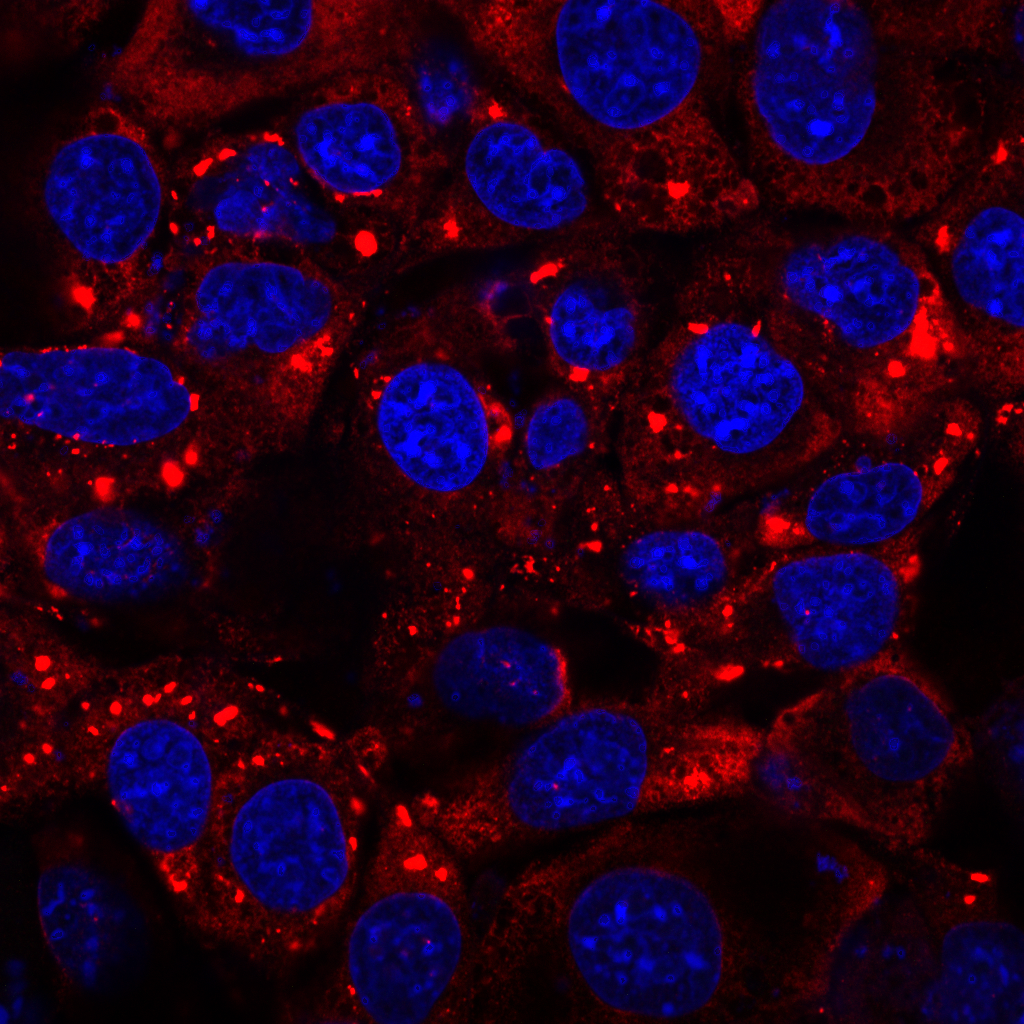

Supplement: Supplementary file 11 — Source Data Fig. 3 [file 44321_2024_32_MOESM11_ESM.zip › Figure 3/Figure 3CArsenate Merge GFP Dox.tif]

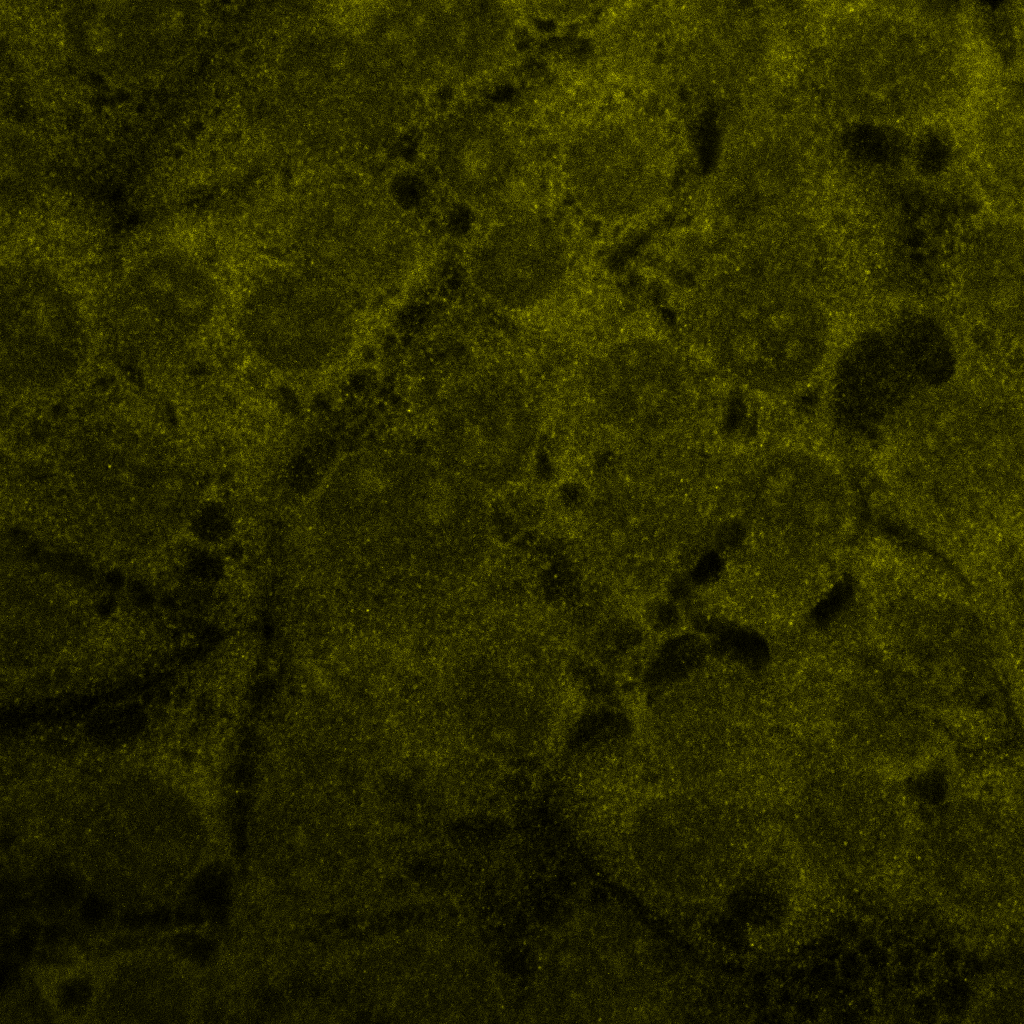

Supplement: Supplementary file 11 — Source Data Fig. 3 [file 44321_2024_32_MOESM11_ESM.zip › Figure 3/Figure 3C ZZW-115+Arsenate Flag NUPR1mut-Flag Vehicle.tif]

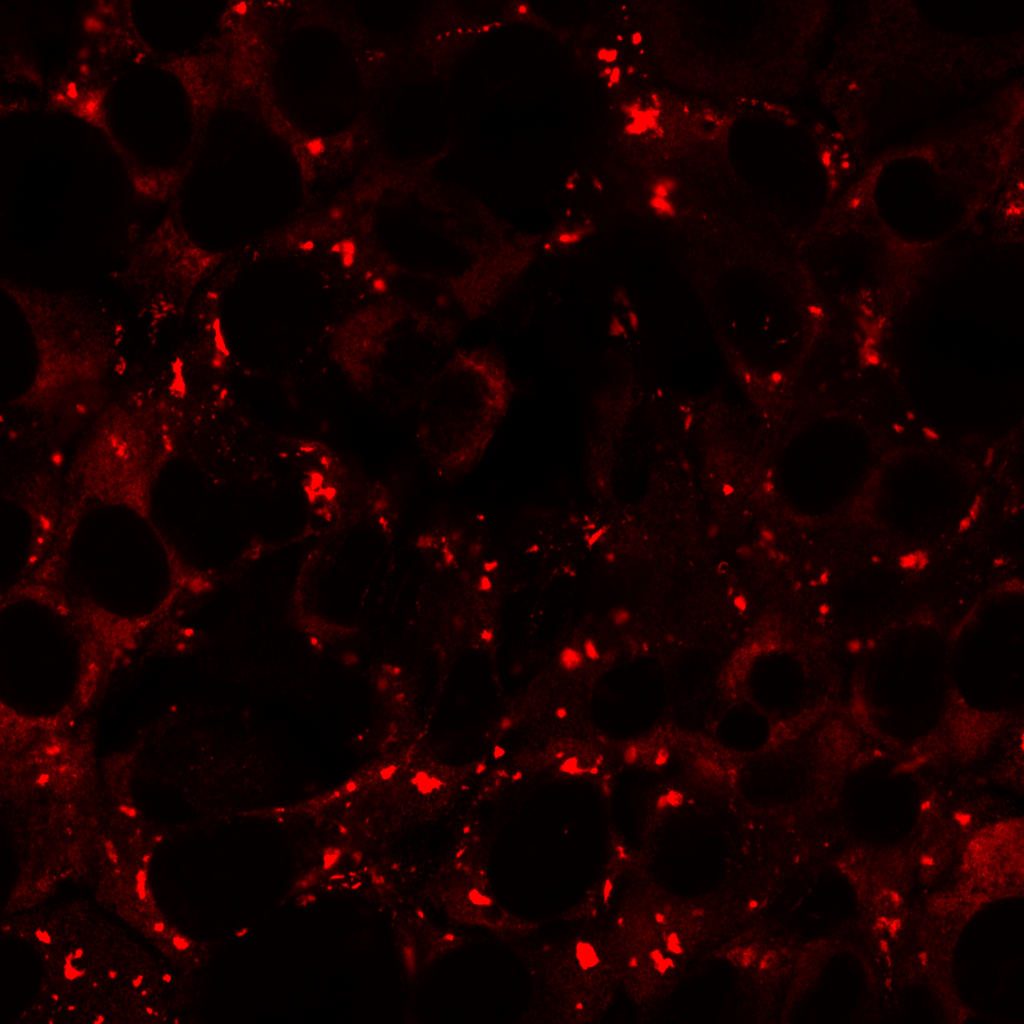

Supplement: Supplementary file 11 — Source Data Fig. 3 [file 44321_2024_32_MOESM11_ESM.zip › Figure 3/Figure 3C Arsenate G3BP1 NUPR1-Flag Dox.tif]

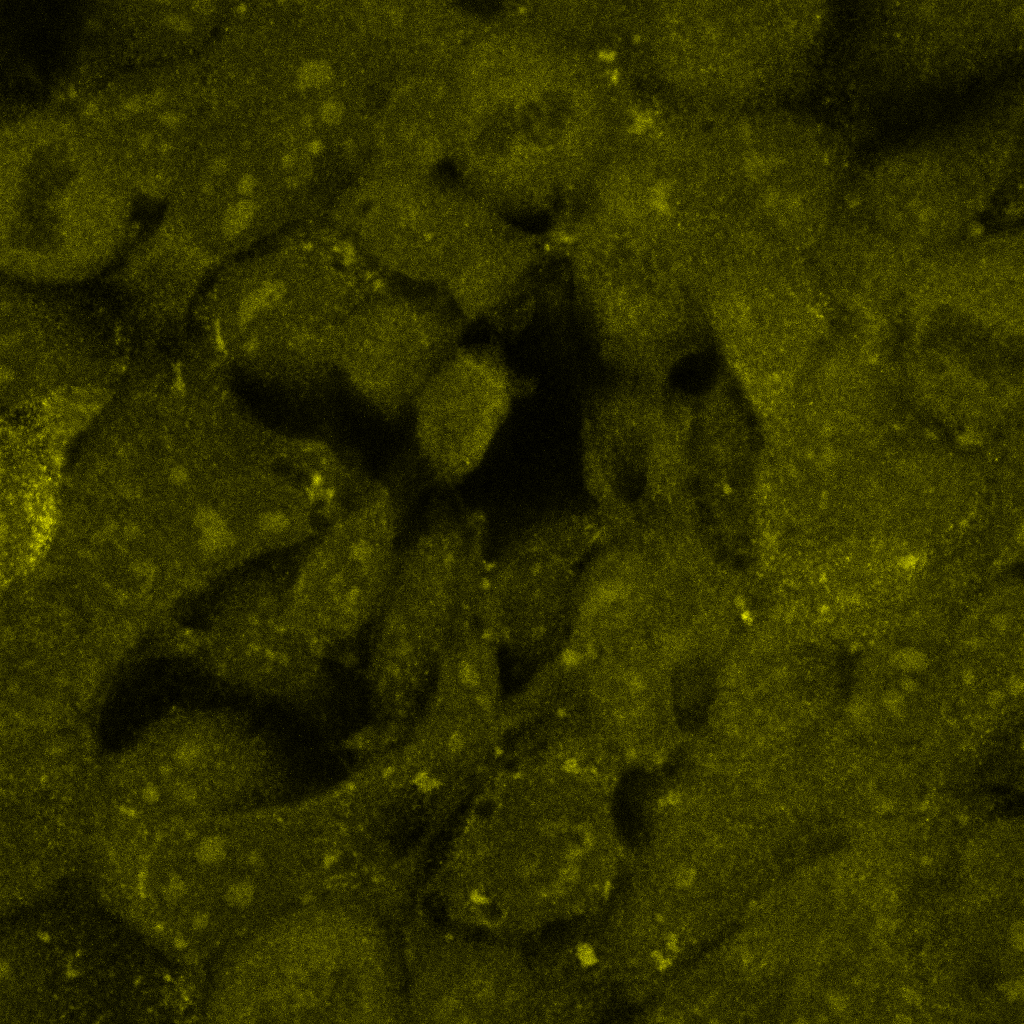

Supplement: Supplementary file 11 — Source Data Fig. 3 [file 44321_2024_32_MOESM11_ESM.zip › Figure 3/Figure 3C Arsenate Flag NUPR1-Flag Dox.tif]

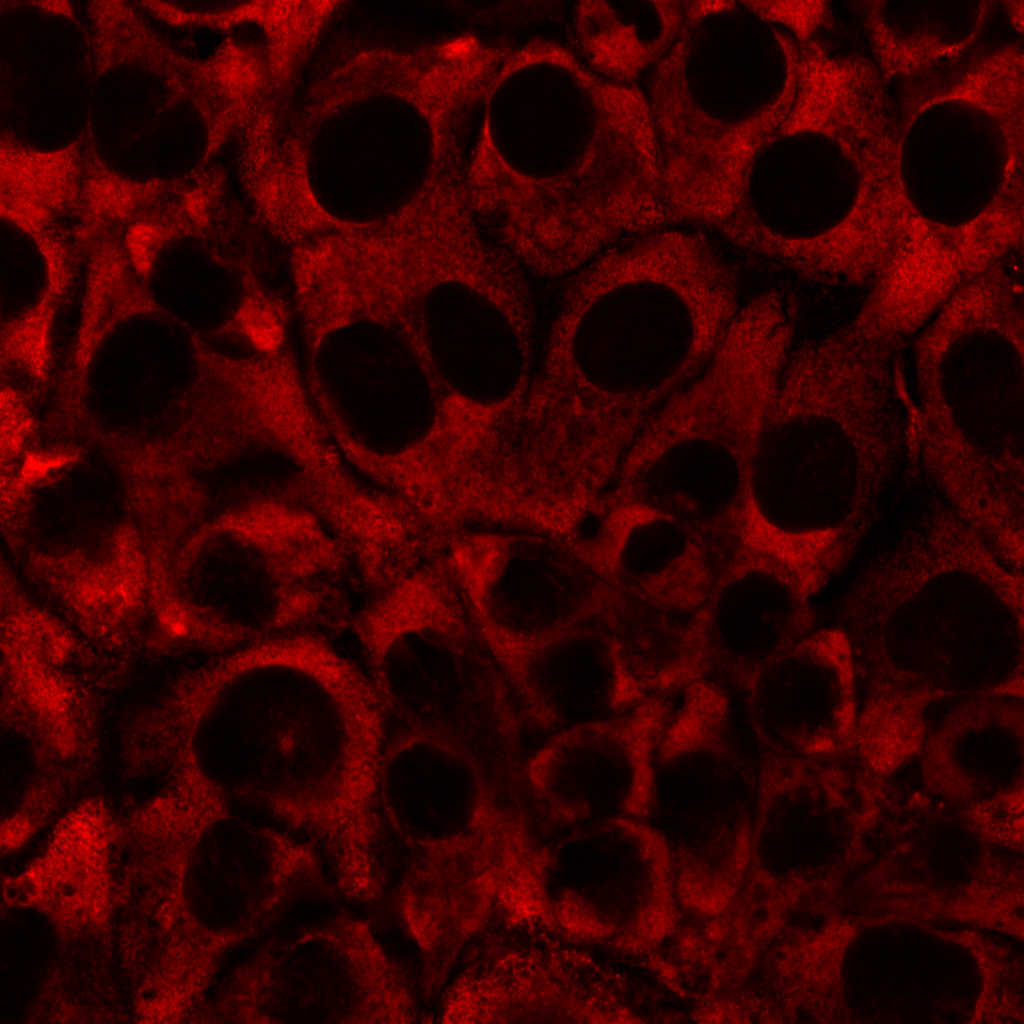

Supplement: Supplementary file 11 — Source Data Fig. 3 [file 44321_2024_32_MOESM11_ESM.zip › Figure 3/Figure 3C ZZW-115+Arsenate G3BP1 GFP Dox.tif]

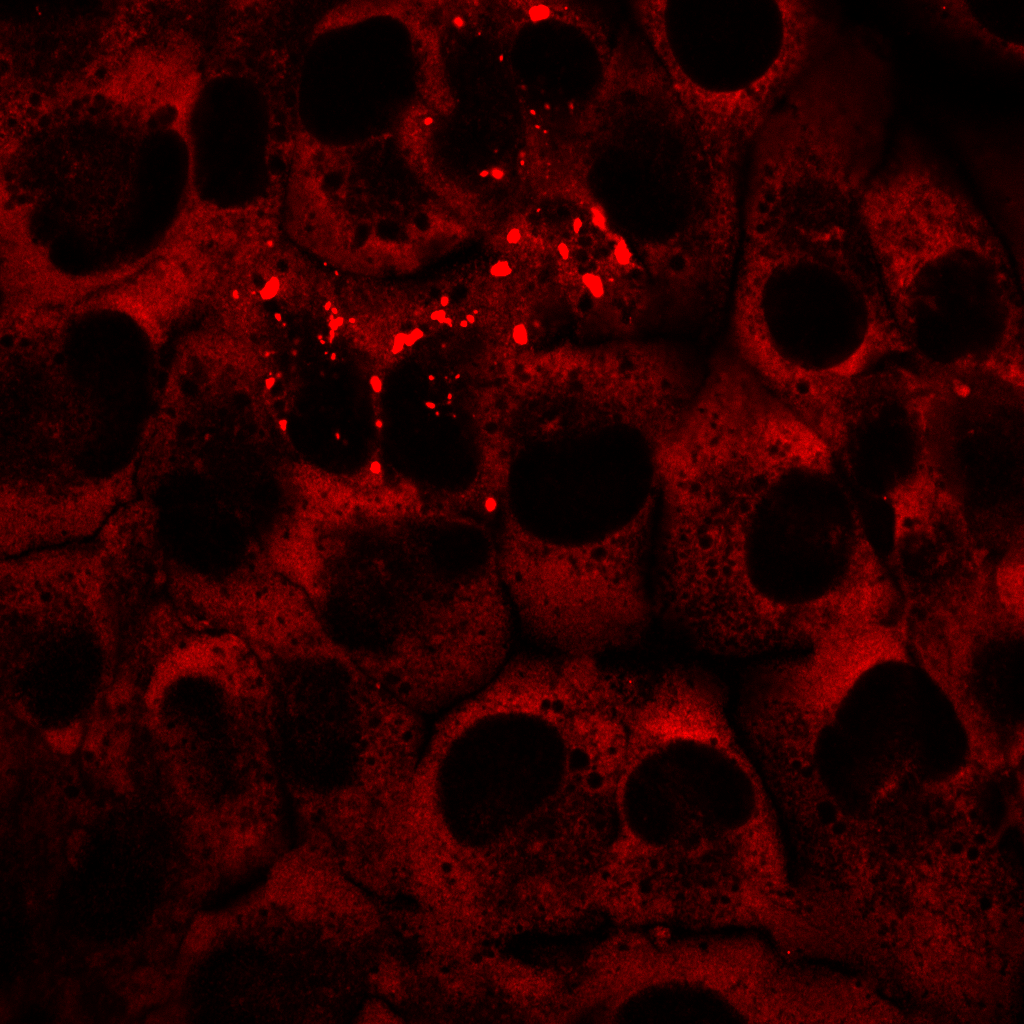

Supplement: Supplementary file 11 — Source Data Fig. 3 [file 44321_2024_32_MOESM11_ESM.zip › Figure 3/Figure 3C ZZW-115+Arsenate G3BP1 NUPR1-Flag Vehicle.tif]

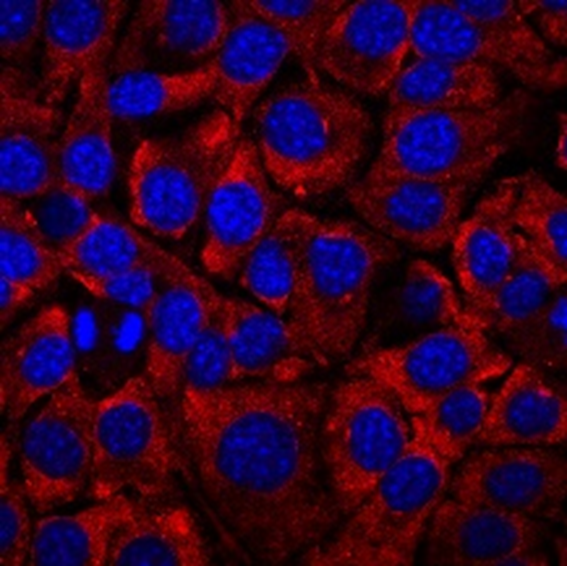

Supplement: Supplementary file 11 — Source Data Fig. 3 [file 44321_2024_32_MOESM11_ESM.zip › Figure 3/Figure 3A 4292 iKras Control Dox.tif]

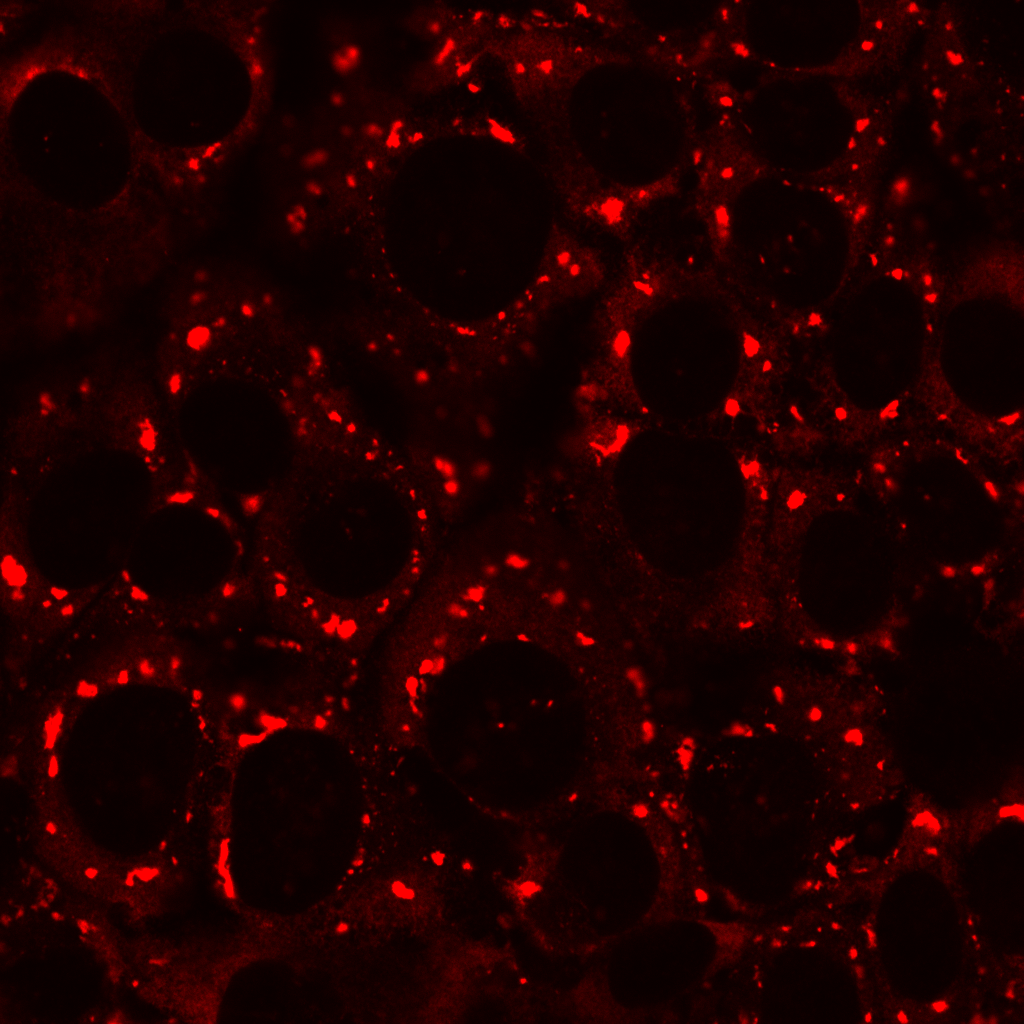

Supplement: Supplementary file 11 — Source Data Fig. 3 [file 44321_2024_32_MOESM11_ESM.zip › Figure 3/Figure 3C Arsenate G3BP1 NUPR1-Flag Vehicle.tif]

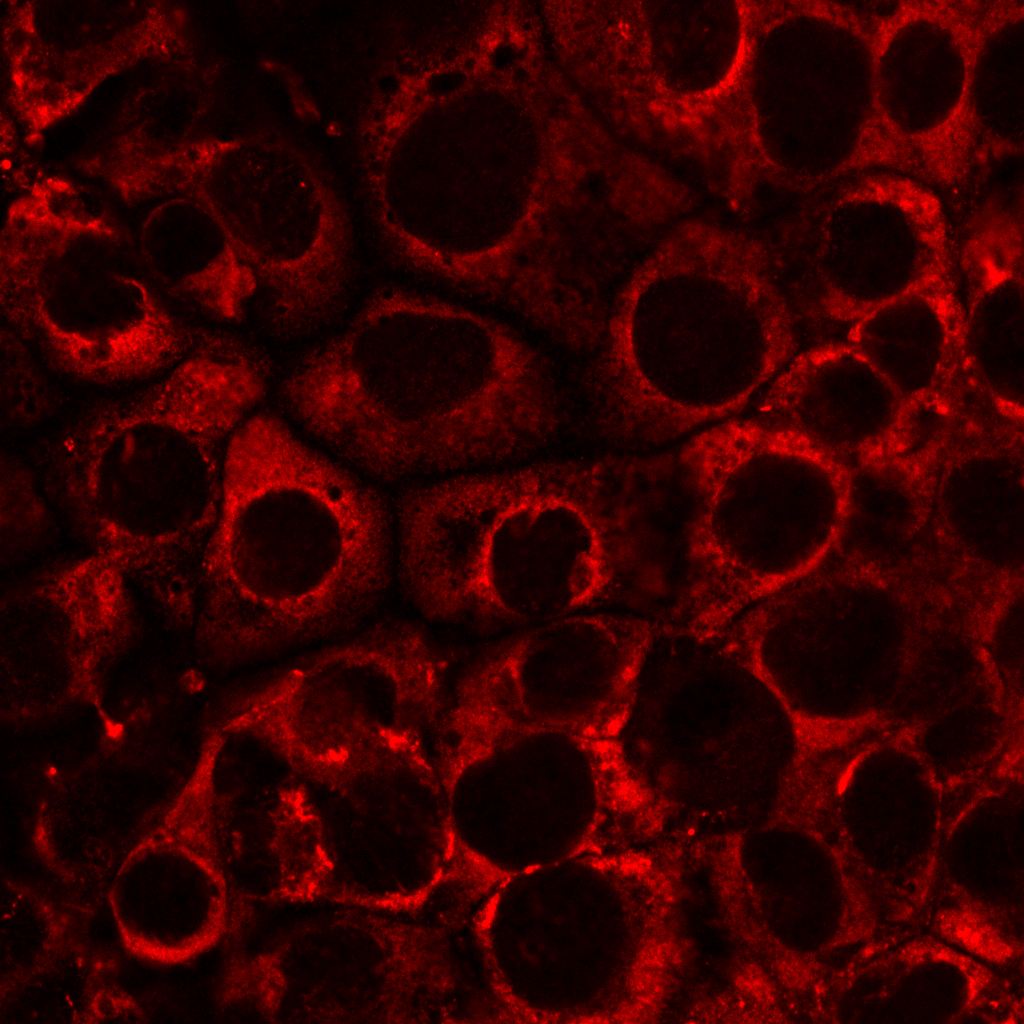

Supplement: Supplementary file 11 — Source Data Fig. 3 [file 44321_2024_32_MOESM11_ESM.zip › Figure 3/Figure 3C ZZW-115+Arsenate G3BP1 GFP Vehicle.tif]

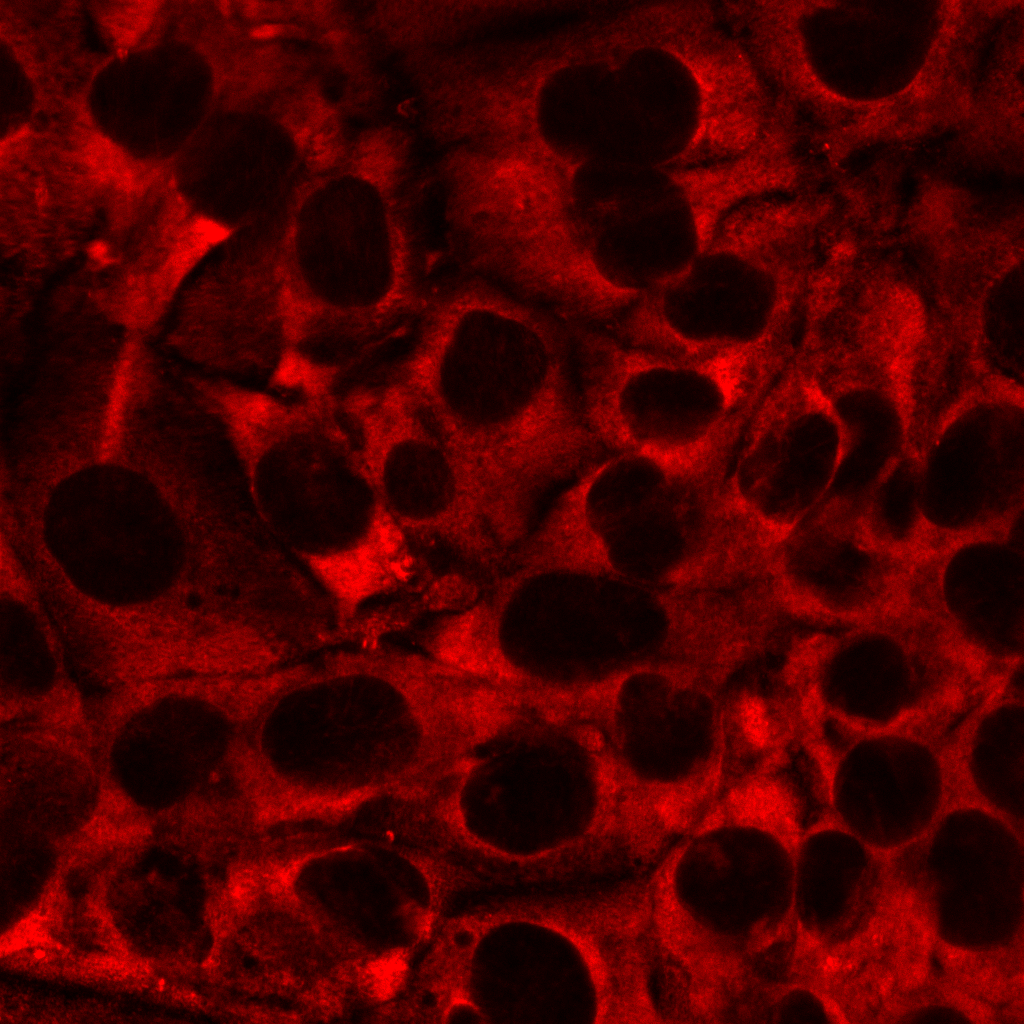

Supplement: Supplementary file 11 — Source Data Fig. 3 [file 44321_2024_32_MOESM11_ESM.zip › Figure 3/Figure 3C Untrated G3BP1 GFP Dox.tif]

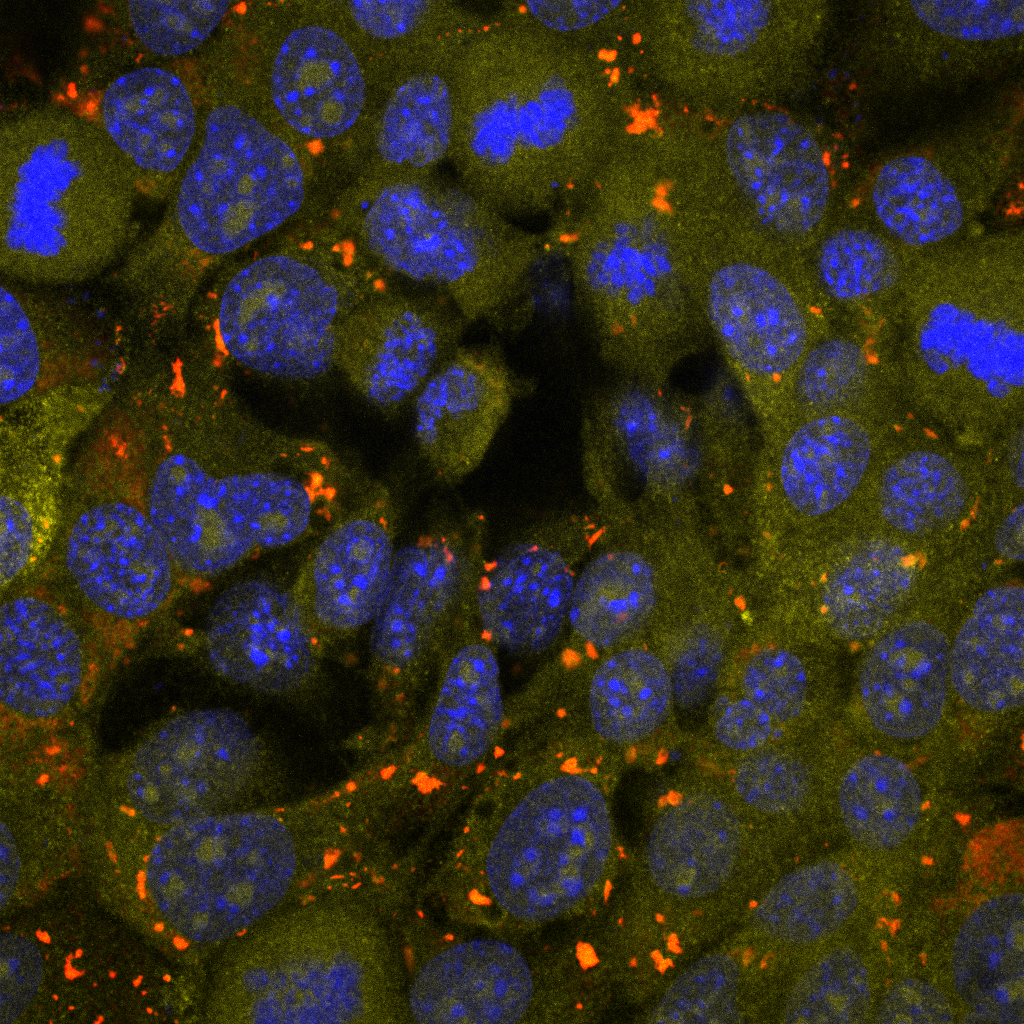

Supplement: Supplementary file 11 — Source Data Fig. 3 [file 44321_2024_32_MOESM11_ESM.zip › Figure 3/Figure 3C Arsenate Merge NUPR1-Flag Dox.tif]
