# Supplementary figures and images for: Targeting NUPR1-dependent stress granules formation to induce synthetic lethality in KrasG12D-driven tumors (part 2 of 3)
Source: EMBO Mol Med. 2024 Feb 15;16(3):4. doi: 10.1038/s44321-024-00032-2 (PMC10940650; doi:10.1038/s44321-024-00032-2)

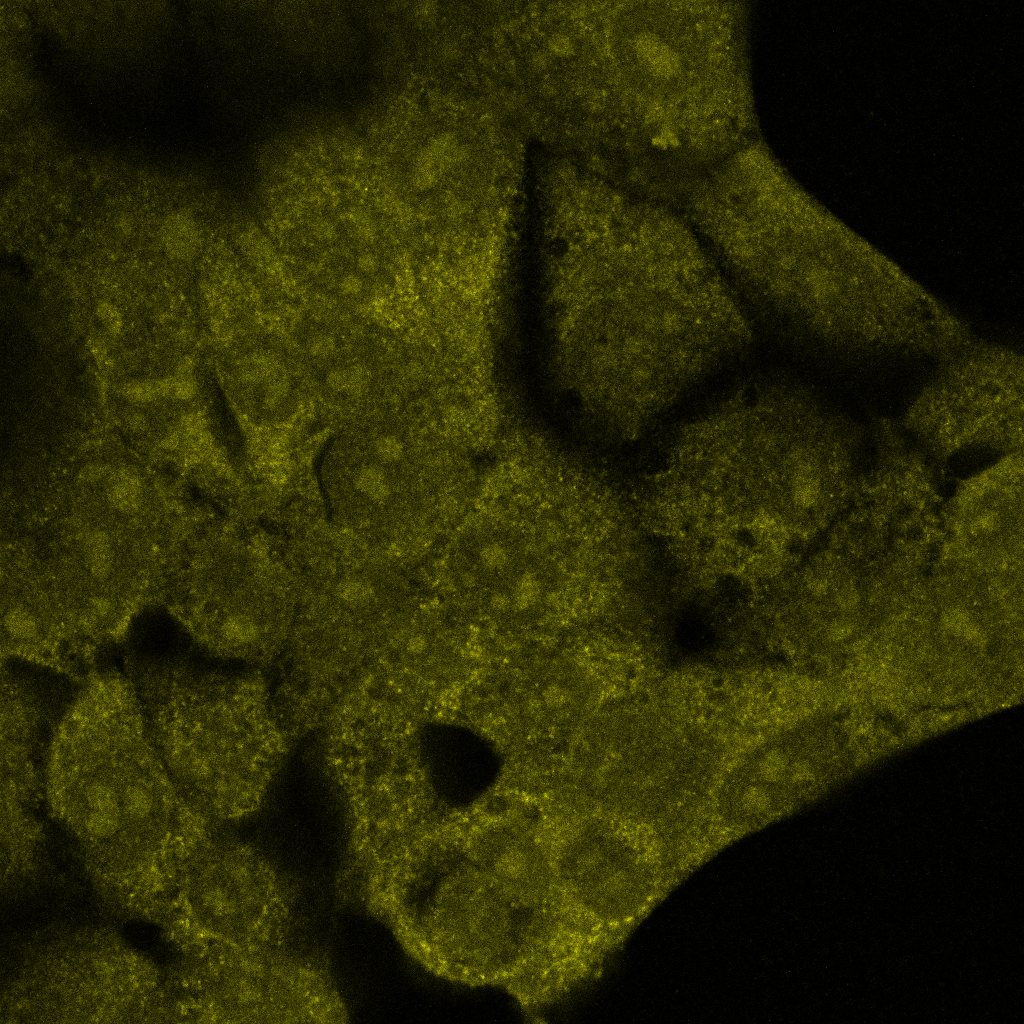

Supplement: Supplementary file 11 — Source Data Fig. 3 [file 44321_2024_32_MOESM11_ESM.zip › Figure 3/Figure 3C Arsenate Flag NUPR1mut-Flag Dox.tif]

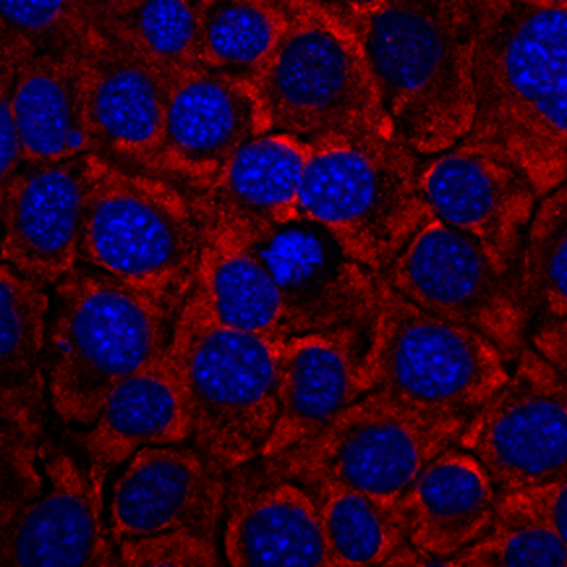

Supplement: Supplementary file 11 — Source Data Fig. 3 [file 44321_2024_32_MOESM11_ESM.zip › Figure 3/Figure 3A 4292 iKras Ar Vehicle.tif]

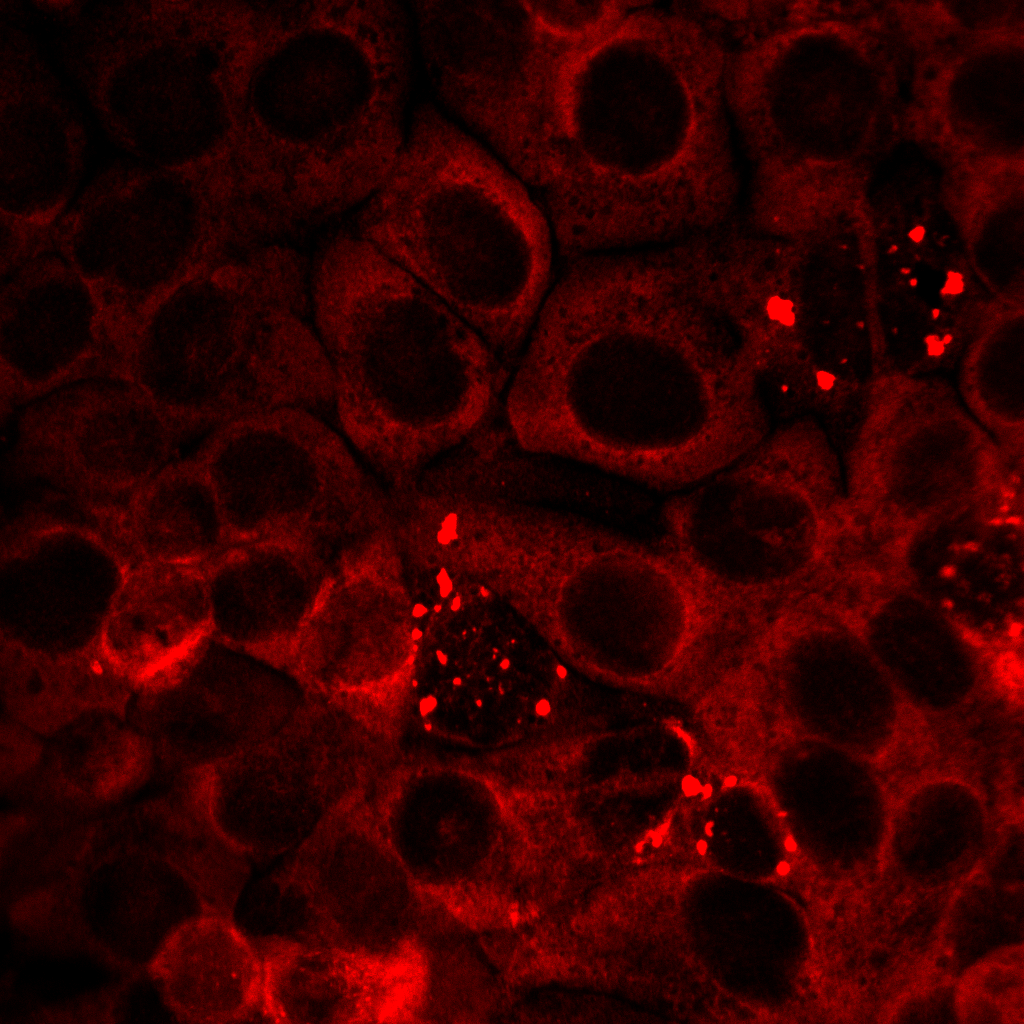

Supplement: Supplementary file 11 — Source Data Fig. 3 [file 44321_2024_32_MOESM11_ESM.zip › Figure 3/Figure 3C ZZW-115+Arsenate G3BP1 NUPR1-Flag Dox.tif]

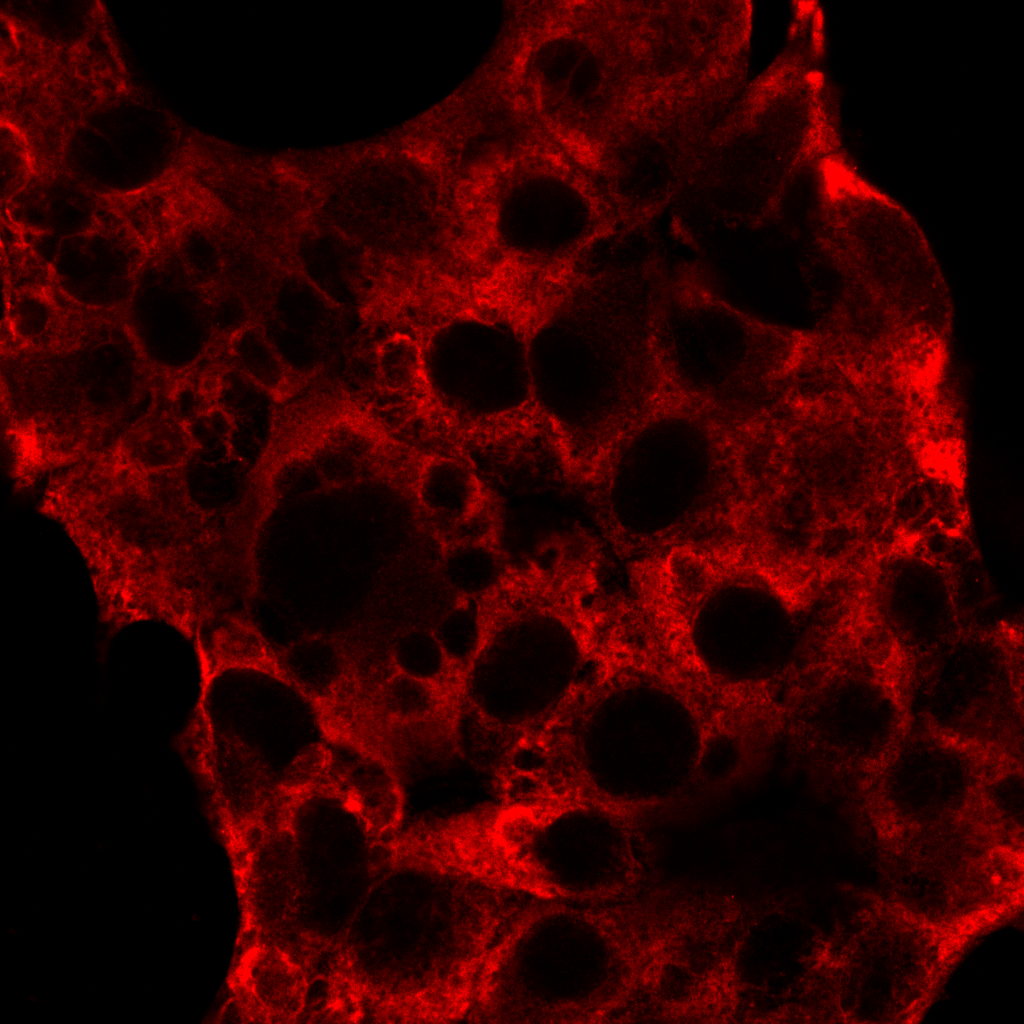

Supplement: Supplementary file 11 — Source Data Fig. 3 [file 44321_2024_32_MOESM11_ESM.zip › Figure 3/Figure 3CArsenate G3BP1 NUPR1mut-Flag Vehicle.tif]

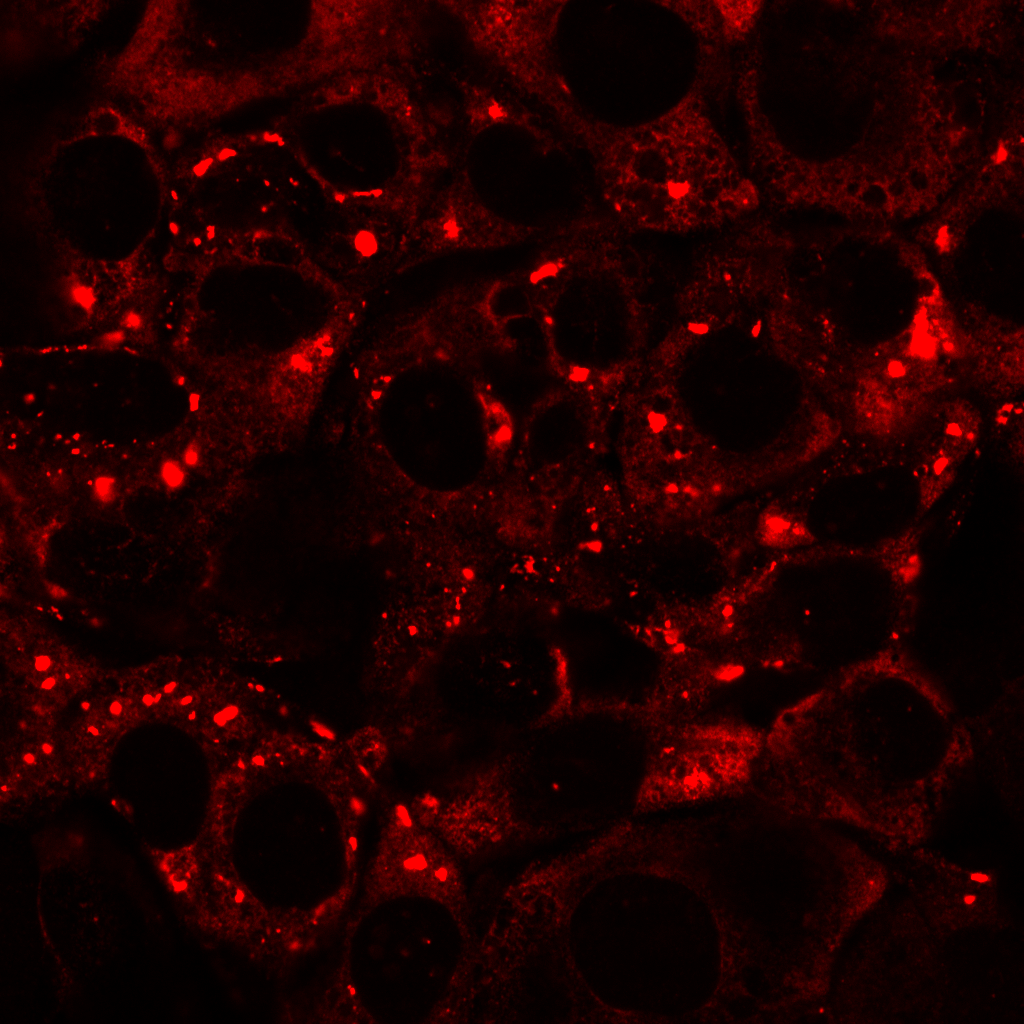

Supplement: Supplementary file 11 — Source Data Fig. 3 [file 44321_2024_32_MOESM11_ESM.zip › Figure 3/Figure 3C Arsenate G3BP1 GFP Dox.tif]

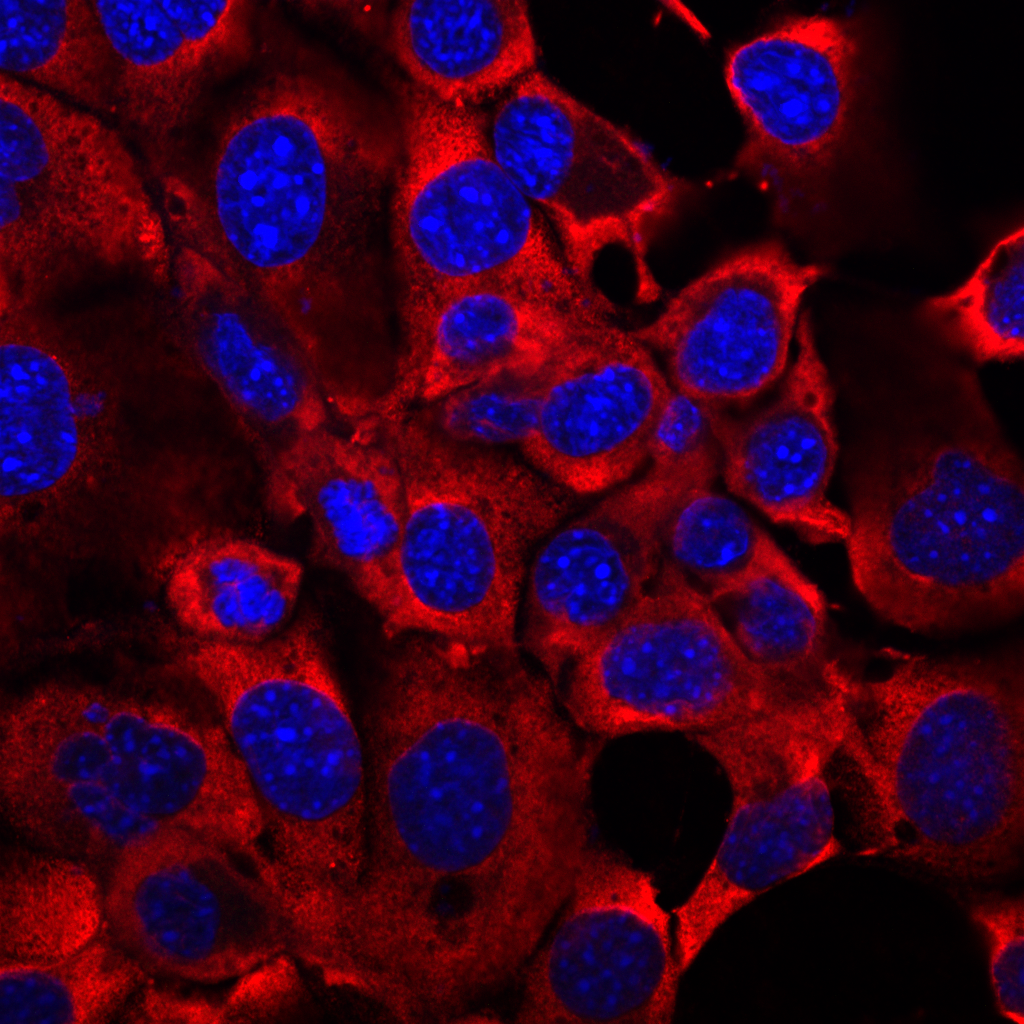

Supplement: Supplementary file 11 — Source Data Fig. 3 [file 44321_2024_32_MOESM11_ESM.zip › Figure 3/Figure 3C Untrated Merge GFP Vehicle.tif]

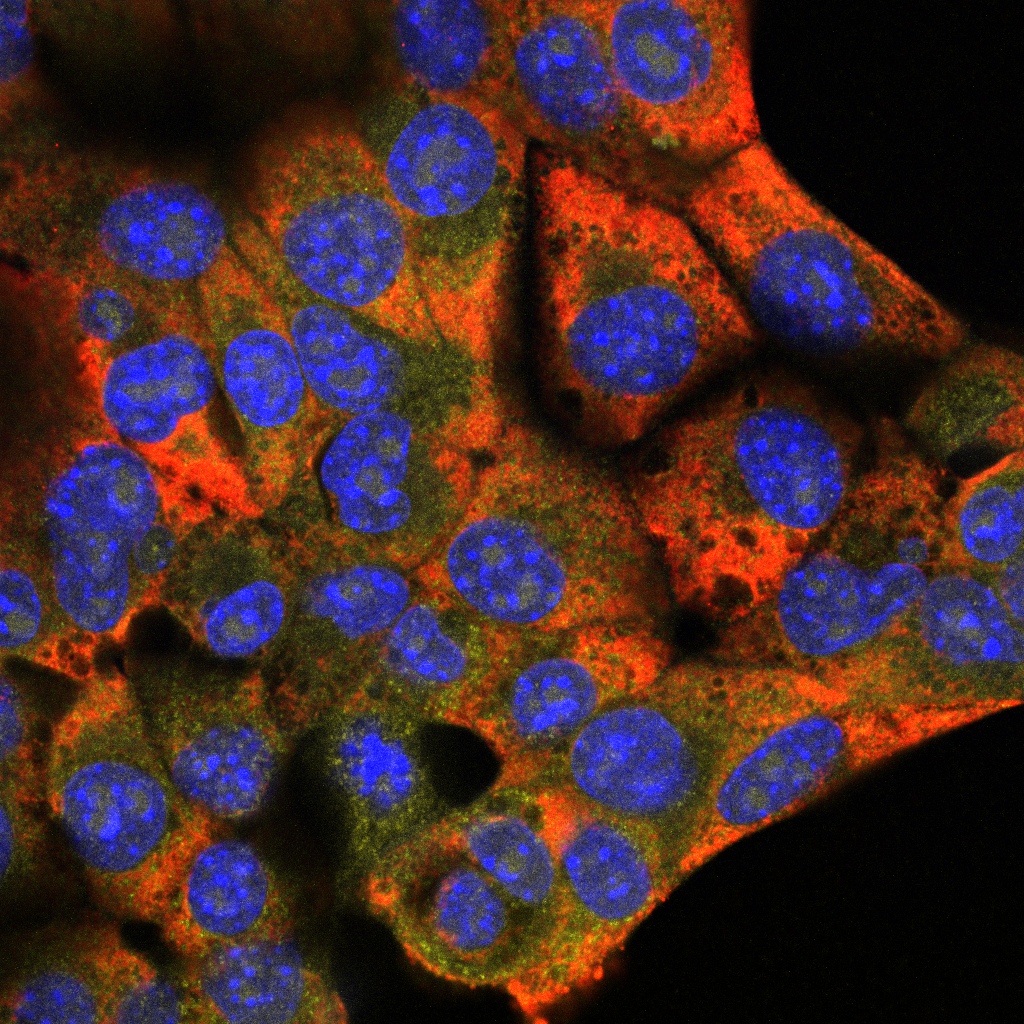

Supplement: Supplementary file 11 — Source Data Fig. 3 [file 44321_2024_32_MOESM11_ESM.zip › Figure 3/Figure 3CArsenate Merge NUPR1mut-Flag Dox.tif]

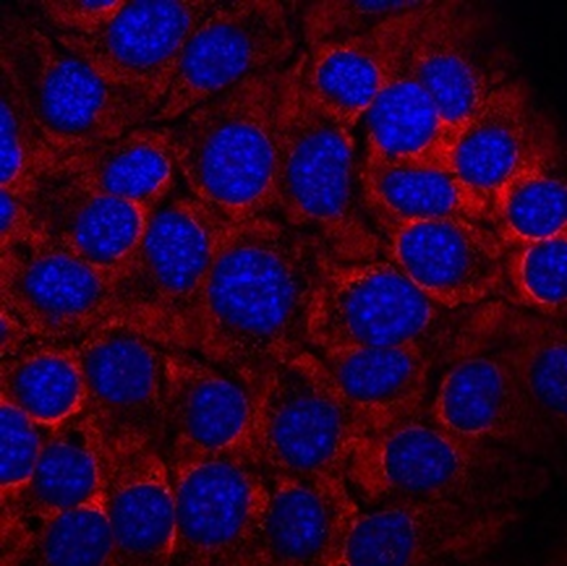

Supplement: Supplementary file 11 — Source Data Fig. 3 [file 44321_2024_32_MOESM11_ESM.zip › Figure 3/Figure 3A 4292 iKras Control Vehicle.tif]

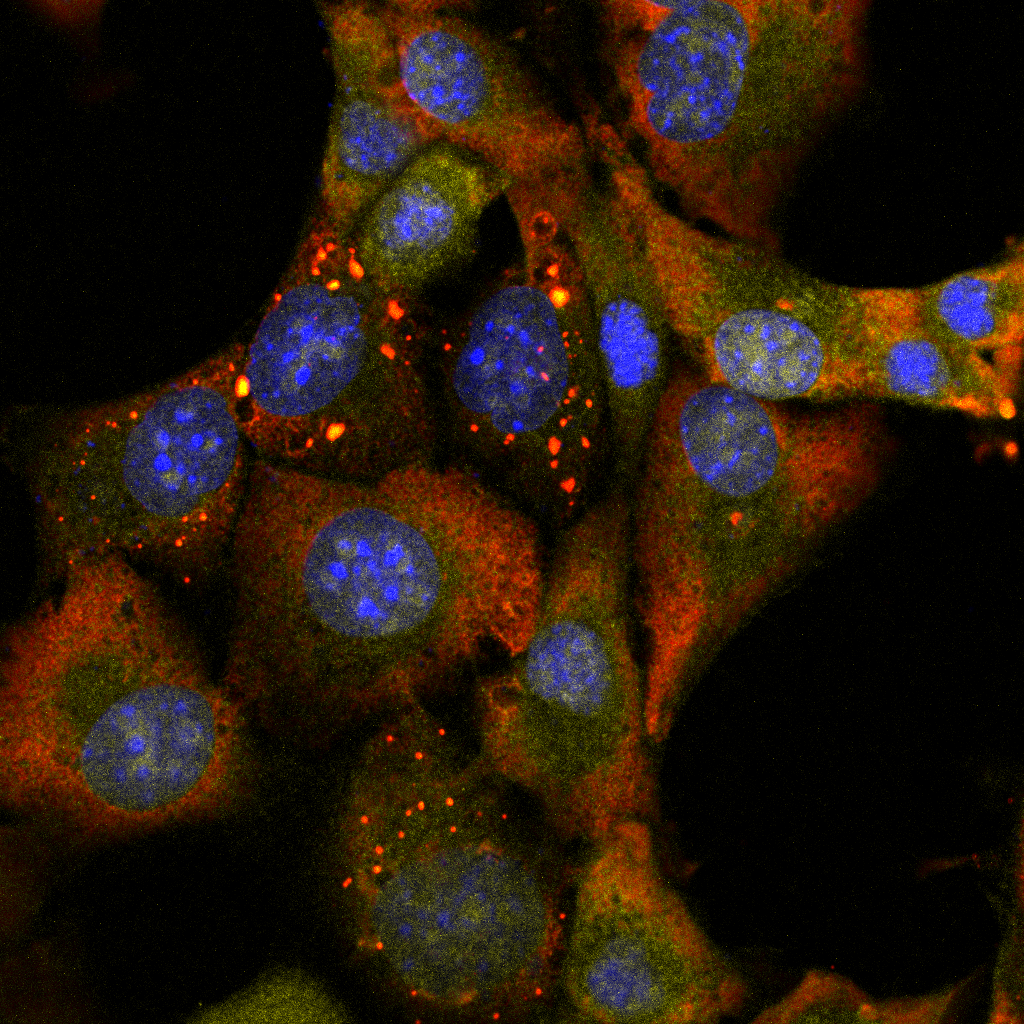

Supplement: Supplementary file 11 — Source Data Fig. 3 [file 44321_2024_32_MOESM11_ESM.zip › Figure 3/Figure 3C Untrated Merge NUPR1-Flag Dox.tif]

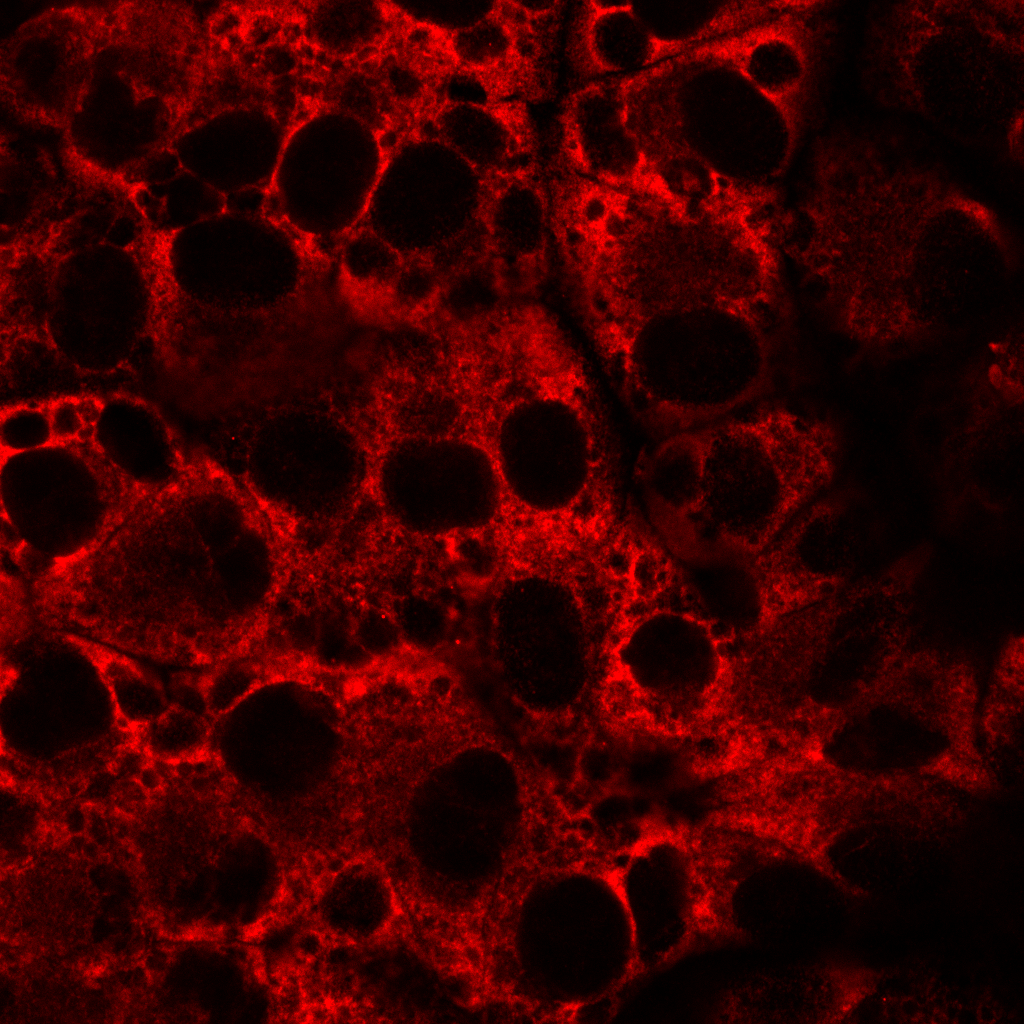

Supplement: Supplementary file 11 — Source Data Fig. 3 [file 44321_2024_32_MOESM11_ESM.zip › Figure 3/Figure 3C ZZW-115+Arsenate G3BP1 NUPR1mut-Flag Dox.tif]

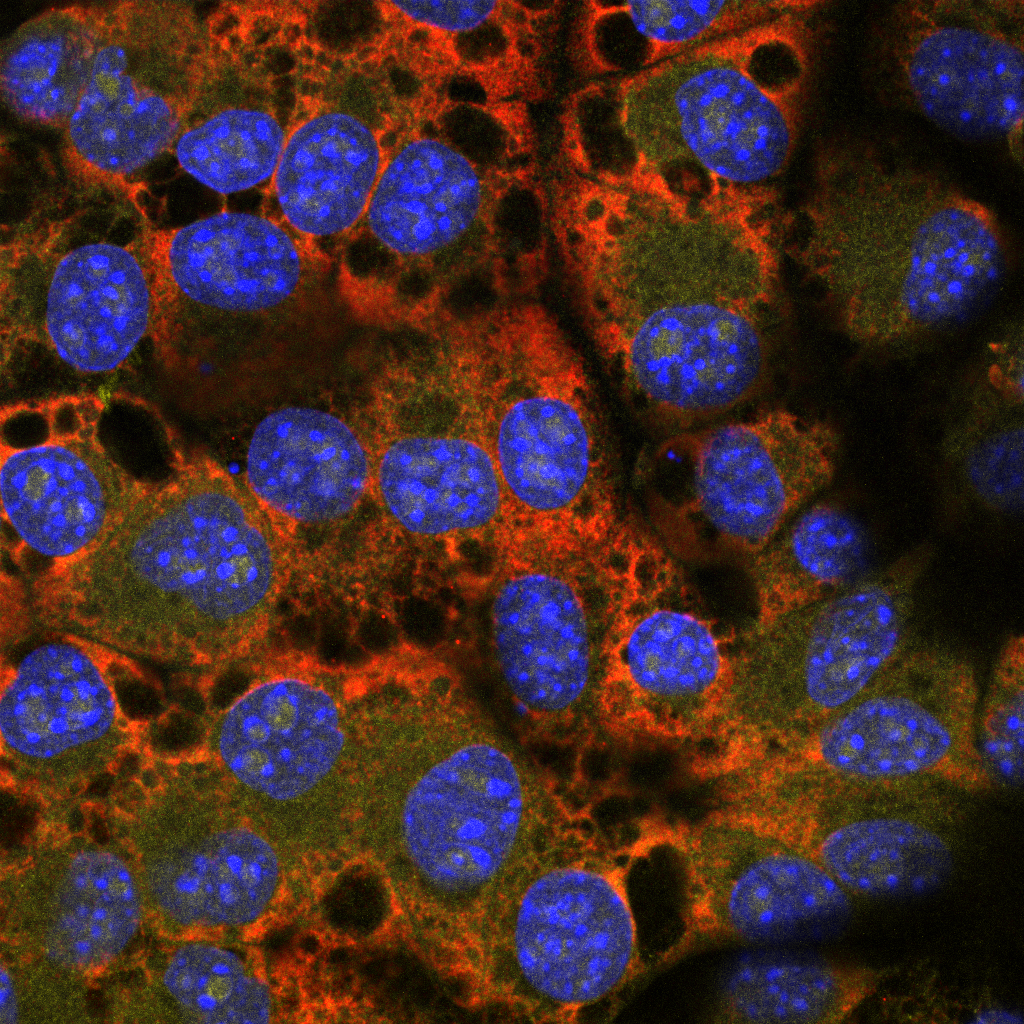

Supplement: Supplementary file 11 — Source Data Fig. 3 [file 44321_2024_32_MOESM11_ESM.zip › Figure 3/Figure 3C ZZW-115+Arsenate Merge NUPR1mut-Flag Dox.tif]

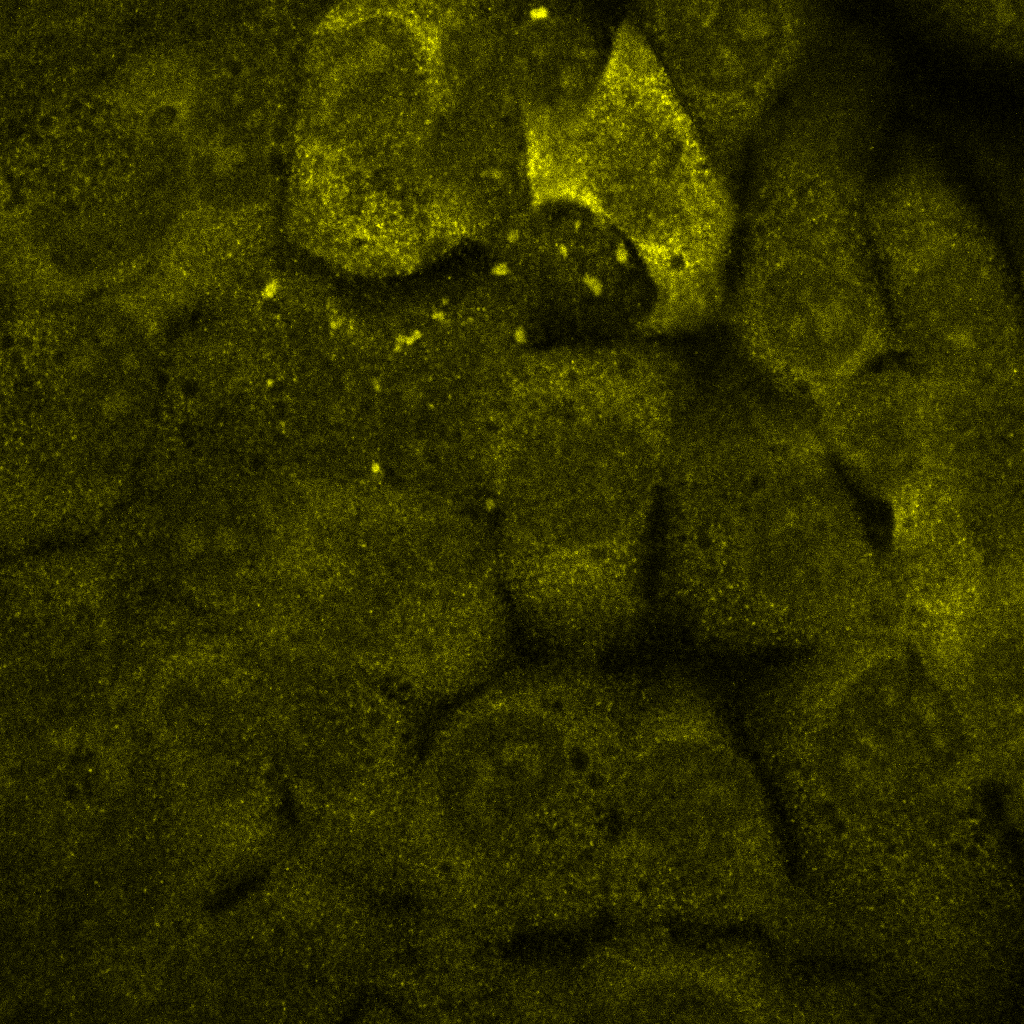

Supplement: Supplementary file 11 — Source Data Fig. 3 [file 44321_2024_32_MOESM11_ESM.zip › Figure 3/Figure 3C ZZW-115+Arsenate Flag NUPR1-Flag Vehicle.tif]

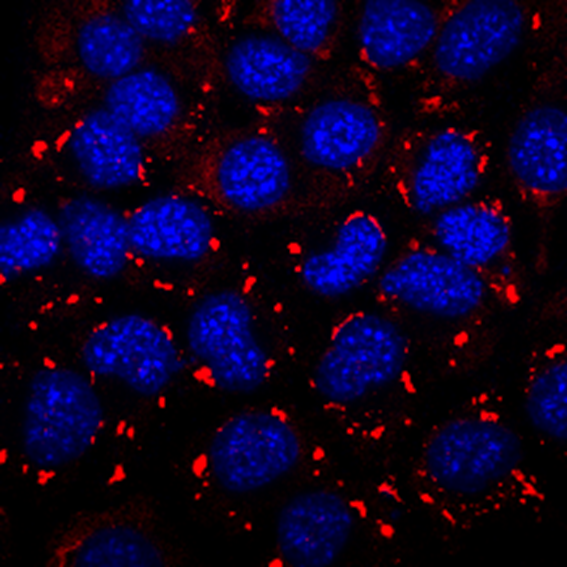

Supplement: Supplementary file 11 — Source Data Fig. 3 [file 44321_2024_32_MOESM11_ESM.zip › Figure 3/Figure 3A 9805 iKras Ar Dox.tif]

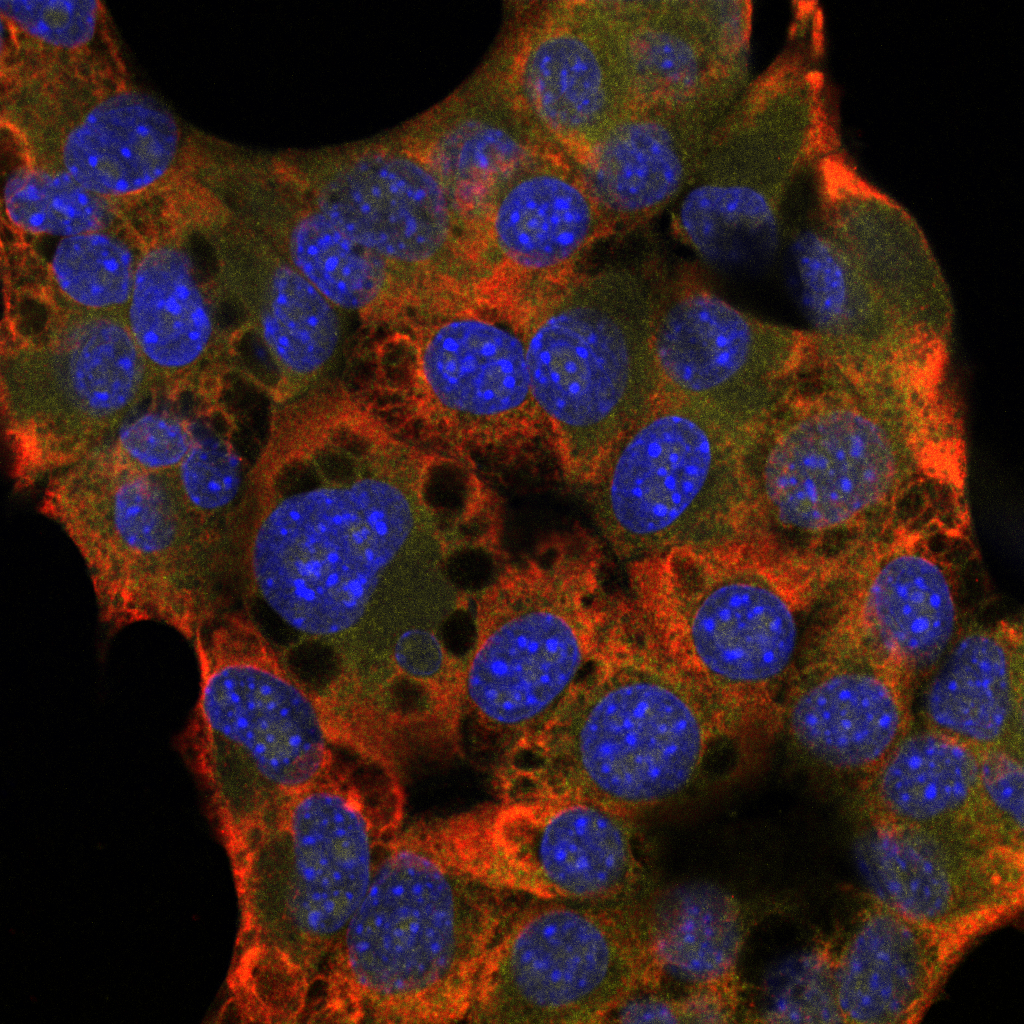

Supplement: Supplementary file 11 — Source Data Fig. 3 [file 44321_2024_32_MOESM11_ESM.zip › Figure 3/Figure 3CArsenate Merge NUPR1mut-Flag Vehicle.tif]

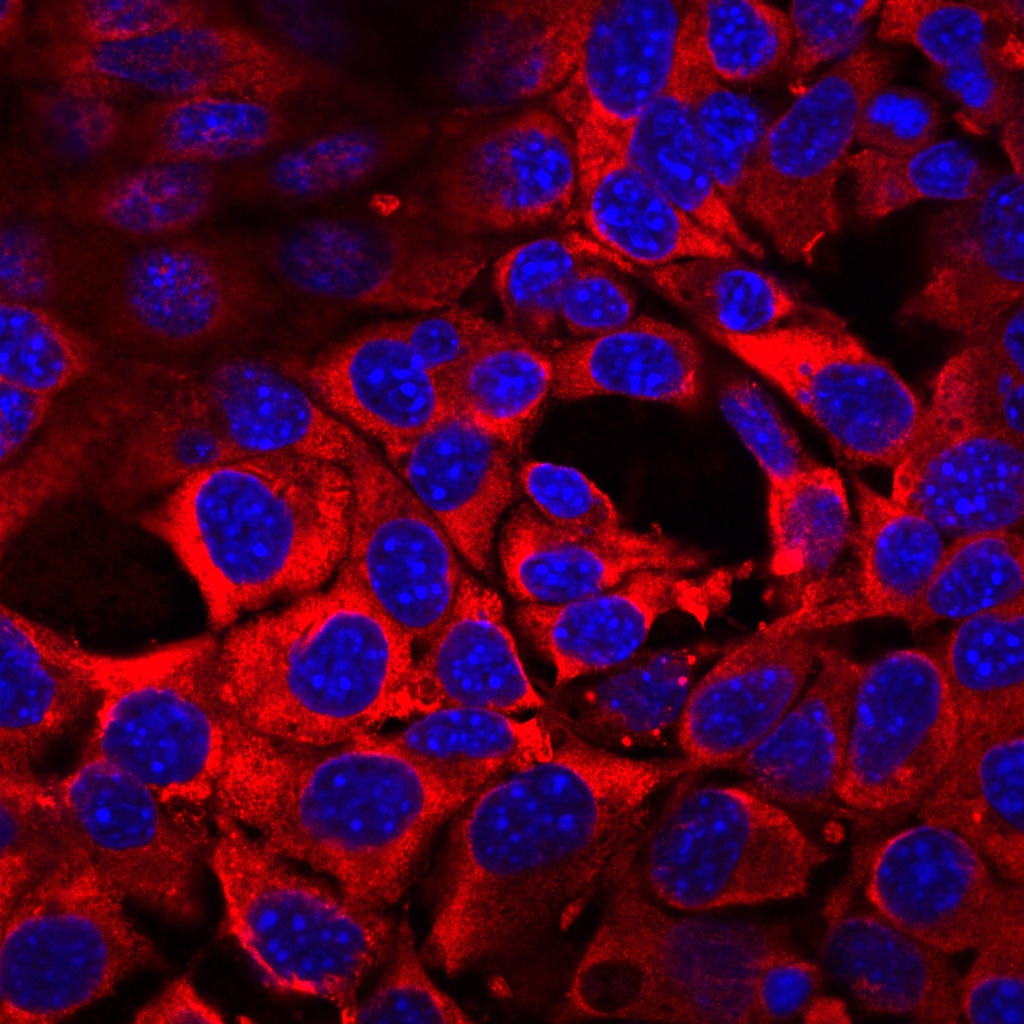

Supplement: Supplementary file 11 — Source Data Fig. 3 [file 44321_2024_32_MOESM11_ESM.zip › Figure 3/Figure 3C Arsenate Merge GFP Vehicle.tif]

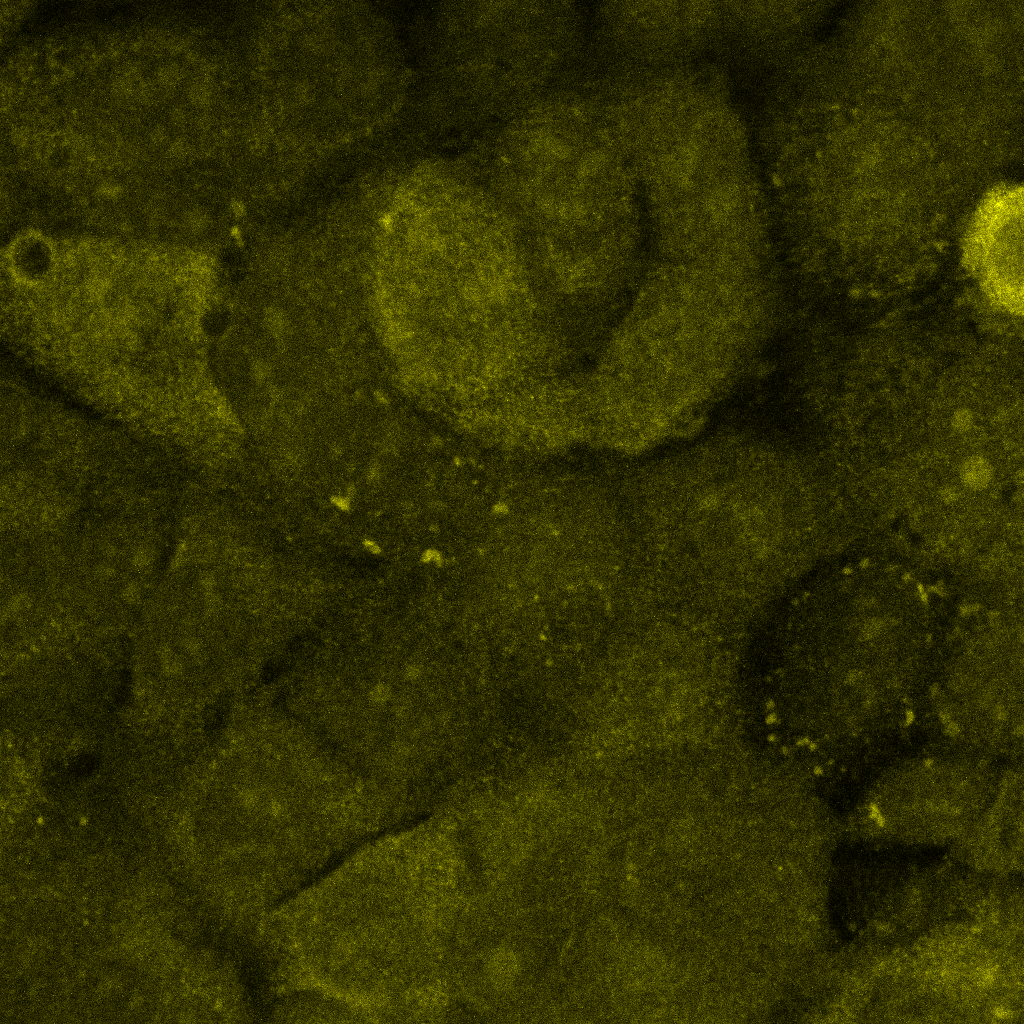

Supplement: Supplementary file 11 — Source Data Fig. 3 [file 44321_2024_32_MOESM11_ESM.zip › Figure 3/Figure 3C Untrated Flag NUPR1-Flag Vehicle.tif]

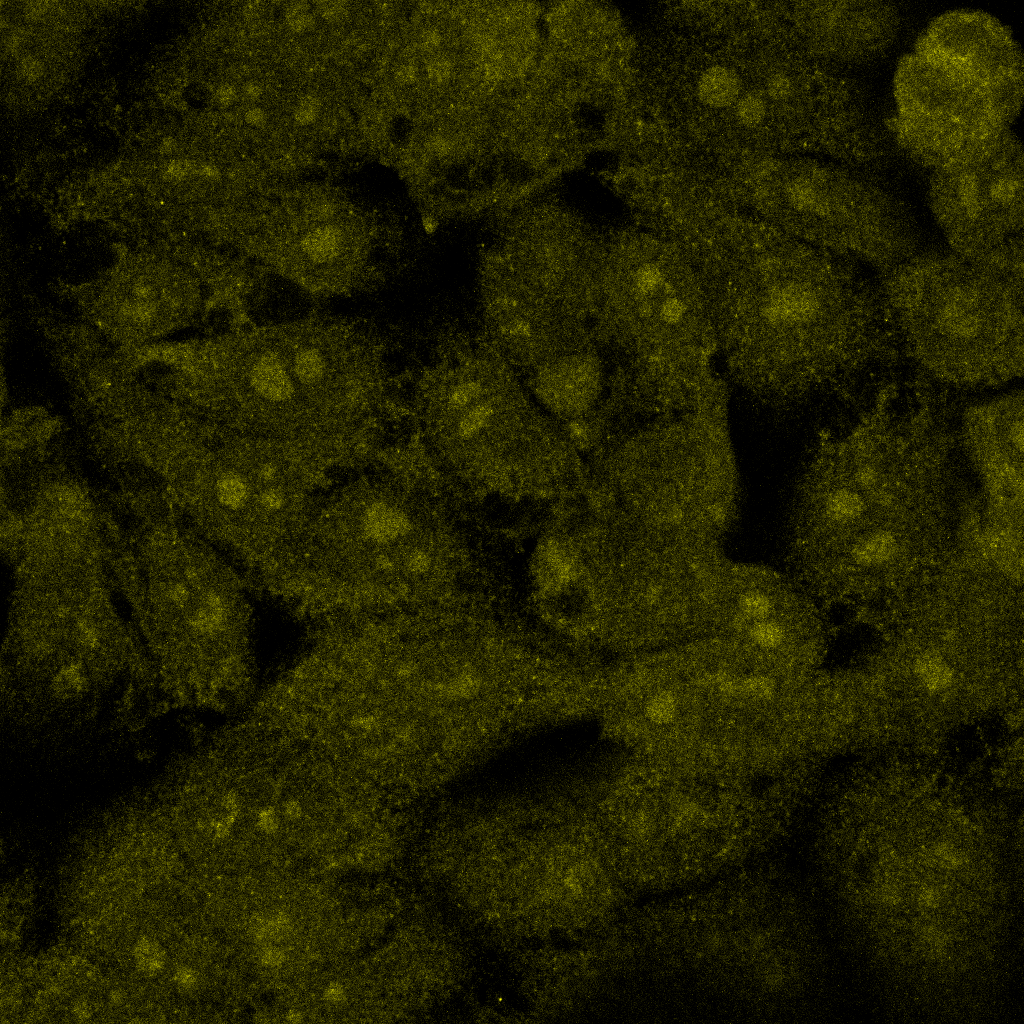

Supplement: Supplementary file 11 — Source Data Fig. 3 [file 44321_2024_32_MOESM11_ESM.zip › Figure 3/Figure 3C Untrated Flag NUPR1mut-Flag Dox.tif]

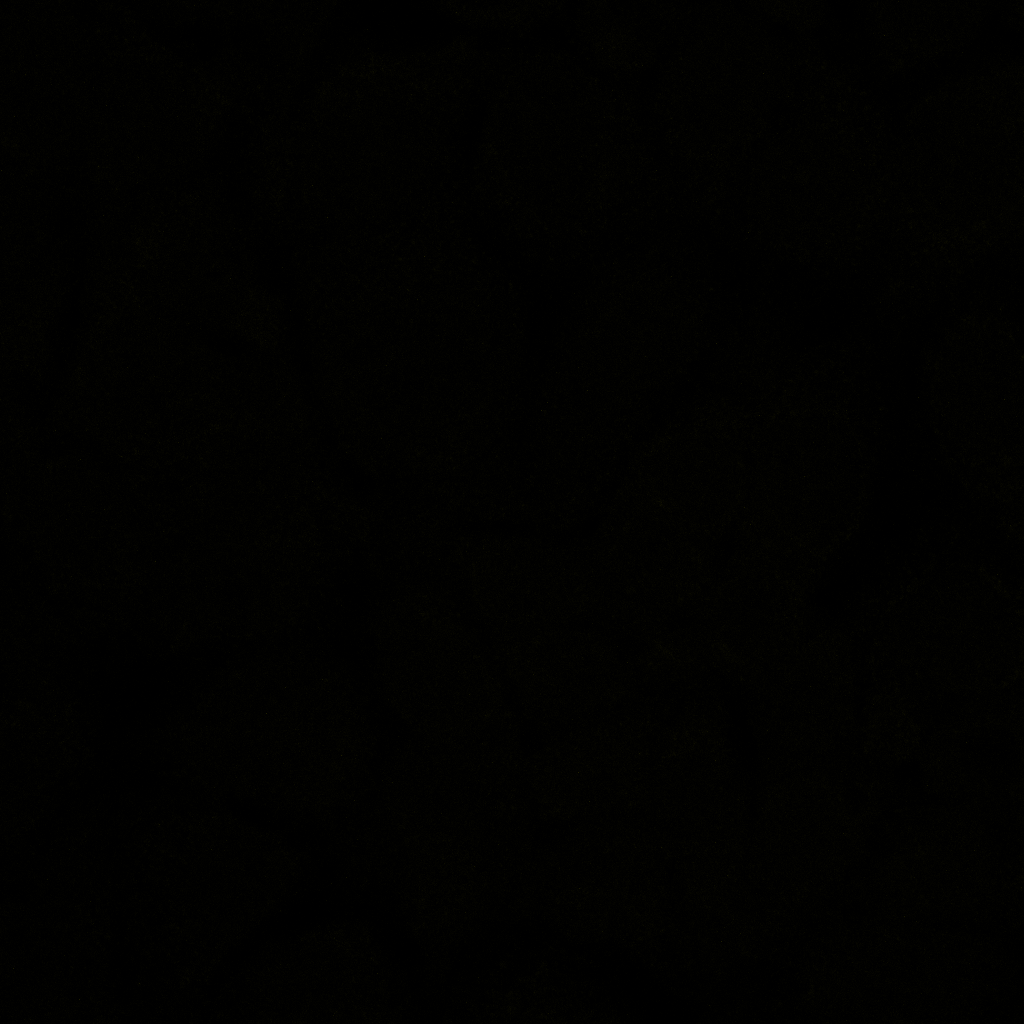

Supplement: Supplementary file 11 — Source Data Fig. 3 [file 44321_2024_32_MOESM11_ESM.zip › Figure 3/Figure 3C ZZW-115+Arsenate Flag GFP Dox.tif]

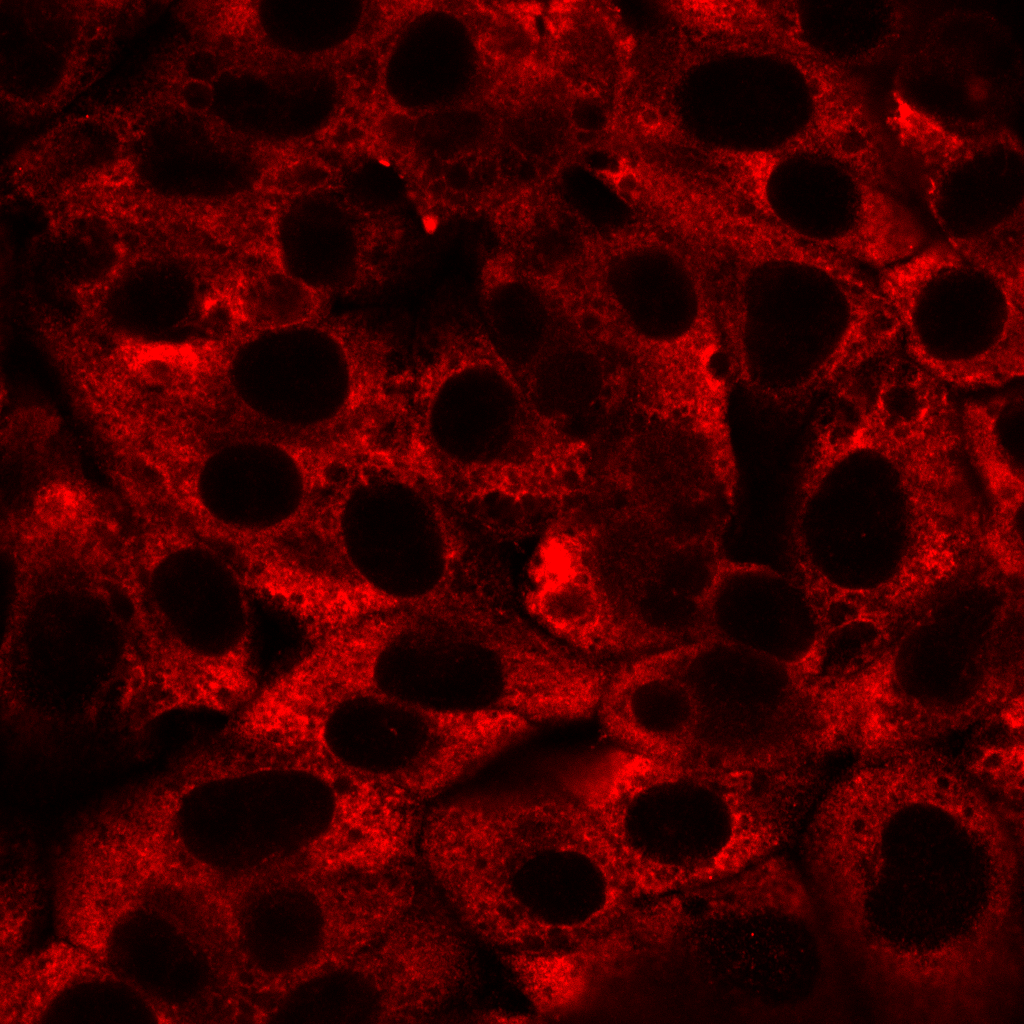

Supplement: Supplementary file 11 — Source Data Fig. 3 [file 44321_2024_32_MOESM11_ESM.zip › Figure 3/Figure 3C Untrated G3BP1 NUPR1mut-Flag Dox.tif]

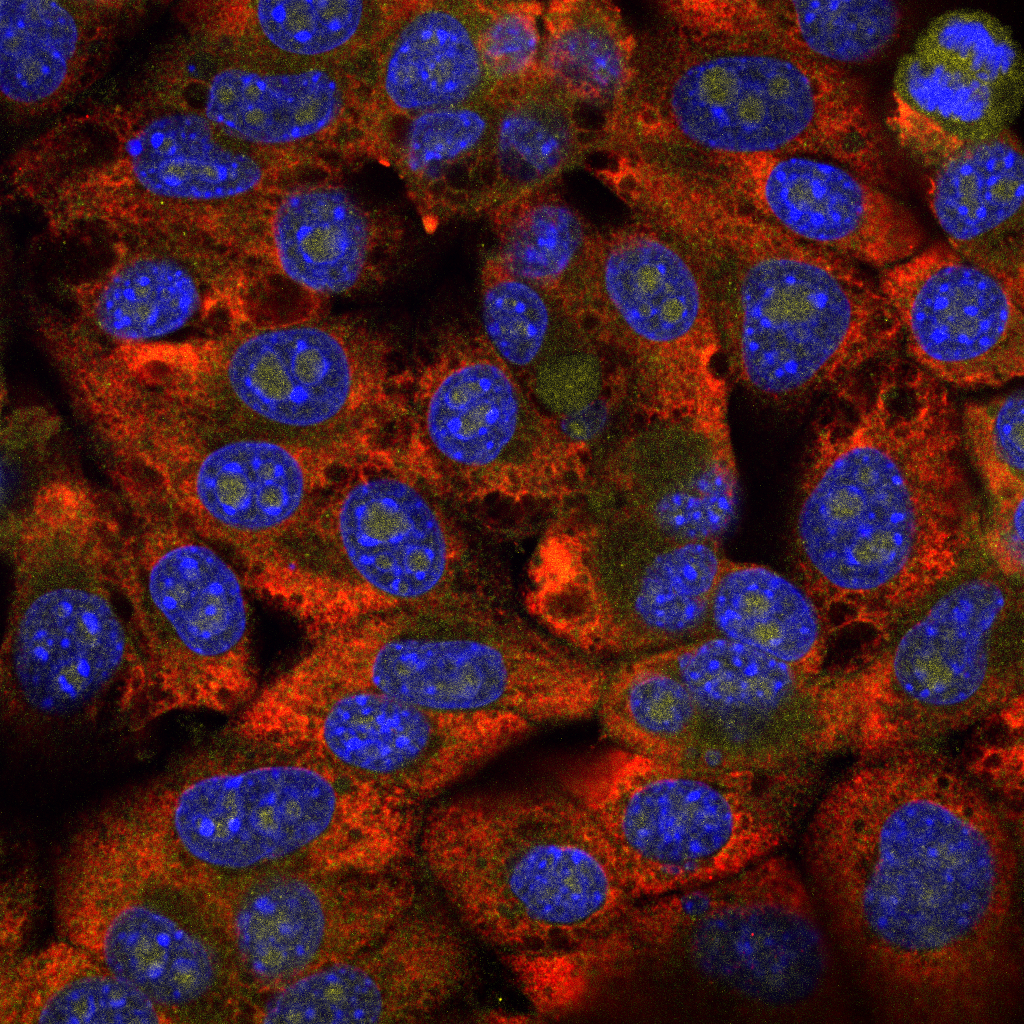

Supplement: Supplementary file 11 — Source Data Fig. 3 [file 44321_2024_32_MOESM11_ESM.zip › Figure 3/Figure 3C Untrated Merge NUPR1mut-Flag Dox.tif]

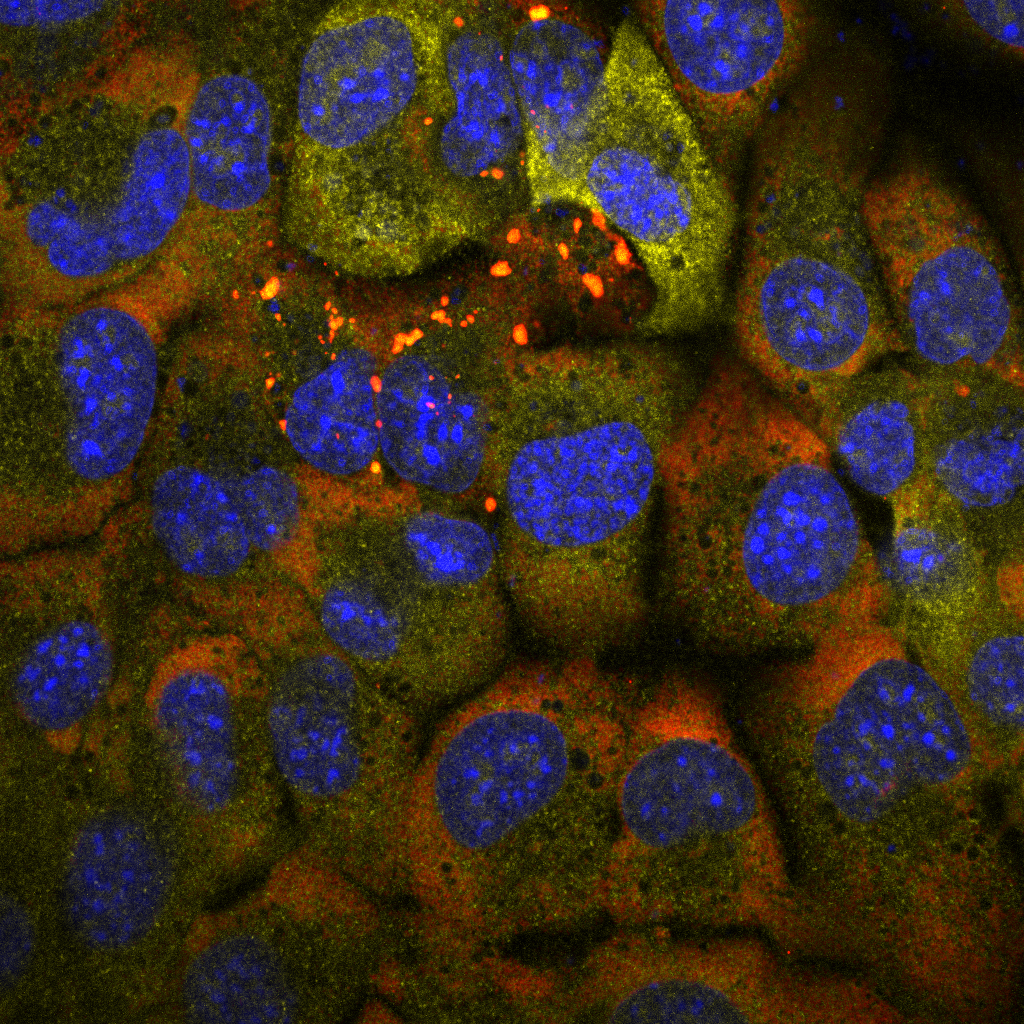

Supplement: Supplementary file 11 — Source Data Fig. 3 [file 44321_2024_32_MOESM11_ESM.zip › Figure 3/Figure 3C ZZW-115+Arsenate Merge NUPR1-Flag Vehicle.tif]

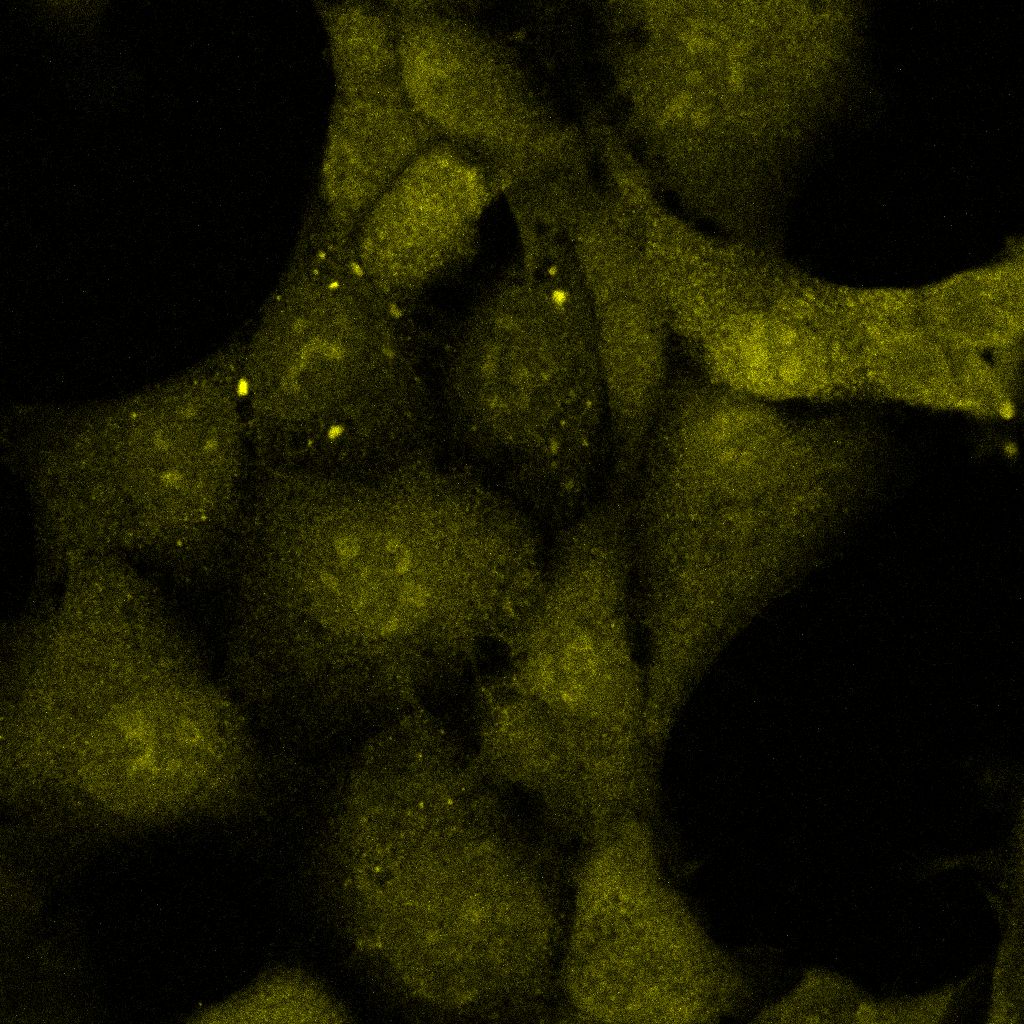

Supplement: Supplementary file 11 — Source Data Fig. 3 [file 44321_2024_32_MOESM11_ESM.zip › Figure 3/Figure 3C Untrated Flag NUPR1-Flag Dox.tif]

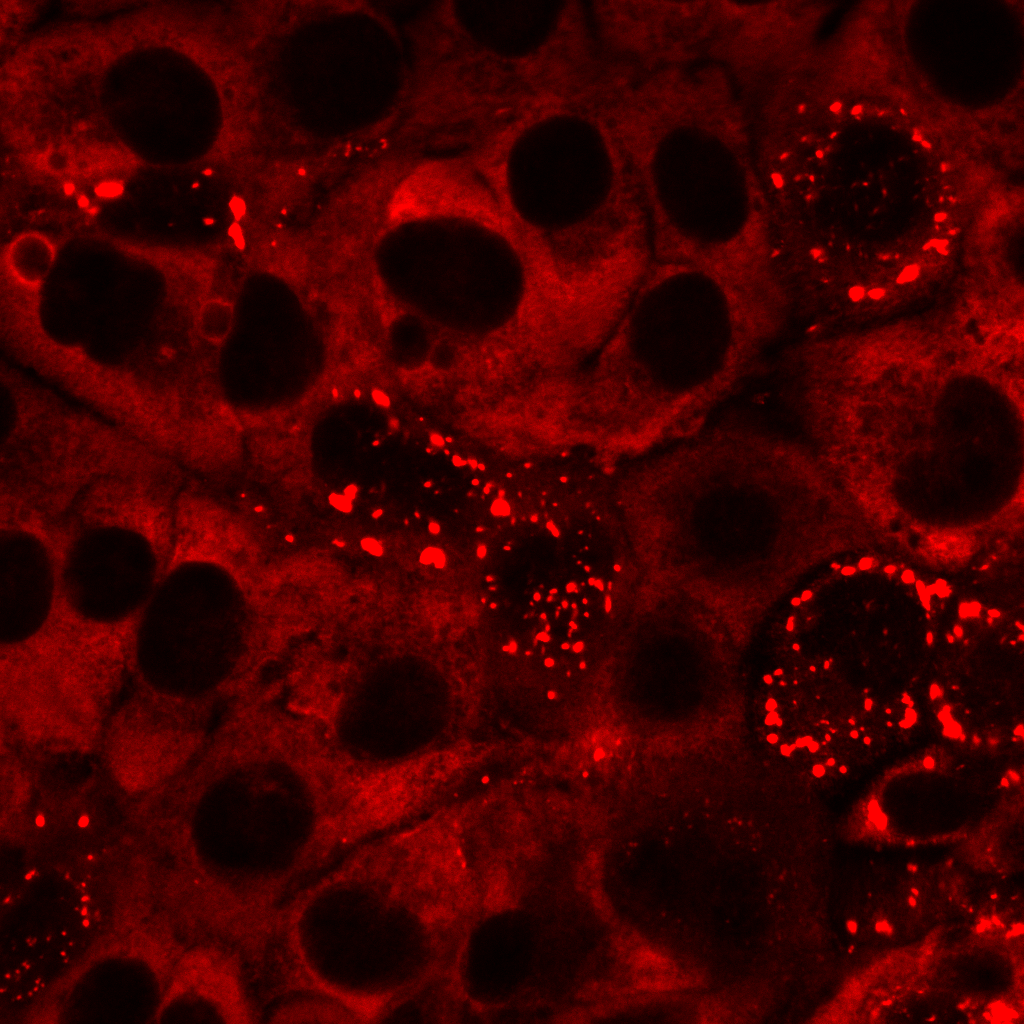

Supplement: Supplementary file 11 — Source Data Fig. 3 [file 44321_2024_32_MOESM11_ESM.zip › Figure 3/Figure 3C Untrated G3BP1 NUPR1-Flag Vehicle.tif]

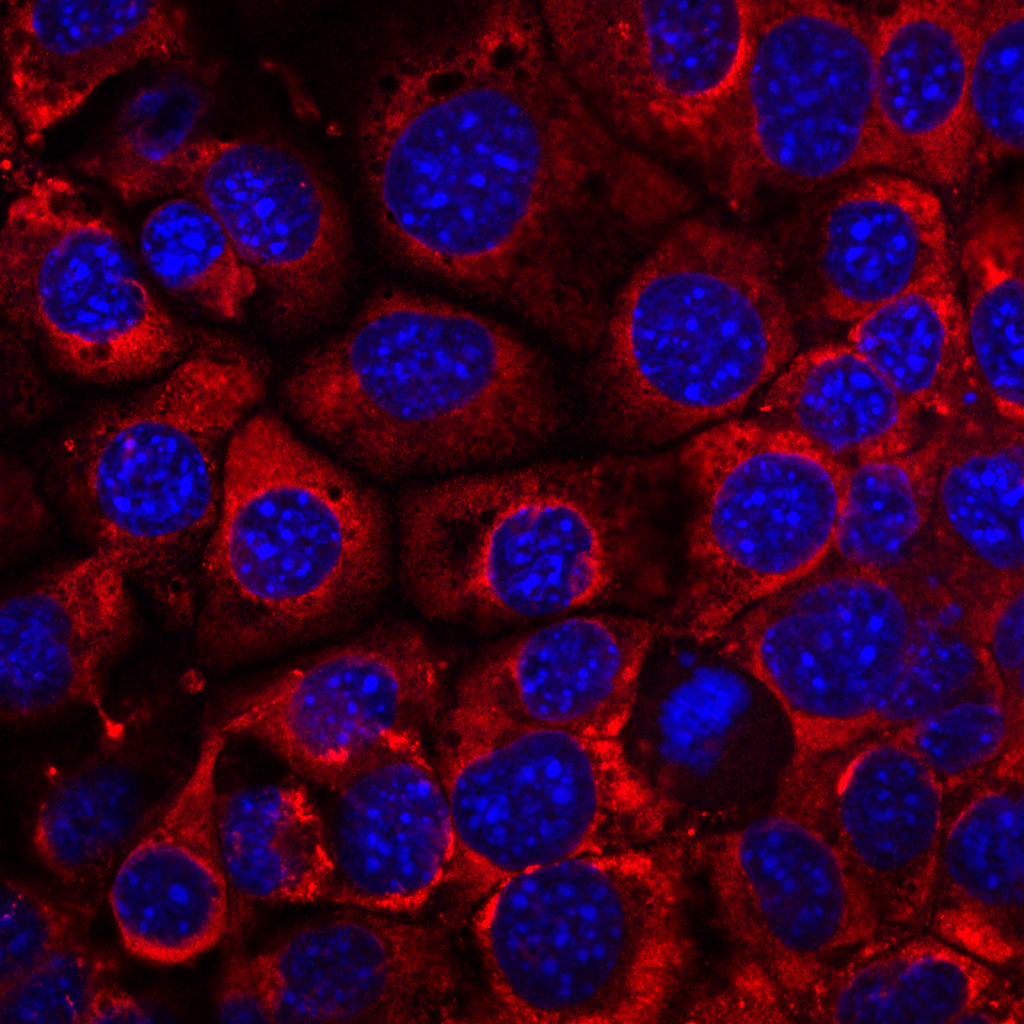

Supplement: Supplementary file 11 — Source Data Fig. 3 [file 44321_2024_32_MOESM11_ESM.zip › Figure 3/Figure 3C ZZW-115+Arsenate Merge GFP Vehicle.tif]

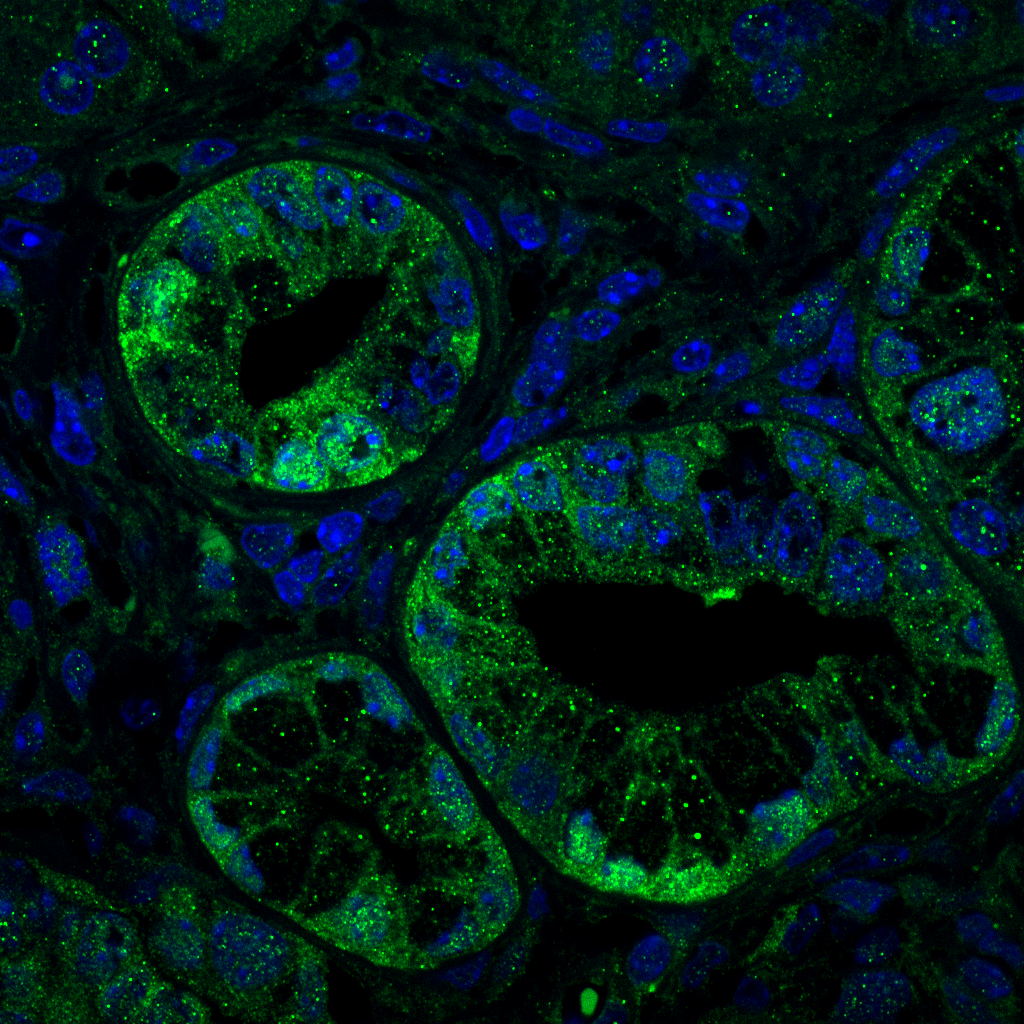

Supplement: Supplementary file 13 — Source Data Fig. 5 [file 44321_2024_32_MOESM13_ESM.zip › Figure 5/Figure 5C Vehicel KC.tif]

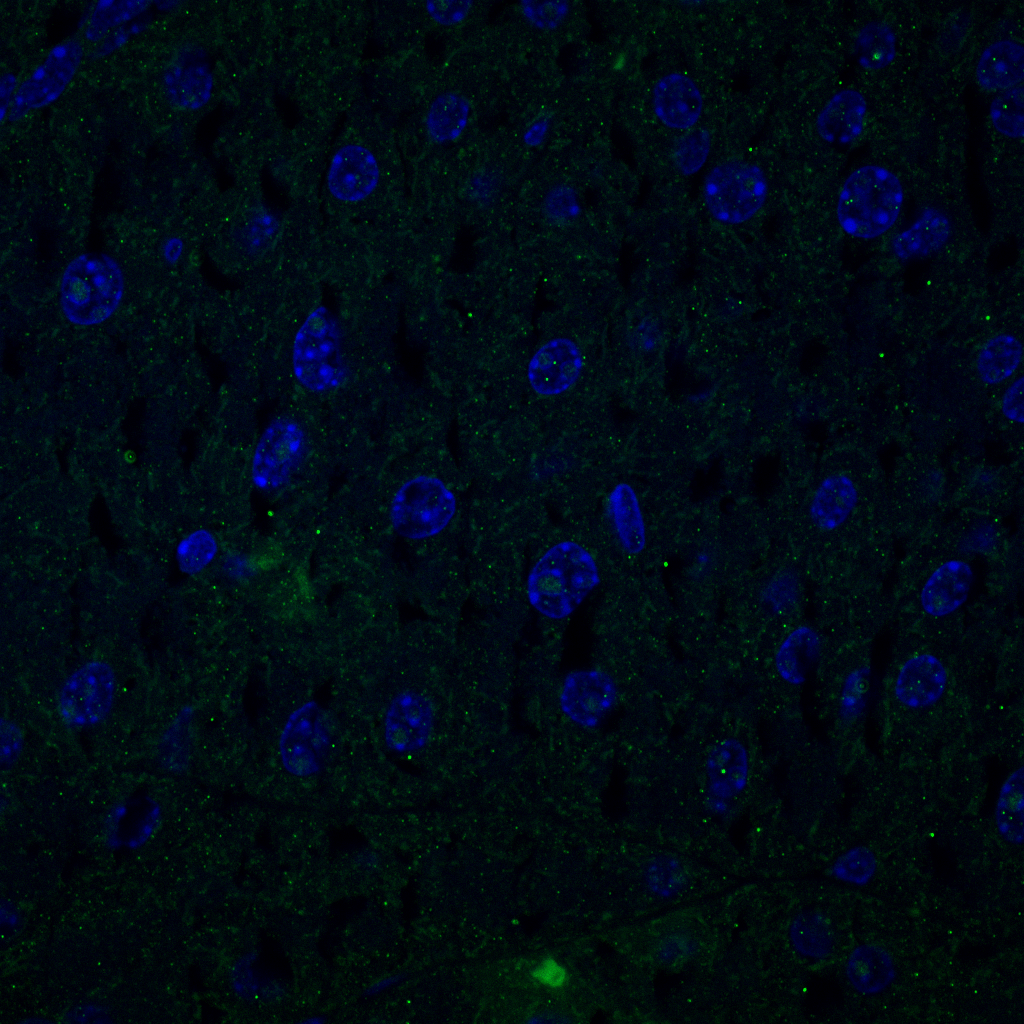

Supplement: Supplementary file 13 — Source Data Fig. 5 [file 44321_2024_32_MOESM13_ESM.zip › Figure 5/Figure 5F ZZW-115 control.tif]

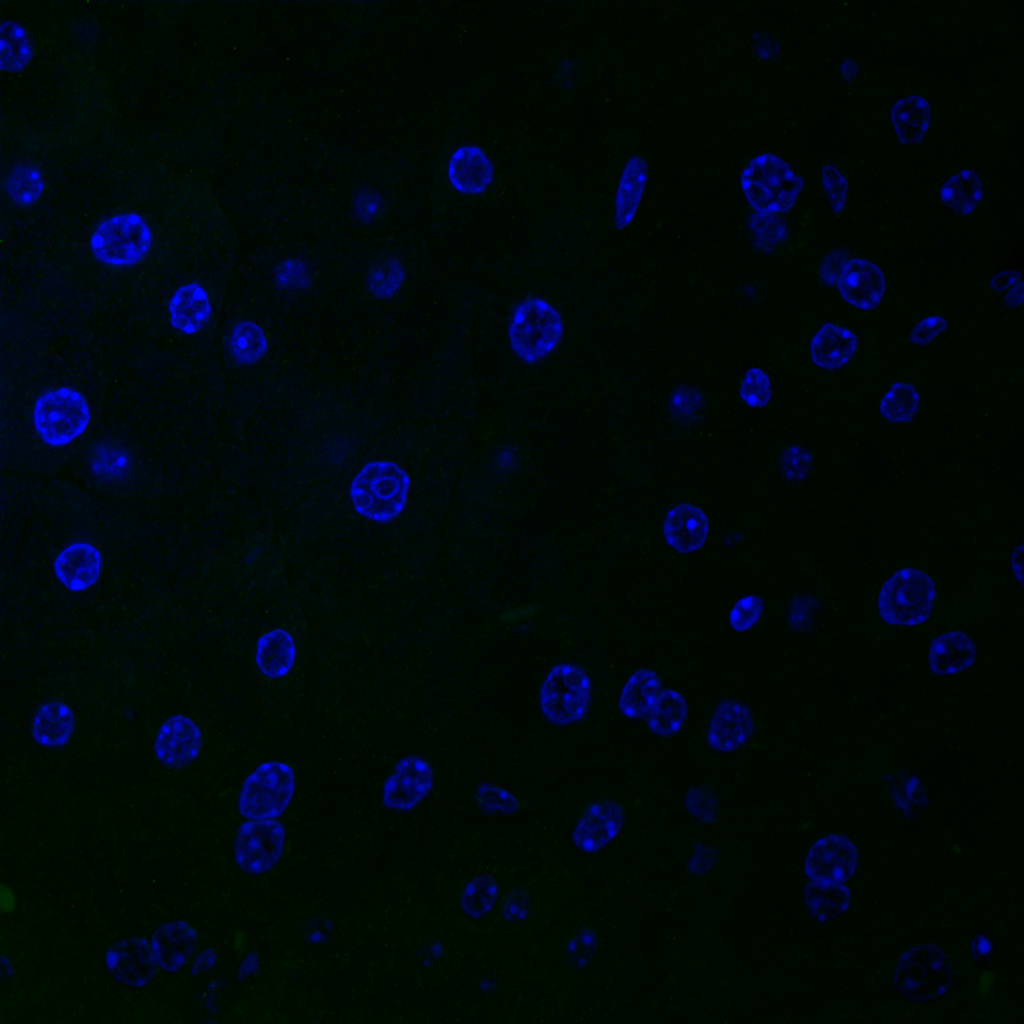

Supplement: Supplementary file 13 — Source Data Fig. 5 [file 44321_2024_32_MOESM13_ESM.zip › Figure 5/Figure 5E ZZW-115 Control.tif]

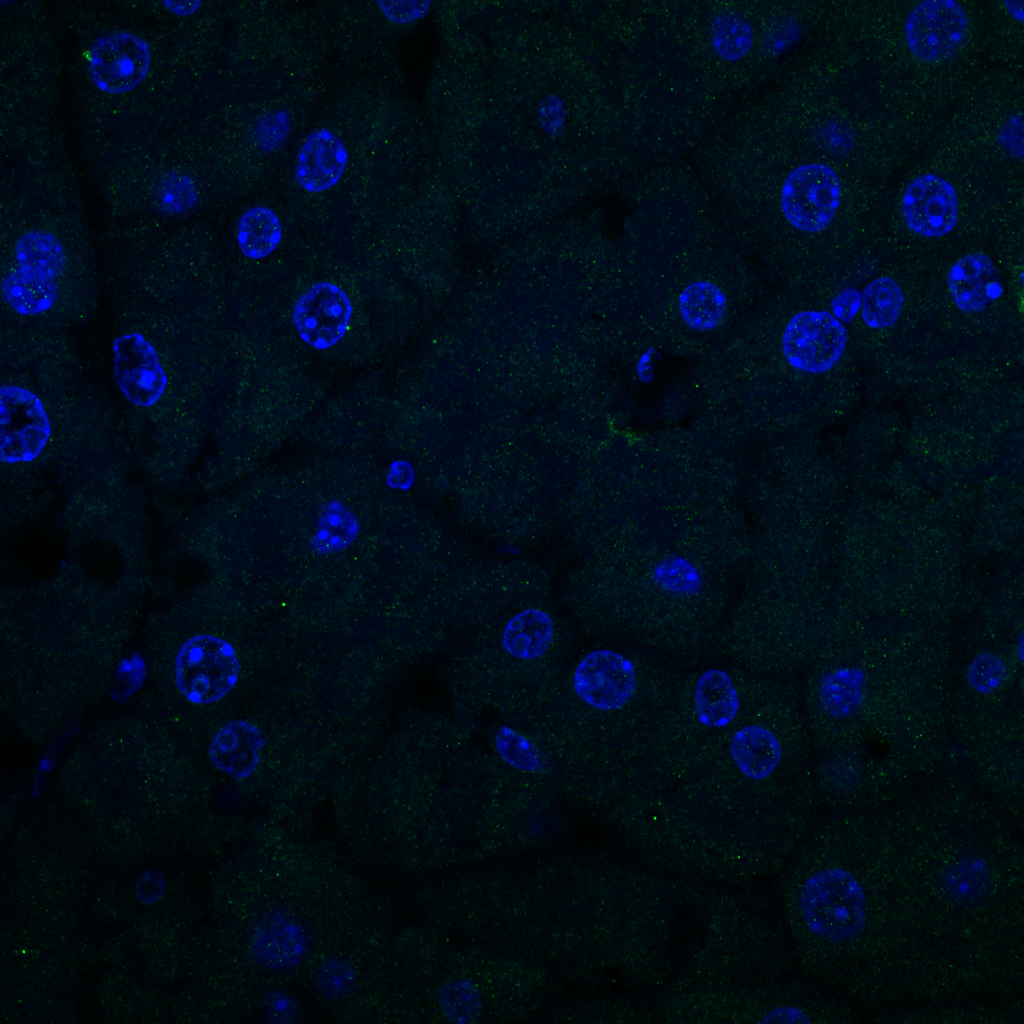

Supplement: Supplementary file 13 — Source Data Fig. 5 [file 44321_2024_32_MOESM13_ESM.zip › Figure 5/Figure 5D ZZW-115 Control.tif]

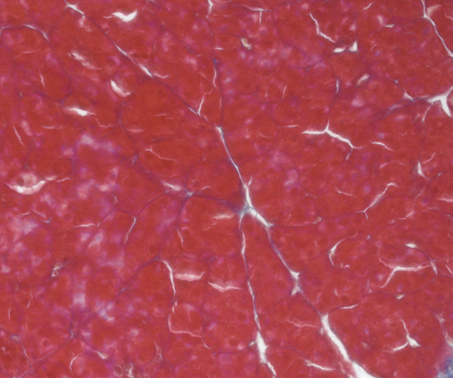

Supplement: Supplementary file 13 — Source Data Fig. 5 [file 44321_2024_32_MOESM13_ESM.zip › Figure 5/Figure 5B ZZW-115 control.tif]

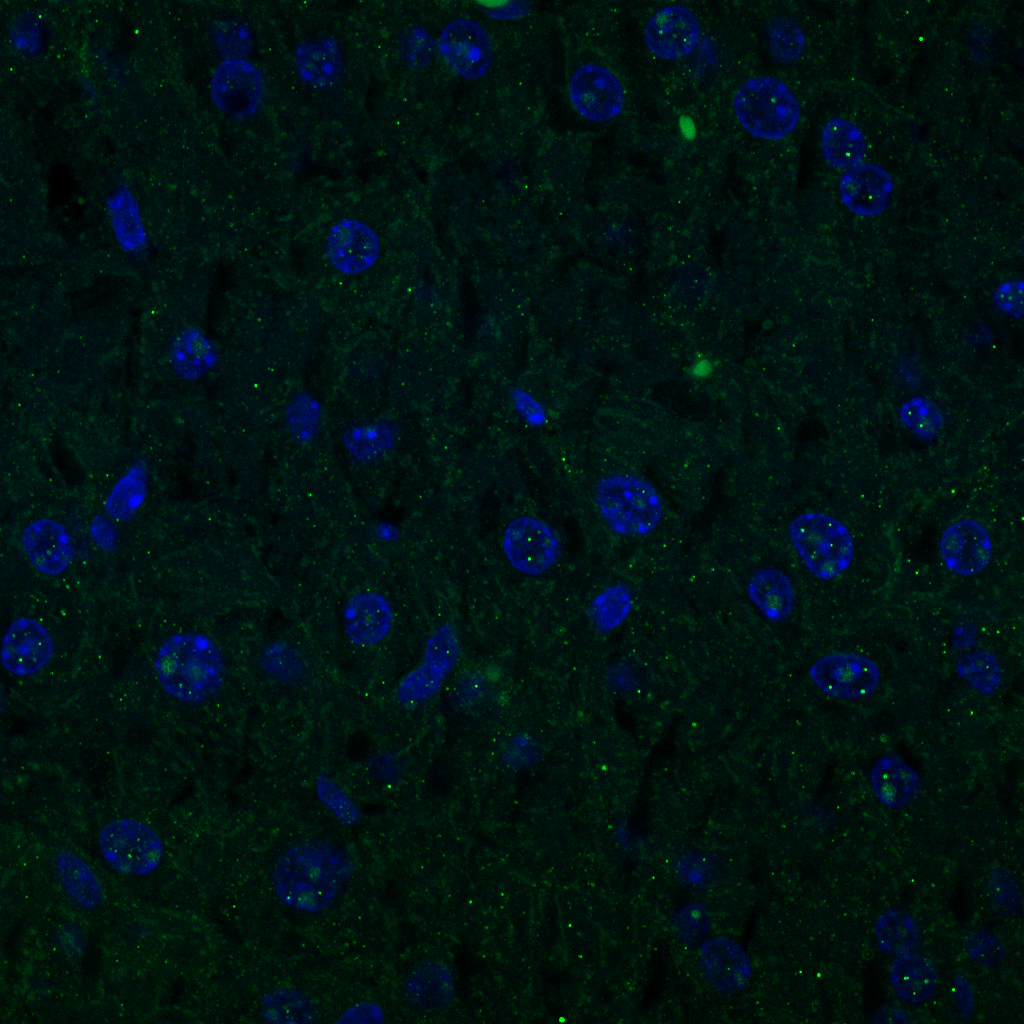

Supplement: Supplementary file 13 — Source Data Fig. 5 [file 44321_2024_32_MOESM13_ESM.zip › Figure 5/Figure 5C ZZW-115 control.tif]

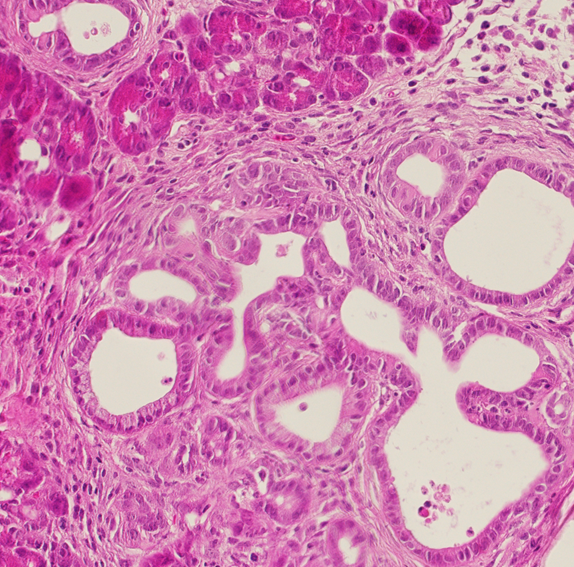

Supplement: Supplementary file 13 — Source Data Fig. 5 [file 44321_2024_32_MOESM13_ESM.zip › Figure 5/Figure 5A Vehicel KC.tif]

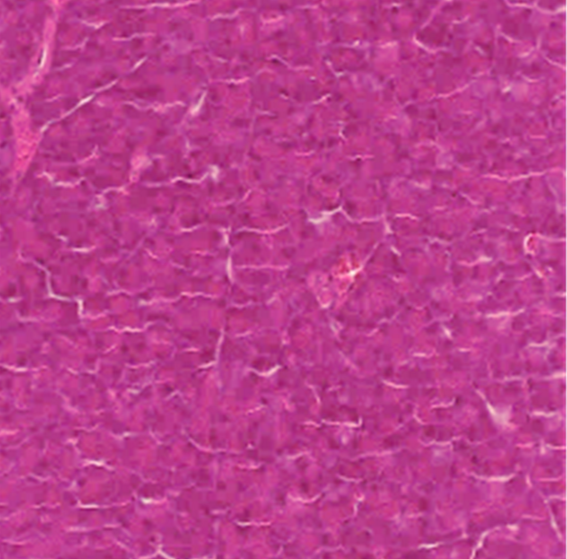

Supplement: Supplementary file 13 — Source Data Fig. 5 [file 44321_2024_32_MOESM13_ESM.zip › Figure 5/Figure 5A ZZW-115 control.tif]

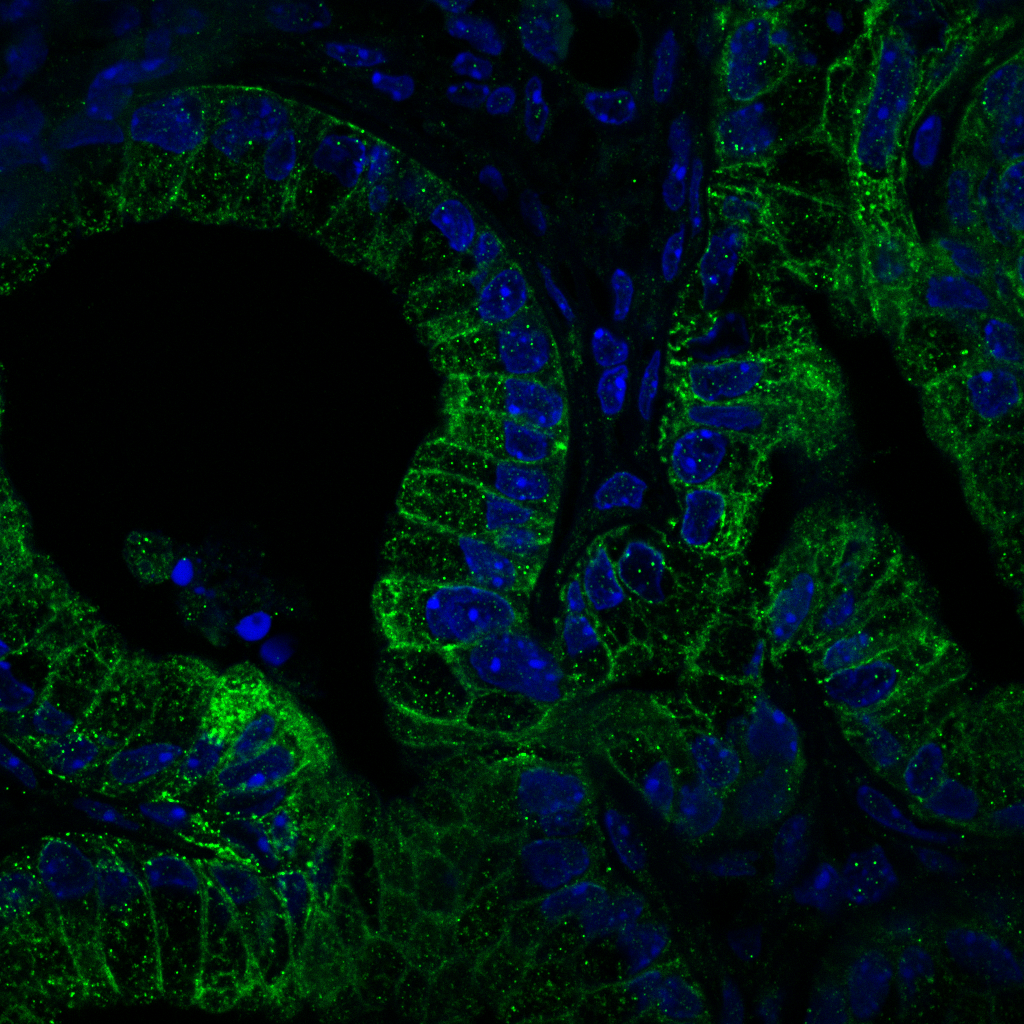

Supplement: Supplementary file 13 — Source Data Fig. 5 [file 44321_2024_32_MOESM13_ESM.zip › Figure 5/Figure 5F Vehicle KC.tif]

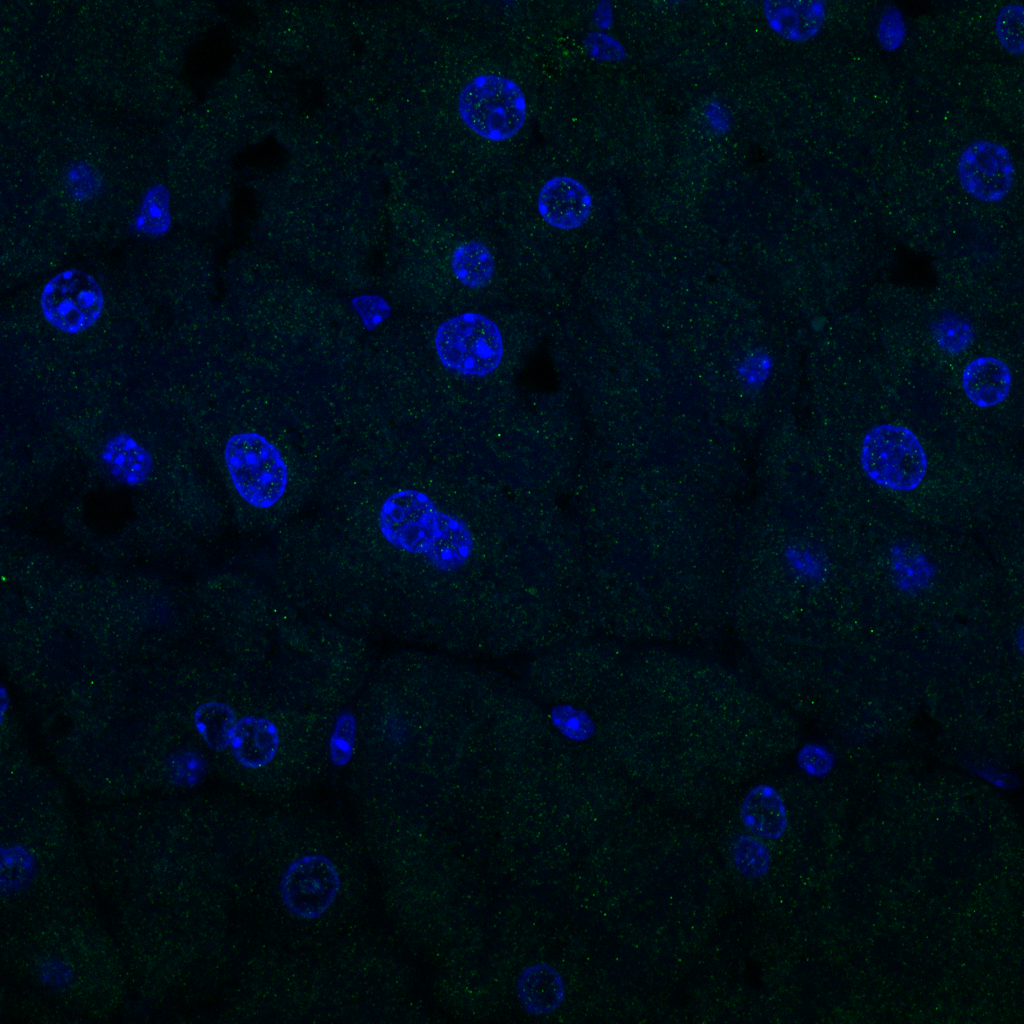

Supplement: Supplementary file 13 — Source Data Fig. 5 [file 44321_2024_32_MOESM13_ESM.zip › Figure 5/Figure 5D ZZW-115 KC.tif]

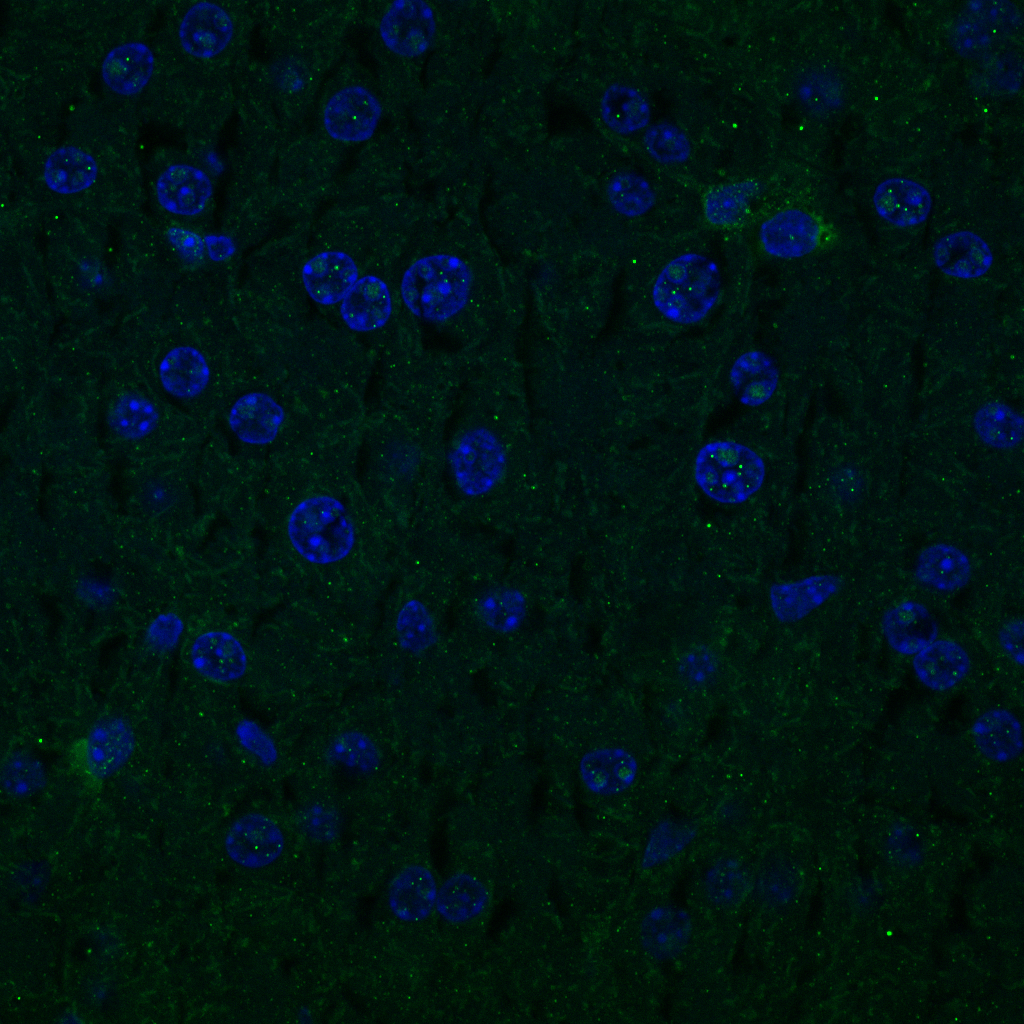

Supplement: Supplementary file 13 — Source Data Fig. 5 [file 44321_2024_32_MOESM13_ESM.zip › Figure 5/Figure 5C ZZW-115 KC.tif]

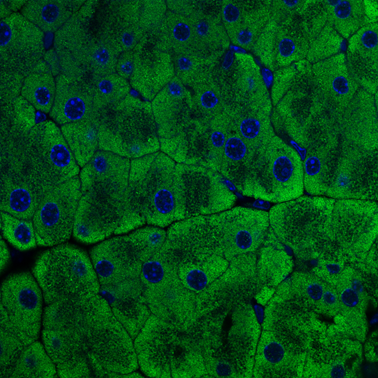

Supplement: Supplementary file 13 — Source Data Fig. 5 [file 44321_2024_32_MOESM13_ESM.zip › Figure 5/Figure 5G Control Vehicel.tif]

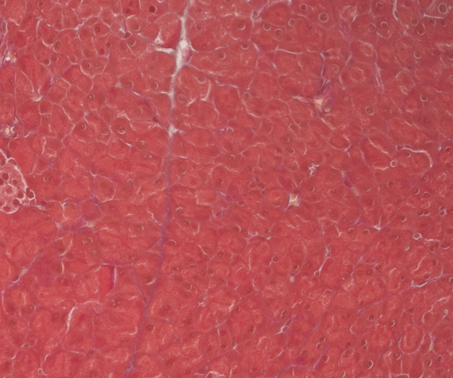

Supplement: Supplementary file 13 — Source Data Fig. 5 [file 44321_2024_32_MOESM13_ESM.zip › Figure 5/Figure 5B ZZW-115 KC.tif]

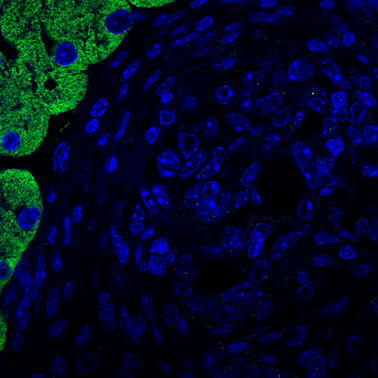

Supplement: Supplementary file 13 — Source Data Fig. 5 [file 44321_2024_32_MOESM13_ESM.zip › Figure 5/Figure 5G KC Vehicel.tif]

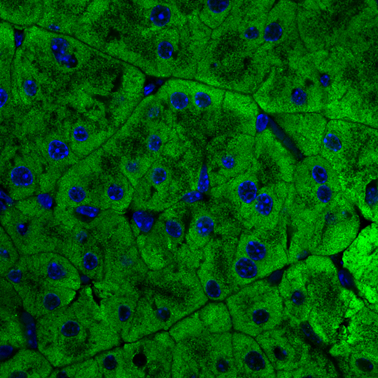

Supplement: Supplementary file 13 — Source Data Fig. 5 [file 44321_2024_32_MOESM13_ESM.zip › Figure 5/Figure 5G KC ZZW-115l.tif]

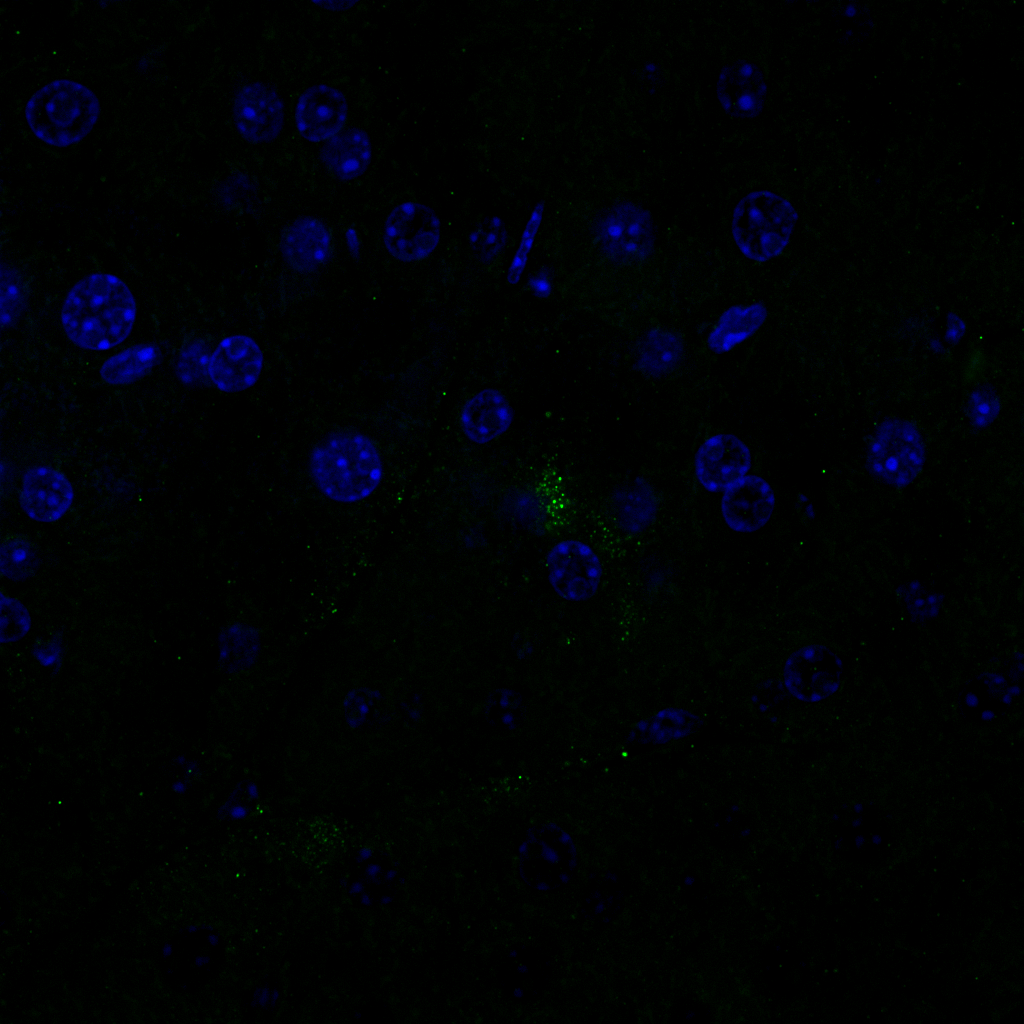

Supplement: Supplementary file 13 — Source Data Fig. 5 [file 44321_2024_32_MOESM13_ESM.zip › Figure 5/Figure 5E ZZW-115 KC.tif]

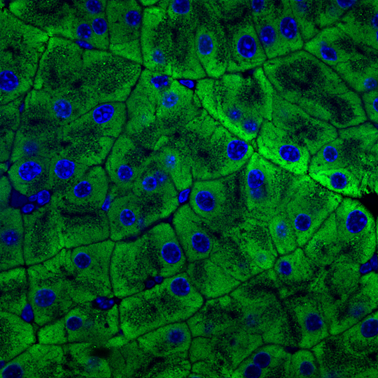

Supplement: Supplementary file 13 — Source Data Fig. 5 [file 44321_2024_32_MOESM13_ESM.zip › Figure 5/Figure 5G Control ZZW-115l.tif]

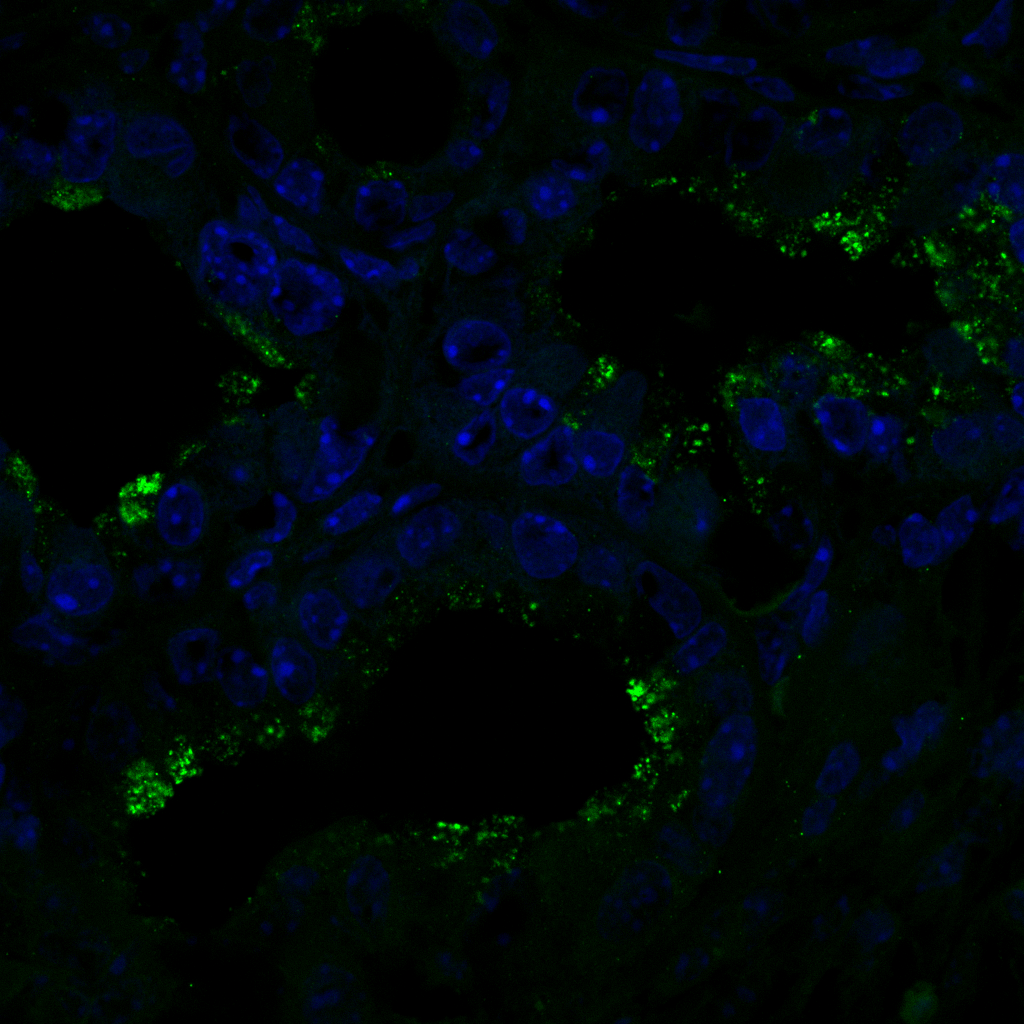

Supplement: Supplementary file 13 — Source Data Fig. 5 [file 44321_2024_32_MOESM13_ESM.zip › Figure 5/Figure 5E Vehicle KC.tif]

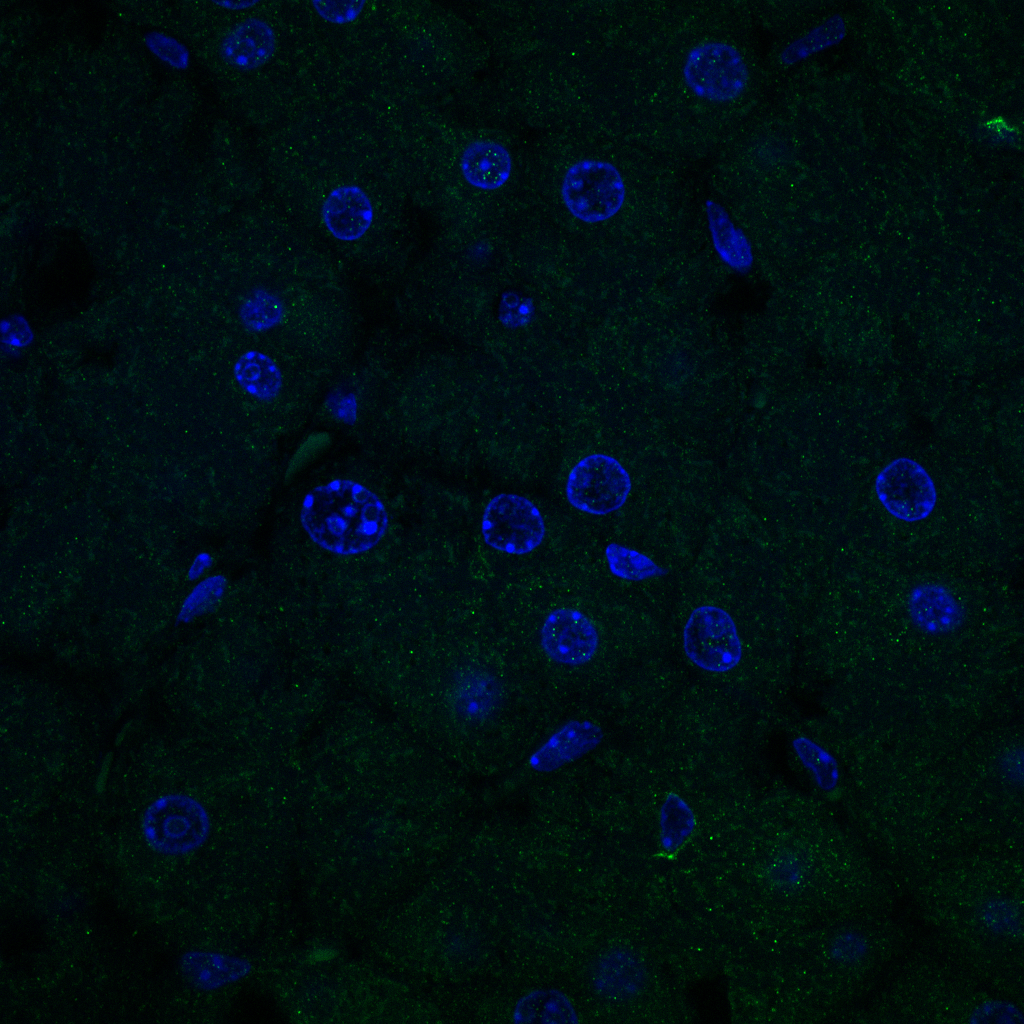

Supplement: Supplementary file 13 — Source Data Fig. 5 [file 44321_2024_32_MOESM13_ESM.zip › Figure 5/Figure 5F Vehicle control.tif]

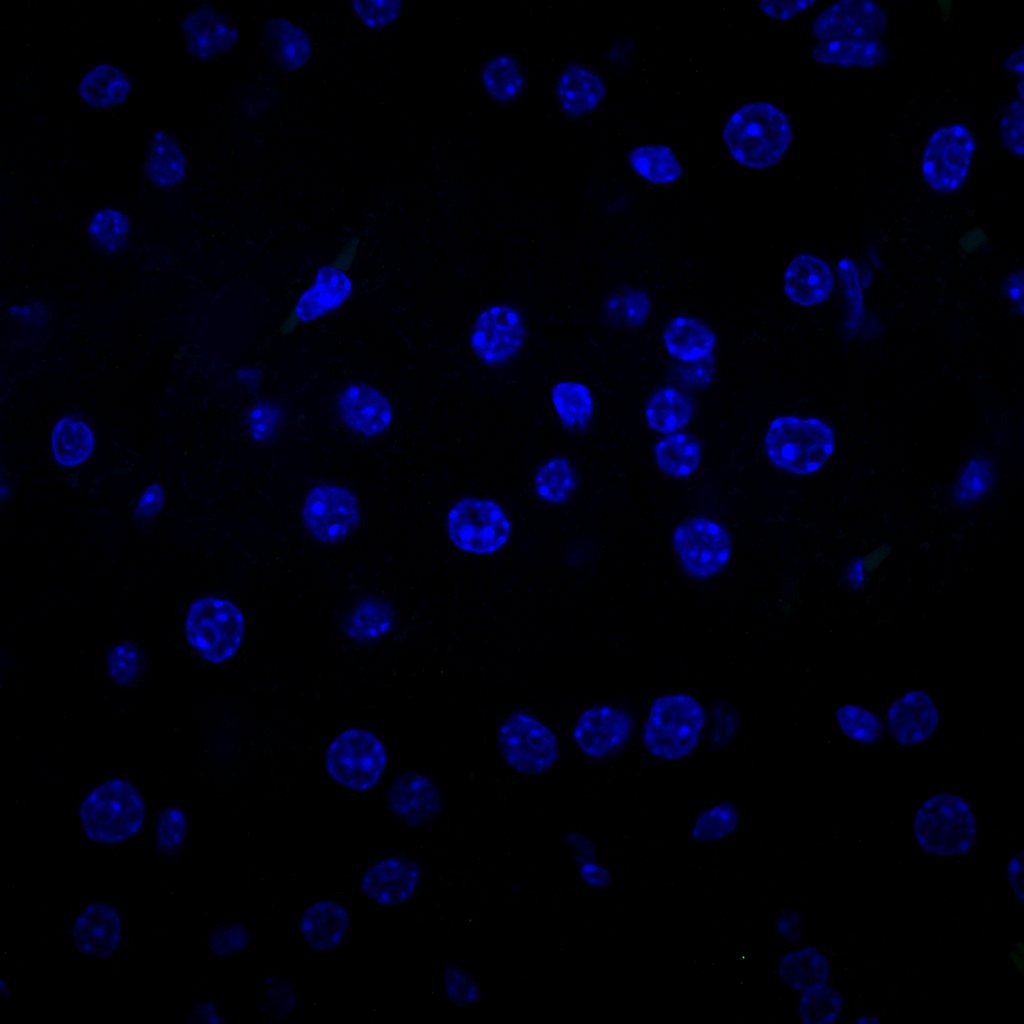

Supplement: Supplementary file 13 — Source Data Fig. 5 [file 44321_2024_32_MOESM13_ESM.zip › Figure 5/Figure 5E Vehicle Control.tif]

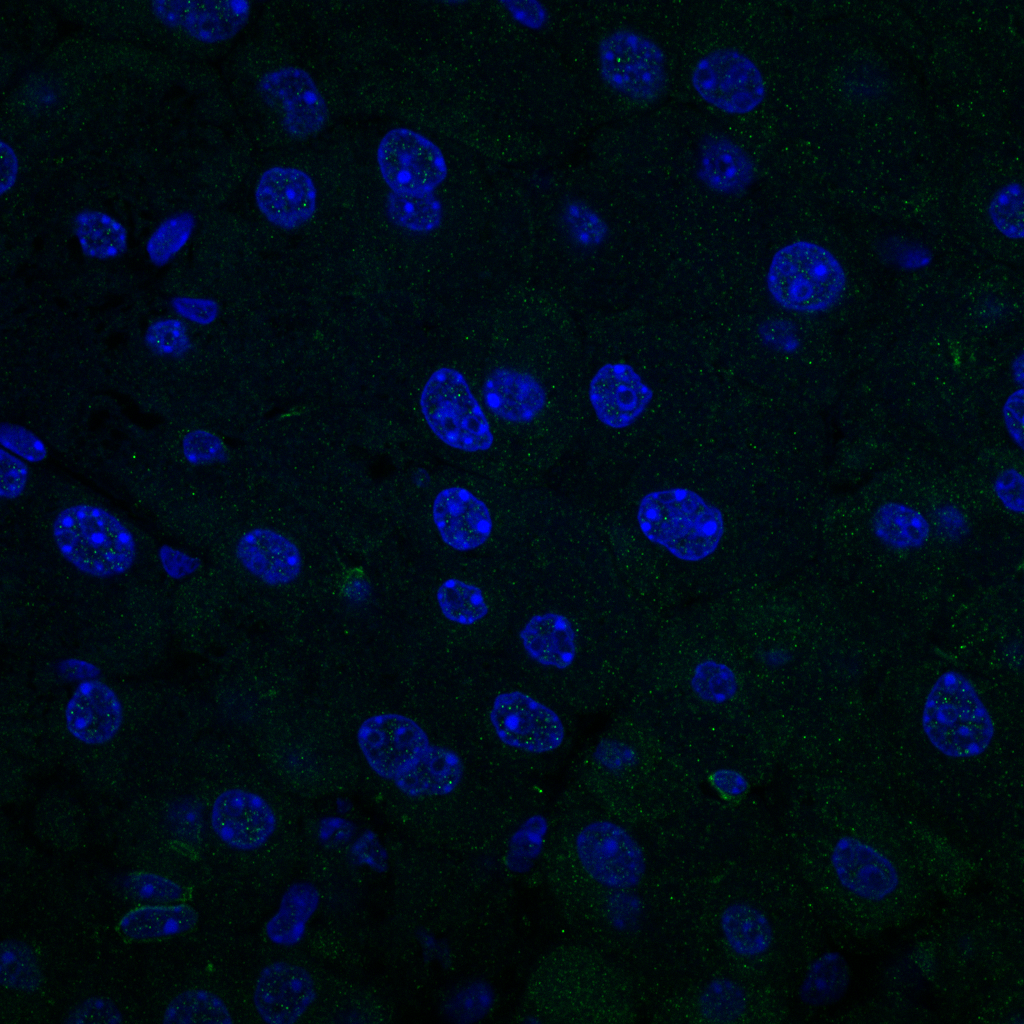

Supplement: Supplementary file 13 — Source Data Fig. 5 [file 44321_2024_32_MOESM13_ESM.zip › Figure 5/Figure 5D Vehicle control.tif]

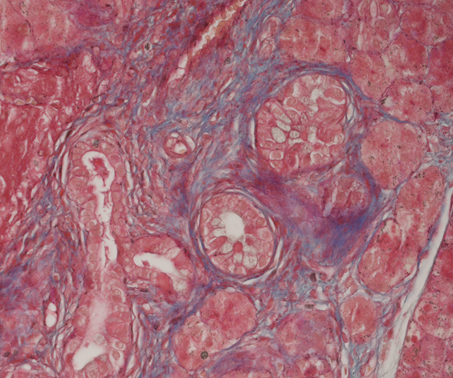

Supplement: Supplementary file 13 — Source Data Fig. 5 [file 44321_2024_32_MOESM13_ESM.zip › Figure 5/Figure 5B Vehicle KC.tif]

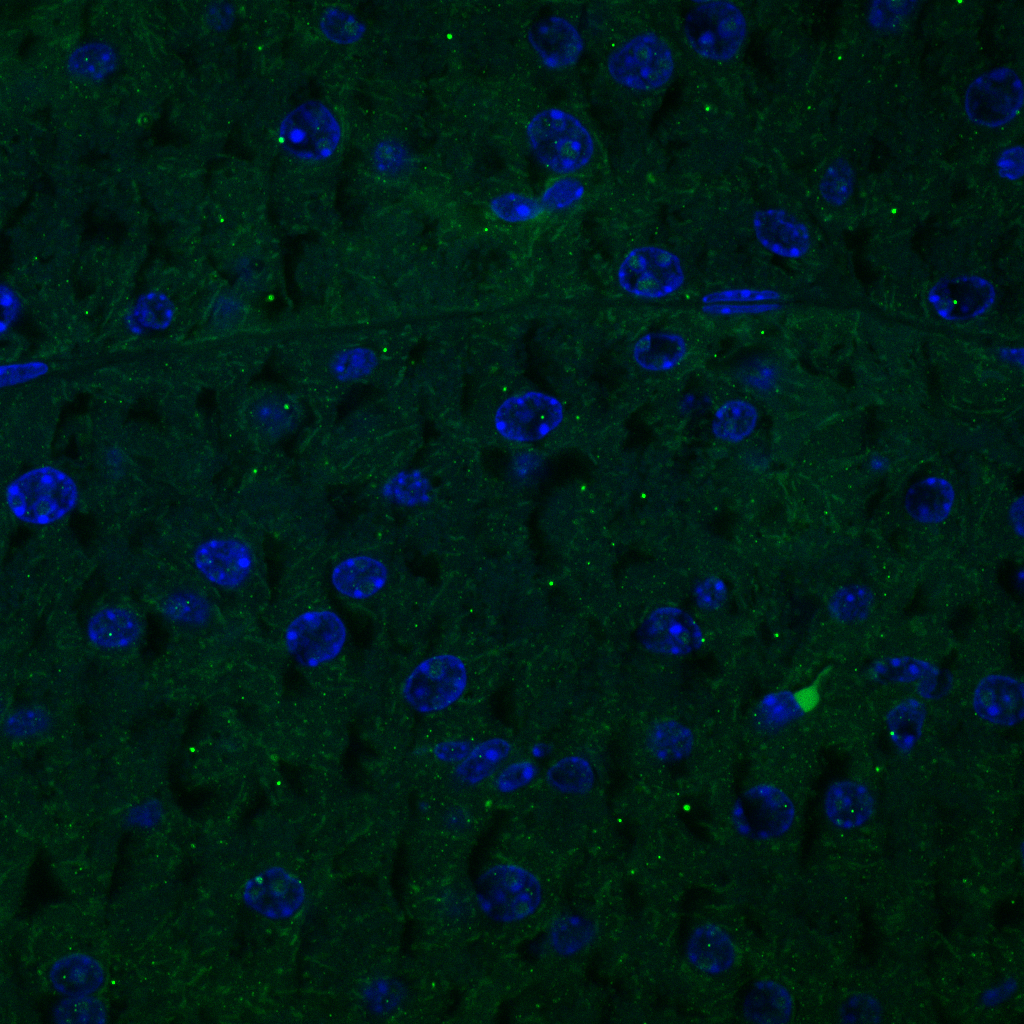

Supplement: Supplementary file 13 — Source Data Fig. 5 [file 44321_2024_32_MOESM13_ESM.zip › Figure 5/Figure 5C Vehicle control.tif]

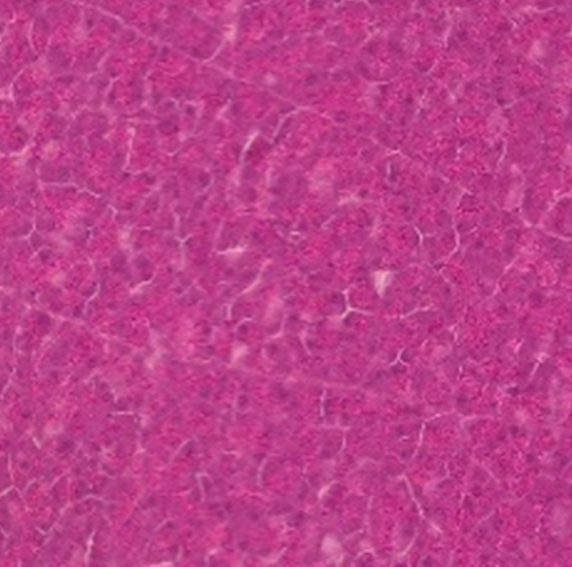

Supplement: Supplementary file 13 — Source Data Fig. 5 [file 44321_2024_32_MOESM13_ESM.zip › Figure 5/Figure 5A ZZW-115 KC.tif]

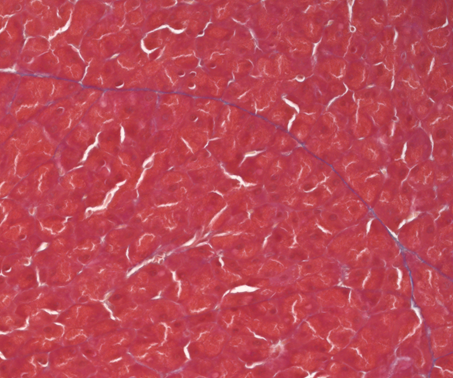

Supplement: Supplementary file 13 — Source Data Fig. 5 [file 44321_2024_32_MOESM13_ESM.zip › Figure 5/Figure 5B Vehicle control.tif]

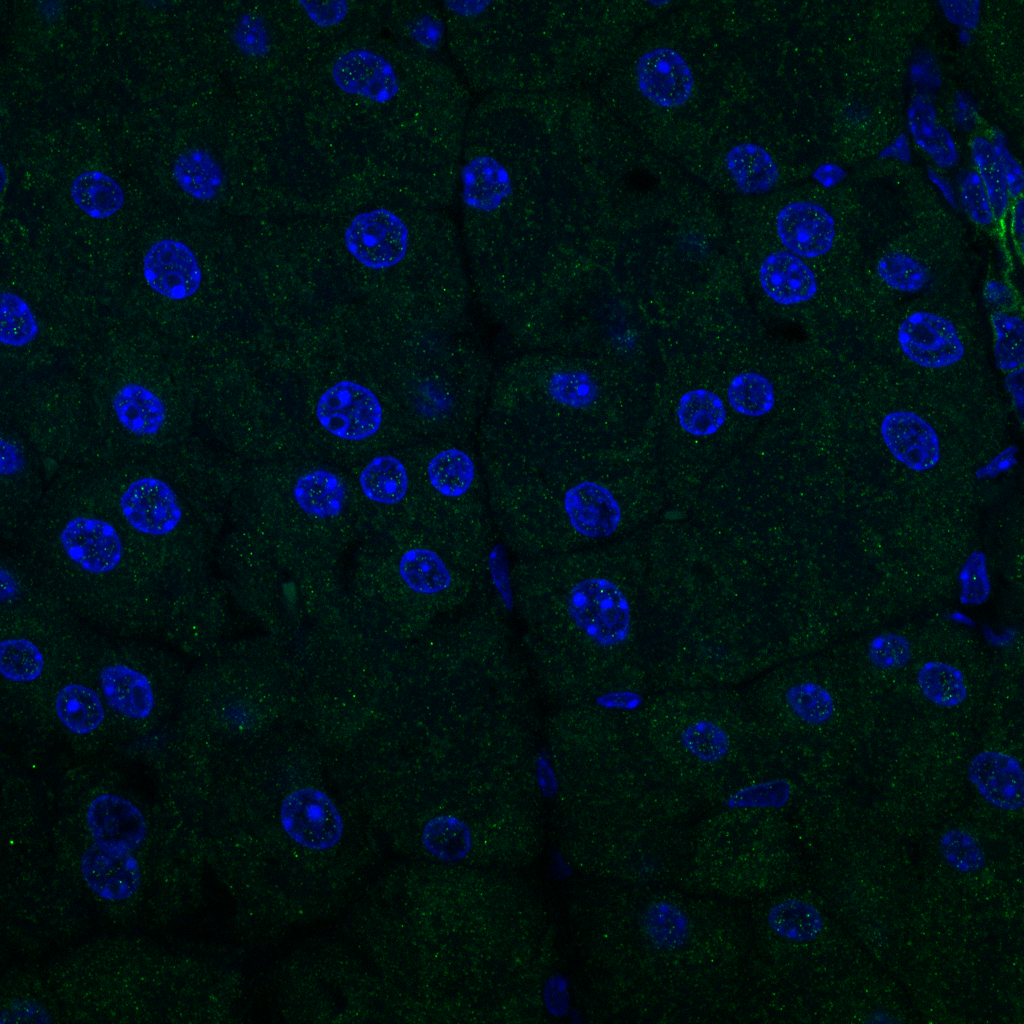

Supplement: Supplementary file 13 — Source Data Fig. 5 [file 44321_2024_32_MOESM13_ESM.zip › Figure 5/Figure 5F ZZW-115 KC.tif]

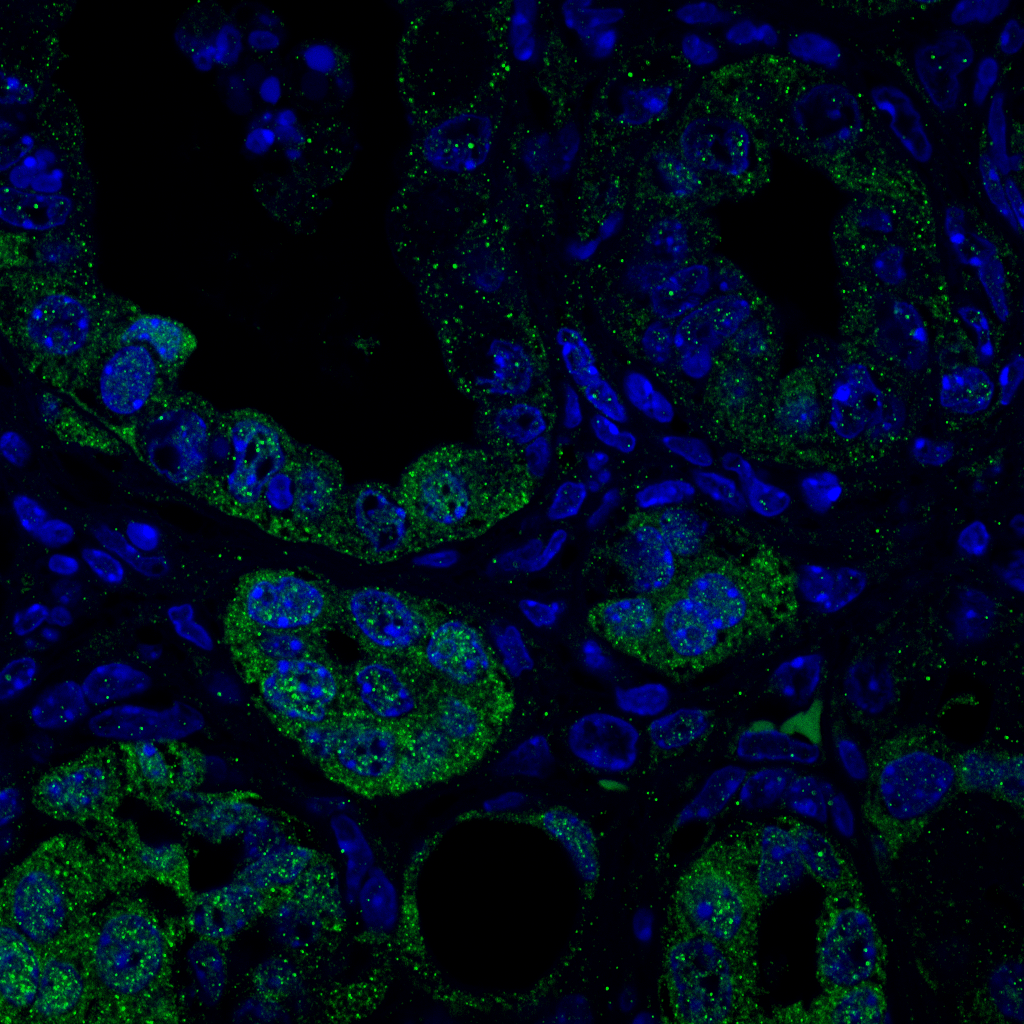

Supplement: Supplementary file 13 — Source Data Fig. 5 [file 44321_2024_32_MOESM13_ESM.zip › Figure 5/Figure 5D Vehicle KC.tif]

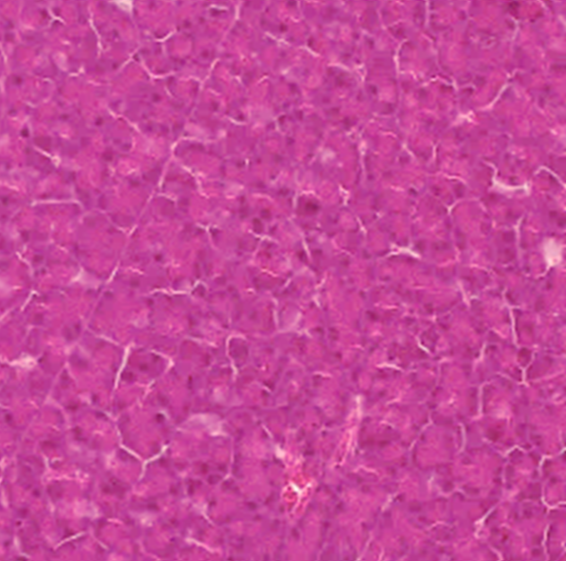

Supplement: Supplementary file 13 — Source Data Fig. 5 [file 44321_2024_32_MOESM13_ESM.zip › Figure 5/Figure 5A Vehicle control.tif]

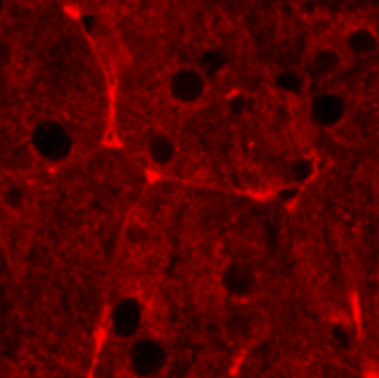

Supplement: Supplementary file 14 — Source Data Fig. 6 [file 44321_2024_32_MOESM14_ESM.zip › Figure 6/Figure 6A G3BP1 Control Vehicle.tif]

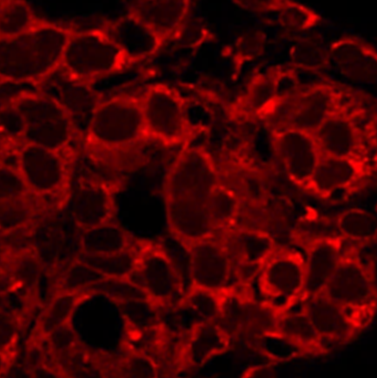

Supplement: Supplementary file 14 — Source Data Fig. 6 [file 44321_2024_32_MOESM14_ESM.zip › Figure 6/Figure 6A G3BP1 KC Vehicle.tif]

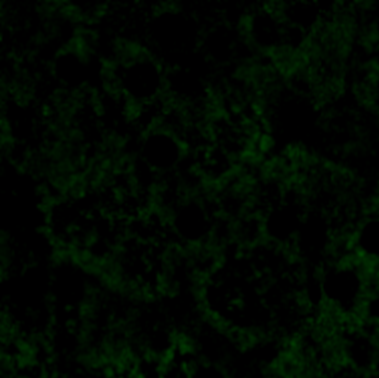

Supplement: Supplementary file 14 — Source Data Fig. 6 [file 44321_2024_32_MOESM14_ESM.zip › Figure 6/Figure 6A NUPR1 KC ZZW-115.tif]

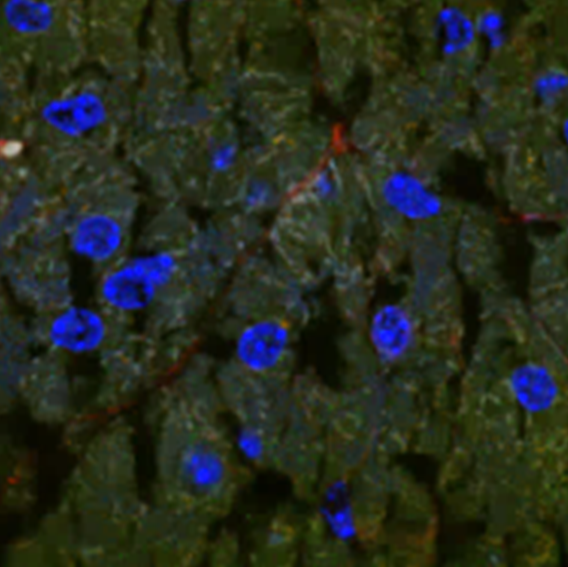

Supplement: Supplementary file 14 — Source Data Fig. 6 [file 44321_2024_32_MOESM14_ESM.zip › Figure 6/Figure 6B Merge KC ZZW-115.tif]

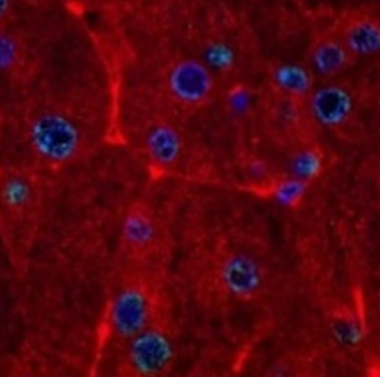

Supplement: Supplementary file 14 — Source Data Fig. 6 [file 44321_2024_32_MOESM14_ESM.zip › Figure 6/Figure 6A Merge Control Vehicle.tif]

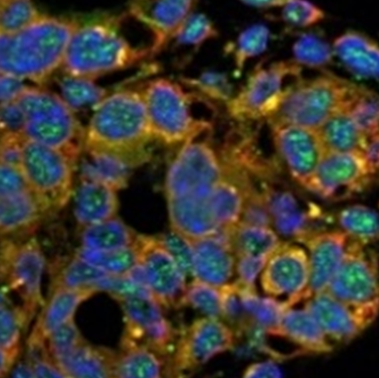

Supplement: Supplementary file 14 — Source Data Fig. 6 [file 44321_2024_32_MOESM14_ESM.zip › Figure 6/Figure 6A Merge KC Vehicle.tif]

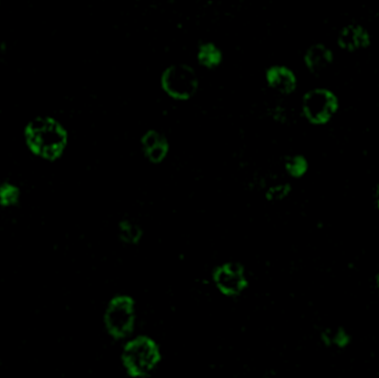

Supplement: Supplementary file 14 — Source Data Fig. 6 [file 44321_2024_32_MOESM14_ESM.zip › Figure 6/Figure 6A NUPR1 Control Vehicle.tif]

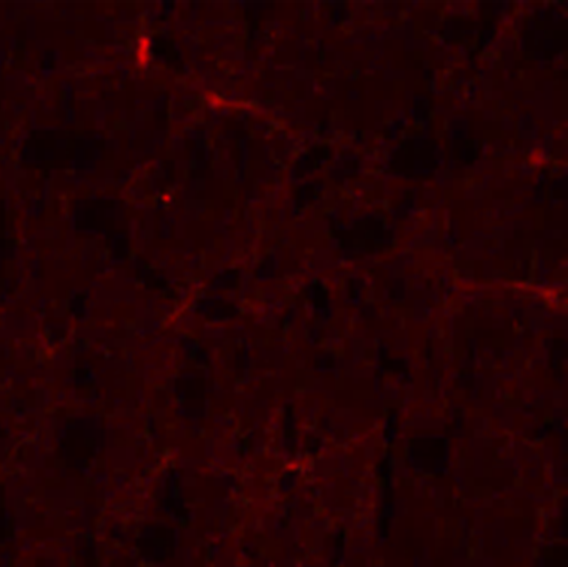

Supplement: Supplementary file 14 — Source Data Fig. 6 [file 44321_2024_32_MOESM14_ESM.zip › Figure 6/Figure 6B PABp Control ZZW-115.tif]

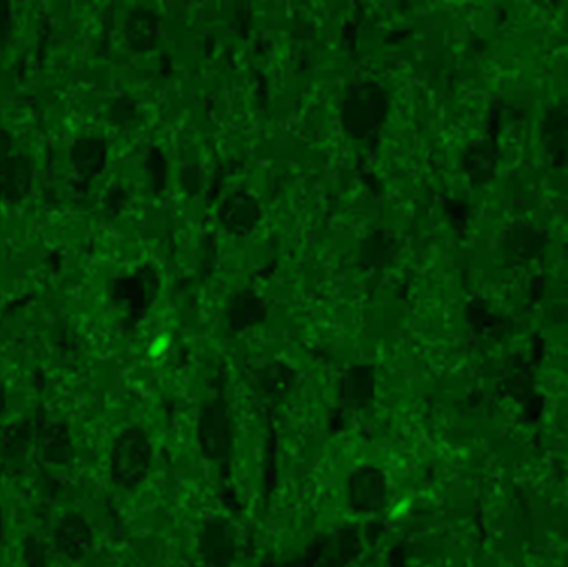

Supplement: Supplementary file 14 — Source Data Fig. 6 [file 44321_2024_32_MOESM14_ESM.zip › Figure 6/Figure 6B pEIF2 Control Vehicle.tif]

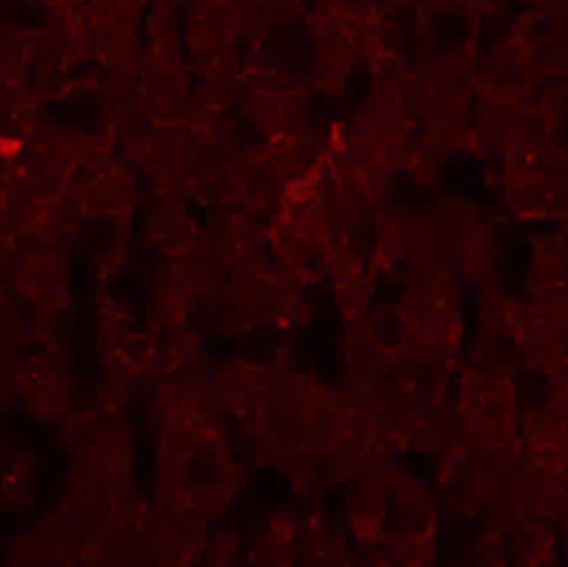

Supplement: Supplementary file 14 — Source Data Fig. 6 [file 44321_2024_32_MOESM14_ESM.zip › Figure 6/Figure 6B PABP KC ZZW-115.tif]

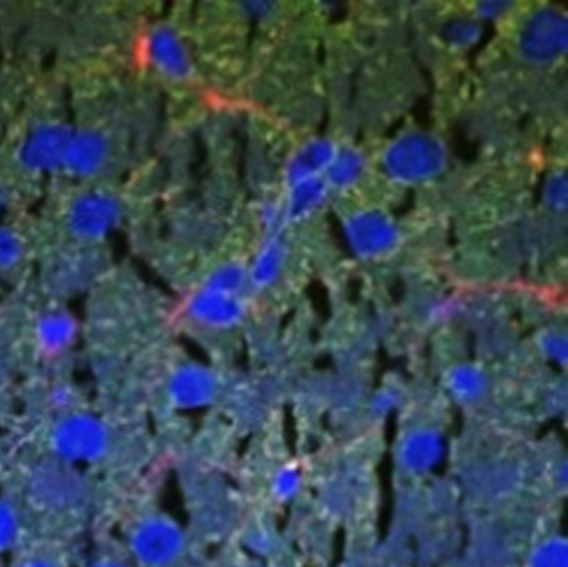

Supplement: Supplementary file 14 — Source Data Fig. 6 [file 44321_2024_32_MOESM14_ESM.zip › Figure 6/Figure 6B Merge Control ZZW-115.tif]

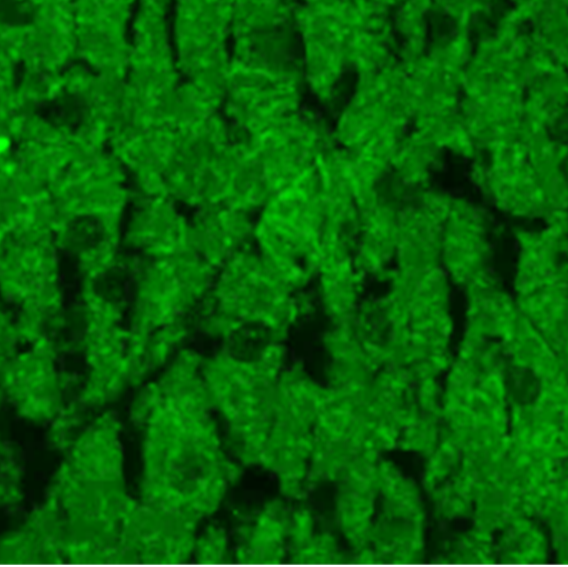

Supplement: Supplementary file 14 — Source Data Fig. 6 [file 44321_2024_32_MOESM14_ESM.zip › Figure 6/Figure 6B pEIF2 KC ZZW-115.tif]

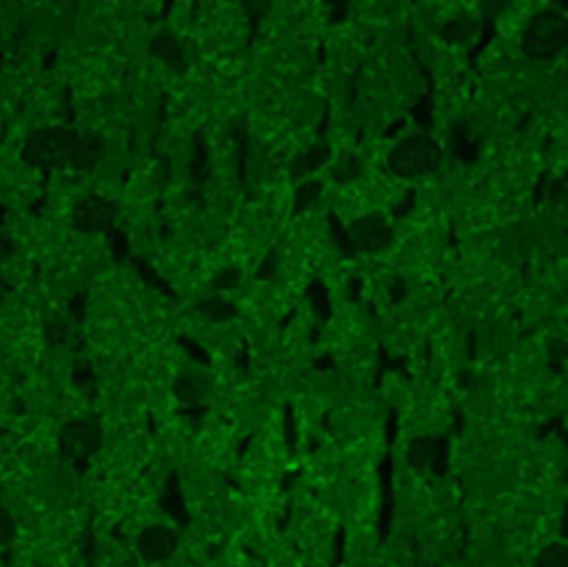

Supplement: Supplementary file 14 — Source Data Fig. 6 [file 44321_2024_32_MOESM14_ESM.zip › Figure 6/Figure 6B pEIF2 Control ZZW-115.tif]

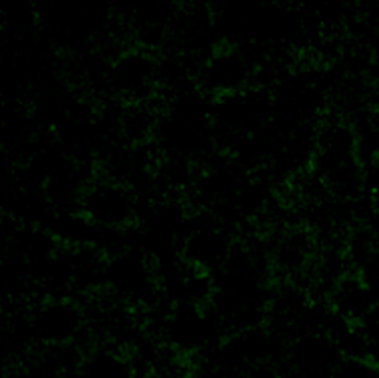

Supplement: Supplementary file 14 — Source Data Fig. 6 [file 44321_2024_32_MOESM14_ESM.zip › Figure 6/Figure 6A NUPR1 Control ZZW-115.tif]

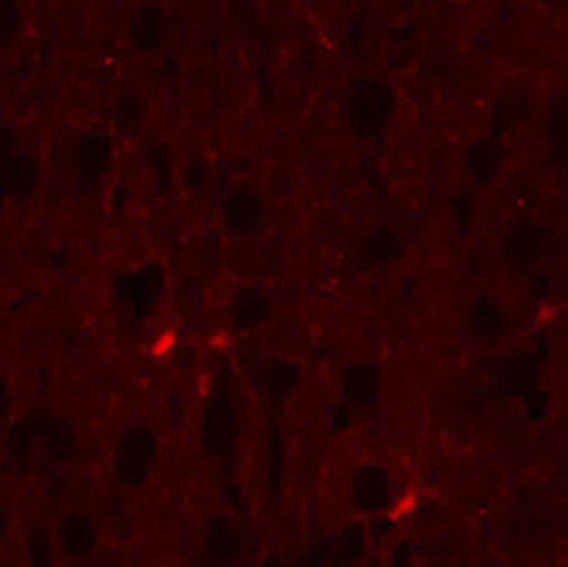

Supplement: Supplementary file 14 — Source Data Fig. 6 [file 44321_2024_32_MOESM14_ESM.zip › Figure 6/Figure 6B PABP Control Vehicle.tif]

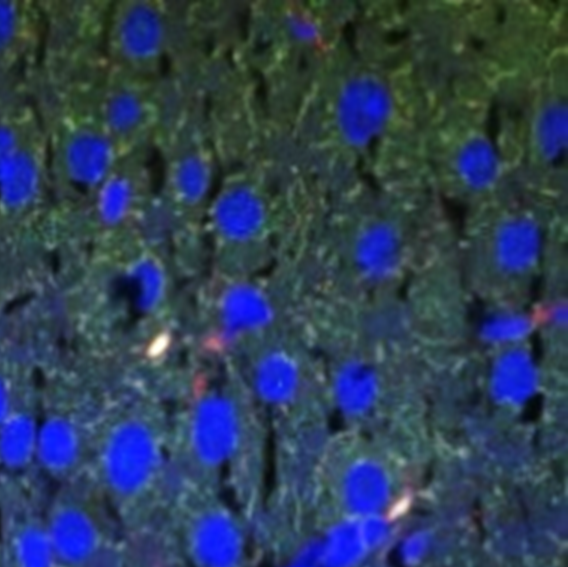

Supplement: Supplementary file 14 — Source Data Fig. 6 [file 44321_2024_32_MOESM14_ESM.zip › Figure 6/Figure 6B Merge Control Vehicle.tif]

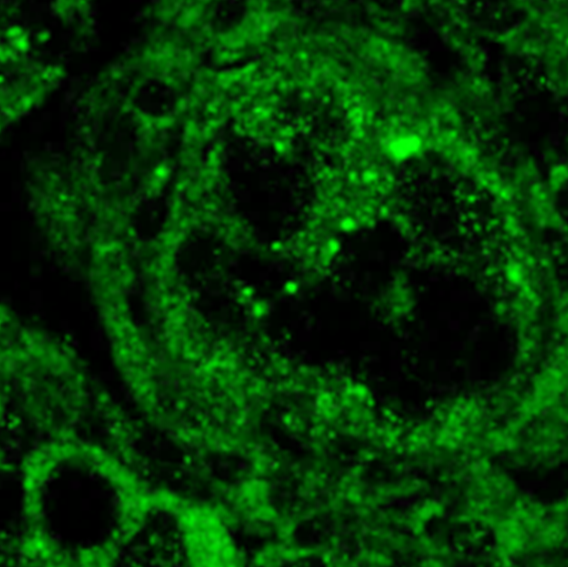

Supplement: Supplementary file 14 — Source Data Fig. 6 [file 44321_2024_32_MOESM14_ESM.zip › Figure 6/Figure 6B pEIF2 KC Vehicle.tif]

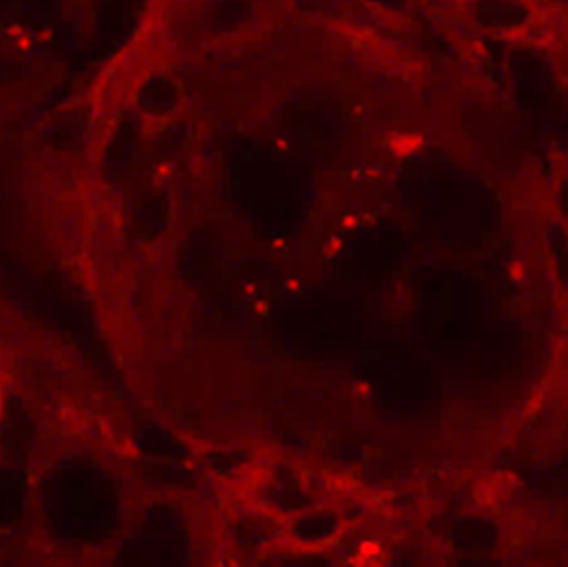

Supplement: Supplementary file 14 — Source Data Fig. 6 [file 44321_2024_32_MOESM14_ESM.zip › Figure 6/Figure 6B PABP KC Vehicle.tif]

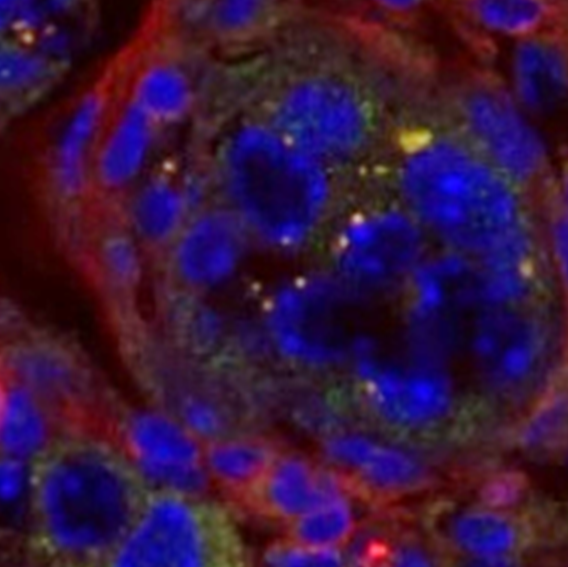

Supplement: Supplementary file 14 — Source Data Fig. 6 [file 44321_2024_32_MOESM14_ESM.zip › Figure 6/Figure 6B Merge KC Vehicle.tif]

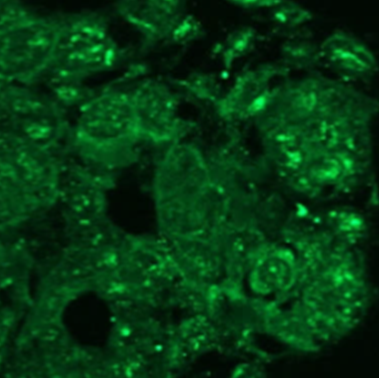

Supplement: Supplementary file 14 — Source Data Fig. 6 [file 44321_2024_32_MOESM14_ESM.zip › Figure 6/Figure 6A NUPR1 KC Vehicle.tif]

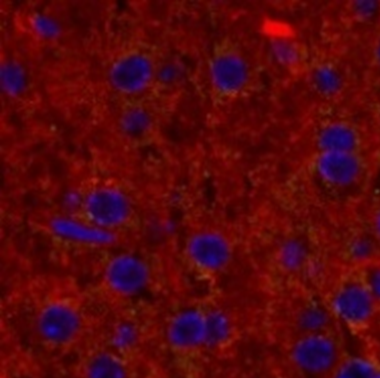

Supplement: Supplementary file 14 — Source Data Fig. 6 [file 44321_2024_32_MOESM14_ESM.zip › Figure 6/Figure 6A Merge Control ZZW-115.tif]

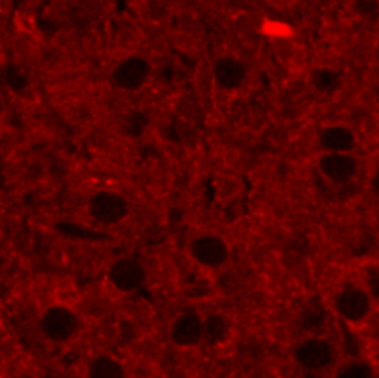

Supplement: Supplementary file 14 — Source Data Fig. 6 [file 44321_2024_32_MOESM14_ESM.zip › Figure 6/Figure 6A G3BP1 Control ZZW-115.tif]

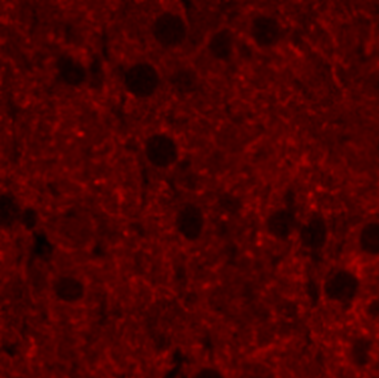

Supplement: Supplementary file 14 — Source Data Fig. 6 [file 44321_2024_32_MOESM14_ESM.zip › Figure 6/Figure 6A G3BP1 KC ZZW-115.tif]

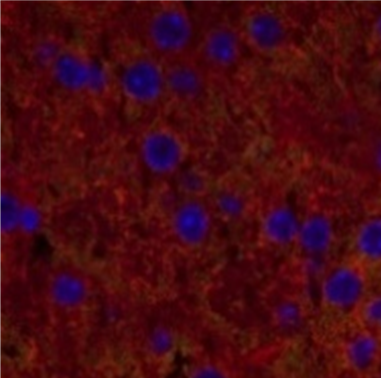

Supplement: Supplementary file 14 — Source Data Fig. 6 [file 44321_2024_32_MOESM14_ESM.zip › Figure 6/Figure 6A Merge KC ZZW-115.tif]

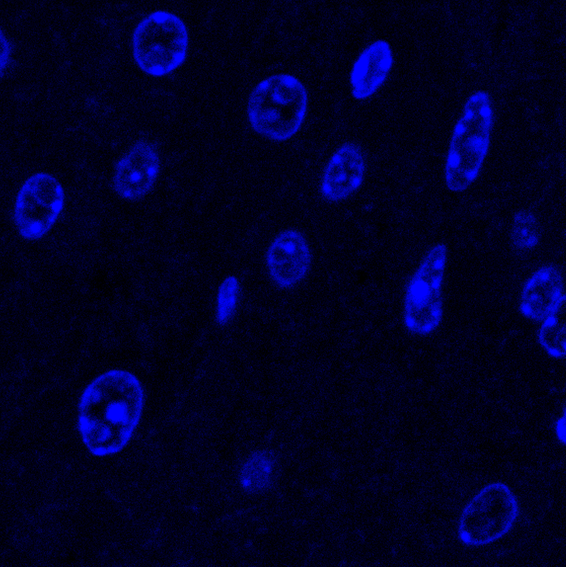

Supplement: Supplementary file 15 — Source Data Fig. 7 [file 44321_2024_32_MOESM15_ESM.zip › Figure 7/Merge Control Vehicle.tif]

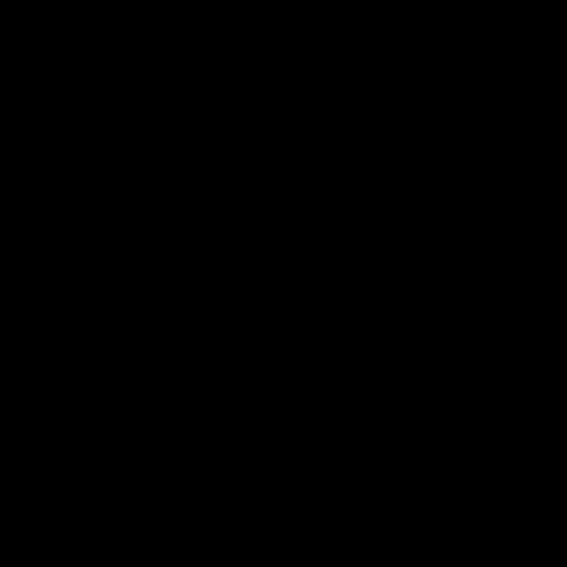

Supplement: Supplementary file 15 — Source Data Fig. 7 [file 44321_2024_32_MOESM15_ESM.zip › Figure 7/cleaved caspase-3 Control ZZW-115.tif]

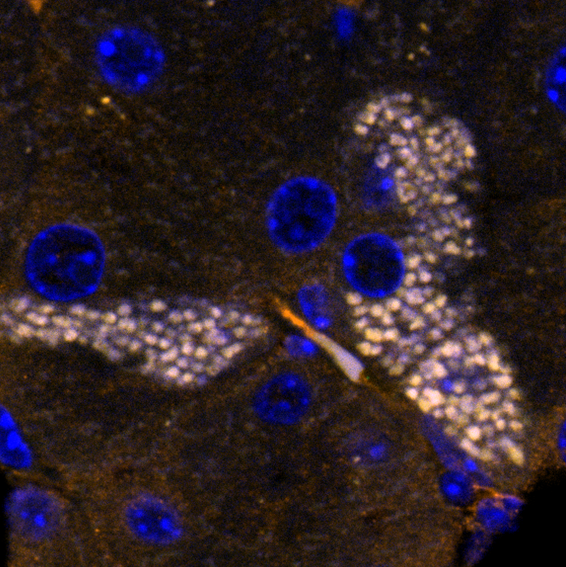

Supplement: Supplementary file 15 — Source Data Fig. 7 [file 44321_2024_32_MOESM15_ESM.zip › Figure 7/Merge KC ZZW-115.tif]

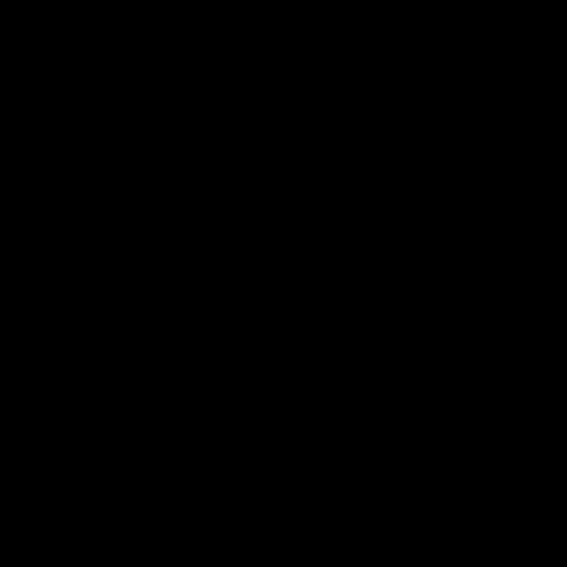

Supplement: Supplementary file 15 — Source Data Fig. 7 [file 44321_2024_32_MOESM15_ESM.zip › Figure 7/KrasG12D Control ZZW-115.tif]

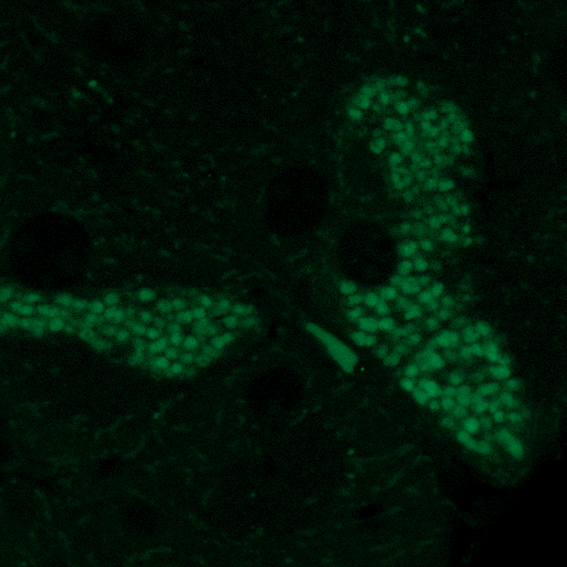

Supplement: Supplementary file 15 — Source Data Fig. 7 [file 44321_2024_32_MOESM15_ESM.zip › Figure 7/KrasG12D KC ZZW-115.tif]

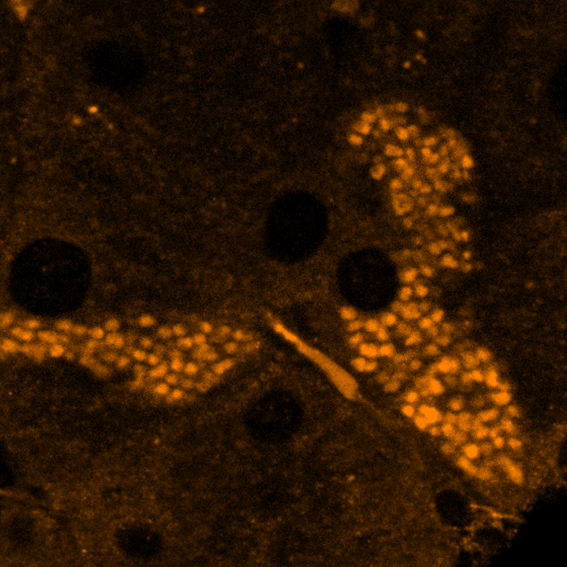

Supplement: Supplementary file 15 — Source Data Fig. 7 [file 44321_2024_32_MOESM15_ESM.zip › Figure 7/cleaved caspase-3 KC ZZW-115.tif]

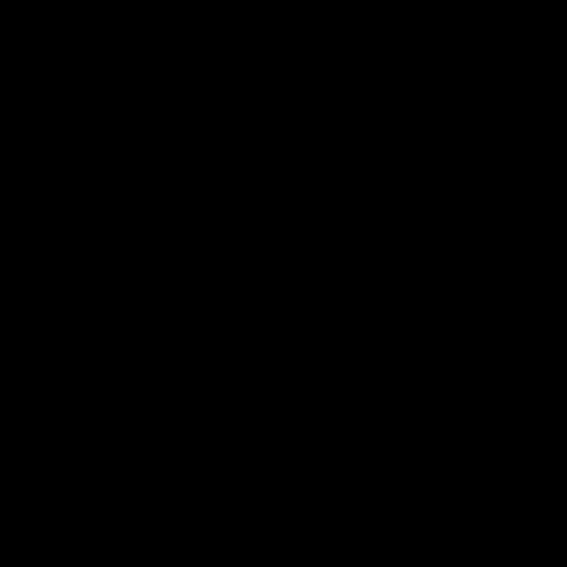

Supplement: Supplementary file 15 — Source Data Fig. 7 [file 44321_2024_32_MOESM15_ESM.zip › Figure 7/KrasG12D Control Vehicel.tif]

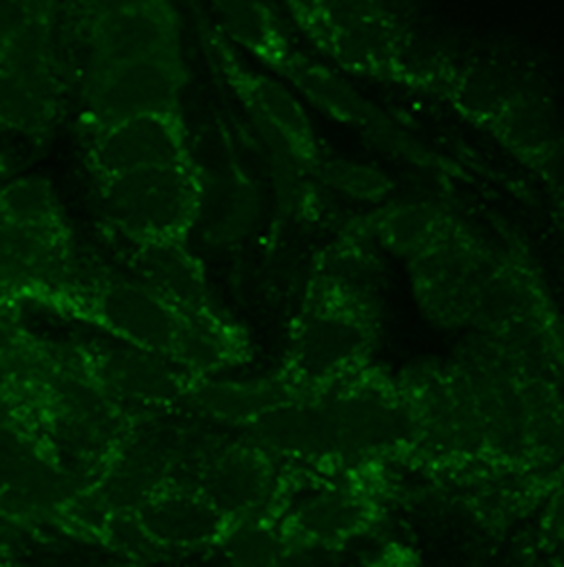

Supplement: Supplementary file 15 — Source Data Fig. 7 [file 44321_2024_32_MOESM15_ESM.zip › Figure 7/KrasG12D KC Vehicel.tif]

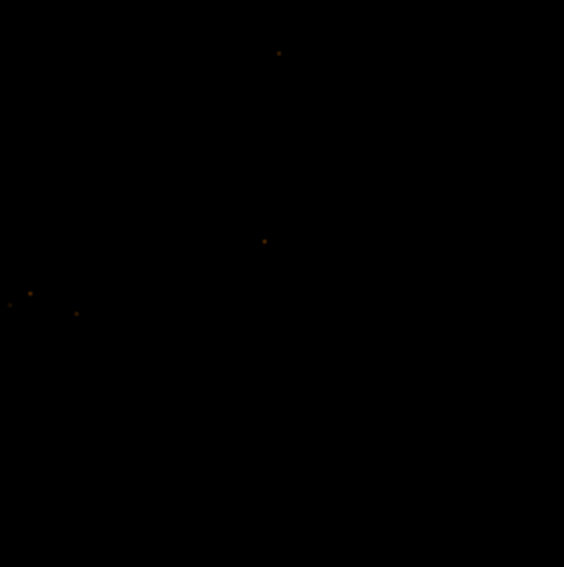

Supplement: Supplementary file 15 — Source Data Fig. 7 [file 44321_2024_32_MOESM15_ESM.zip › Figure 7/cleaved caspase-3 KC Vehicle.tif]

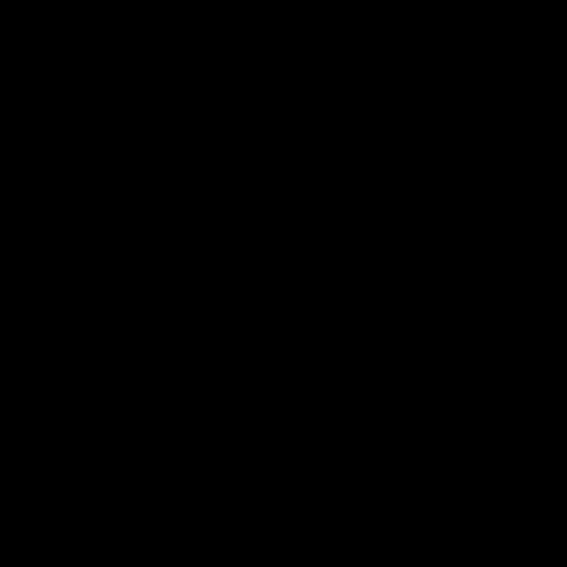

Supplement: Supplementary file 15 — Source Data Fig. 7 [file 44321_2024_32_MOESM15_ESM.zip › Figure 7/cleaved caspase-3 Control Vehicle.tif]

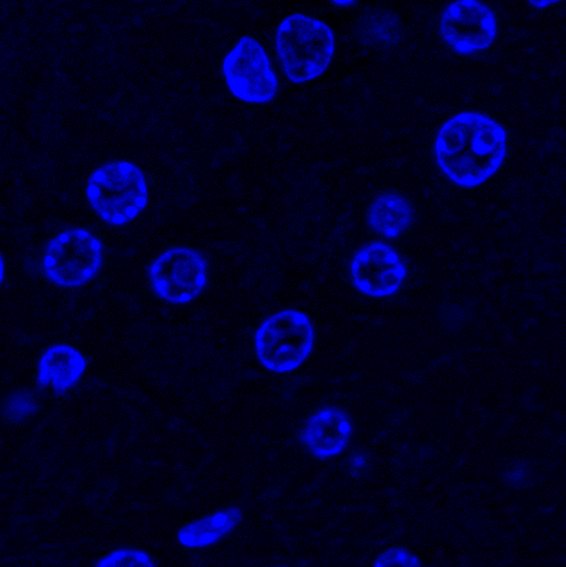

Supplement: Supplementary file 15 — Source Data Fig. 7 [file 44321_2024_32_MOESM15_ESM.zip › Figure 7/Merge Control ZZW-115.tif]

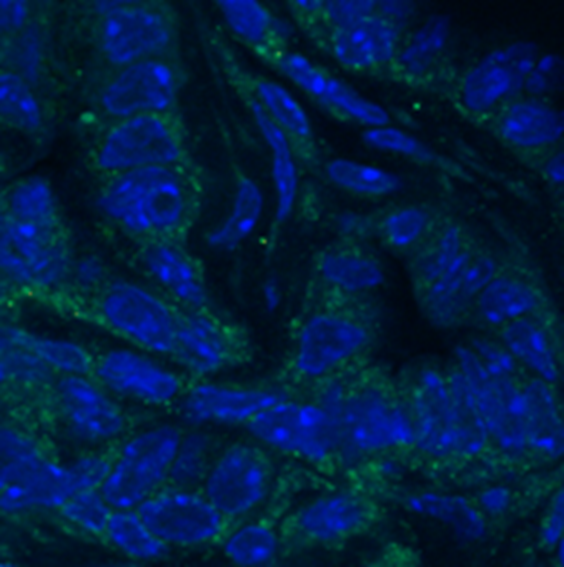

Supplement: Supplementary file 15 — Source Data Fig. 7 [file 44321_2024_32_MOESM15_ESM.zip › Figure 7/Merge KC Vehicle.tif]

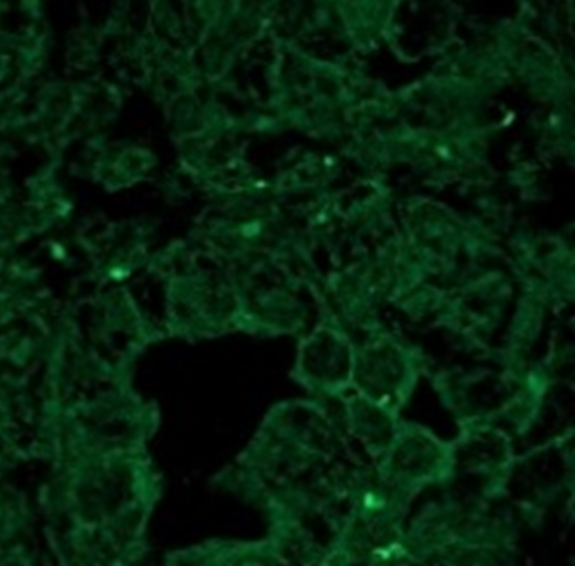

Supplement: Supplementary file 16 — Source Data Fig. 8 [file 44321_2024_32_MOESM16_ESM.zip › Figure 8/NUPR1 KC Vehicle.tif]

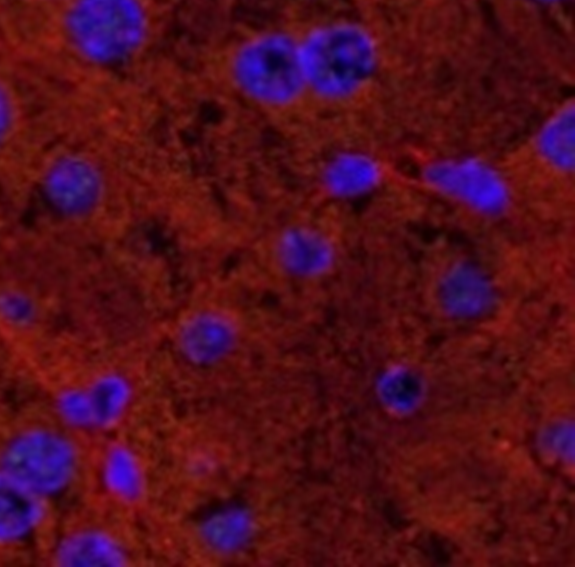

Supplement: Supplementary file 16 — Source Data Fig. 8 [file 44321_2024_32_MOESM16_ESM.zip › Figure 8/Merge Control Vehicle.tif]

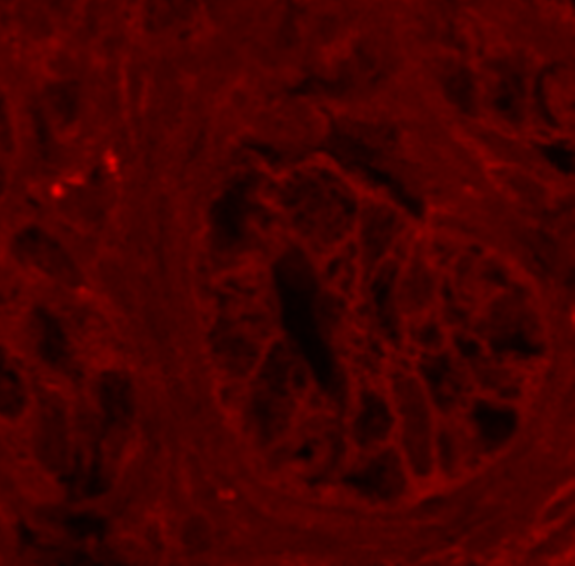

Supplement: Supplementary file 16 — Source Data Fig. 8 [file 44321_2024_32_MOESM16_ESM.zip › Figure 8/G3BP1 KC ZZW-115.tif]

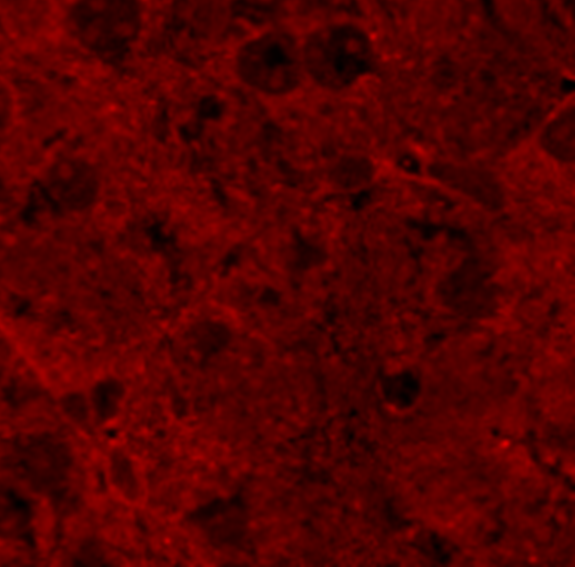

Supplement: Supplementary file 16 — Source Data Fig. 8 [file 44321_2024_32_MOESM16_ESM.zip › Figure 8/G3BP1 Control Vehicle.tif]

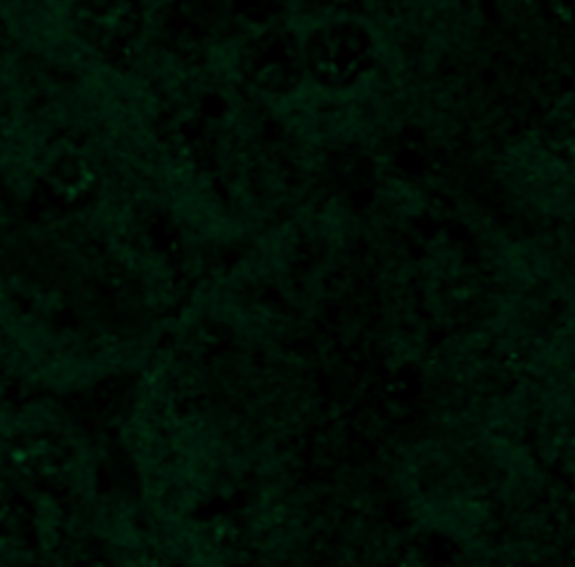

Supplement: Supplementary file 16 — Source Data Fig. 8 [file 44321_2024_32_MOESM16_ESM.zip › Figure 8/NUPR1 Control Vehicle.tif]

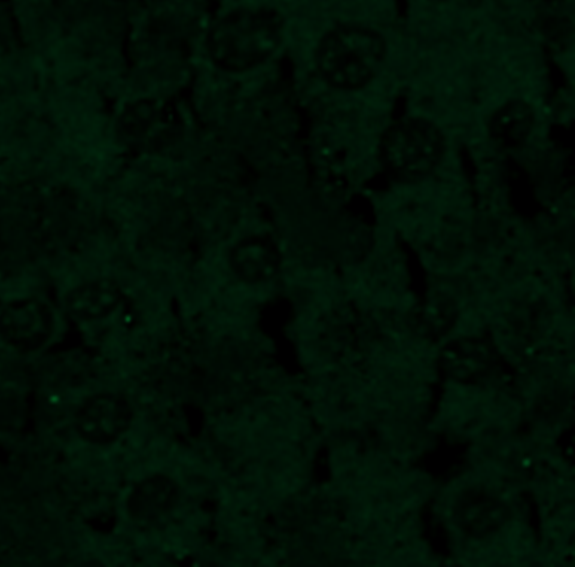

Supplement: Supplementary file 16 — Source Data Fig. 8 [file 44321_2024_32_MOESM16_ESM.zip › Figure 8/NUPR1 Control ZZW-115.tif]

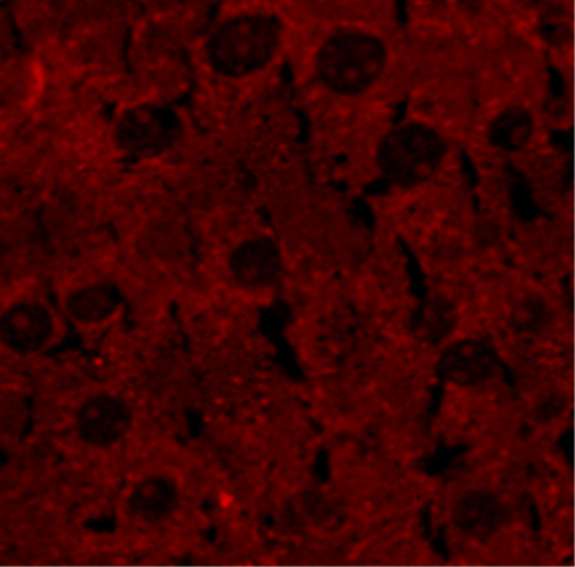

Supplement: Supplementary file 16 — Source Data Fig. 8 [file 44321_2024_32_MOESM16_ESM.zip › Figure 8/G3BP1 Control ZZW-115.tif]

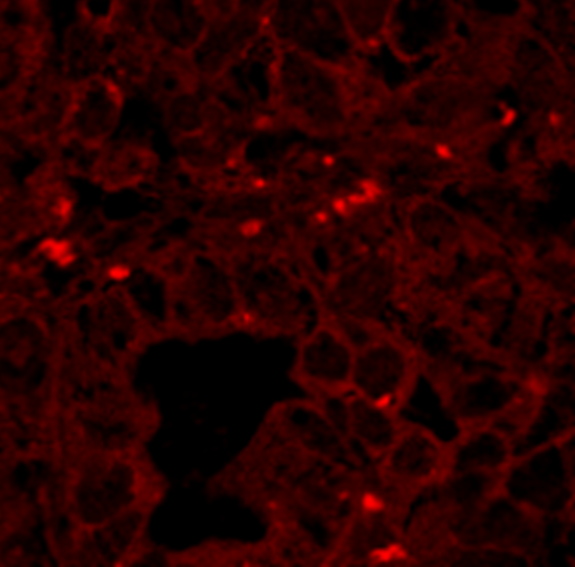

Supplement: Supplementary file 16 — Source Data Fig. 8 [file 44321_2024_32_MOESM16_ESM.zip › Figure 8/G3BP1 KC Vehicle.tif]

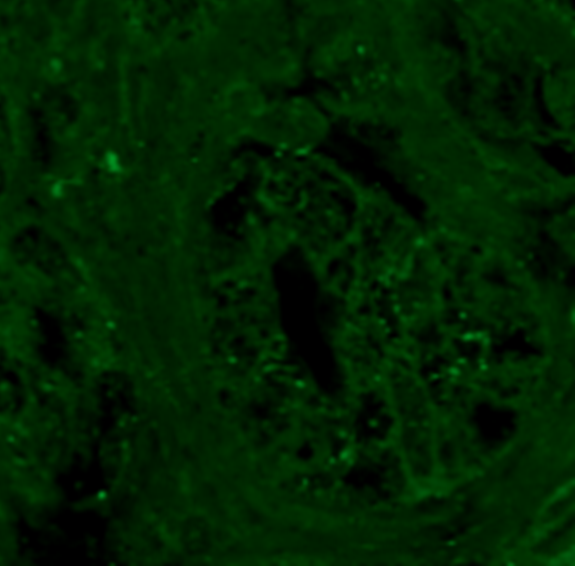

Supplement: Supplementary file 16 — Source Data Fig. 8 [file 44321_2024_32_MOESM16_ESM.zip › Figure 8/NUPR1 KC ZZW-115.tif]

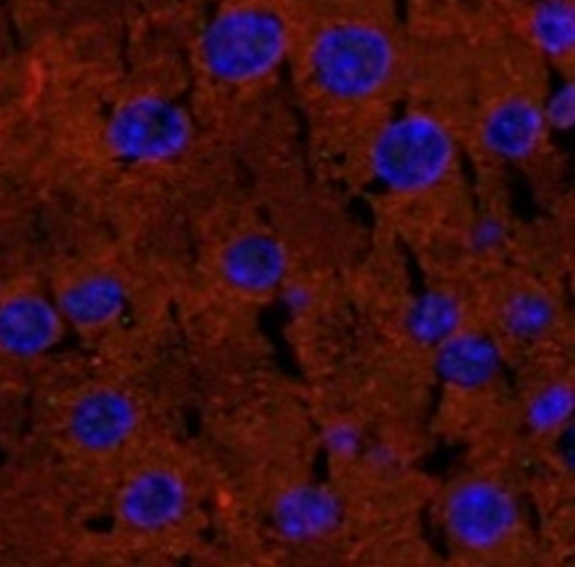

Supplement: Supplementary file 16 — Source Data Fig. 8 [file 44321_2024_32_MOESM16_ESM.zip › Figure 8/Merge Control ZZW-115.tif]

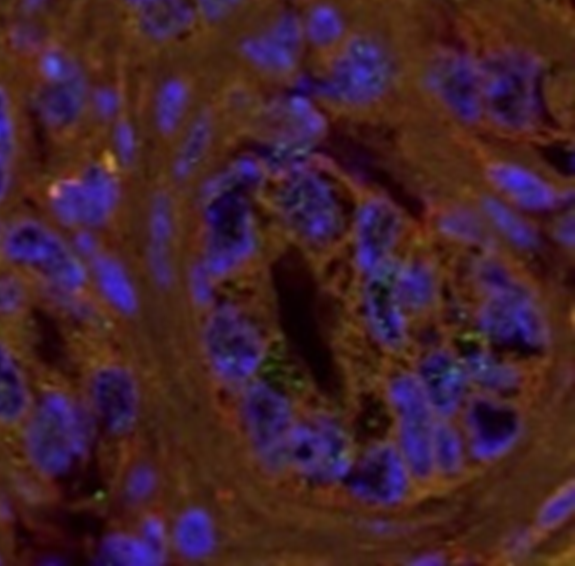

Supplement: Supplementary file 16 — Source Data Fig. 8 [file 44321_2024_32_MOESM16_ESM.zip › Figure 8/Merge KC ZZW-115.tif]

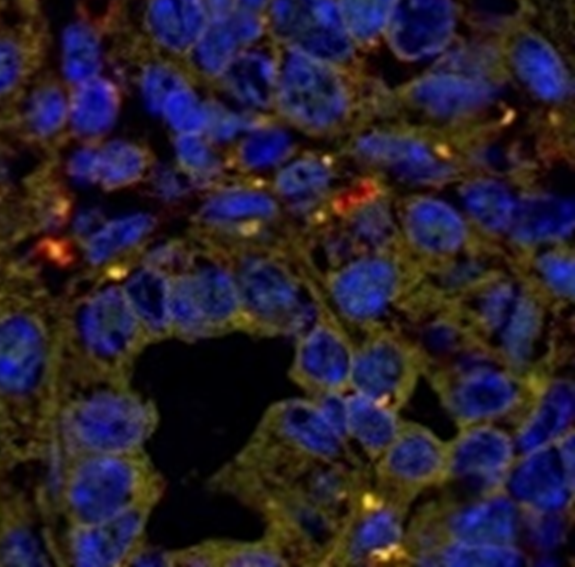

Supplement: Supplementary file 16 — Source Data Fig. 8 [file 44321_2024_32_MOESM16_ESM.zip › Figure 8/Merge KC Vehicle.tif]
