# Supplementary material for: Targeting NUPR1-dependent stress granules formation to induce synthetic lethality in KrasG12D-driven tumors
Source: EMBO Mol Med. 2024 Feb 15;16(3):4. doi: 10.1038/s44321-024-00032-2 (PMC10940650; doi:10.1038/s44321-024-00032-2)
Supplement: Supplementary file 13 — Source Data Fig. 5 [file 44321_2024_32_MOESM13_ESM.zip › Figure 5/Figure5H .pptx]

## Slide 1
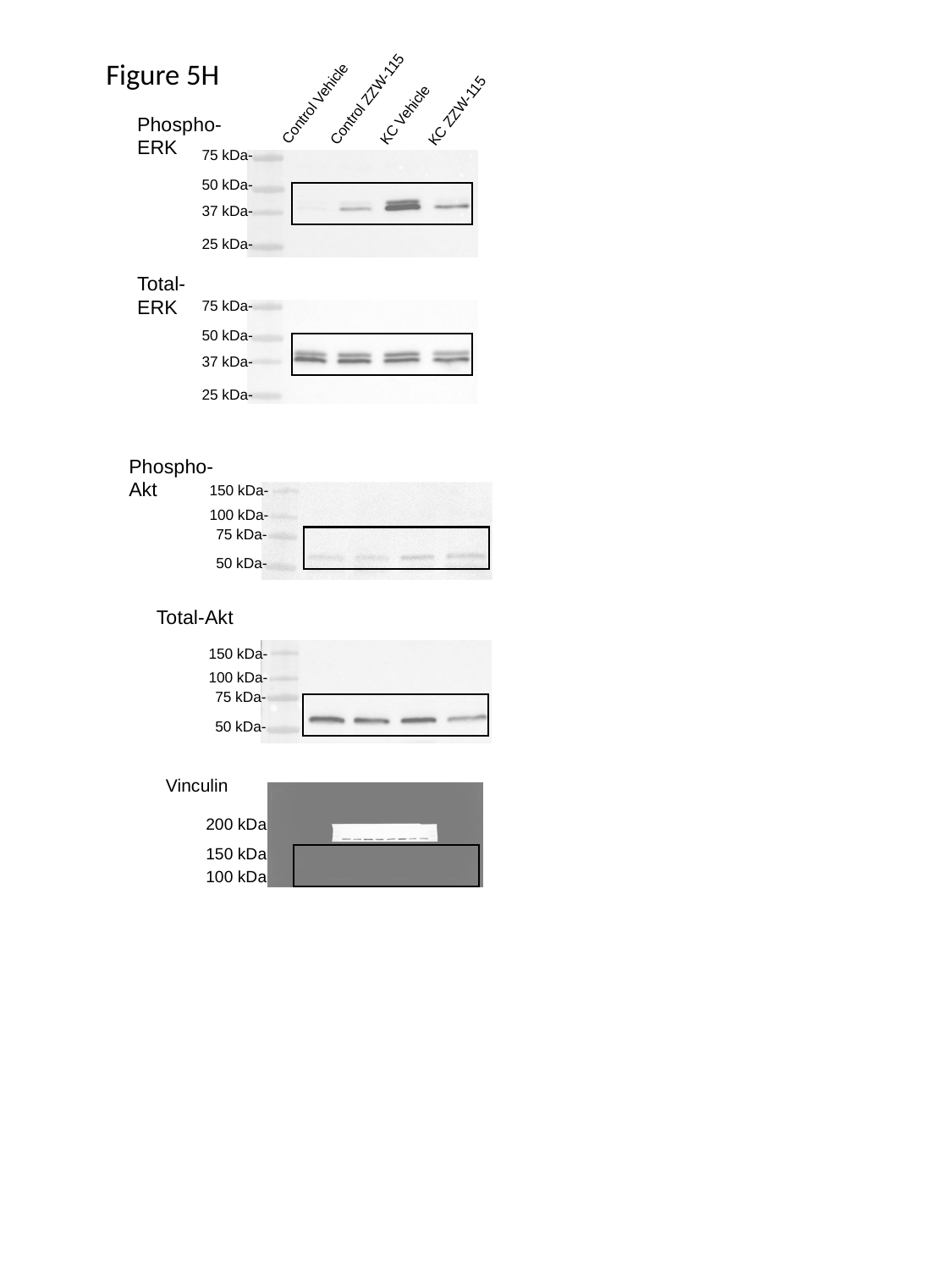

Figure 5H
Control ZZW-115
Control Vehicle
KC ZZW-115
KC Vehicle
Phospho-ERK
75 kDa-
50 kDa-
37 kDa-
25 kDa-
Total-ERK
75 kDa-
50 kDa-
37 kDa-
25 kDa-
Phospho-Akt
150 kDa-
100 kDa-
75 kDa-
50 kDa-
Total-Akt
150 kDa-
100 kDa-
75 kDa-
50 kDa-
Vinculin
200 kDa
150 kDa
100 kDa
