# Supplementary figures and images for: Targeting NUPR1-dependent stress granules formation to induce synthetic lethality in KrasG12D-driven tumors (part 3 of 3)
Source: EMBO Mol Med. 2024 Feb 15;16(3):4. doi: 10.1038/s44321-024-00032-2 (PMC10940650; doi:10.1038/s44321-024-00032-2)

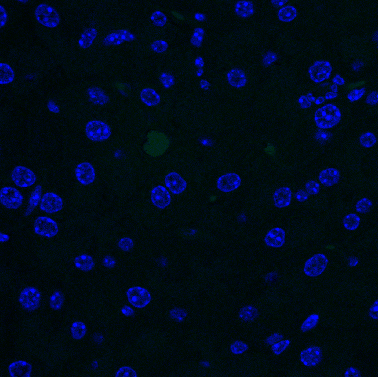

Supplement: Supplementary file 17 — Source Data Fig. 9 [file 44321_2024_32_MOESM17_ESM.zip › Figure 9/Figure 9B Vehicle control.tif]

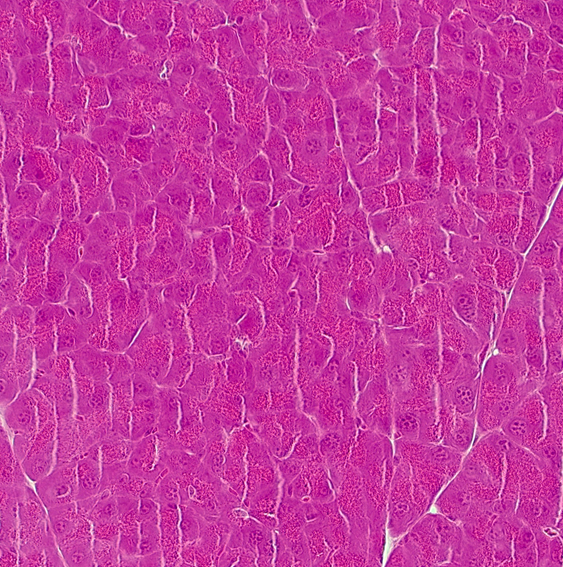

Supplement: Supplementary file 17 — Source Data Fig. 9 [file 44321_2024_32_MOESM17_ESM.zip › Figure 9/Figure 9A Vehicle control.tif]

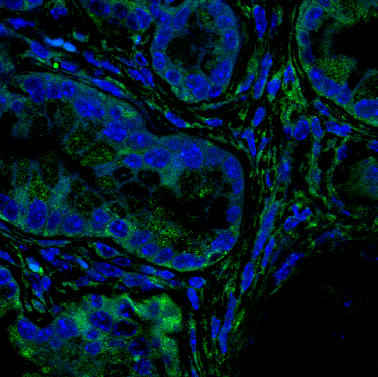

Supplement: Supplementary file 17 — Source Data Fig. 9 [file 44321_2024_32_MOESM17_ESM.zip › Figure 9/Figure 9B ZZW-115 KC.tif]

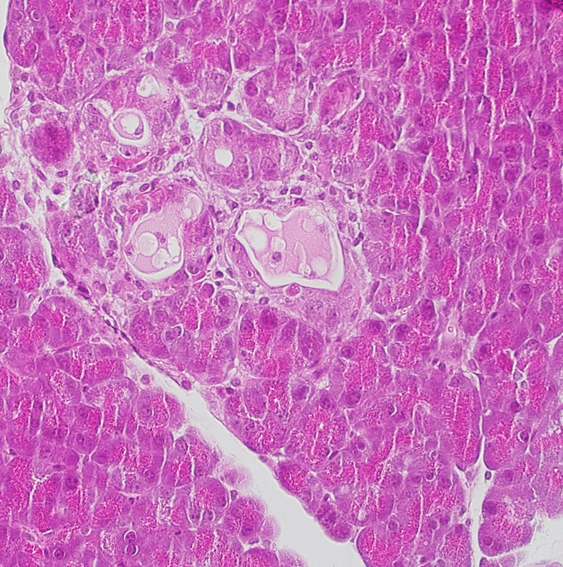

Supplement: Supplementary file 17 — Source Data Fig. 9 [file 44321_2024_32_MOESM17_ESM.zip › Figure 9/Figure 9A ZZW-115 KC.tif]

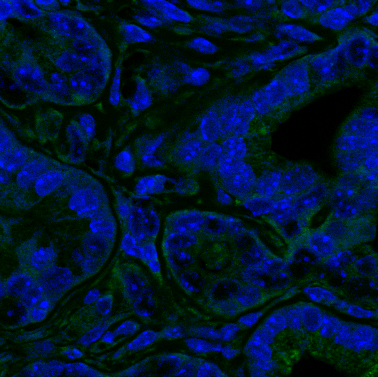

Supplement: Supplementary file 17 — Source Data Fig. 9 [file 44321_2024_32_MOESM17_ESM.zip › Figure 9/Figure 9B Vehicle KC.tif]

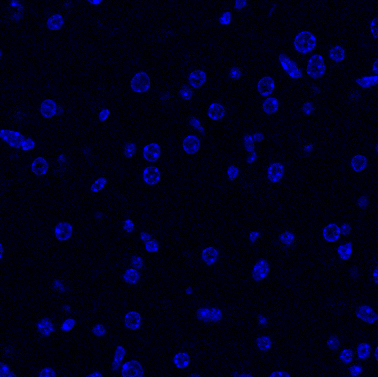

Supplement: Supplementary file 17 — Source Data Fig. 9 [file 44321_2024_32_MOESM17_ESM.zip › Figure 9/Figure 9B ZZW-115 control.tif]

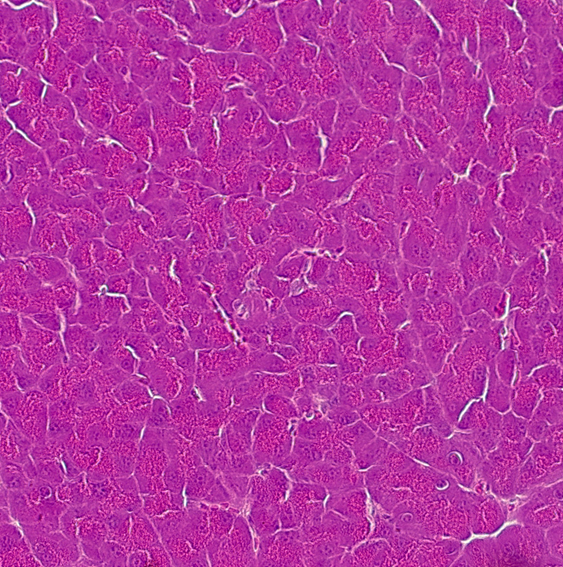

Supplement: Supplementary file 17 — Source Data Fig. 9 [file 44321_2024_32_MOESM17_ESM.zip › Figure 9/Figure 9A ZZW-115 control.tif]

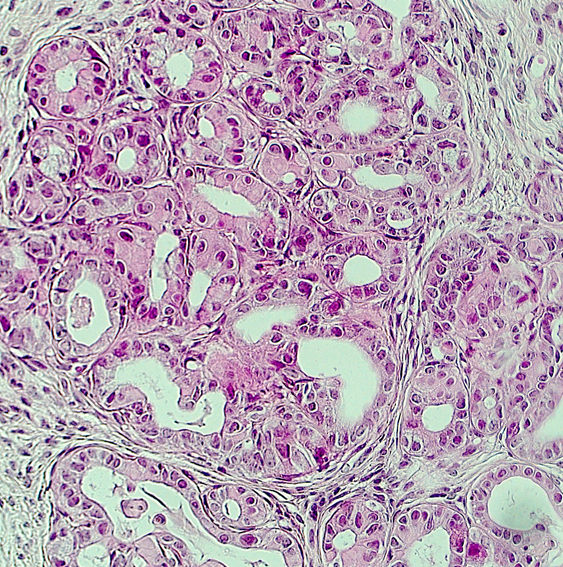

Supplement: Supplementary file 17 — Source Data Fig. 9 [file 44321_2024_32_MOESM17_ESM.zip › Figure 9/Figure 9A Vehicle KC.tif]
