# Supplementary material for: Targeting NUPR1-dependent stress granules formation to induce synthetic lethality in KrasG12D-driven tumors
Source: EMBO Mol Med. 2024 Feb 15;16(3):4. doi: 10.1038/s44321-024-00032-2 (PMC10940650; doi:10.1038/s44321-024-00032-2)
Supplement: Supplementary file 20 — Expanded View Figures [file 44321_2024_32_MOESM20_ESM.pdf]

Expanded View Figures

Figure EV1. NUPR1 is a key protein for the formation of SGs.

(**A**) NUPR1 and G3BP1 mRNA levels were measured in 4292 i-*Kras*, 4668 i-*Kras* and 9805 i-*Kras* cells 48 h after transfection, expressed as fold changes ($n = 3$ independent experiments, triplicates were made on each one). Data represent mean ± SD. One-way ANOVA, Sidak correction. (**B**) Western-blot analysis was performed in MiaPaCa-2 cells to evaluate G3BP1, NUPR1 and vinculin levels ($n = 3$). (**C**) Immunofluorescence was performed in MiaPaCa-2 cells transfected with siControl or siNUPR1 and after 24 h, with G3BP1 or GFP plasmid, cells were fix 24 h later. Mouse anti-G3BP1 and Alexa 568-labeled goat anti-mouse secondary antibodies were used. A representative experiment is shown ($n = 2$). (**D**) Cell count of MiaPaCa-2 cells in the previous conditions was evaluated ($n = 3$ independent experiments, triplicates were made on each one), data represent mean ± SD, two-way ANOVA, Sidak correction. (**E**) Immunofluorescence was performed in PDAC primary cell lines, mouse anti-G3BP1 and Alexa 568-labeled goat anti-mouse secondary antibodies were used ($n = 3$). (**F**) Chemogram assays were done on pancreatic cancer cell lines with increasing concentrations of ZZW-115 for 72 h ($n = 3$). Source data are available online for this figure.

© The Author(s)

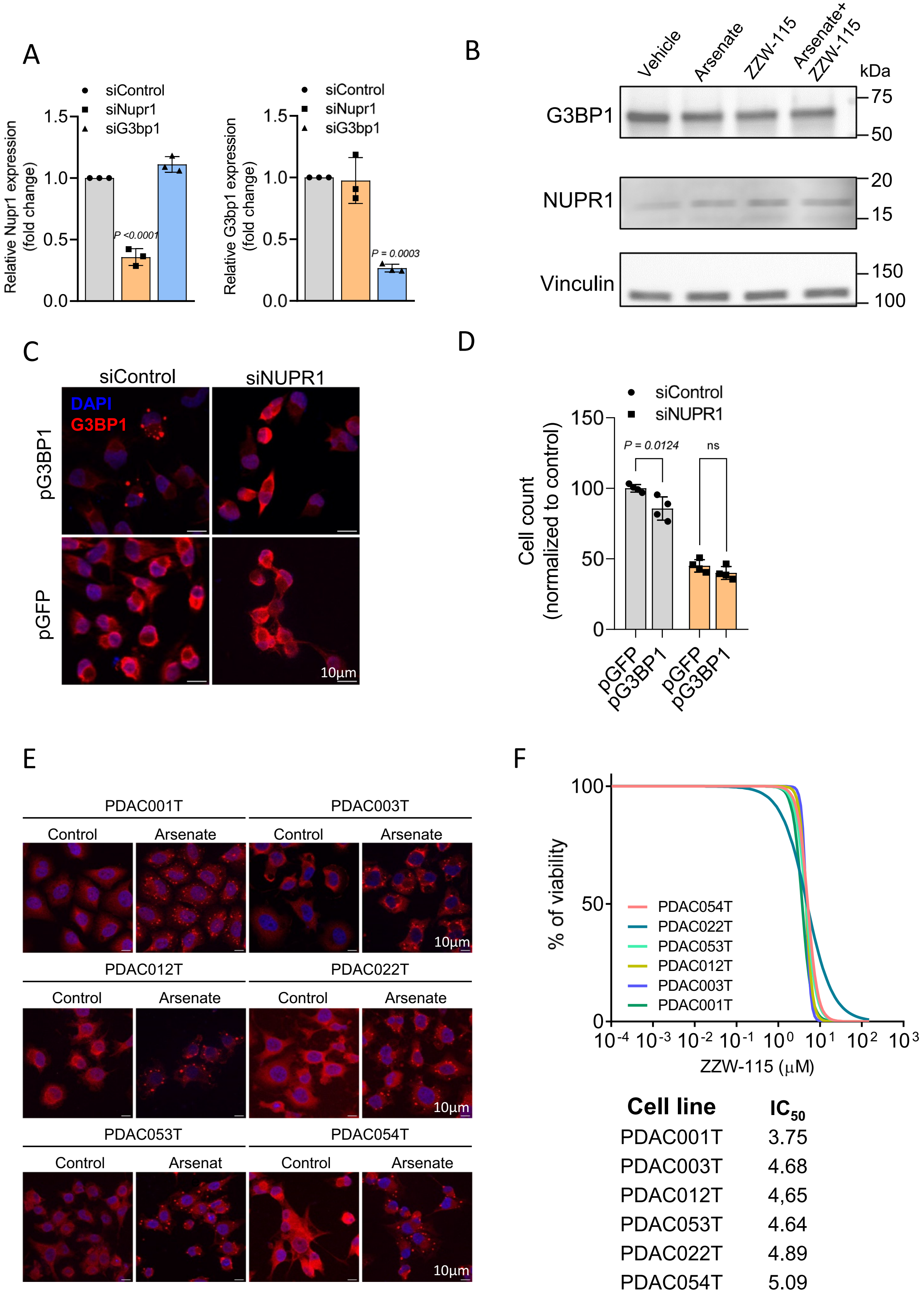
A
siControl
siNupr1
siG3bp1
Relative Nupr1 expression (fold change)
P <0.0001
Relative G3bp1 expression (fold change)
P = 0.0003
1.5
1.0
0.5
0.0
B
Vehicle
Arsenate
ZZW-115
Arsenate+ ZZW-115
kDa
G3BP1
NUPR1
Vinculin
75
50
20
15
150
100
C
siControl
siNUPR1
DAPI
G3BP1
pG3BP1
pGFP
10μm
D
siControl
siNUPR1
Cell count (normalized to control)
P = 0.0124
ns
150
100
50
0
pGFP
pG3BP1
E
PDAC001T
PDAC003T
Control
Arsenate
10μm
PDAC012T
PDAC022T
PDAC053T
PDAC054T
Arsenat
F
% of viability
100
50
0
PDAC054T
PDAC022T
PDAC053T
PDAC012T
PDAC003T
PDAC001T
10-4 10-3 10-2 10-1 100 101 102 103
ZZW-115 (μM)
Cell line IC50
PDAC001T 3.75
PDAC003T 4.68
PDAC012T 4,65
PDAC053T 4.64
PDAC022T 4.89
PDAC054T 5.09

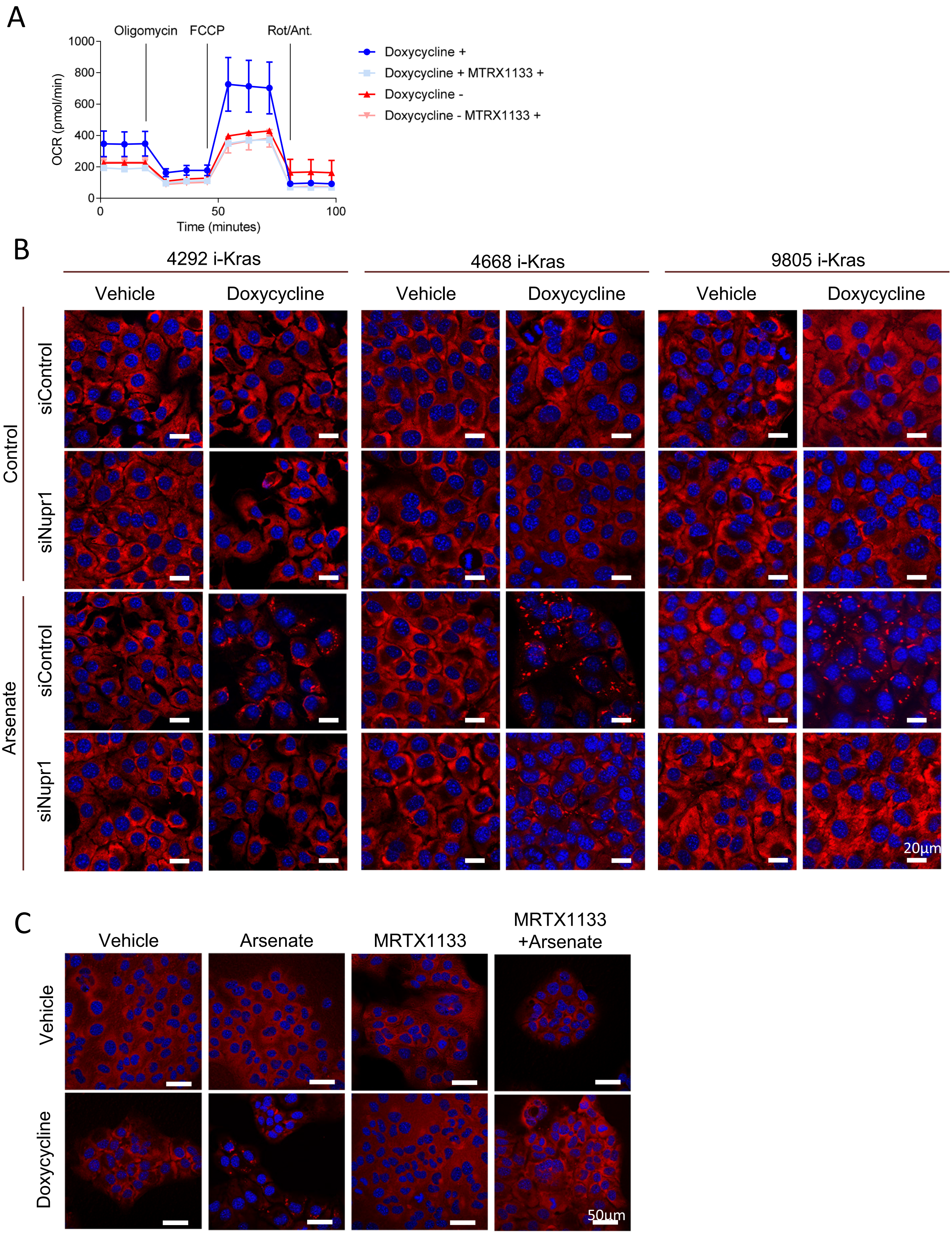
A
Oligomycin
FCCP
Rot/Ant.
OCR (pmol/min)
1000
800
600
400
200
0
0
50
100
Time (minutes)
Doxycycline +
Doxycycline + MTRX1133 +
Doxycycline -
Doxycycline - MTRX1133 +
B
4292 i-Kras
4668 i-Kras
9805 i-Kras
Vehicle
Doxycycline
Control
Arsenate
siControl
siNupr1
20μm
C
Vehicle
Arsenate
MRTX1133
MRTX1133 +Arsenate
Doxycycline
50μm

Figure EV2. Inhibition of NUPR1 by siRNA or *Kras* inhibitor MRTX1133 prevents SGs formation in i-*Kras* cells.

(**A**) OXPHOS metabolism, reflected by oxygen consumption rate (OCR) levels, were measured in 9805 i-*Kras* cells in the absence of presence of doxycycline and/or 30 nM of MRTX1133 for 24 h, a representative experiment is shown (data represent mean ± SEM, $n = 3$ independent experiments, triplicates were made on each one). (**B**) Immunofluorescence staining was performed in 4292 i-*Kras*, 4668 i-*Kras* and 9805 i-*Kras* cells 48 h after transfection with siControl of siNUPR1. Mouse anti-G3BP1 and then, Alexa 568-labeled goat anti-mouse secondary antibody were used ($n = 3$). (**C**) Immunofluorescence staining was performed in 9805 i-*Kras* cells in the absence of presence of arsenate and/or 30 nM of MRTX1133 for 24 h. Mouse anti-G3BP1 and Alexa 568-labeled goat anti-mouse secondary antibodies were used ($n = 3$). Source data are available online for this figure.

© The Author(s)

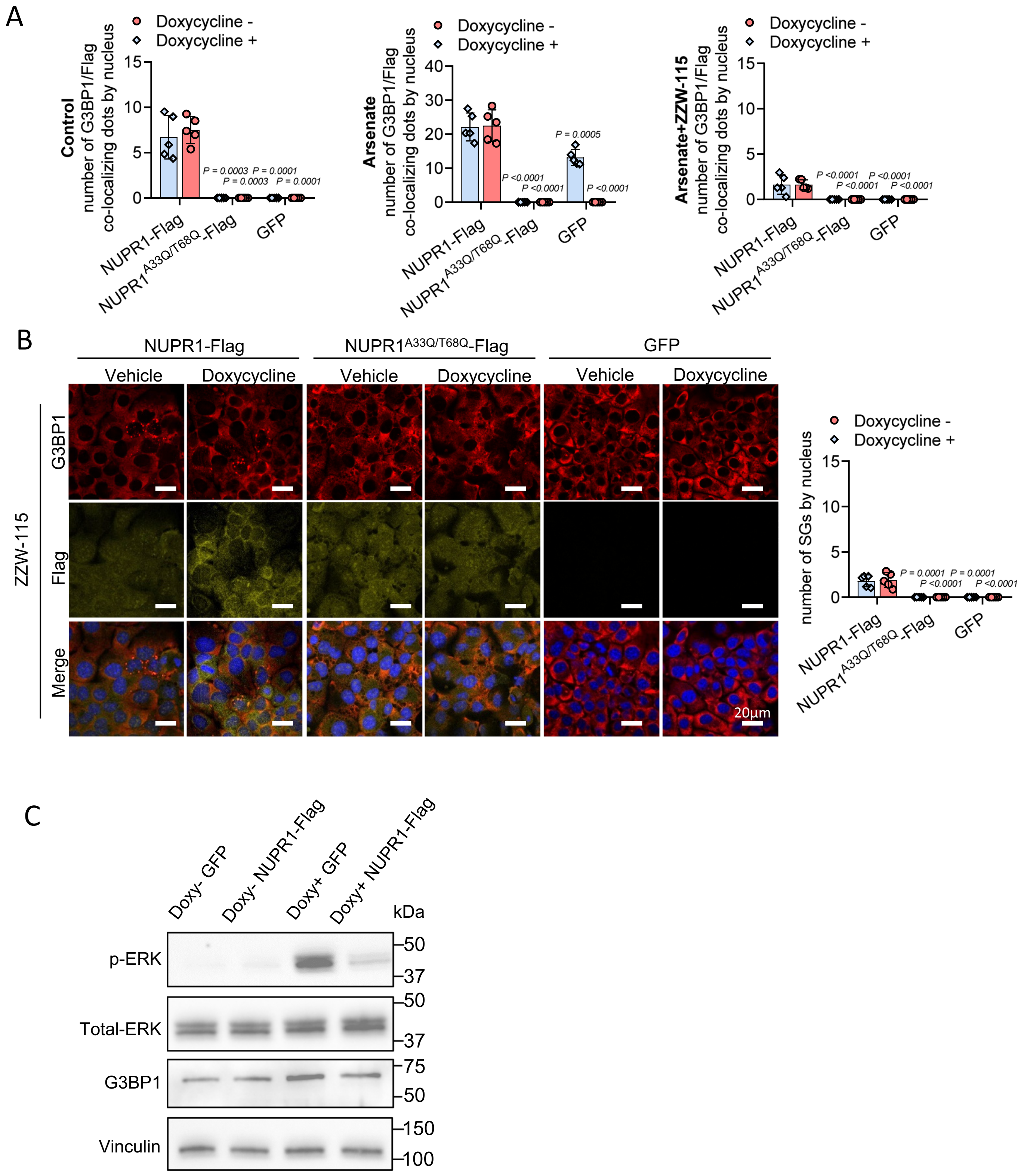
A
Doxycycline -
Doxycycline +
Control
number of G3BP1/Flag co-localizing dots by nucleus
Arsenate
Arsenate+ZZW-115
NUPR1-Flag
NUPR1A33Q/T68Q-Flag
GFP
P = 0.0003
P = 0.0001
P = 0.0005
P <0.0001
B
NUPR1-Flag
NUPR1A33Q/T68Q-Flag
GFP
Vehicle
Doxycycline
ZZW-115
G3BP1
Flag
Merge
20µm
number of SGs by nucleus
C
Doxy- GFP
Doxy- NUPR1-Flag
Doxy+ GFP
Doxy+ NUPR1-Flag
kDa
p-ERK
Total-ERK
G3BP1
Vinculin

Figure EV3. ZZW-115 prevents SGs formation in cells overexpressing NUPR1 independent of *Kras* signaling.

(**A**) Quantification of number of G3BP1 or NUPR1 by nucleus or number of colocalizing NUPR1/G3BP1 dots by nucleus in 9805 i-*Kras* cells is shown ($n = 5$). Data represent mean ± SD, two-way ANOVA with Sidak correction. (**B**) Immunofluorescence staining was performed in 9805 i-*Kras* cells 24 h post-transfection of NUPR1-Flag wild-type, its double mutant NUPR1 A33Q /T68Q-Flag, or a GFP plasmids upon treatment with ZZW-115 at 6 μM for 6 h. Mouse anti-G3BP1 and rabbit anti-Flag and then, Alexa 568-labeled goat anti-mouse and Alexa 647-labeled goat anti-rabbit secondary antibodies were used ($n = 5$ independent experiments, 5 pictures were used to calculate the mean of each experiment). Data represent mean ± SD, two-way ANOVA with Sidak correction. (**C**) Western blot analysis was performed in 9805 i-*Kras* cells to evaluate p-ERK, total-ERK, G3BP1 and vinculin levels ($n = 3$). Source data are available online for this figure.

 © The Author(s)

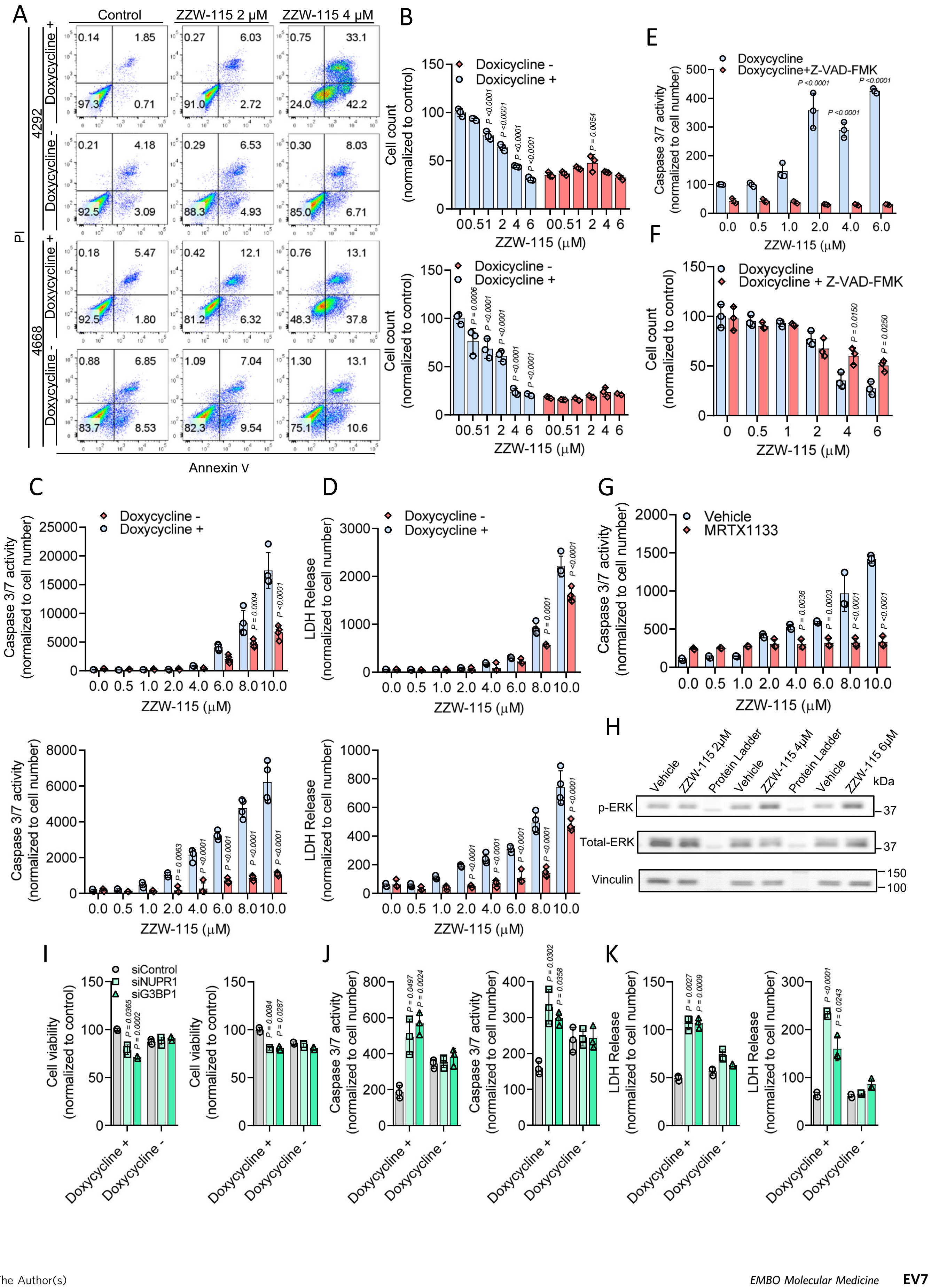
A
Control
ZZW-115 2 μM
ZZW-115 4 μM
4292
4668
Doxycycline +
Doxycycline -
PI
Annexin V
B
Doxicycline -
Doxicycline +
Cell count
(normalized to control)
ZZW-115 (μM)
C
Doxycycline -
Doxycycline +
Caspase 3/7 activity
(normalized to cell number)
ZZW-115 (μM)
D
LDH Release
(normalized to cell number)
E
Doxycycline
Doxycycline+Z-VAD-FMK
F
Doxycycline
Doxicycline + Z-VAD-FMK
G
Vehicle
MRTX1133
H
Vehicle
ZZW-115 2μM
Protein Ladder
ZZW-115 4μM
ZZW-115 6μM
kDa
p-ERK
Total-ERK
Vinculin
I
siControl
siNUPR1
siG3BP1
Cell viability
(normalized to control)
Doxycycline +
Doxycycline -
J
K

Figure EV4. Inhibition of NUPR1 induced cell death in *Kras*^{G12D}-activated cells.

(**A**) Flow cytometry analysis of annexin V/PI staining of 4292 i-*Kras* (up), 4668 i-*Kras* (down) cells, following 24 h of treatment with increasing concentrations of ZZW-115 in presence or in the absence of doxycycline was done. A representative experiment of the dot plot profile of cells is shown ($n = 3$). (**B**) Cell count of 4292 i-*Kras* (up), 4668 i-*Kras* (down) cells measured by IncuCyte live-cell imaging after 30 h of treatment of increasing concentrations of ZZW-115 in the presence or in the absence of doxycycline was evaluated ($n = 3$ independent experiments, triplicates were made on each one). Data represent mean ± SD, Two-way ANOVA with Sidak correction. 4292 i-*Kras* (up), 4668 i-*Kras* (down) cells were incubated at increasing concentrations ZZW-115 in presence or in the absence of doxycycline for 24 h and (**C**) caspase 3/7 activity ($n = 3$) and (**D**) LDH release were measured ($n = 3$). For both, data represent mean ± SD, Two-way ANOVA with Sidak correction. (**E**) Caspase 3/7 activity was measured in 9805 i-*Kras* cells after 24 h of treatment of increasing concentrations of ZZW-115 in the presence or in the absence of Z-VAD-FMK. Data represent mean ± SD, ($n = 3$) two-way ANOVA with Sidak correction. (**F**) Cell count was measured in 9805 i-*Kras* cells after 24 h of treatment of increasing concentrations of ZZW-115 in the presence or in the absence of Z-VAD-FMK ($n = 3$). Data represent mean ± SD, two-way ANOVA with Sidak correction. (**G**) Caspase 3/7 activity was measured in 9805 i-*Kras* cells after 24 h of treatment at increasing concentrations of ZZW-115 in the presence or in the absence of 30 nM of MRTX1133 (n = 3). Data represent mean ± SD, two-way ANOVA with Sidak correction. (**H**) Western blot analysis was performed in 9805 i-*Kras* cells to evaluate p-ERK, total-ERK and vinculin levels upon ZZW-115-treatment ($n = 3$). (**I**) Cell viability (**J**) caspase 3/7 activity and (**K**) LDH release were measured in 4292 i-*Kras* (left), 4668 i-*Kras* (right) cells transfected with siControl, siG3BP1 or siNupr1 for 48 h in the presence or absence of doxycycline ($n = 3$ independent experiments, triplicates were made on each one). Data represent mean ± SD, Two-way ANOVA with Sidak correction. Source data are available online for this figure.

 © The Author(s)

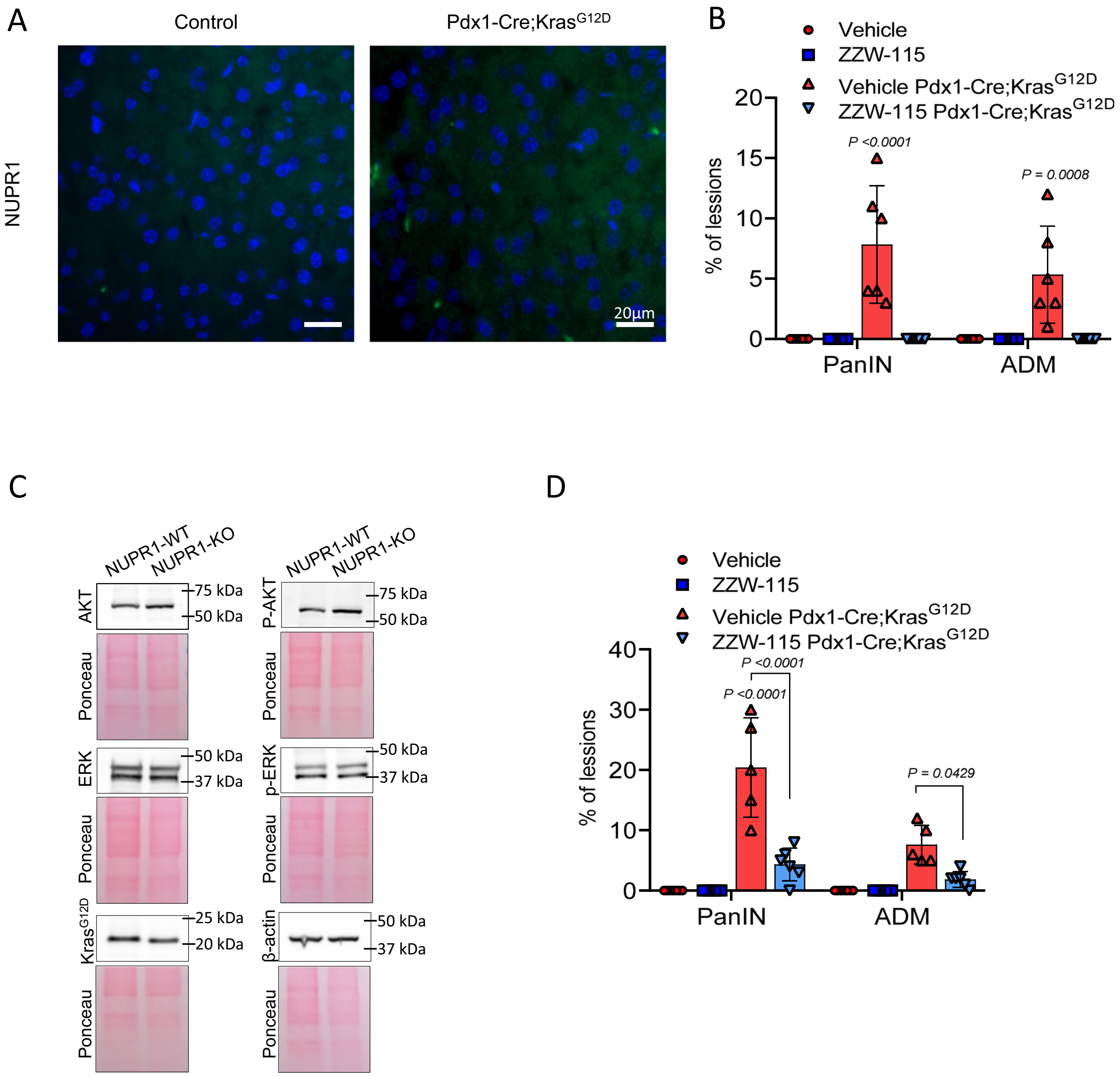

Figure EV5. NUPR1 inhibition induced cell death in vivo.

(**A**) Immunohistofluorescence staining was performed on histologic sections of the pancreas of the different experimental groups at 5 weeks of age. Rabbit anti-NUPR1 antibody was used, then, Alexa 488-labeled goat anti-rabbit ($n = 3$). (**B**) Percentage of tissue affected by ADM and PanIN lesions per tissue field in Control and Pdx1-Cre;$Kras^{G12D}$ mice treated with vehicle solution or ZZW-115 for ten weeks (from 5 to 15 weeks) ($n = 6$). Data represent mean ± SD, Two-way ANOVA with Sidak correction. (**C**) Western-blot analysis was performed in *Pdx1-cre;LSL-*$Kras^{G12D}$*/INK4a/*$Arf^{fl/fl}$*/*$NUPR1^{+/+}$ and *Pdx1-cre;LSL-*$Kras^{G12D}$*/INK4a/*$Arf^{fl/fl}$*/*$NUPR1^{-/-}$ mice cells to evaluate AKT, p-AKT, ERK, p-ERK, *Kras*^{G12D} or β-actin levels. (**D**) Percentage of tissue affected by ADM and PanIN lesions per tissue field in Control and Pdx1-Cre;$Kras^{G12D}$ mice treated with vehicle solution or ZZW-115 for four weeks (from 15 to 19 weeks) ($n = 5$). Data represent mean ± SD, Two-way ANOVA with Sidak correction. Source data are available online for this figure.
