# Supplementary material for: Targeting NUPR1-dependent stress granules formation to induce synthetic lethality in KrasG12D-driven tumors
Source: EMBO Mol Med. 2024 Feb 15;16(3):4. doi: 10.1038/s44321-024-00032-2 (PMC10940650; doi:10.1038/s44321-024-00032-2)
Supplement: Supplementary file 18 — Source Data EV Figure [file 44321_2024_32_MOESM18_ESM.zip › Figure EV3.zip!/Fig EV3 WB SG.pdf]

Figure EV3C

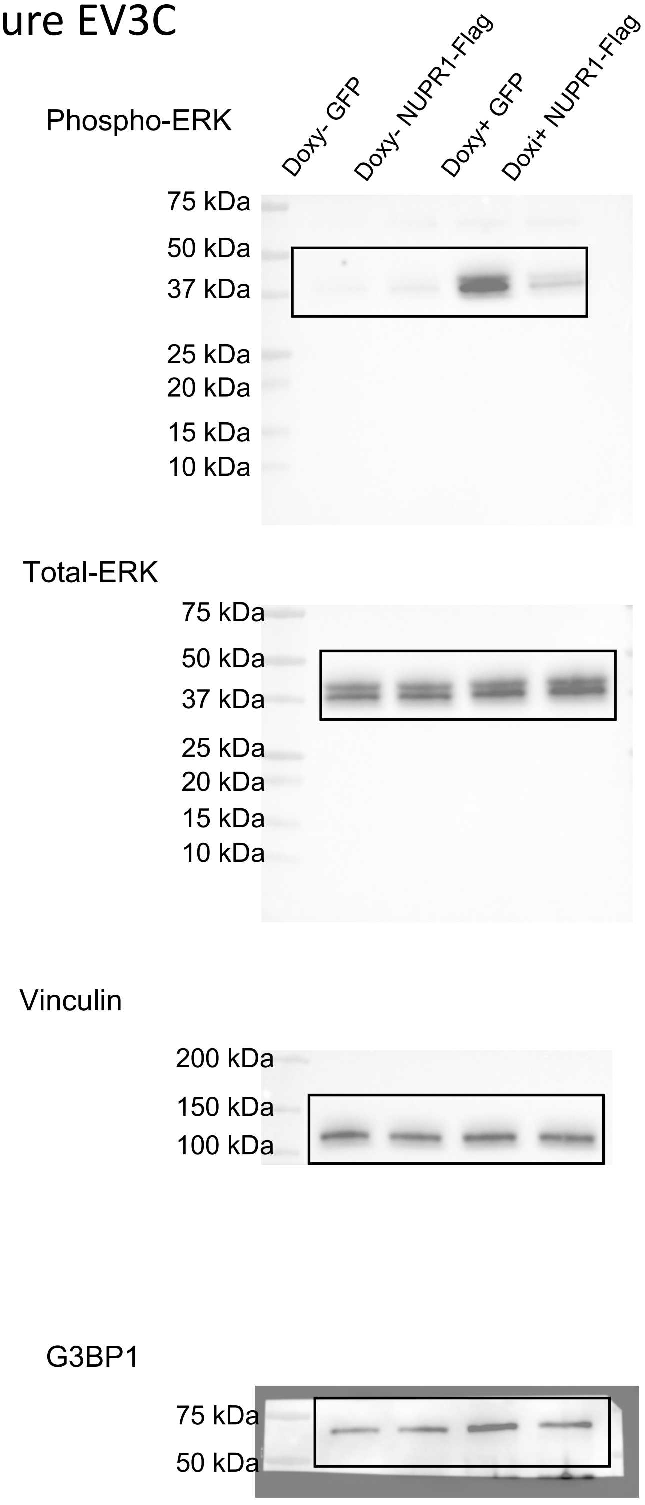
Doxy- GFP
Doxy- NUPR1-Flag
Doxy+ GFP
Doxi+ NUPR1-Flag
Phospho-ERK
75 kDa
50 kDa
37 kDa
25 kDa
20 kDa
15 kDa
10 kDa
Total-ERK
75 kDa
50 kDa
37 kDa
25 kDa
20 kDa
15 kDa
10 kDa
Vinculin
200 kDa
150 kDa
100 kDa
G3BP1
75 kDa
50 kDa
