# Supplementary material for: Targeting NUPR1-dependent stress granules formation to induce synthetic lethality in KrasG12D-driven tumors
Source: EMBO Mol Med. 2024 Feb 15;16(3):4. doi: 10.1038/s44321-024-00032-2 (PMC10940650; doi:10.1038/s44321-024-00032-2)
Supplement: Supplementary file 18 — Source Data EV Figure [file 44321_2024_32_MOESM18_ESM.zip › Figure EV4.zip!/Fig EV4 WB SG.pdf]

Figure EV4H

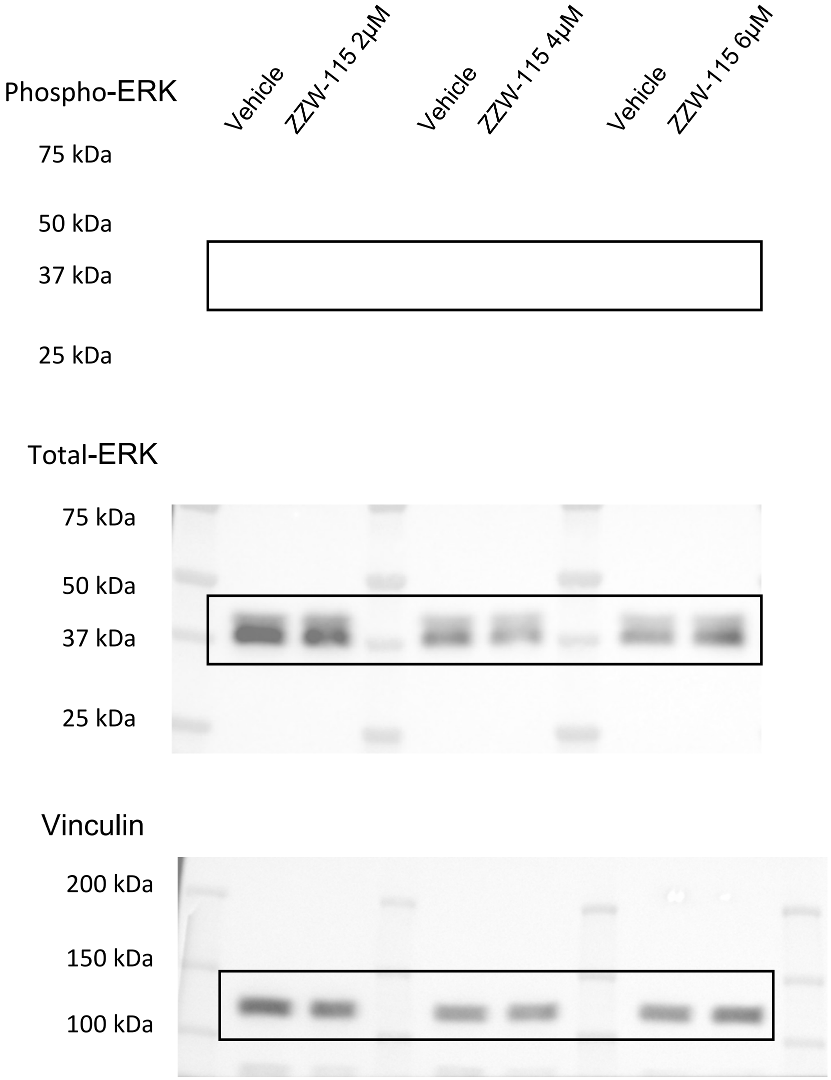
Vehicle
ZZW-115 2µM
Vehicle
ZZW-115 4µM
Vehicle
ZZW-115 6µM
Phospho-ERK
75 kDa
50 kDa
37 kDa
25 kDa
Total-ERK
75 kDa
50 kDa
37 kDa
25 kDa
Vinculin
200 kDa
150 kDa
100 kDa
